# Genome-wide association mapping for component traits of drought and heat tolerance in wheat

**DOI:** 10.3389/fpls.2022.943033

**Published:** 2022-08-16

**Authors:** Narayana Bhat Devate, Hari Krishna, Sunil Kumar V. Parmeshwarappa, Karthik Kumar Manjunath, Divya Chauhan, Shweta Singh, Jang Bahadur Singh, Monu Kumar, Ravindra Patil, Hanif Khan, Neelu Jain, Gyanendra Pratap Singh, Pradeep Kumar Singh

**Affiliations:** ^1^Division of Genetics, ICAR-Indian Agricultural Research Institute, New Delhi, India; ^2^Division of Genetics and Plant Breeding, ICAR-Indian Agricultural Research Institute, Gauria Karma, India; ^3^Genetics and Plant Breeding Group, Agharkar Research Institute, Pune, India; ^4^ICAR-Indian Institute of Wheat and Barley Research, Karnal, India

**Keywords:** genome-wide association study (GWAS), wheat, drought, heat, single nucleotide polymorphism (SNPs)

## Abstract

Identification of marker trait association is a prerequisite for marker-assisted breeding. To find markers linked with traits under heat and drought stress in bread wheat (*Triticum aestivum* L.), we performed a genome-wide association study (GWAS). GWAS mapping panel used in this study consists of advanced breeding lines from the IARI stress breeding programme produced by pairwise and complex crosses. Phenotyping was done at multi locations namely New Delhi, Karnal, Indore, Jharkhand and Pune with augmented-RCBD design under different moisture and heat stress regimes, namely timely sown irrigated (IR), timely sown restricted irrigated (RI) and late sown (LS) conditions. Yield and its component traits, *viz*., Days to Heading (DH), Days to Maturity (DM), Normalized Difference Vegetation Index (NDVI), Chlorophyll Content (SPAD), Canopy temperature (CT), Plant Height (PH), Thousand grain weight (TGW), Grain weight per spike (GWPS), Plot Yield (PLTY) and Biomass (BMS) were phenotyped. Analysis of variance and descriptive statistics revealed significant differences among the studied traits. Genotyping was done using the 35k SNP Wheat Breeder's Genotyping Array. Population structure and diversity analysis using filtered 10,546 markers revealed two subpopulations with sufficient diversity. A large whole genome LD block size of 7.15 MB was obtained at half LD decay value. Genome-wide association search identified 57 unique markers associated with various traits across the locations. Twenty-three markers were identified to be stable, among them nine pleiotropic markers were also identified. *In silico* search of the identified markers against the IWGSC ref genome revealed the presence of a majority of the SNPs at or near the gene coding region. These SNPs can be used for marker-assisted transfer of genes/QTLs after validation to develop climate-resilient cultivars.

## Introduction

Wheat (*Triticum aestivum* L.) is the staple food crop of one-third of the world population (Guo et al., 2018). To meet the food requirements, it has become mandatory to increase its production with limited resources. The development of high-yielding varieties became utmost important to meet the demand. Decades of breeding in wheat through conventional and molecular approaches enhanced the wheat productivity to ever-reached heights. Enormous improvement in the productivity of wheat has been attained by understanding the genetic principles and phenotypic evaluation and selection through conventional breeding methods (Pingali, 2012).

However, enhancing productivity under global climatic change is a challenging task. Abiotic stresses impact wheat production and tend to the shortage of food supply due to unpredictable crop loss. Among all the abiotic stresses curtailing wheat productivity, drought and heat are the most important and have detrimental effects (Zhang et al., 2018; Gajghate et al., 2020). The impact of drought and heat increased due to increment in global temperature and dry spells in arable land. The terminal drought that occurs at the time of grain filling will decrease the spike weight and the yield (Amiri et al., 2013; Saeidi and Abdoli, 2015). Hence, climate-resilient wheat cultivars are the ultimate means of safeguarding the crop against adverse effects of heat and drought and to fulfill future food needs.

An essential component of the Indian wheat improvement effort is breeding for resilience to abiotic stresses like drought and terminal heat. The interrelationship among breeding, molecular biology and physiology is the foundation of the breeding approach for drought and heat tolerance (Sukumaran et al., 2021), with an emphasis on precise dissection of morpho-physiological traits by precision phenotyping (Lopes et al., 2012). Physiological traits like Canopy Temperature (CT) and grain yield under drought stress environments have a well-established relationship (Gautam et al., 2015; Rehman et al., 2021; Munawar et al., 2022; Singh et al., 2022), and drought and heat tolerance are correlated with lower canopy temperature (CT) (Pinto et al., 2010). Maintenance of lower canopy temperature indirectly attributes to higher transpiration and deep root system in the variety to absorb water from the deeper soil horizon. CT influences the stay-green of leaves, a drought-adaptive trait characterized by a distinct green leaf phenotype during grain filling during terminal drought (Borrell et al., 2014). Drought and heat stress have a significant impact on leaf chlorophyll content (Barboričová et al., 2022). The level of chlorophyll in flag leaves is measured by a portable equipment (SPAD meter), which is thought to be a sign of ‘stay-green' or delayed senescence (Lopes et al., 2012). In the post-anthesis phase, “Stay-green” is reported to be associated with drought tolerance in wheat (Kumar et al., 2021) and is utilized for breeding drought tolerant varieties in wheat along with NDVI (normalized difference vegetative index) (Christopher et al., 2015; Rutkoski et al., 2016; Singh et al., 2016). In crop canopies, NDVI is sensitive to biomass and nitrogen (N) variability, both of which are regulated by stress experienced by the crop. Tools for proximal canopy sensing, like Green Seeker (Trimble Navigation Limited, Sunnyvale, California, USA), can detect reflected light from the crop canopy to capture vegetation indices, such as the simple ratio or the NDVI (Naser et al., 2020). For high throughput screening of drought and heat tolerance in wheat, CT, chlorophyll content (SPAD) and NDVI have been successfully integrated into breeding programmes (Singh et al., 2016; Phuke et al., 2020).

Drought and heat tolerance are complex traits that are influenced by a number of genes and have a complex genetic inheritance (Phuke et al., 2020; Saini et al., 2022). Due to its polygenic inheritance and genotype by environment interaction, drought and heat tolerance typically has low heritability (Blum, 2010; Khakwani et al., 2012). Genetic improvement under abiotic stress can be achieved by identifying sources of stress-tolerant traits, as well as introgress, and mobilize the genes underlying the desired traits into locally adapted cultivars (Edae et al., 2014). The challenges in implementing this method in breeding programmes determine the most appropriate target traits for various stress scenarios in a timely and cost-effective manner (Passioura, 2012). Recent advances in high-throughput genotyping and phenotyping have increased our understanding of the physiological and genetic basis of complex characteristics like drought (Mir et al., 2012; Sinclair, 2012) and heat tolerance (Paliwal et al., 2012). One of the most important tools for understanding the genetic architecture of complex characteristics in plants is QTL mapping (Holland, 2007; Xu et al., 2017). However, QTL mapping utilizing biparental populations can only explain a limited percentage of a trait's genetic architecture. Low-mapping resolution, population specificity of discovered QTL and the requirement of a long time to establish mapping populations are further constraints of biparental populations (Edae et al., 2014).

Precise improvement of the complex quantitative trait for adaption to the particular environmental condition like drought and heat needs identification of related genomic regions like QTLs. For the identification of genes/QTLs based on the linkage disequilibrium (LD), GWAS is one of the effective methods. For the prediction of candidate genes, GWAS has been widely used in several crops using genome-wide dense markers (Liu et al., 2018; Srivastava et al., 2020; Alseekh et al., 2021; Tiwari et al., 2022), including wheat (Negisho et al., 2022; Zhang et al., 2022). Advantages of GWAS is, QTLs for several traits can be found with high resolution in one go. Since association mapping uses diverse germplasm, making the procedure more efficient and less expensive than bi-parental QTL mapping (Ersoz et al., 2009; Jin et al., 2016). The resolution and power of association studies, however, depend on the extent of linkage disequilibrium (LD) across the genome. LD needs to be determined in each study as it is affected by several factors, such as population history, recombination frequency and mating system (Edae et al., 2014).

Association mapping has been used successfully to detect marker trait associations and QTLs in wheat for various traits. There are few studies targeting traits under abiotic stress (Sukumaran et al., 2018; Li H. et al., 2019; Shokat et al., 2020; Abou-Elwafa and Shehzad, 2021; Ahmed A. A. et al., 2021; Ahmed et al., 2022a). High-density SNPs markers used for genome-wide association study (GWAS) can inspect large gene pools representative of diverse breeding reservoirs. GWAS is the most suitable approach to locate robust QTLs that show effect in both normal and stressed conditions (Jamil et al., 2019; Ahmed H. G. M. D. et al., 2021; Saini et al., 2022). Hence, genome-wide association studies (GWAS) have developed into a powerful and ubiquitous tool for the investigation of complex traits (Tibbs Cortes et al., 2021).

In the Indian context, improving wheat cultivars for drought and heat stress resistance is critical for the country's food security. In this study, SNPs markers associated with component traits of drought and heat tolerance were mapped in the advanced breeding lines of Indian hexaploid wheat with the genome-wide markers using the Axiom Wheat Breeder's Genotyping Array (Affymetrix, Santa Clara, CA, United States) having 35,143 SNPs and well suited for high-throughput genotyping in hexaploid wheat (Allen et al., 2017). The aim here is to identify the genomic regions related to yield- and stress-related traits under multi-location stress conditions.

## Materials and methods

### Plant material

The association panel under study was constituted by pairwise and multi-parent crosses of the selected Indian varieties, cultivars, superior breeding lines and exotic introductions. The crosses were advanced with the modified bulk pedigree method and each line was maintained with the respective pedigree record (Supplementary Table 1), and finally, 295 diverse advanced lines were used in the current investigation. However, 282 lines were retained after matching with the obtained genotyping data for further analysis.

### Phenotyping

The phenotypic evaluation was conducted at multiple locations, namely, IARI, New Delhi—DL (28.6550° N, 77.1888° E, MSL 228.61 m), ARI, Pune—PUNE (18.5204° N, 73.8567° E, MSL 560 m), IIWBR, Karnal—IIWBR (29.6857° N, 76.9905°E, MSL 243 m), IARI, Jharkhand—JR (24.1929° N, 85.3756° E, MSL 580 m) and IARI RS, Indore—IND (22.7196° N, 75.8577° E, MSL 553 m). Irrigated trials (IR) were conducted in *rabi* season in 2019 in Delhi and in 2020 in Delhi, Karnal and Pune. Whereas Restricted irrigation (RI) trials were conducted in 2019 in Delhi and in 2020 in Delhi, Indore, Pune and Jharkhand. LS trial was conducted only in IARI New Delhi in the years 2019 and 2020 (Table 1). A total of six irrigations were given for irrigated trials, whereas only 1 irrigation was given (21 days after sowing besides pre-sowing irrigation) in restricted irrigation trials to induce the terminal drought stress. Heat stress was induced by sowing the crop (Late sown—LS) in the second fortnight of December. The experiment was planned with an augmented RCBD design (Federer, 1956, 1961; Searle, 1965) with 295 genotypes and 4 checks replicated twice in 6 blocks. Lines and checks were randomized and sown in plots in three lines of 1 m with a 30 cm inter-line distance. All agronomic practices were carried out according to the recommended package of practices at each location.

**Table 1:** Details of sowing conditions, locations and year of experiment along with abbreviations.

**Treatment**	**Location**	**GPS location**	**Year**	**Abbreviation**
Irrigated–(IR)	ICAR-Indian Agricultural Research Institute (IARI)-New Delhi	28.6550° N, 77.1888° E, MSL 228.61 m	2019	DL_IR_2019
			2020	DL_IR_2020
	Agharkar Research Institute-ARI, Pune	18.5204° N, 73.8567° E with MSL 560 m	2020	PUNE_IR_2020
	Indian Institute of Wheat and Barley Research-IIWBR, Karnal	29.6857° N, 76.9905°E, MSL 243 m	2020	IIWBR_IR_2020
Restricted irrigated–(RI)	ICAR-Indian Agricultural Research Institute (IARI)-New Delhi	28.6550° N, 77.1888° E, MSL 228.61 m	2019	DL_RI_2019
			2020	DL_RI_2020
	Agharkar Research Institute-ARI, Pune	18.5204° N, 73.8567° E with MSL 560 m	2020	PUNE_RI_2020
	ICAR-Indian Agricultural Research Institute, Regional station-IARI RS, Indore	22.7196° N, 75.8577° E, MSL 553 m	2020	IND_RI_2020
	ICAR-Indian Agricultural Research Institute-IARI, Jharkhand	24.1929° N, 85.3756° E, MSL 580 m	2020	JR_RI_2020
Late Sown–(LS)	ICAR-Indian Agricultural Research Institute (IARI)-New Delhi	28.6550° N, 77.1888° E, MSL 228.61 m	2019	DL_LS_2019
			2020	DL_LS_2020

The standard procedure for data collection was followed as given in the manual ‘Wheat Physiological Breeding II: A Field Guide to Wheat Phenotyping' (Pask et al., 2012). Data were collected for traits like Days to heading (DH), Days to maturity (DM), Normalized Difference Vegetation Index (NDVI) at anthesis and grain filling stage, chlorophyll content (SPAD) of flag leaf at the post-anthesis stage, Plant height (PH), Canopy temperature (CT), Grain weight per spike (GWPS), Thousand Grain weight (TGW), Plot Yield (PLTY) and Biomass.

### Genotyping

DNA isolation was carried out using the leaves of 7-days-old seedlings grown under controlled conditions. DNA was extracted using the CTAB method (Murray and Thompson, 1980) with minor modifications. DNA quality was checked with the 0.8% agarose gel electrophoresis and 20 ng/μl DNA was used for further SNP genotyping. Out of 295 DNA samples, 282 passed the quality check step and were used for further genotyping. The remaining 13 genotypes were excluded from genotyping due to poor DNA quality. Hybridisation-based SNP chip genotyping of 282 genotypes was performed using the 35K Axiom^®^ Wheat Breeder's Array of Affymetrix GeneTitan^®^ system according to the procedure described by Affymetrix. Allele calling was carried out using the Affymetrix proprietary software package Axiom Analysis Suite, following the Axiom^®^ Best Practices Genotyping Workflow (https://media.affymetrix.com/support/downloads/manuals/axiom_analysis_suite_user_guide.pdf). SNP marker data were obtained in the Hap Map format. The chip had 35,143 SNPs; however, after filtration for monomorphic alleles, minor allele frequency (MAF) was >0.05, missing data frequency was >0.2 and heterozygote frequency >0.25; a total of 10,546 SNPs were retained for GWAS analysis.

### Analysis of data

Phenotypic data at each location and condition (IR, RI and LS) were analyzed using the r package ‘augmentedRCBD' (Aravind et al., 2021) for ANOVA, and adjusted means for each genotype under study were estimated based on Federer (1956, 1961). Calculated adjusted mean eliminating block effect at each environment is used further in all the analyses, including GWAS. The adjusted mean of each block was calculated with the formula (Federer, 1961):


$$\begin{array}{l} Vi=ui-bj \end{array}$$

where

Vi is the adjusted mean of *i*^*th*^ variety

*ui* is the unadjusted mean of *i*^*th*^ variety

*bj* is *j*^*th*^ block effect.

Principal component analysis was carried out using the R package “FactoMineR version 2.4” (Multivariate Exploratory Data Analysis and Data Mining) by Husson et al. (2016). Graphical representation of PCA results was done with the R package “factoextra version 1.0.7” (Kassambara, 2020). Pearson's correlation was calculated among the studied traits and figures were drawn.

A total of 5,480 SNPs equally spaced around 1 MB distance throughout the genome were filtered and used for the estimation of population structure using the STURUCTURE software (Pritchard et al., 2010). The parameters, *viz*., burn-in cycles and MCMC (Monte Carlo Markov Chains) were set to 100,000. Three iterations for each *k* value ranging from 1 to 7 were conducted to determine the population structure. The ideal number of delta K (subpopulations) was found out by the Evanno method (Evanno et al., 2005) using Structure Harvester (http://taylor0.biology.ucla.edu/structureHarvester/). Furthermore, the filtered 10,546 SNPs were analyzed using TASSEL 5.0 (Trait Analysis by Association, Evolution and Linkage) (Bradbury et al., 2007) to construct the neighbor-joining dendrogram. SNP marker-based PCA and Kinship analysis were conducted with GAPIT (Lipka et al., 2012).

### Association analysis

The filtered SNP markers were utilized for determining marker-trait associations using TASSEL v 5.0. The *r*^2^ values between marker pairs were obtained and filtered for pairs within each chromosome. LD decay curve was drawn for each A, B and D genomes along with whole the genome. LD block size was estimated by plotting the *r*^2^ value against the distance in base pairs (bp) and the distance at the half LD Decay point was noted.

The filtered 10,546 SNPs and the location-wise “adjusted mean” for each trait were used for the genome-wide association analysis using GAPIT v3 in R with PCA 3 and default parameters (https://zzlab.net/GAPIT/gapit_help_document.pdf) using the “BLINK” (Bayesian-information and Linkage-disequilibrium Iteratively Nested Keyway) model. The BLINK model is presumed to be superior in identifying QTNs and avoiding false positives to decipher true associations (Huang et al., 2019). Quality of association model fitting was found out using a Q-Q plot drawn with expected vs. observed −*log10(p)* value. Stringent selection of MTAs was done using the Bonferroni correction (*p*-value cut-off at 0.05/total number of markers) to avoid false positives and a Manhattan plot was drawn to represent MTAs. Stable MTAs across the location were found out with a significant *p*-value cut-off at 0.001. Pleiotropic SNPs having association with more than one trait were also found out. Stable and pleiotropic SNPs were compared with the IWGSC Reference genome using BLAST search in the ensemble plants platform (http://plants.ensembl.org/Triticum_aestivum/Tools/Blast). To identify the candidate genes associated with significant SNPs, gene coding regions located within the 10 KB flanking region of the MTAs were considered.

## Results

### Phenotypic evaluation

There was high variability in means and range for each trait at the respective location. Mean DH and PLTY were highest in Delhi as compared to other locations, and Pune had the least. Late sown trials planted for heat stress had lower DH, DM, PH, GWPS, TGW, BIOMASS and PLTY as compared to IR and RI trials (Table 2). NDVI had a similar pattern across the environments except in the late sown where it had lower NDVI values. CT at anthesis was highest in RI trials followed by LS and IR. Jharkhand and Indore locations displayed higher TGW as compared to Delhi. IR trials at Delhi had higher TGW compared to RI and LS due to shrinkage of grains under drought and heat stress (Table 2). Descriptive statistics like minimum, maximum, average, standard deviation, coefficient of variation and critical difference of all the studied traits in each environment were calculated (Supplementary Table 2).

**Table 2:** A summary of yield and stress related traits in GWAS panel evaluated across the different environment.

**Condition**	**Environment**	**DH**	**PH**	**DM**	**NDVI_1**	**NDVI_2**	**NDVI_3**	**SPAD**	**CT**	**GWPS**	**BIOMASS**	**PLTY**	**TGW**
**IR**	DL-2019	94.1 ± 6.32 (79.24–112.76)	107.48 ± 7.21 (75.06–128.06)	–	0.8 ± 0.01 (0.71–0.82)	0.7 ± 0.35 (0.54–6.66)	0.71 ± 0.04 (0.56–0.81)	–	–	2.29 ± 0.74 (0.49–4.48)	–	350.96 ± 75.79 (75.12–477.56)	–
	PUNE-2020	63.12 ± 3.78 (51.02–72.02)	–	–	0.86 ± 0.04 (0.71–0.96)	0.83 ± 0.04 (0.69–0.94)	–	–	26.91 ± 1.17 (24.07–29.98)	1.47 ± 0.13 (1.05–1.82)	–	186.5 ± 45.68 (41.45–321.8)	42.99 ± 3.52 (31.83–50.36)
	DL-2020	94.99 ± 6.45 (75.21–113.96)	108.12 ± 6.25 (87.91–125.12)	133.23 ± 3.84 (123.81–144.81)	0.68 ± 0.05 (0.54–0.8)	0.59 ± 0.06 (0.43–0.74)	0.21 ± 0.09 (0.06–0.5)	46.66 ± 4.02 (33.76–56.84)	27.46 ± 1.48 (23.19–30.68)	1.98 ± 0.44 (0.71–4.06)	1661.34 ± 332.87 (337.62–2775.25)	484.53 ± 103.21 (101.77–708.65)	38.82 ± 4.43 (24.16–57.28)
	IIWBR-2020	88.08 ± 2.51 (81.9–100.02)	–	123.56 ± 1.66 (118.98–127.98)	0.6 ± 0.09 (0.26–0.82)	–	–	–	–	2.07 ± 0.32 (1.32–3.09)	–	261.68 ± 83.68 (46.55–485.27)	36.83 ± 5.03 (23.63–53.33)
**LS**	DL-2019	93.3 ± 2.99 (85.5–100.5)	–	–	0.81 ± 0.02 (0.71–0.85)	0.96 ± 4.14 (0.55–72.01)	0.4 ± 0.12 (0.07–0.72)	–	21.97 ± 0.87 (19.91–24.51)	1.39 ± 0.31 (1.1–2.75)	–	291.41 ± 64.32 (80.69–441.52)	–
	DL-2020	82.99 ± 2.7 (77.69–93.44)	87.23 ± 6.31 (69.4–103.98)	109.41 ± 3.05 (101.44–120.06)	0.57 ± 0.05 (0.38–0.72)	0.42 ± 0.07 (0.22–0.6)	0.2 ± 0.07 (0.09–0.56)	49.75 ± 4.32 (36.55–60.79)	29.01 ± 1.39 (26.27–39.02)	1.68 ± 0.3 (0.95–2.9)	995.87 ± 344.44 (55.9–2267.6)	340.12 ± 110.94 (53.88–597.63)	36.28 ± 4.06 (23.15–48.15)
**RI**	DL-2019	94.38 ± 6.49 (75.5–107.5)	–	–	0.8 ± 0.01 (0.71–0.83)	0.65 ± 0.04 (0.53–0.76)	–	–	–	2.49 ± 0.56 (1.14–4.54)	–	–	–
	PUNE-2020	58.36 ± 2.95 (50.48–68.48)	–	–	0.85 ± 0.05 (0.68–0.97)	0.81 ± 0.04 (0.68–0.97)	–	–	27.93 ± 1.06 (25.43–30.24)	1.66 ± 0.15 (1.23–2.01)	–	92.31 ± 33.67 (19.04–215.16)	40.85 ± 3.45 (31.13–49.01)
	JR-2020	78.06 ± 3.52 (61.53–87.53)	90.61 ± 7.08 (64.74–112.74)	112.18 ± 3.99 (101.67–124.94)	–	–	–	–	–	2.2 ± 0.34 (0.9–3.21)	320.49 ± 98.54 (42.75–627.75)	163.85 ± 48.18 (13.48–286)	43.38 ± 3.87 (29.47–58.86)
	DL-2020	97.84 ± 6.37 (77.71–115.46)	101.41 ± 6.62 (77.47–119.97)	130.43 ± 3.46 (120.52–140.4)	0.64 ± 0.05 (0.51–0.82)	0.53 ± 0.05 (0.38–0.67)	0.21 ± 0.08 (0.07–0.52)	52.31 ± 4.71 (35.34–62.24)	32.54 ± 1.17 (29.51–36.21)	1.74 ± 0.36 (0.85–3.2)	1199.13 ± 321.87 (30.2–1937.15)	300.78 ± 86.3 (34.58–560.21)	32.99 ± 4.91 (20.98–50.1)
	IND-2020	–	–	–	–	–	–	–	–	–	–	301.16 ± 64.6 (82.73–466.83)	44.38 ± 3.15 (36–53.62)

*DL-Delhi, PUNE-Pune, IIWBR-Karnal, JR-Jharkhand, IND-Indore. IR, Irrigated; RI, Restricted irrigated; LS, Late sown.

**(Mean ± SD) with (Min–Max) values are given in brackets for each trait.

***DH, Days to Heading; PH, Plant Height; DM, Days to maturity; NDVI – Normalized difference vegetation index, SPAD, Chlorophyll content; CT, Canopy Temperature; GWPS, Grain Weight per Spike; BIOMASS – Biomass; PLTY, Plot yield; TGW, Thousand grain weight.

**Figure 1:**
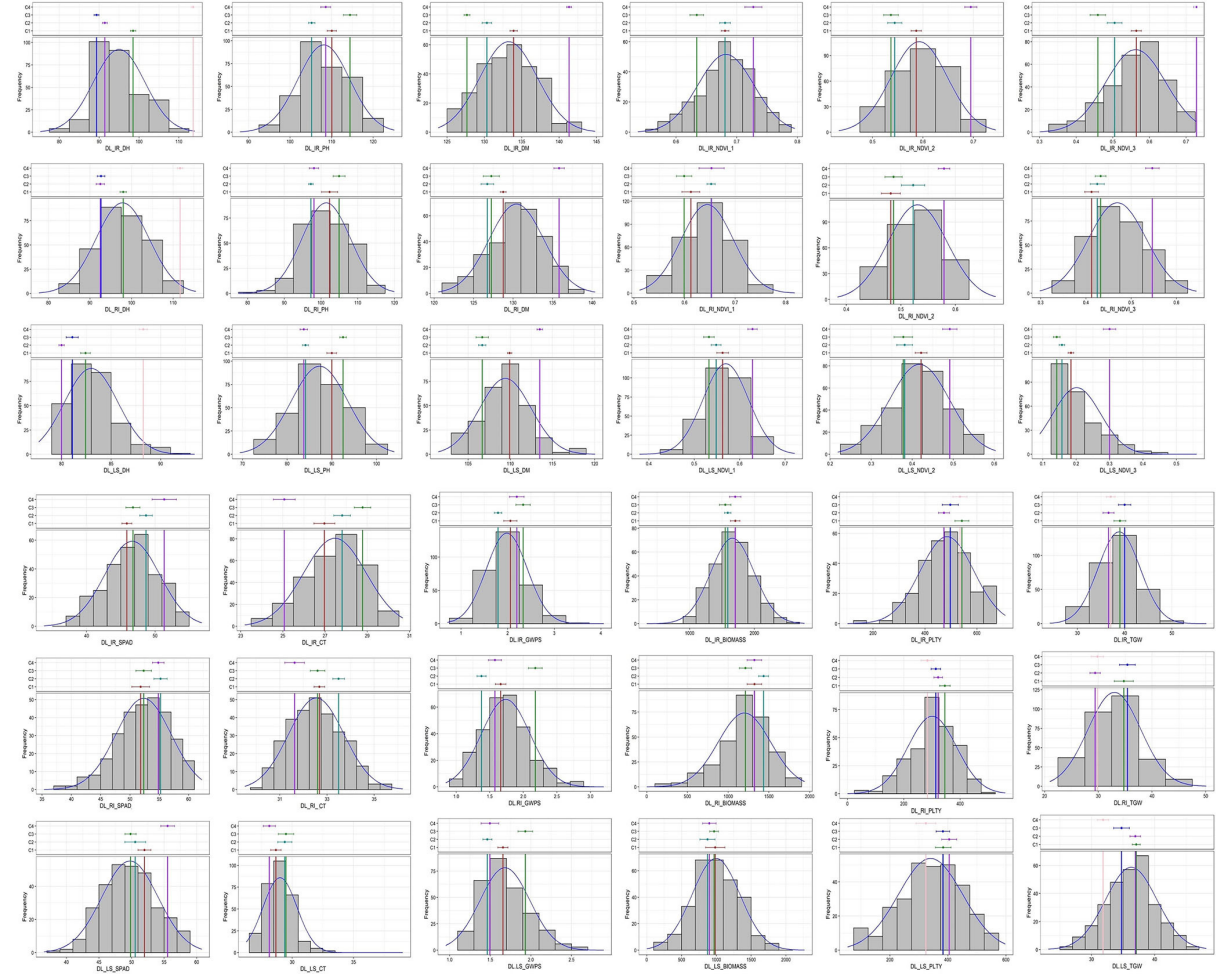
Frequency distribution of studied traits in GWAS panel evaluated at IARI Delhi under three conditions *viz*., IR, RI and LS during 2020–2021.

Analysis of variance indicated a significant difference between the studied traits at 5% and 1% as noted in Supplementary Table 2. The frequency distribution curve indicated the near normal distribution for the majority of the traits in Delhi under all three conditions and a similar pattern was observed at other locations too (Figure 1; Supplementary Figure 1). There was the least variation for days to heading in Delhi under irrigated condition (0.78%) followed by NDVI_1 (0.89%) at the late sown condition in Delhi. Being high environmental responsive traits, plot yield, biomass, NDVI_3 and GWPS were the highly variable traits having higher CV values compared to other traits in all the locations.

### Correlation- and phenotype-based PCA

**Figure 2:**
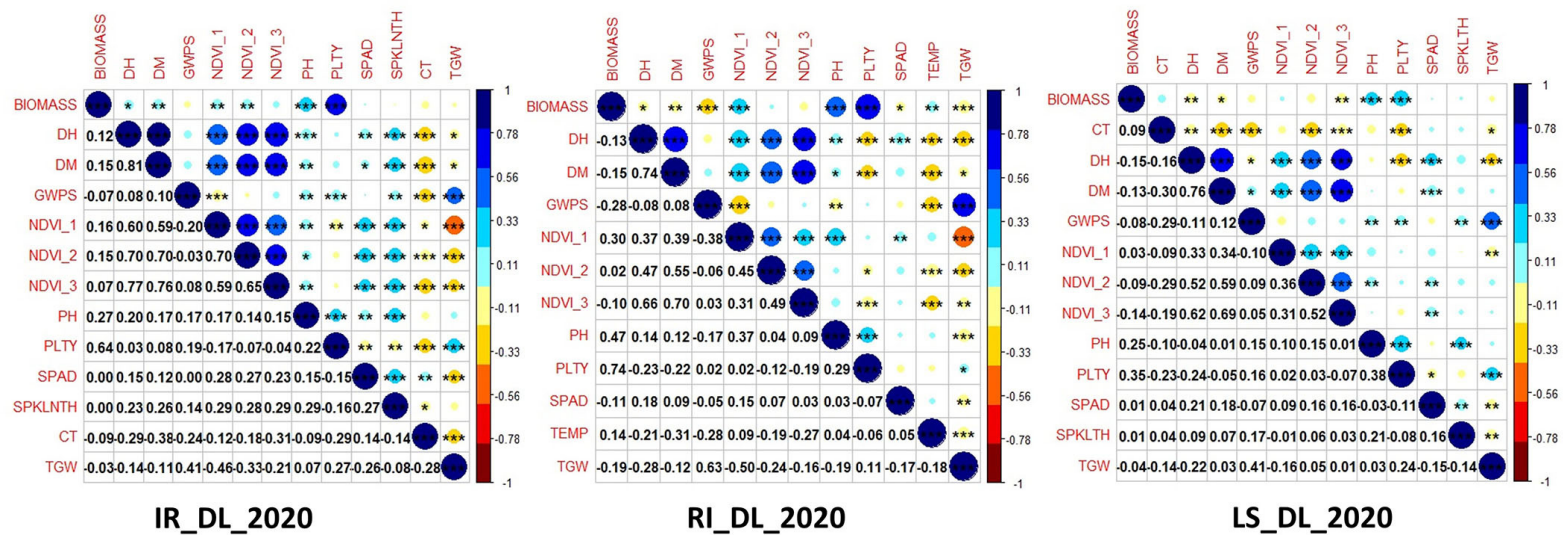
Correlation among the studied traits in the GWAS panel evaluated at IARI Delhi under three conditions, *viz*., IR, RI and LS during 2020–2021.

There was a positive correlation among the traits like NDVI, SPAD, DH, DM and PH in all the three conditions at the Delhi location and a similar trend was observed in all other locations. Similarly, yield-related traits like GWPS, TGW and PLTY also showed a positive correlation across the studied locations and treatment conditions. CT was negatively correlated with all the studied traits (Figure 2; Supplementary Figure 2).

**Figure 3:**
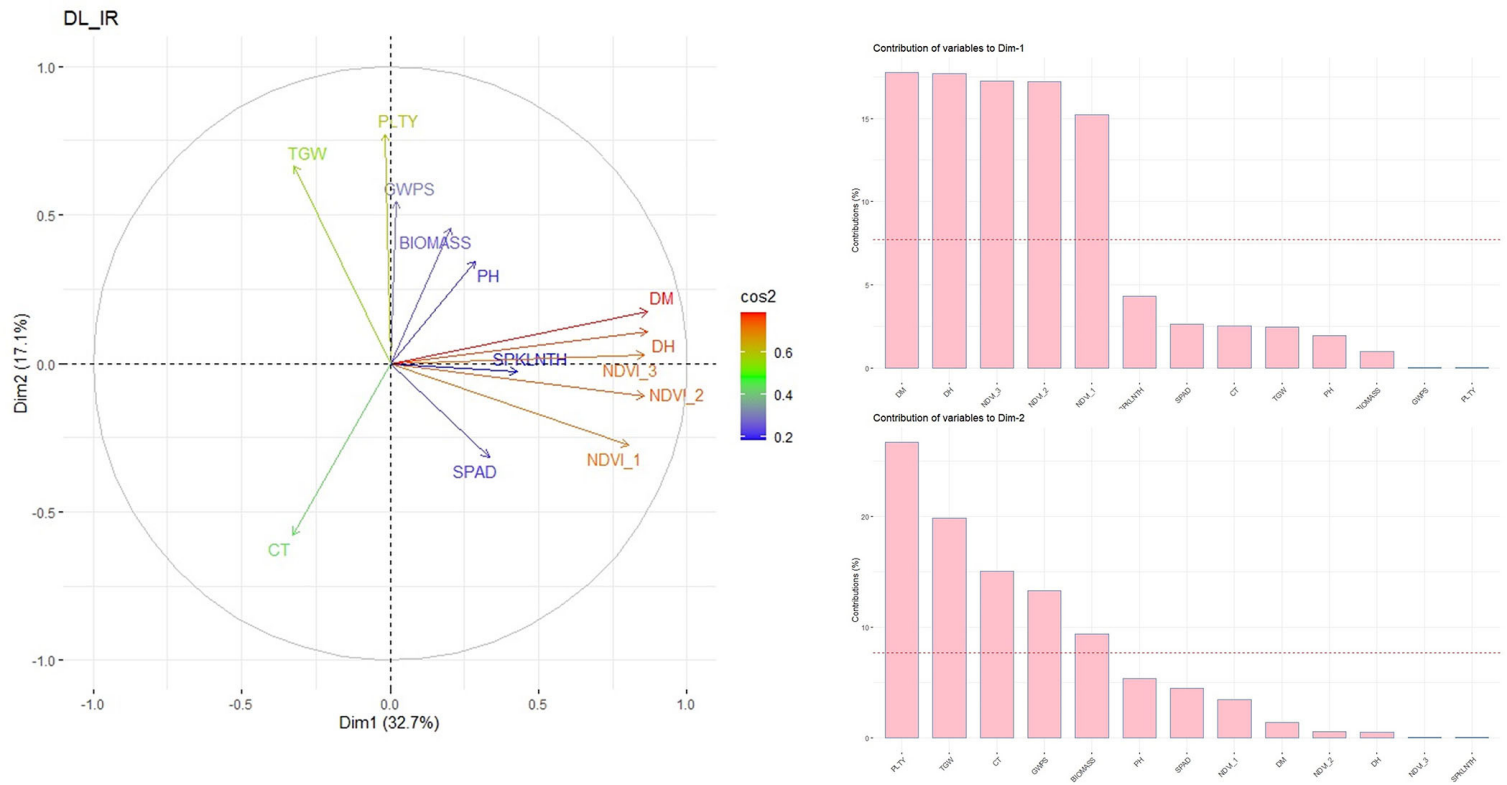
PCA biplot and contribution of studied traits to dimension 1 and dimension 2 in GWAS panel evaluated at IARI Delhi under IR condition during 2020–2021.

PCA based on phenotypic data indicated that in Delhi irrigated condition, the first principal component was contributing 32.7% variation; the major contributors were DM, DH and NDVI (Figure 3), whereas the second dimension represents 17.2% variation receiving contribution from yield-related traits, *viz*., PLTY, TGW, CT, GWPS and BIOMASS (Figure 3). The traits DH, DM and NDVI are clustered together with an acute angle indicating a positive correlation among them. Similarly, TGW, PLTY, GWPS and BIOMASS were clustered together. Whereas CT indicated a negative correlation to all the studied traits. Similarly, PCA analysis under RI and LS conditions at Delhi indicated that dimension 1 was explaining 28.6 and 25.3%; dimension 2 was explaining 21 and 15.6% variation, respectively. Variation explained by both the dimension (i.e. Dim-1 and Dim-2) of PCA from other locations were also found out using PCA analysis viz., IIWBR (Dim1-31.8%, Dim2-25%), PUNE_IR (Dim1-25.3%, Dim2-19.5%), PUNE_RI (Dim1-20.9%, Dim2-18.6%) and JR (Dim1-37.2%, Dim2-21.9%) locations (Supplementary Figure 3).

### Genotyping

Out of 35,143 SNPs screened over 282 genotypes, 10,546 SNPs were retained after filtering. Genome-wide SNP distribution analysis showed 3,350, 4,083 and 3,113 SNPs in A, B and D genomes, respectively. Having 777 SNPs, 2B was the chromosome containing the highest number of polymorphic SNPs followed by 2D having 767. Chromosome 4D had the lowest number of polymorphic SNPs (184) (Table 3).

**Table 3:** Chromosome wise distribution of SNP markers on the three sub genomes of wheat.

	**Sub genome**		
**Chromosome**	**A**	**B**	**D**
1	489	705	616
2	628	777	767
3	432	499	348
4	397	299	184
5	477	652	433
6	404	570	323
7	523	581	442
Total	3,350	4,083	3,113

### Population structure and diversity

**Figure 4:**
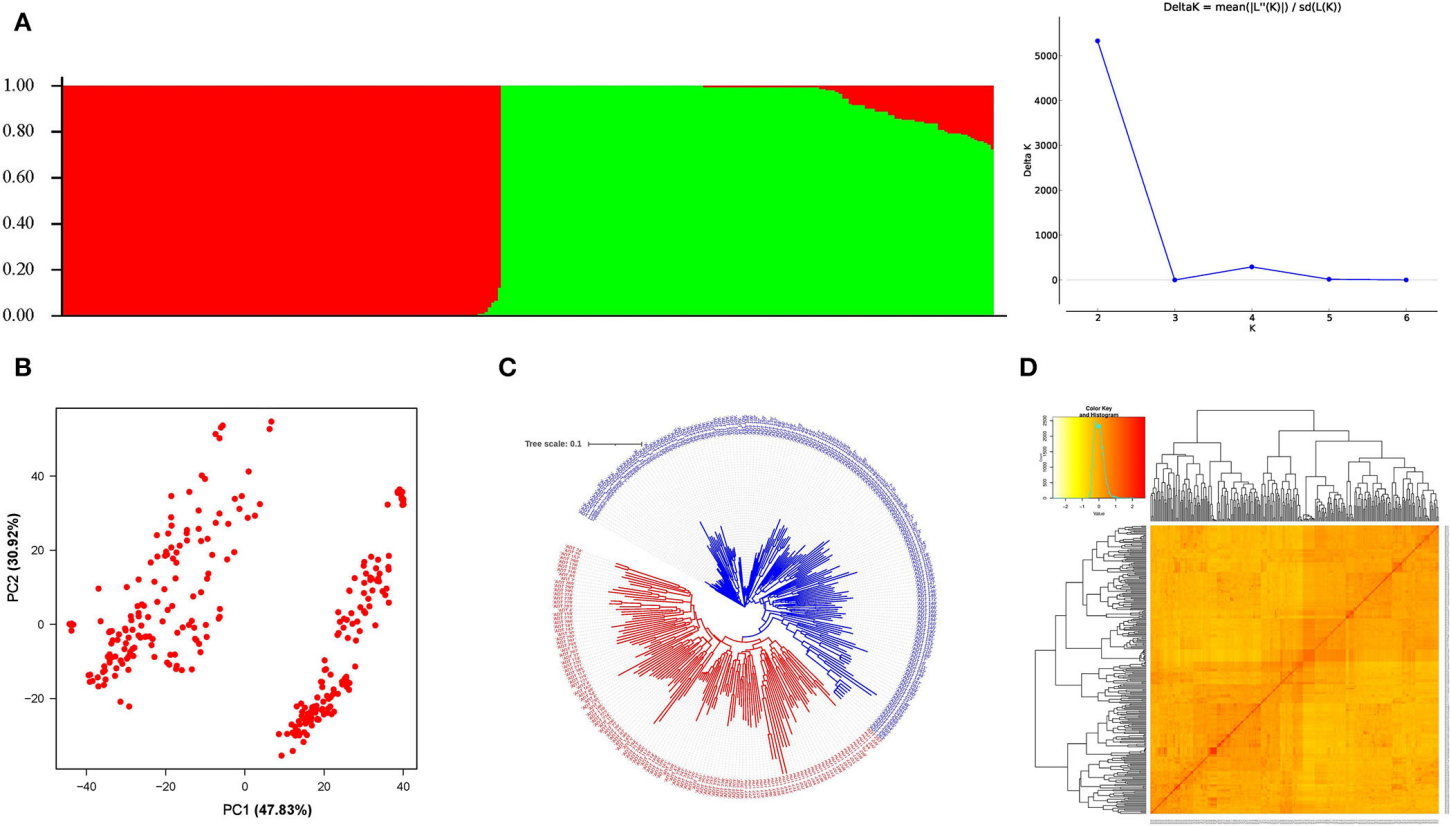
Population groupings in the GWAS panel from different models. **(A)** Population structure-based grouping of genotype from STUCTURE analysis. **(B)** The 2D plot of the principal component-based grouping. **(C)** Neighbor-joining tree-based diversity. **(D)** Heat map of pair-wise kinship matrix.

Population structure was determined with burn-in and MCMC of 100,000 with three iterations using STRUCTURE HARVESTER. The best *K*-value obtained was 2 indicating 2 subpopulations in the GWAS panel (Figure 4A). The subpopulations 1 and 2 had 133 and 135 genotypes, respectively, whereas 14 genotypes were considered as the admixtures population (Supplementary Table 3). The result was verified with the PCA analysis based on SNP marker data; two clusters were also obtained in a marker-based PCA plot (Figure 4B), indicating two subpopulations in the panel. Kinship and neighbor-joining cluster analysis verified the presence of two clusters as shown in Figures 4C,D.

### Linkage disequilibrium (LD) block

**Figure 5:**
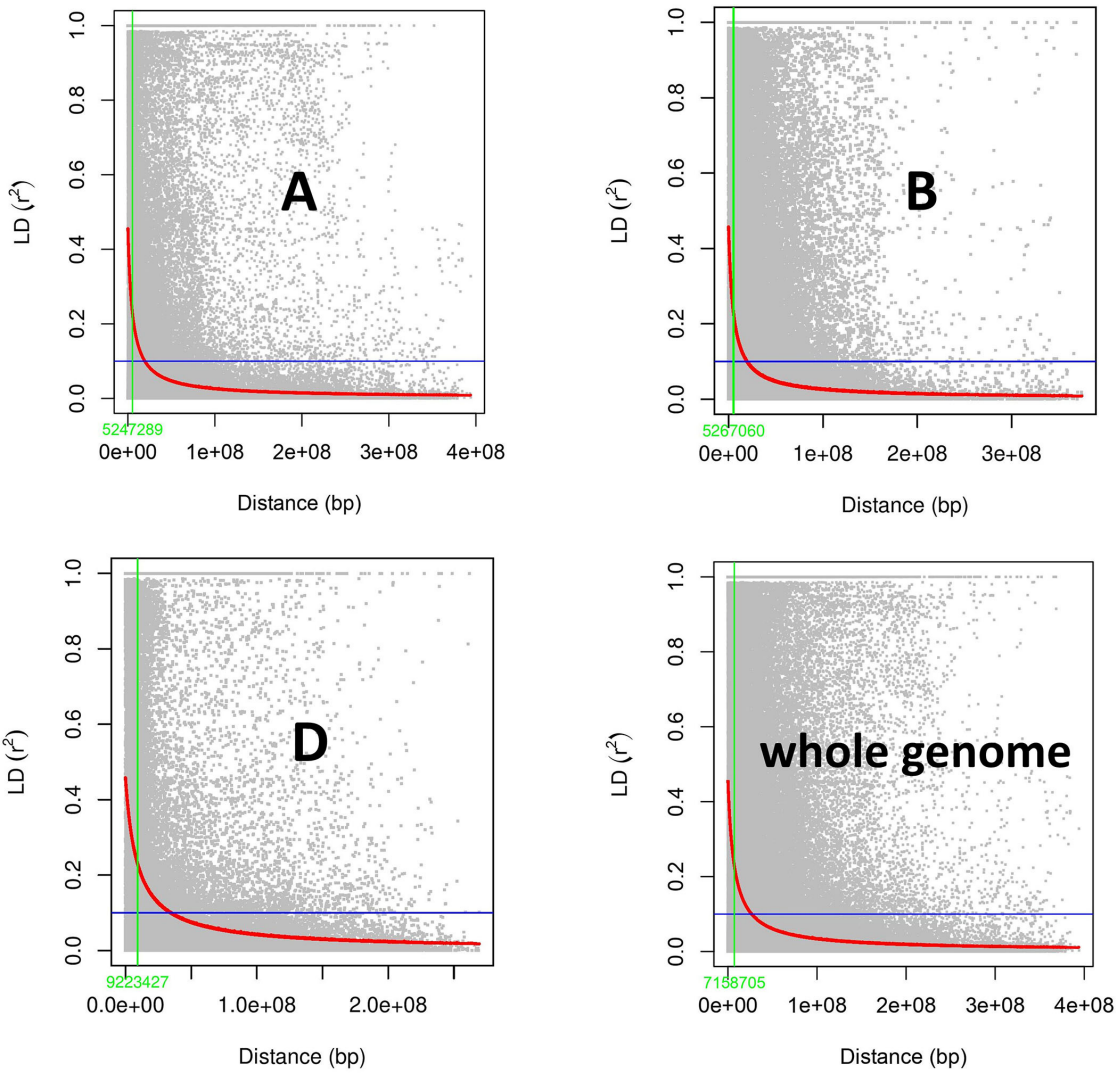
Subgenome and whole genome-wide linkage disequilibrium (LD) decay in GWAS panel of 282 diverse bread wheat genotypes.

To estimate LD, the *r*^2^ (squared allele frequency correlation) value was calculated among all the possible pairs of SNPs in each chromosome using TASSEL 5.0 (Bradbury et al., 2007). LD decay map was constructed by plating *r*^2^ values against genetic distance in bp for each genome and whole genome. LD block size at half LD decay was 5.24, 5.26, and 9.22 MB for A, B and D genomes, respectively (Figure 5). Whole genome LD decay was observed to be 7.15 MB, indicating any SNPs within this distance are said to behave as inheritance block.

### Marker trait associations (MTAs)

**Figure 6:**
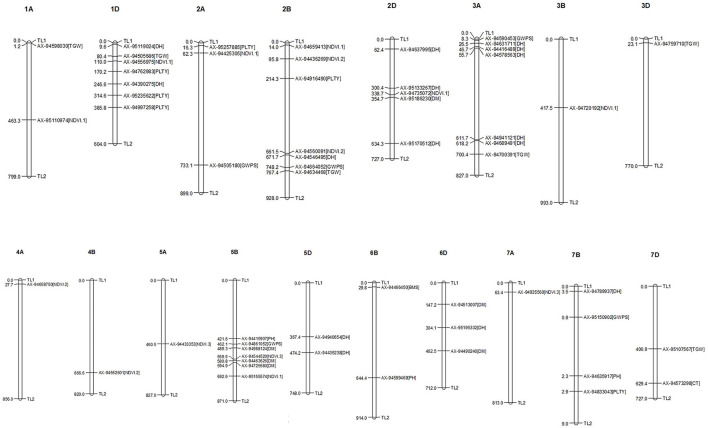
Distribution and position of identified MTAs (*-log(p)* above 5.32) at their respective chromosome.

**Figure 7:**
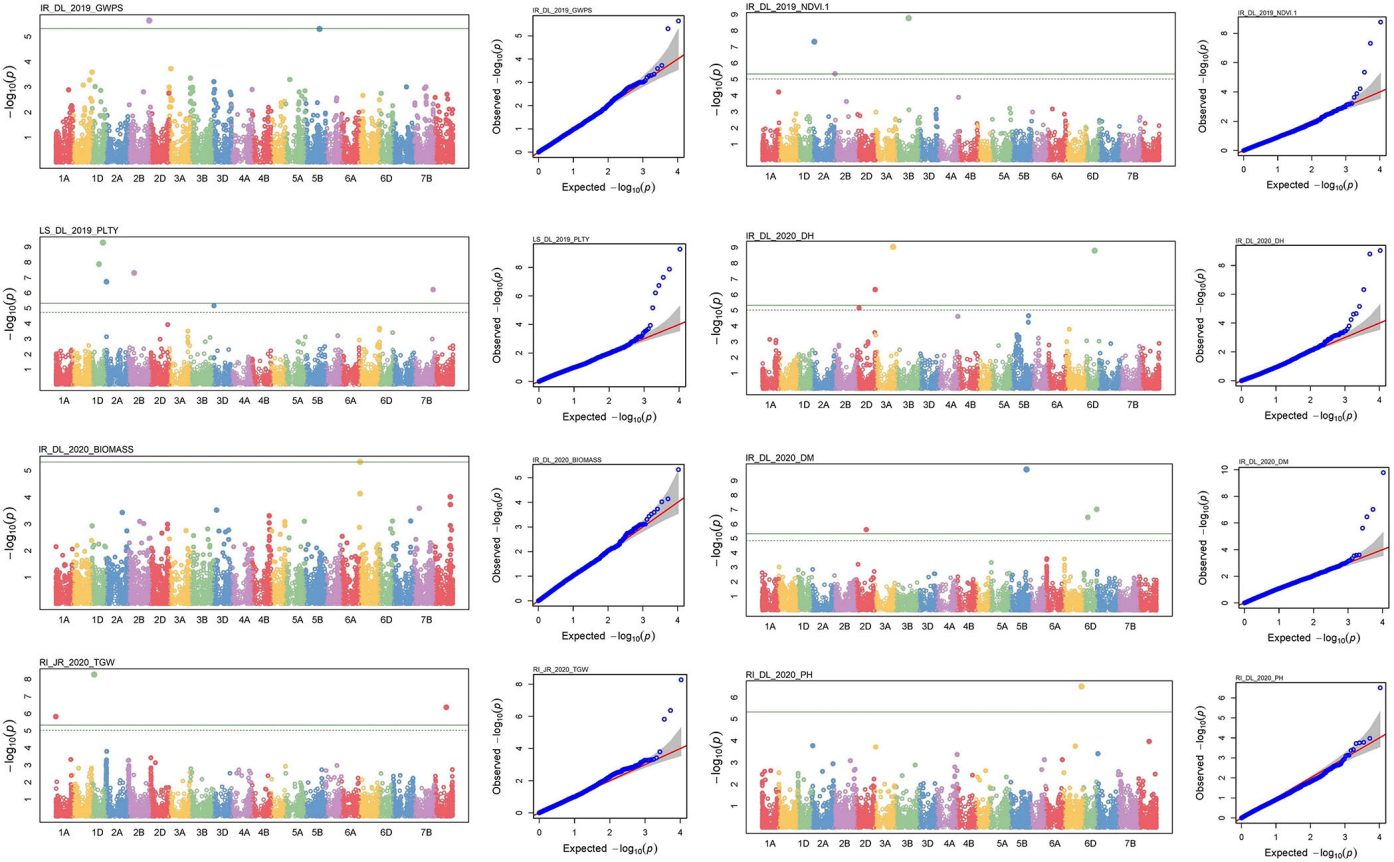
Manhattan and respective-QQ plots of significant associations at IR_Delhi_2019 for GWPS, LS_DL_2019 for PLTY, IR_DL_2020 for BIOMASS and RI_JR_2020 for TGW, IR_DL_2019 for NDVI, IR_DL_2020 for DH, IR_DL_2020 for DM and IR_DL_2020 for PH.

For all the studied traits together across the location with different treatment conditions, *viz*., IR, RI and LS, a total of 761 MTAs were identified with a significance −*log*_10_*(p)* value of >3. The highest number of SNPs were obtained for the trait NDVI (242 MTAs) and the lowest for the SPAD (19 MTAs). Associated SNPs identified for each trait under different conditions are listed with their respective *p*-values (Supplementary Table 4). MTAs were filtered with Bonferroni correction value (−*log*_10_*(p)* >5.32) to increase the stringency of selection, and 57 SNPs were obtained that were located on 18 different chromosomes (Figure 6). Out of 57 SNPs, 28 were identified under IR condition, which were linked with BIOMASS, DH, DM, GWPS NDVI, PH and TGW. Under RI condition, 16 SNPs linked with DH, GWPS, NDVI and PH were obtained. Thirteen significant associations were obtained under LS conditions for traits, *viz*., CT, DH, DM, GWPS, NDVI and PLTY (Table 3). Pictorial representation of some significant SNPs identified at Delhi and other locations for the studied traits were depicted with Manhattan plots along with QQ plots (Figure 7; Supplementary Figure 4). The details of MTAs above Bonferroni correction with their position in the genome are noted down (Table 4). Among them, 22 SNPs were stable and pleiotropic and were associated with six different traits, namely, DH, DM, NDVI, TGW, PH and BIOMASS. NDVI and DH were showing the highest number of stable MTAs, i.e., 7, followed by DM and TGW having 3 and 2 MTAs, respectively. Whereas BIOMASS and PH were having one stable association each (Table 5). Percent phenotypic variation explained (*r*^2^) by the stable MTAs ranged from 3.87% in PH to 19.22% in DM (Table 4).

**Table 4:** Significant marker trait associations at Bonferroni corrected *p value* for traits under study at each environment.

**Trait**	**Condition/location**	**SNP**	**Chromosome**	**Position in MB**	***P*-value**	**R square**	***-log_10_(P)***
BIOMASS	IR_DL_2020	AX-94466450	6B	29.84924	4.67E-06	0.071452	5.33089
CT	LS_DL_2019	AX-94573298	7D	629.4069	3.01E-06	0.20653	5.521182
DH	LS_DL_2020	AX-94416489	3A	46.7133	1.51E-06	0.081693	5.820014
	IR_PUNE_2020	AX-94435238	5D	474.2051	3.92E-09	0.098932	8.407157
	RI_PUNE_2020	AX-94435238	5D	474.2051	7.68E-10	0.097184	9.114737
	IR_IIWBR_2020	AX-94546495	2B	671.7411	1.26E-08	0.069093	7.900783
	IR_PUNE_2020	AX-94578563	3A	55.74342	1.40E-08	0.087051	7.852822
	RI_PUNE_2020	AX-94578563	3A	55.74342	1.78E-07	0.080392	6.749976
	IR_PUNE_2020	AX-94631711	3A	26.46988	3.07E-06	0.08455	5.513009
	IR_DL_2020	AX-94689491	3A	618.1646	9.18E-10	0.137955	9.037012
	IR_IIWBR_2020	AX-94789937	7B	33.53386	4.13E-07	0.076708	6.384131
	LS_DL_2020	AX-94940654	5D	367.436	4.17E-06	0.104355	5.379682
	IR_PUNE_2020	AX-94941121	3A	611.7026	3.28E-11	0.110475	10.48453
	RI_DL_2019	AX-94941121	3A	611.7026	2.34E-09	0.13456	8.630091
	RI_PUNE_2020	AX-94941121	3A	611.7026	2.77E-10	0.095057	9.557035
	RI_DL_2019	AX-95119024	1D	9.592365	1.21E-06	0.135519	5.917904
	RI_DL_2019	AX-95133267	2D	300.437	1.71E-06	0.138337	5.766205
	IR_DL_2020	AX-95170512	2D	634.3167	4.74E-07	0.129128	6.323975
	IR_DL_2020	AX-95195332	6D	304.1167	1.59E-09	0.119489	8.798271
	RI_JR_2020	AX-95235622	1D	314.5742	3.25E-10	0.111089	9.488192
DM	IR_DL_2020	AX-94463626	5B	580.8401	1.66E-10	0.189125	9.780337
	IR_DL_2020	AX-94490240	6D	462.537	9.50E-08	0.192265	7.022311
	IR_DL_2020	AX-94513007	6D	147.2393	3.43E-07	0.183054	6.465213
	IR_IIWBR_2020	AX-94725580	5B	594.8691	3.20E-08	0.085151	7.494575
	LS_DL_2020	AX-94725580	5B	594.8691	2.69E-08	0.09407	7.570521
	IR_DL_2020	AX-95186230	2D	354.7437	2.43E-06	0.15434	5.615057
GWPS	LS_DL_2019	AX-94505180	2A	733.0912	1.64E-07	0.108636	6.785046
	RI_DL_2019	AX-94590453	3A	8.325489	2.69E-08	0.141871	7.569559
	IR_DL_2019	AX-94664052	2B	748.1526	2.30E-06	0.081038	5.637959
	IR_DL_2019	AX-94988124	5B	489.2835	4.97E-06	0.077532	5.303543
	RI_DL_2019	AX-95150902	7B	200.9228	2.36E-07	0.133409	6.62789
NDVI.1	IR_DL_2019	AX-94425305	2A	62.27144	4.79E-08	0.089911	7.319923
	IR_DL_2019	AX-94659413	2B	14.04945	4.53E-06	0.10992	5.343983
	IR_DL_2019	AX-94720192	3B	417.493	1.69E-09	0.110884	8.771124
	IR_DL_2020	AX-94735072	2D	338.6773	3.35E-07	0.078254	6.474963
	LS_DL_2019	AX-94762983	1D	170.2247	1.57E-11	0.098748	10.80411
	LS_DL_2019	AX-95110974	1A	463.2904	2.06E-08	0.107363	7.685398
	LS_DL_2019	AX-95155574	5B	692.565	1.31E-06	0.116167	5.883924
NDVI.2	RI_DL_2019	AX-94436269	2B	95.79736	6.06E-10	0.115805	9.21725
	LS_DL_2020	AX-94552601	4B	666.5719	3.51E-08	0.093756	7.454121
	RI_DL_2020	AX-94560091	2B	661.4501	1.64E-08	0.078829	7.784441
	RI_DL_2020	AX-94658750	4A	27.67354	3.91E-07	0.059888	6.407471
NDVI.3	LS_DL_2019	AX-94433353	5A	460.5185	5.61E-10	0.110697	9.251103
	IR_DL_2020	AX-94463626	5B	580.8401	1.9E-08	0.121709	7.720863
	LS_DL_2019	AX-94935560	7A	63.38946	9.13E-09	0.132109	8.039646
PH	IR_JR_2020	AX-94415907	5B	421.6436	2.25E-07	0.038779	6.648635
	RI_DL_2020	AX-94599469	6B	644.4316	3.15E-07	0.116949	6.501722
PLTY	LS_DL_2019	AX-94390275	1D	246.6495	1.32E-08	0.187926	7.878685
	LS_DL_2019	AX-94833043	7B	682.8865	6.12E-07	0.200662	6.212957
	LS_DL_2019	AX-94916490	2B	214.2827	4.97E-08	0.20182	7.303398
	LS_DL_2019	AX-94997258	1D	385.8051	5.22E-10	0.21241	9.282206
	LS_DL_2019	AX-95257885	2A	16.25963	1.86E-07	0.203534	6.731365
TGW	RI_JR_2020	AX-94505686	1D	80.44586	5.32E-09	0.124761	8.273984
	RI_JR_2020	AX-94598030	1A	1.159536	1.51E-06	0.117123	5.822309
	IR_PUNE_2020	AX-94634468	2B	767.3743	1.52E-07	0.073127	6.817671
	IR_IIWBR_2020	AX-94700391	3A	700.422	6.70E-08	0.084706	7.173684
	RI_JR_2020	AX-95107567	7D	406.8963	4.32E-07	0.12292	6.364986

**Table 5:** List of stable SNPs expressed at more than one environment and Pleiotropic^*^ SNPs (Bold ones) linked to more than one traits.

**SNP**	**Chromosome**	**Position**	**Trait**	**Location**	***-log10(p)* Value**
**AX-94466450**	6B	3E+07	BIOMASS, PLTY	DL.IR / DL.IR	5.33 / 4.46
AX-94631711	3A	2.6E+07	DH	PUNE IR / PUNE RI	5.51 / 3.86
AX-94578563	3A	5.6E+07	DH	PUNE IR / PUNE RI	7.85 / 6.75
AX-94637995	2D	6.2E+07	DH	DL.IR / DL.2019.LS	5.16 / 3.21
AX-95133267	2D	3E+08	DH	DL.2019.RI / DL.2019.RI / DL.2019.LS	5.77 / 5.23 / 3.42
AX-95235622	1D	3.1E+08	DH	JR.RI / DL.RI / DL.2019LS / DL.LS	9.49 / 3.99 / 3.4 / 3.24
AX-94435238	5D	4.7E+08	DH	PUNE RI / PUNE IR	9.11 / 8.41
AX-94941121	3A	6.1E+08	DH	PUNE IR / PUNE RI / DL.2019.RI	10.48 / 9.56 / 8.63
**AX-94725580**	5B	5.9E+08	DM, DM, DH	DL.LS / IIWBR / DL.LS	7.57 / 7.49 / 4.72
**AX-94490240**	6D	4.6E+08	DM, NDVI, DH	DL.IR / DL.IR / DL.IR	7.02 / 3.3 / 3.04
**AX-94463626**	5B	5.8E+08	DM, NDVI, DH, NDVI, NDVI	DL.IR / DL.IR / DL.IR / DL.IR / DL. LS	9.78 / 7.72 / 4.66 / 3.11 / 3
**AX-94513007**	6D	1.5E+08	DM, TGW	DL.IR / IIWBR	6.47 / 3.21
**AX-94988124**	5B	4.9E+08	GWPS, DH	DL.2019.IR / DL.2019.LS	5.3 / 4.07
AX-94552601	4B	6.7E+08	NDVI	DL.LS / DL.RI	7.45 / 3.03
AX-94436269	2B	9.6E+07	NDVI	DL.2019.RI / DL.IR	9.22 / 3.04
**AX-94560091**	2B	6.6E+08	NDVI, DH	DL.RI / DL.2019.LS	7.78 / 3.64
AX-95155574	5B	6.9E+08	NDVI	DL.2019.LS	5.88 / 3.14
AX-94433353	5A	4.6E+08	NDVI	DL.2019.LS / DL.2020.LS / DL.2019.LS.NDVI.3	9.25 / 4.2 / 3.73
AX-94762983	1D	1.7E+08	NDVI	DL.2019.LS / DL.2019.LS	10.8 / 3.01
AX-94415907	5B	4.2E+08	PH	JR.RI / IIWBR	6.65 / 3.41
**AX-94598030**	1A	1159536	TGW, NDVI	JR.RI / DL.IR	5.82 / 3.36
**AX-94759710**	3D	2.3E+07	TGW, NDVI	PUNE RI	5.3 / 3.16

*Bold one are pleiotropic MTAs.

We identified nine pleiotropic MTAs having associations with different traits (Table 5). The SNP marker AX-94490240 and AX-94463626 were associated with DM, NDVI and DH, whereas AX-94598030 and AX-94759710 were linked with TGW and NDVI. Similarly, GWPS and DH were associated with AX-94988124 and AX-94466450 with BIOMASS and PLTY. However, their strength of association differs depending on traits and location (Table 5).

### *In silico* analysis

BLAST analysis of stable SNPs against the IWGSC reference genome of *Triticum aestivum* revealed the location of SNPs in the gene-rich region of the genome. Almost all SNPs were near to one or the other transcript coding for some proteins or transcription factors except SNP AX-94466450. SNPs were located near the genes coding for proteins like peroxisomal membrane protein, ran binding protein, augmin family, etc. (Table 6). SNPs AX-94762983 and AX-95133267 were located in a protein-coding region whose protein is still unknown. The SNPs markers like AX-94578563, AX-94941121 and AX-94631711 were linked to the genes governing the traits like endosperm, ascorbate content and root growth, and are helpful in the heading of the wheat. Similarly, SNPs like AX-94759710 and AX-94598030 linked are to TGW and are present near the gene coding region for endosperm development and resistance to oxidative stress (Table 6).

**Table 6:** Putative candidate genes identified at the 10 kb region of Linked SNPs along with their molecular functions.

**Trait**	**SNP**	**Gene**	**Position**	**Protein**	**Role**	**Reference**
DH	AX-94637995	TraesCS2D02G112700.1	2D: 62,401,570-62,405,841	AIG1-type guanine nucleotide-binding (G) domain		
	AX-94631711	TraesCS3A02G050211.2	3A: 26,472,148-26,482,725	Wall-associated receptor kinase, galacturonan-binding domain	Regulation of root growth	Kaur et al., 2013
	AX-95133267	TraesCS2D02G328100LC.1	2D: 300,439,172-300,440,223	Protein coding		
	AX-94578563	TraesCS3A02G086500.1	3A: 55,743,110-55,743,857	Invertase/pectin methylesterase inhibitor domain superfamily	Early development of wheat grain endosperm and outer layers	Mehdi et al., 2020
	AX-94941121	TraesCS3A02G363000.1	3A: 611,700,692-611,703,172	SUGAR-1-PHOSPHATE GUANYL TRANSFERASE	L-galactose guanyltransferase, increases leaf ascorbate content	Laing et al., 2007
	AX-94435238	TraesCS5D02G410900.1	5D: 474,200,732-474,205,422	AUGMIN FAMILY	Centrosome cycle spindle assembly	Hotta et al., 2012
	AX-95235622	TraesCS1D02G226200.1	1D: 314,573,418-314,574,779	RAN BINDING PROTEIN		
DM	AX-94513007	TraesCS6D02G166200.1	6D: 147,236,496-147,241,863	RNA recognition motif domain		
	AX-94490240	TraesCS6D02G383800.1	6D: 462,536,412-462,540,932	ZINC FINGER, RING/FYVE/PHD-TYPE		
	AX-94725580	TraesCS5B02G418600.1	5B: 594,867,010-594,872,193	Inosine-5'-monophosphate dehydrogenase	Regulation of cell growth.	Uniprot
NDVI	AX-95155574	TraesCS5B02G536500.1	5B: 692,559,588-692,565,223	Serine/threonine-protein kinase, active site	Flag leaf width, plant height and water-soluble carbohydrates under drought conditions	Zhang et al., 2013
	AX-94552601	TraesCS4B02G390400.1	4B: 666,571,620-666,572,590	Ubiquitin-like domain		
	AX-94463626	TraesCS5B02G405200.1	5B: 580,830,088-580,840,255	ALPHA-N-ACETYLGLUCOSAMINIDASE	Fertilization and seed development in Arabidopsis	Ronceret et al., 2008
	AX-94560091	TraesCS2A02G444900.1	2A: 694,905,983-694,909,467	Peptidase S8 and S53	Integrin-mediated signaling pathway, calcium ion binding	Uniport
	AX-94436269	TraesCS2B02G127800.1.	2B: 95,794,615-95,797,096	AP2/ERF DOMAIN-CONTAINING PROTEIN	Ethylene-responsive transcription factor	Djemal and Khoudi, 2015
	AX-94433353	TraesCS5A02G246700.1	5A: 460,516,405-460,520,083	Protein kinase, ATP binding site		
	AX-94762983	TraesCS1D02G197500LC.1	1D: 170,223,005-170,223,304	Protein coding		
PH	AX-94415907	TraesCS5B02G241800.1	5B: 421,643,604-421,643,664	ACTIN T1-LIKE PROTEIN		
TGW	AX-94759710	TraesCS3D02G055400.1	3D: 23,057,692-23,061,395	Glycosyl transferase, family 1	Development of Rice Endosperm	Yang et al., 2021
	AX-94598030	TraesCS1A02G001900.6	1A: 1,162,817-1,166,405	PEROXISOMAL MEMBRANE PROTEIN	Plant proteases, protein degradation, and oxidative stress	Palma et al., 2002

## Discussion

Drought and heat have the greatest influence on wheat varieties, therefore identifying the genomic region using genome-wide SNP markers is a smart way to gain knowledge, which can then be used to create climate-resilient varieties. The goal of this study is to identify a new region of the wheat genome that is responsible for drought and heat stress resistance. The use of elite breeding material for GWAS invariably reduces the number of significant SNPs compared with other studies, where diverse plant materials with high diversity and larger phenotypic differences were used (Bordes et al., 2014; Zanke et al., 2015). In such material QTL with large effects on the traits may be fixed in the breeding lines, and will therefore not be detectable (Kristensen et al., 2018). However, the utilization of an advanced breeding line will help to explore untouched parts of the genome having minor effects on the target traits by avoiding the influence of major QTL regions during the study that are already fixed. Furthermore, advanced breeding lines are ready to use the material in the breeding programme as a parent (Kristensen et al., 2018) with preferred qualities and having a meager problem of linkage drag.

In this study, the near normal distribution among all the studied traits (Figure 1) indicated the polygenic nature of the studied traits. Significant variation was observed from the analysis of variance indicating the data can be used for further analysis. The coefficient of variation was low for the traits, such as DH, DM and NDVI, whereas high CV was observed for biomass and plot yield which was due to the high influence of environmental factors. To nullify the effect of environmental influence, multi-location and multi-year data were used to find out stable associations.

In GWAS analysis, population structure might be a confounding factor that must be addressed to avoid false associations. STRUCTURE and PCA are two popular approaches for inferring the population structure of the genome-wide association panel using high-density SNPs (Abraham and Inouye, 2014). The use of genotypes from Indian and exotic introduction in the study might be the reason for clear-cut two subpopulations (Figure 4A) in the mapping panel. As many as 14 admixture genotypes carrying genomic regions from both the subpopulations were observed, which is due to the advanced breeding line developed from common founding parents used in their crossing plan. AM panels with subpopulations were used efficiently by using either PCA-based grouping (Odilbekov et al., 2019; Rathan et al., 2022) or with the uses of the Q matrix from the STRUCTURE analysis as covariates (Beyer et al., 2019; Luján Basile et al., 2019; Danakumara et al., 2021; Alotaibi et al., 2022). Apart from that, suitable diversity for GWAS study among the genotypes in AM panel was confirmed by neighbor-joining clustering from the distance matrix. The use of a diverse panel of genotypes can provide more valuable inference compared to bi-parental populations (Vos-Fels et al., 2017) by taking advantage of maximum allelic diversity (Ayalew et al., 2018; Onyemaobi et al., 2018). The present study material being developed from multiple crossing ensures the required diversity for the association study, and estimation is based on neighbor-joining clusters and kinship-based heat map (Figure 4D).

In outcrossing crop species like maize, LD block was observed at a short distance and thus decays were faster, and in the case of self-pollinated crops longer distance is attributed to a lower decay rate as in wheat (Yu et al., 2014; Roncallo et al., 2021). For example, the genome-wide LD decay distance is ~100 kb in rice and ~2 kb in maize (Huang and Han, 2014); however, in wheat, up to 30.4 mb LD decay was observed in Argentinian germplasm collection (Roncallo et al., 2021). LD is important in population genetics and crop improvement using molecular techniques (Gupta et al., 2005). The number of markers required for association mapping is determined by the extent of LD decay, based on the genetic distance between markers (Mather et al., 2007). LD decay varies widely among wheat populations. In this study, LD at half LD decay (Figure 5) varies for each genome with larger whole genome LD of 7.15 Mb, inferring the lower decay for the advanced breeding materials. Similarly, a large LD block size of 4.4 MB was observed by Pang et al. (2020). As much as 9.22 Mb distance was observed for the D genome inferring lower decay, which is in accordance with the previous study (Ogbonnaya et al., 2017; Jamil et al., 2019; Li G. et al., 2019; Pang et al., 2020). In contrast, faster LD decay in the D genome was observed; comparable to the A and B genomes in a study using breeding lines developed from synthetics, driving more recombination in the D genome (Ledesma-Ramírez et al., 2019). The significant variations in LD decay rates among the A, B and D genomes imply that the three genomes and their donors, *T. turgidum* (A and B) and *Ae. tauschii* (D), might have evolved independently and under different selection pressures throughout the domestication and modern breeding objectives (Mirzaghaderi and Mason, 2017). LD decay depends on cultivation patterns, breeding methods, breeding history and evolutionary history. Wheat is grown once a year, hence has a much slower rate of evolution, and likely accumulated far fewer historical recombination events and mutations, resulting in a slower LD decay than in other self-pollinated crops like rice (Pang et al., 2020).

At a significant *p* < 0.001, a total of 761 SNPs were identified to be associated with traits under investigation. Such a huge number of MTAs can be justifiable for high-throughput genotyping data and with the cut-off *p-value* of 0.001, similar to earlier reports for various agronomic traits (Ma et al., 2018; Pang et al., 2020). A huge number of SNPs obtained for yield-related traits under stress conditions may include many of the false positives due to lower threshold values. To avoid such biasness, a stringent selection procedure of Bonferroni correction was applied like Kumar et al. (2018), and in total 57 MTAs were retained. If geographical and environmental variation among different locations was significantly high, a location-wise association study was carried out to avoid flattening of the genetic variation as mentioned in previous references (Pujar et al., 2020; Rathan et al., 2022). MTAs observed in more than one location with Bonferroni corrected *p-value* in at least one location (Stable MTAs) are presumed to be the true association, which is supported by the presence of the candidate gene (Table 6).

NDVI is an indicator of vegetation response to drought based on the relationships between NDVI and drought index (Rutkoski et al., 2016; Singh et al., 2016). In our study, as many as 242 SNPs were linked with NDVI at an LOD score of 3. When we observe the stable expressing SNPs across the environment, 10 SNPs belonging to NDVI indicated a true association with it. The reason might be drought and heat conditions applied by restricted irrigation and late sown conditions have induced the drought and heat tolerant genotypes to exhibit related traits and associated SNPs were identified. Susceptibility indices of drought and heat could be a potential targeting trait to encounter consistent yield in the stress breeding programme (Devi et al., 2022; Mutari et al., 2022). An extensive analysis of susceptibility indices could be our further target to dissect the genomic regions concerned with drought and heat tolerant traits.

A total of 22 stable SNPs were found for different traits having major number of MTAs for NDVI (10) and DH (11). Easy phenotyping and having high accuracy might lead to a greater number of associations between DH and NDVI. DH-linked SNPs were detected on chromosomes 3A, 2D, 1D, 5D, 3A, 5B, and 6D are on par with earlier reports like the presence of VRN genes responsible for flowering on chromosomes 5A, 5B and 5D (Ogbonnaya et al., 2017). Markers linked with the DH were also found on chromosomes 1D, 2A, 4A, 5B, 5D, 6A, 6B, and 7A (Jamil et al., 2019). It is notable that we got clusters of markers on chromosome 5B for DM, GWPS, NDVI and PH that might be influenced by the presence of the *vrn B-1* gene on 5B. Earlier reports denote 29.1% of phenotypic variations for heading date from this region (Rivera-Burgos et al., 2022). Previous studies confirmed the influence of the chromosome 5B on flowering and the presence of *VRN* genes at 5B, influencing the vegetative and reproductive traits (Kiseleva et al., 2016; Huang et al., 2018). For NDVI, MTAs were obtained on chromosomes 1A and 5A on the A genome, 2B, 4B, and 5B on the B genome and 1D, 3D, and 6D on the D genome. The MLM-Q+K-based analysis detected unique NDVI QTLs on chromosomes 1A, 1B, 2B, 4A, 4B, 5A, 6A, 6B, and 7A in a study conducted by Condorelli et al. (2018). Similarly, NDVI-related MTAs on 1A and 7A were found out by Ward et al. (2019). As argued by Paliwal et al. (2012), a chromosomal region on 2B is of prime importance for heat stress. We obtained a stable SNP on the 2B chromosome for NDVI with an 11.5% variation explained. Apart from these, MTAs, *viz.*, AX-94466450 (6B), AX-94988124 (5B) and AX-94415907 (5B) were linked stably to the traits, plot yield, GWPS and PH, respectively. Three SNPs, AX-94513007, AX-94598030 and AX-94759710, were identified for TGW on chromosomes 1A, 3D and 6D. In previous studies, markers linked with TGW were observed in 6D (Wang et al., 2012; Chen et al., 2016), 1A (Ogbonnaya et al., 2017) and pleiotropic regions affecting kernel weight-related traits on chromosomes 1B, 2A and 3A (Chen et al., 2016). Contrary to this, stable SNPs for TGW were observed on chromosomes 5A (Wang et al., 2017), 1D and 7A (Edae et al., 2014). SNPs location on 1A, 3D and 6D were novel in our study.

Stable expression along the different locations and conditions is presumed to be the real association of these markers with the studied traits. Significant SNPs detected in this study for grain yield parameter can be indirectly selected under drought and heat condition having an influence on the stress tolerance mechanism and pathway involved in abiotic stress tolerance, which is also observed by Schmidt et al. (2020). Hence, it is common to find markers to be associated with more than one trait, i.e. pleiotropic influence. Significant pleiotropic loci were detected for yield and stress tolerance-related traits showing yield and stress tolerant traits have an influence on one another as reported by Mathew et al. (2018). Similarly, we found nine different stable MTAs showing pleiotropic effects between different yield related and stress-tolerant traits as depicted in Table 5. Important yield trait like TGW linked SNPs, *viz*., AX-94598030 and AX-94759710 were pleiotropic with NDVI, an important drought tolerant trait. Pleiotropy between yield related and NDVI was found in a QTL mapping study by Shi et al. (2017). SNP, AX-94513007, was having pleiotropy between TGW and DM, a stay-green trait was helpful in heat and drought tolerance. It is clear that markers, *vi*z., AX-94725580, AX-94490240, AX-94560091 and AX-94463626 exhibit pleiotropy among traits DH, DM and NDVI. Due to the interdependence on one another they are bound to share genes in common and the results can also be supported by the presence of positive correlation among these traits (Figure 2). Pleiotropy between NDVI and TGW observed by the markers AX-94598030 and AX-94759710 indicates the collinearity between stress tolerant and yield-related traits under stress condition. Markers such as AX-94560091 located near to the transcript TraesCS1A02G001900 related to Integrin-mediated signaling pathway and calcium ion-binding protein obviously having multiple roles in drought tolerance in plant system (Lü et al., 2007; Takahashi et al., 2020). Pleiotropic maker AX-94598030 was mapped near the proteins involved in stress tolerance, such as peroxisomal membrane protein (Table 6), which are involved in the mitigation of protein degradation and oxidative stress tolerance (Palma et al., 2002), in turn may have influence on yield parameters in stress. The pleiotropic effect between stress tolerance and grain traits are previously reported in many studies (Ahmed et al., 2022b). Markers such as AX-94578563 and AX-94941121 associated with DH were present near the gene coding for Invertase/pectin methylesterase inhibitor domain superfamily and sugar-1-phosphate guanyl transferase, respectively. The first has a role in early development of wheat grain endosperm and outer layers (Mehdi et al., 2020), which is related to flowering fertilization in flowering plants. Whereas the second one has a role in L-galactose guanyl-transferase, which increases leaf ascorbate content that induces early flowering (Laing et al., 2007). These findings found out that novel MTAs that are detected here can be evaluated further for the validation of the markers.

Furthermore, such markers can be utilized for marker assisted breeding for genes related to drought and heat tolerance along with high yield. MTAs that are stable across the environment have great potential to be deployed in developing new wheat varieties through molecular breeding. Marker validation and pathway followed by the genes associated with markers can be analyzed for further evidence to support the reliability of associations, thereby have utilization in breeding programmes. As the plant materials used in the study are advanced breeding lines that are used for further evaluation to release variety or can be directly used as parents in breeding programmes.

## Conclusion

Genetic dissection of the genomic region responsible for drought and heat tolerance is having immense importance in the development of climate-resilient varieties. A total of 295 advanced breeding lines used in GWAS panel showed continuous variation for most of the studied traits. Sufficient genetic diversity was observed in AM panel with structured two subpopulations. A large LD block size of 7.15 MB was found out showing reliable linkage of markers with the trait of interest for more generations. Fifty-seven high-confident markers associated with drought and heat tolerance and yield related traits, *viz.*, DH, DM, NDVI, PH and TGW were discovered in this study. Many of the identified MTAs were located near the putative candidate gene and protein coding transcript influencing the traits of interest. A total of 22 stable MTAs identified across the locations were having practical utilization in future wheat breeding programmes.

## Data availability statement

The original contributions presented in the study are included in the article/Supplementary material, and in the DRYAD repository, accessible at https://datadryad.org/stash/share/SwCnD0OA5Pi0oa96xplZAv3k51QMn0FFU_0kEZNFpn0 further inquiries can be directed to the corresponding author/s.

## Author contributions

PS, NJ, GS, and HKr conceptualized the investigation and edited the manuscript. PS supervised the conduct of the experiment. ND conducted the investigation and prepared the draft of the manuscript. ND, SP, MK, HKh, RP, and JS generated the phenotypic data. HKr, SS, DC, and KM contributed in the generation of genotyping data. ND and HKr did the statistical and GWAS analysis. PS, HKr, MK, HKh, RP, and JS conducted field trials and provided help in recording observations. All authors contributed to the article and approved the submitted version.

## Funding

This study was supported by funding provided by the Indian Council of Agricultural Research (ICAR) and the Bill & Melinda Gate Foundation (BMGF) under the project ICAR-BMGF (Grant number: OPP1194767).

## Conflict of interest

The authors declare that the research was conducted in the absence of any commercial or financial relationships that could be construed as a potential conflict of interest.

## Publisher's note

All claims expressed in this article are solely those of the authors and do not necessarily represent those of their affiliated organizations, or those of the publisher, the editors and the reviewers. Any product that may be evaluated in this article, or claim that may be made by its manufacturer, is not guaranteed or endorsed by the publisher.
