# Supplementary material for: Genome-wide association mapping for component traits of drought and heat tolerance in wheat
Source: Front Plant Sci. 2022 Aug 16;13:943033. doi: 10.3389/fpls.2022.943033 (PMC9429996; doi:10.3389/fpls.2022.943033)
Supplement: Supplementary file 1 [file Data_Sheet_1.ZIP › Supp,Table 1.docx]

**Supplementary table 1. Details of lines in GWAS panel, pedigree with origin**

*ADT – Advanced Trials, nomenclature to identify lines.

| **Genotype** | **Pedigree** | **Origin** |
| --- | --- | --- |
| ADT-1 | HD 3076/HD 2781 | IARI New Delhi |
| ADT-2 | HD 3070/HI 1500 | IARI New Delhi |
| ADT-3 | HD 2967/HD 3043 | IARI New Delhi |
| ADT-4 | HD 2733/HD 3016 | IARI New Delhi |
| ADT-5 | HD 3070/HD 2781 | IARI New Delhi |
| ADT-6 | RAC 875/HD 2733 | IARI New Delhi |
| ADT-7 | HD 3078/HD 2781 | IARI New Delhi |
| ADT-8 | HD2932/CHIRIYA3 | IARI New Delhi |
| ADT-9 | HD 3076/HD 2781 | IARI New Delhi |
| ADT-10 | HD 3076/HD 3078 | IARI New Delhi |
| ADT-11 | HD 3078/HI 1500 | IARI New Delhi |
| ADT-12 | HD 3043/HD 3086 | IARI New Delhi |
| ADT-13 | BERKUT/PBW 550 | IARI New Delhi |
| ADT-14 | HD 3087/HD 3016 | IARI New Delhi |
| ADT-15 | HD 3076/HD 3086 | IARI New Delhi |
| ADT-16 | HD 3087/GW 322 | IARI New Delhi |
| ADT-17 | HD 2967/HD 3043 | IARI New Delhi |
| ADT-18 | 34SAWSN_3007 | CIMMYT Mexico |
| ADT-19 | 34SAWSN_3015 | CIMMYT Mexico |
| ADT-20 | HD 3076/HD 2781 | IARI New Delhi |
| ADT-21 | HD 2733/HD 3043 | IARI New Delhi |
| ADT-22 | HD 2733/HD 3076 | IARI New Delhi |
| ADT-23 | HD 2967/HD 3043 | IARI New Delhi |
| ADT-24 | 34SAWSN_3041 | CIMMYT Mexico |
| ADT-25 | HD 3076/HD 2781 | IARI New Delhi |
| ADT-26 | HD 3070/HD 2781 | IARI New Delhi |
| ADT-27 | 34SAWSN_3245 | CIMMYT Mexico |
| ADT-28 | HD 2733/HD 3043 | IARI New Delhi |
| ADT-29 | HD 3070/HD 3078 | IARI New Delhi |
| ADT-30 | HD 2733/HD 3043 | IARI New Delhi |
| ADT-31 | RAC 875/GW 322 | IARI New Delhi |
| ADT-32 | BERKUT/GW 322 | IARI New Delhi |
| ADT-33 | GW 322/DBW 14//HD 2733 | IARI New Delhi |
| ADT-34 | GW 366/3/SOKOLL//FRTL/2*PIFED | IARI New Delhi |
| ADT-35 | PBW 550/6/CNO79//PF70354/MUS/ 3/PASTOR/4/ BAV92/5/FRET2/KUKUNA//FRET2/6/MILAN/KAUZ//PRINIA/3/BAV92 | IARI New Delhi |
| ADT-36 | PBW 550/7/CNO79//PF70354/MUS/ 3/PASTOR/4/ BAV92/5/FRET2/KUKUNA//FRET2/6/MILAN/KAUZ//PRINIA/3/BAV92 | IARI New Delhi |
| ADT-37 | PBW 550/7/CNO79//PF70354/MUS/ 3/PASTOR/4/ BAV92/5/FRET2/KUKUNA//FRET2/6/MILAN/KAUZ//PRINIA/3/BAV92 | IARI New Delhi |
| ADT-38 | HD 2733/BACANORA | IARI New Delhi |
| ADT-39 | HD 2733/BACANORA | IARI New Delhi |
| ADT-40 | HD 2733/BACANORA | IARI New Delhi |
| ADT-41 | HD 2733/BACANORA | IARI New Delhi |
| ADT-42 | PAIRED TIN LINE/HD 3059 | IARI New Delhi |
| ADT-43 | PAIRED TIN LINE/HD 3059 | IARI New Delhi |
| ADT-44 | PAIRED TIN LINE/HD 3059 | IARI New Delhi |
| ADT-45 | HD 2733/HD 3043 | IARI New Delhi |
| ADT-46 | HD 2733/HD 3043 | IARI New Delhi |
| ADT-47 | HD 2733/HD 3043 | IARI New Delhi |
| ADT-48 | HD 2733/HD 3043 | IARI New Delhi |
| ADT-49 | HD 2733/HD 3043 | IARI New Delhi |
| ADT-50 | HD 2733/HD 3076 | IARI New Delhi |
| ADT-51 | HD 2733/HD 3076 | IARI New Delhi |
| ADT-52 | HD 2733/HD 3076 | IARI New Delhi |
| ADT-53 | HD 2733/HD 3076 | IARI New Delhi |
| ADT-54 | HD 2733/HD 3076 | IARI New Delhi |
| ADT-55 | HD 2733/HD 3078 | IARI New Delhi |
| ADT-56 | HD 2733/HD 3078 | IARI New Delhi |
| ADT-57 | HD 2733/HD 3078 | IARI New Delhi |
| ADT-58 | HD 2733/HD 2781 | IARI New Delhi |
| ADT-59 | HD 3043/HD 3076 | IARI New Delhi |
| ADT-60 | HD 3043/HD 3076 | IARI New Delhi |
| ADT-61 | HD 3043/HD 3076 | IARI New Delhi |
| ADT-62 | HD 3043/HD 3076 | IARI New Delhi |
| ADT-63 | HD 3043/HD 3076 | IARI New Delhi |
| ADT-64 | HD 3043/HD 3078 | IARI New Delhi |
| ADT-65 | HD 3043/HD 3078 | IARI New Delhi |
| ADT-66 | HD 3043/HD 3086 | IARI New Delhi |
| ADT-67 | HD 3043/HD 2781 | IARI New Delhi |
| ADT-68 | HD 3043/HD 2781 | IARI New Delhi |
| ADT-69 | HD 3043/HD 2781 | IARI New Delhi |
| ADT-70 | HD 3070/HD 3078 | IARI New Delhi |
| ADT-71 | HD 3070/HD 3078 | IARI New Delhi |
| ADT-72 | HD 3070/HD 3078 | IARI New Delhi |
| ADT-73 | HD 3070/HD 2781 | IARI New Delhi |
| ADT-74 | HD 3070/HD 2781 | IARI New Delhi |
| ADT-75 | HD 3070/HD 2781 | IARI New Delhi |
| ADT-76 | HD 3070/HD 2781 | IARI New Delhi |
| ADT-77 | HD 3070/HD 2781 | IARI New Delhi |
| ADT-78 | HD 3076/HD 3078 | IARI New Delhi |
| ADT-79 | HD 3076/HD 3078 | IARI New Delhi |
| ADT-80 | HD 3078/HD 3086 | IARI New Delhi |
| ADT-81 | HD 3078/HD 3086 | IARI New Delhi |
| ADT-82 | HD 3078/HD 3086 | IARI New Delhi |
| ADT-83 | HD 3078/HD 3086 | IARI New Delhi |
| ADT-84 | HD 3078/HD 2781 | IARI New Delhi |
| ADT-85 | HD 3078/HD 2781 | IARI New Delhi |
| ADT-86 | HD 3078/HD 2781 | IARI New Delhi |
| ADT-87 | HD 3078/HD 2781 | IARI New Delhi |
| ADT-88 | HD 3078/HI 1500 | IARI New Delhi |
| ADT-89 | HD 3086/HD 2781 | IARI New Delhi |
| ADT-90 | HD 3086/HD 2781 | IARI New Delhi |
| ADT-91 | HD 3086/HD 2781 | IARI New Delhi |
| ADT-92 | HD 3086/HD 2781 | IARI New Delhi |
| ADT-93 | HD 3086/HD 2781 | IARI New Delhi |
| ADT-94 | HD 3086/HD 2781 | IARI New Delhi |
| ADT-95 | HD 3086/HD 2781 | IARI New Delhi |
| ADT-96 | HD 3086/HD 2781 | IARI New Delhi |
| ADT-97 | HD 3086/HI 1500 | IARI New Delhi |
| ADT-98 | HD 3086/HI 1500 | IARI New Delhi |
| ADT-99 | HD 3086/HI 1500 | IARI New Delhi |
| ADT-100 | HD 3086/HI 1500 | IARI New Delhi |
| ADT-101 | HD 3086/HI 1500 | IARI New Delhi |
| ADT-102 | HD 3086/HI 1500 | IARI New Delhi |
| ADT-103 | HD 3086/HI 1500 | IARI New Delhi |
| ADT-104 | HD 3086/HI 1500 | IARI New Delhi |
| ADT-105 | HD 3086/HI 1500 | IARI New Delhi |
| ADT-106 | HD 3086/HI 1500 | IARI New Delhi |
| ADT-107 | HD 3086/HI 1500 | IARI New Delhi |
| ADT-108 | CIMCOG-26/HD2932 | IARI New Delhi |
| ADT-109 | CIMCOG-26/HD2932 | IARI New Delhi |
| ADT-110 | CIMCOG-26/HD2932 | IARI New Delhi |
| ADT-111 | CIMCOG-26/HD2932 | IARI New Delhi |
| ADT-112 | CIMCOG-26/PBW550 | IARI New Delhi |
| ADT-113 | CIMCOG-31/HUW468 | IARI New Delhi |
| ADT-114 | CIMCOG-31/HUW468 | IARI New Delhi |
| ADT-115 | CIMCOG-31/HUW468 | IARI New Delhi |
| ADT-116 | CIMCOG-31/HUW468 | IARI New Delhi |
| ADT-117 | CIMCOG-31/HUW510 | IARI New Delhi |
| ADT-118 | CIMCOG-31/HUW510 | IARI New Delhi |
| ADT-119 | CIMCOG-31/PBW550 | IARI New Delhi |
| ADT-120 | CIMCOG-35/HD2733 | IARI New Delhi |
| ADT-121 | CIMCOG-35/HD2733 | IARI New Delhi |
| ADT-122 | CIMCOG-35/HUW468 | IARI New Delhi |
| ADT-123 | CIMCOG-35/HUW468 | IARI New Delhi |
| ADT-124 | CIMCOG-35/HUW468 | IARI New Delhi |
| ADT-125 | CIMCOG-35/HUW468 | IARI New Delhi |
| ADT-126 | CIMCOG-35/HUW468 | IARI New Delhi |
| ADT-127 | CIMCOG-35/HUW510 | IARI New Delhi |
| ADT-128 | CIMCOG-35/PBW550 | IARI New Delhi |
| ADT-129 | CIMCOG-35/PBW550 | IARI New Delhi |
| ADT-130 | CIMCOG-37/FLW-9 | IARI New Delhi |
| ADT-131 | CIMCOG-37/FLW-9 | IARI New Delhi |
| ADT-132 | CIMCOG-44/RAJ4229 | IARI New Delhi |
| ADT-133 | CIMCOG-44/RAJ4229 | IARI New Delhi |
| ADT-134 | CIMCOG-50/WH1105 | IARI New Delhi |
| ADT-135 | CIMCOG-50/WH1105 | IARI New Delhi |
| ADT-136 | CIMCOG-55/PBW550 | IARI New Delhi |
| ADT-137 | CIMCOG-55/PBW550 | IARI New Delhi |
| ADT-138 | MP 4010 /Lok-1//Synthetic_46 / HD 2967 | IARI New Delhi |
| ADT-139 | IBWSN_1020 | CIMMYT Mexico |
| ADT-140 | IBWSN_1081 | CIMMYT Mexico |
| ADT-141 | IBWSN_1085 | CIMMYT Mexico |
| ADT-142 | IBWSN_1088 | CIMMYT Mexico |
| ADT-143 | IBWSN_1110 | CIMMYT Mexico |
| ADT-144 | IBWSN_1143 | CIMMYT Mexico |
| ADT-145 | IBWSN_1150 | CIMMYT Mexico |
| ADT-146 | IBWSN_1158 | CIMMYT Mexico |
| ADT-147 | IBWSN_1191 | CIMMYT Mexico |
| ADT-148 | IBWSN_1220 | CIMMYT Mexico |
| ADT-149 | IBWSN_1272 | CIMMYT Mexico |
| ADT-150 | IBWSN_1275 | CIMMYT Mexico |
| ADT-151 | 35 SAWYT_3001 | CIMMYT Mexico |
| ADT-152 | 35 SAWYT_3003 | CIMMYT Mexico |
| ADT-153 | 35 SAWYT_3011 | CIMMYT Mexico |
| ADT-154 | 35 SAWYT_3029 | CIMMYT Mexico |
| ADT-155 | 35 SAWYT_3045 | CIMMYT Mexico |
| ADT-156 | 35 SAWYT_3048 | CIMMYT Mexico |
| ADT-157 | 35 SAWYT_3076 | CIMMYT Mexico |
| ADT-158 | 35 SAWYT_3078 | CIMMYT Mexico |
| ADT-159 | 35 SAWYT_3092 | CIMMYT Mexico |
| ADT-160 | 35 SAWYT_3103 | CIMMYT Mexico |
| ADT-161 | 35 SAWYT_3125 | CIMMYT Mexico |
| ADT-162 | 35 SAWYT_3151 | CIMMYT Mexico |
| ADT-163 | 35 SAWYT_3175 | CIMMYT Mexico |
| ADT-164 | 35 SAWYT_3176 | CIMMYT Mexico |
| ADT-165 | 35 SAWYT_3178 | CIMMYT Mexico |
| ADT-166 | 35 SAWYT_3180 | CIMMYT Mexico |
| ADT-167 | 35 SAWYT_3200 | CIMMYT Mexico |
| ADT-168 | 35 SAWYT_3205 | CIMMYT Mexico |
| ADT-169 | 35 SAWYT_3219 | CIMMYT Mexico |
| ADT-170 | 35 SAWYT_3224 | CIMMYT Mexico |
| ADT-171 | 35 SAWYT_3155 | CIMMYT Mexico |
| ADT-172 | 35 SAWYT_3167 | CIMMYT Mexico |
| ADT-173 | 35 SAWYT_3173 | CIMMYT Mexico |
| ADT-174 | RAC 875/GW 322 | IARI New Delhi |
| ADT-175 | HD 3043/HD 3078 | IARI New Delhi |
| ADT-176 | HD 3070/HD 3076 | IARI New Delhi |
| ADT-177 | HD 3070/HD 3076 | IARI New Delhi |
| ADT-178 | HD 3070/HD 3078 | IARI New Delhi |
| ADT-179 | HD 3076/HD 3078 | IARI New Delhi |
| ADT-180 | HD 3076/HD 2781 | IARI New Delhi |
| ADT-181 | HD 3078/HD 3086 | IARI New Delhi |
| ADT-182 | HD 3078/HD 2781 | IARI New Delhi |
| ADT-183 | HD 3078/HD 2781 | IARI New Delhi |
| ADT-184 | HD 3086/HD 2781 | IARI New Delhi |
| ADT-185 | HD 2781/HI 1500 | IARI New Delhi |
| ADT-186 | HD 2733/HD 3016 | IARI New Delhi |
| ADT-187 | HD 2733/HD 3016 | IARI New Delhi |
| ADT-188 | HD 2967/HD 3043 | IARI New Delhi |
| ADT-189 | HD 2967/HD 3043 | IARI New Delhi |
| ADT-190 | HD 3087/DBW 17 | IARI New Delhi |
| ADT-191 | HD 3087/HD 3070 | IARI New Delhi |
| ADT-192 | HD 3087/GW 322 | IARI New Delhi |
| ADT-193 | HD 3087//HD 2967 | IARI New Delhi |
| ADT-194 | KAUZ/AA//KAUZ/HD 2733 | IARI New Delhi |
| ADT-195 | BERKUT/PBW 550 | IARI New Delhi |
| ADT-196 | MARFED_M1/2*SILVERSTAR// RHT 5/GW 366 | IARI New Delhi |
| ADT-197 | HD 2733/HD 3043 | IARI New Delhi |
| ADT-198 | HD 2733/HD 3076 | IARI New Delhi |
| ADT-199 | HD 2733/HI 1500 | IARI New Delhi |
| ADT-200 | HD 3043/HD 3076 | IARI New Delhi |
| ADT-201 | HD 3043/HD 3086 | IARI New Delhi |
| ADT-202 | HD 3043/HD 3086 | IARI New Delhi |
| ADT-203 | HD 3070/HD 2781 | IARI New Delhi |
| ADT-204 | HD 3070/HD 2781 | IARI New Delhi |
| ADT-205 | HD 3076/HD 3078 | IARI New Delhi |
| ADT-206 | HD 3076/HD 3086 | IARI New Delhi |
| ADT-207 | HD 3076/HD 2781 | IARI New Delhi |
| ADT-208 | KAUZ/AA//KAUZ/HD 3076 | IARI New Delhi |
| ADT-209 | HD 2967/HI 1500 | IARI New Delhi |
| ADT-210 | HD 3087/HD 3070 | IARI New Delhi |
| ADT-211 | HD 3087/HD 3070 | IARI New Delhi |
| ADT-212 | HD 2967/WH 760 | IARI New Delhi |
| ADT-213 | DL 788-2/RS 937//HW 4005 | IARI New Delhi |
| ADT-214 | KAUZ/HD 3016 | IARI New Delhi |
| ADT-215 | KAUZ/HD 3016 | IARI New Delhi |
| ADT-216 | GW 322/3/SOKOLL//FRTL/2*PIFED | IARI New Delhi |
| ADT-217 | RSC3_3/M43//WYALKATCHEM/HD 3076 | IARI New Delhi |
| ADT-218 | RSC3_3/M43//WYALKATCHEM/3/HD 3076 | IARI New Delhi |
| ADT-219 | RSC3_2/M43//WYALKATCHEM/3/HD3076 | IARI New Delhi |
| ADT-220 | HD 3043/HD 3076 | IARI New Delhi |
| ADT-221 | HD 3043/HD 3086 | IARI New Delhi |
| ADT-222 | HD 3043/HD 2781 | IARI New Delhi |
| ADT-223 | HD 3070/HD 3078 | IARI New Delhi |
| ADT-224 | HD 3070/HD 3078 | IARI New Delhi |
| ADT-225 | HD 3070/HD 3078 | IARI New Delhi |
| ADT-226 | HD 3070/HD 3078 | IARI New Delhi |
| ADT-227 | HD 3070/HD 3078 | IARI New Delhi |
| ADT-228 | HD 3070/HD 3078 | IARI New Delhi |
| ADT-229 | HD 3070/HI 1500 | IARI New Delhi |
| ADT-230 | HD 3070/HI 1500 | IARI New Delhi |
| ADT-231 | HD 3076/HD 3078 | IARI New Delhi |
| ADT-232 | HD 3076/HD 3078 | IARI New Delhi |
| ADT-233 | HD 3078/HD 3086 | IARI New Delhi |
| ADT-234 | HD 3078/HD 3086 | IARI New Delhi |
| ADT-235 | HD 3078/HD 2781 | IARI New Delhi |
| ADT-236 | HD 3086/HI 1500 | IARI New Delhi |
| ADT-237 | HD 2733/HD 3016 | IARI New Delhi |
| ADT-238 | HD 2733/HD 3016 | IARI New Delhi |
| ADT-239 | HD 2733/HD 3016 | IARI New Delhi |
| ADT-240 | HD 2733/HD 3016 | IARI New Delhi |
| ADT-241 | HD 2733/HD 3043 | IARI New Delhi |
| ADT-242 | HD 2733/HD 3043 | IARI New Delhi |
| ADT-243 | HD 2967/HD 3043 | IARI New Delhi |
| ADT-244 | HD 2967/HD 3043 | IARI New Delhi |
| ADT-245 | HD 2967/HD 3043 | IARI New Delhi |
| ADT-246 | HD 3087//HD 2967 | IARI New Delhi |
| ADT-247 | HD 3087//HD 2967 | IARI New Delhi |
| ADT-248 | HD 3087/PBW 343 | IARI New Delhi |
| ADT-249 | HD 3078/HD 3086 | IARI New Delhi |
| ADT-250 | HD 3078/HI 1500 | IARI New Delhi |
| ADT-251 | HD 3086/HI 1500 | IARI New Delhi |
| ADT-252 | HD 3086/HI 1500 | IARI New Delhi |
| ADT-253 | HD 2733/HD 3016 | IARI New Delhi |
| ADT-254 | HD 2967/HD 3043 | IARI New Delhi |
| ADT-255 | HD 3087//HD 2967 | IARI New Delhi |
| ADT-256 | HD 3043/HD 3078 | IARI New Delhi |
| ADT-257 | HD 3043/HD 3078 | IARI New Delhi |
| ADT-258 | HD 3043/HD 3078 | IARI New Delhi |
| ADT-259 | DBW44/HD3059 | IARI New Delhi |
| ADT-260 | DBW44/HD3059 | IARI New Delhi |
| ADT-261 | DBW44/HD3059 | IARI New Delhi |
| ADT-262 | DBW44/HD3059 | IARI New Delhi |
| ADT-263 | DBW44/HD3059 | IARI New Delhi |
| ADT-264 | DBW44/HD3059 | IARI New Delhi |
| ADT-265 | DBW44/HD3059 | IARI New Delhi |
| ADT-266 | DBW44/HD3059 | IARI New Delhi |
| ADT-267 | DBW44/HD2967 | IARI New Delhi |
| ADT-268 | DBW44/HD2967 | IARI New Delhi |
| ADT-269 | DBW44/HD2967 | IARI New Delhi |
| ADT-270 | DBW44/HD2967 | IARI New Delhi |
| ADT-271 | DBW44/HD2967 | IARI New Delhi |
| ADT-272 | DBW44/HD2967 | IARI New Delhi |
| ADT-273 | DBW44/HD2967 | IARI New Delhi |
| ADT-274 | DBW44/HD2967 | IARI New Delhi |
| ADT-275 | DBW44/HD2967 | IARI New Delhi |
| ADT-276 | DBW44/HD2967 | IARI New Delhi |
| ADT-277 | DBW44/HD2967 | IARI New Delhi |
| ADT-278 | DBW44/HD2967 | IARI New Delhi |
| ADT-279 | DBW43/HI1500 | IARI New Delhi |
| ADT-280 | DBW43/HI1500 | IARI New Delhi |
| ADT-281 | DBW43/HI1500 | IARI New Delhi |
| ADT-282 | DBW43/HI1500 | IARI New Delhi |
| ADT-283 | DBW43/HI1500 | IARI New Delhi |
| ADT-284 | DBW43/HI1500 | IARI New Delhi |
| ADT-285 | DBW43/HI1500 | IARI New Delhi |
| ADT-286 | DBW43/HI1500 | IARI New Delhi |
| ADT-287 | DBW43/HI1500 | IARI New Delhi |
| ADT-288 | DBW43/HI1500 | IARI New Delhi |
| ADT-289 | DBW43/HI1500 | IARI New Delhi |
| ADT-290 | DBW43/HI1500 | IARI New Delhi |
| ADT-291 | DBW43/HI1500 | IARI New Delhi |
| ADT-292 | DBW43/HI1500 | IARI New Delhi |
| ADT-293 | DBW43/HI1500 | IARI New Delhi |
| ADT-294 | DBW43/HI1500 | IARI New Delhi |
| ADT-295 | DBW43/HI1500 | IARI New Delhi |
