# Supplementary material for: Genome-wide association mapping for component traits of drought and heat tolerance in wheat
Source: Front Plant Sci. 2022 Aug 16;13:943033. doi: 10.3389/fpls.2022.943033 (PMC9429996; doi:10.3389/fpls.2022.943033)
Supplement: Supplementary file 1 [file Data_Sheet_1.ZIP › Supp.Table 2.docx]

Supplementary table 2. Descriptive statistics and ANOVA of all the studied traits across the year and locations

| Trait | Condition | Year | Location | Mean | SD | Min | Max | CV | CD | MSSe |
| --- | --- | --- | --- | --- | --- | --- | --- | --- | --- | --- |
| 1.DH | IR | 2019-20 rabi | IARI NewDelhi | 94.1 | 6.32 | 79.24 | 112.76 | 0.78 | 6.45 | 41.67 ** |
|  | IR | 2020-21 rabi | ARI Pune | 63.12 | 3.78 | 51.02 | 72.02 | 2.87 | 4.44 | 14.53 ** |
|  | IR | 2020-21 rabi | IARI NewDelhi | 94.99 | 6.45 | 75.21 | 113.96 | 2.2 | 5.13 | 40.84 ** |
|  | IR | 2020-21 rabi | IIWBR Karnal | 88.08 | 2.51 | 81.9 | 100.02 | 1.89 | 4.06 | 7.78 ** |
|  | LS | 2019-20 rabi | IARI NewDelhi | 93.3 | 2.99 | 85.5 | 100.5 | 0.9 | 3.23 | 8.4 ** |
|  | LS | 2020-21 rabi | IARI NewDelhi | 82.99 | 2.7 | 77.69 | 93.44 | 1.89 | 6.67 | 39.13 ** |
|  | RI | 2019-20 rabi | IARI NewDelhi | 94.38 | 6.49 | 75.5 | 107.5 | 1.77 | 9.33 | 6.13* |
|  | RI | 2020-21 rabi | ARI Pune | 58.36 | 2.95 | 50.48 | 68.48 | 2.55 | 5.08 | 12.05 ** |
|  | RI | 2020-21 rabi | IARI Jharkhand | 78.06 | 3.52 | 61.53 | 87.53 | 2.66 | 3.64 | 8.88 ** |
|  | RI | 2020-21 rabi | IARI NewDelhi | 97.84 | 6.37 | 77.71 | 115.46 | 2.79 | 3.84 | 7.53 ** |
| 2.PH | IR | 2019-20 rabi | IARI NewDelhi | 107.48 | 7.21 | 75.06 | 128.06 | 0.92 | 3.1 | 5.5 ** |
|  | IR | 2020-21 rabi | IARI NewDelhi | 108.12 | 6.25 | 87.91 | 125.12 | 3.71 | 9.83 | 37.8 ** |
|  | LS | 2020-21 rabi | IARI NewDelhi | 87.23 | 6.31 | 69.4 | 103.98 | 3.34 | 10.03 | 45.42 ** |
|  | RI | 2020-21 rabi | IARI Jharkhand | 90.61 | 7.08 | 64.74 | 112.74 | 5.73 | 12.7 | 52.46 ** |
|  | RI | 2020-21 rabi | IARI NewDelhi | 101.41 | 6.62 | 77.47 | 119.97 | 4.05 | 7.11 | 39.85 ** |
| 3.DM | IR | 2020-21 rabi | IARI NewDelhi | 133.23 | 3.84 | 123.81 | 144.81 | 1.22 | 3.98 | 14.28 ** |
|  | IR | 2020-21 rabi | IIWBR Karnal | 123.56 | 1.66 | 118.98 | 127.98 | 1.02 | 8.13 | 20.7 * |
|  | LS | 2020-21 rabi | IARI NewDelhi | 109.41 | 3.05 | 101.44 | 120.06 | 1.31 | 5.49 | 10.08 ** |
|  | RI | 2020-21 rabi | IARI Jharkhand | 112.18 | 3.99 | 101.67 | 124.94 | 2.97 | 3.09 | 3.09 ** |
|  | RI | 2020-21 rabi | IARI NewDelhi | 130.43 | 3.46 | 120.52 | 140.4 | 1.72 | 3.51 | 10.24 ** |
| 4.NDVI_1 | IR | 2019-20 rabi | IARI NewDelhi | 0.8 | 0.01 | 0.71 | 0.82 | 1.12 | 0.04 | 0.00021 ns |
|  | IR | 2020-21 rabi | ARI Pune | 0.86 | 0.04 | 0.71 | 0.96 | 3.89 | 0.1 | 0.0027 * |
|  | IR | 2020-21 rabi | IARI NewDelhi | 0.68 | 0.05 | 0.54 | 0.8 | 5.57 | 0.09 | 0.0024 * |
|  | IR | 2020-21 rabi | IIWBR Karnal | 0.6 | 0.09 | 0.26 | 0.82 | 11.18 | 0.17 | 0.01 * |
|  | LS | 2019-20 rabi | IARI NewDelhi | 0.81 | 0.02 | 0.71 | 0.85 | 0.89 | 3.92 | 1.04 ns |
|  | LS | 2020-21 rabi | IARI NewDelhi | 0.57 | 0.05 | 0.38 | 0.72 | 6.06 | 0.15 | 0.003 ns |
|  | RI | 2019-20 rabi | IARI NewDelhi | 0.8 | 0.01 | 0.71 | 0.83 | 1.25 | 0.07 | 0.12 ** |
|  | RI | 2020-21 rabi | ARI Pune | 0.85 | 0.05 | 0.68 | 0.97 | 4.87 | 0.08 | 0.0016 ns |
|  | RI | 2020-21 rabi | IARI NewDelhi | 0.64 | 0.05 | 0.51 | 0.82 | 7.95 | 0.08 | 0.002 * |
| 5.NDVI_2 | IR | 2019-20 rabi | IARI NewDelhi | 0.7 | 0.35 | 0.54 | 6.66 | 2.53 | 0.05 | 0.0017 ** |
|  | IR | 2020-21 rabi | ARI Pune | 0.83 | 0.04 | 0.69 | 0.94 | 4.69 | 0.11 | 0.0014 ns |
|  | IR | 2020-21 rabi | IARI NewDelhi | 0.59 | 0.06 | 0.43 | 0.74 | 7.3 | 0.11 | 0.0031 * |
|  | LS | 2019-20 rabi | IARI NewDelhi | 0.96 | 4.14 | 0.55 | 0.72 | 1.85 | 0.03 | 0.00047 * |
|  | LS | 2020-21 rabi | IARI NewDelhi | 0.42 | 0.07 | 0.22 | 0.6 | 15.19 | 0.13 | 0.0044 * |
|  | RI | 2019-20 rabi | IARI NewDelhi | 0.65 | 0.04 | 0.53 | 0.76 | 1.9 | 0.05 | 0.002 ** |
|  | RI | 2020-21 rabi | ARI Pune | 0.81 | 0.04 | 0.68 | 0.97 | 5.42 | 0.1 | 0.0018 ns |
|  | RI | 2020-21 rabi | IARI NewDelhi | 0.53 | 0.05 | 0.38 | 0.67 | 11.26 | 0.15 | 0.01 ns |
| 6.NDVI_3 | IR | 2019-20 rabi | IARI NewDelhi | 0.71 | 0.04 | 0.56 | 0.81 | 1.71 | 0.03 | 2e-04 ns |
|  | IR | 2020-21 rabi | IARI NewDelhi | 0.21 | 0.09 | 0.06 | 0.5 | 24.81 | 0.13 | 0.01 ** |
|  | LS | 2019-20 rabi | IARI NewDelhi | 0.4 | 0.12 | 0.07 | 0.72 | 12.96 | 0.31 | 0.03 * |
|  | LS | 2020-21 rabi | IARI NewDelhi | 0.2 | 0.07 | 0.09 | 0.56 | 22.1 | 0.1 | 0.01 ** |
|  | RI | 2020-21 rabi | IARI NewDelhi | 0.21 | 0.08 | 0.07 | 0.52 | 20.44 | 0.12 | 0.0029 ns |
| 7.SPAD | IR | 2020-21 rabi | IARI NewDelhi | 46.66 | 4.02 | 33.76 | 56.84 | 8.48 | 9.7 | 18.31 ns |
|  | LS | 2020-21 rabi | IARI NewDelhi | 49.75 | 4.32 | 36.55 | 60.79 | 7.04 | 6.01 | 10.82 * |
|  | RI | 2020-21 rabi | IARI NewDelhi | 52.31 | 4.71 | 35.34 | 62.24 | 8.21 | 8.6 | 13.48 ns |
| 8.CT | IR | 2020-21 rabi | ARI Pune | 26.91 | 1.17 | 24.07 | 29.98 | 3.91 | 2.43 | 1.22 ns |
|  | IR | 2020-21 rabi | IARI NewDelhi | 27.46 | 1.48 | 23.19 | 30.68 | 4.55 | 3.05 | 2.82 * |
|  | LS | 2019-20 rabi | IARI NewDelhi | 21.97 | 0.87 | 19.91 | 24.51 | 4.63 | 0.33 | 0.02 ns |
|  | LS | 2020-21 rabi | IARI NewDelhi | 29.01 | 1.39 | 26.27 | 39.02 | 4.18 | 2.49 | 1.41 ns |
|  | RI | 2020-21 rabi | ARI Pune | 27.93 | 1.06 | 25.43 | 30.24 | 3.56 | 2.58 | 1.27 ns |
|  | RI | 2020-21 rabi | IARI NewDelhi | 32.54 | 1.17 | 29.51 | 36.21 | 3.13 | 2.96 | 3.24 ** |
| 9.GWPS | IR | 2019-20 rabi | IARI NewDelhi | 2.29 | 0.74 | 1.10 | 4.48 | 3.46 | 3.82 | 5.31 ** |
|  | IR | 2020-21 rabi | ARI Pune | 1.47 | 0.13 | 1.05 | 1.82 | 9.74 | 0.69 | 0.14 * |
|  | IR | 2020-21 rabi | IARI NewDelhi | 1.98 | 0.44 | 0.71 | 4.06 | 23.99 | 0.63 | 0.11 * |
|  | IR | 2020-21 rabi | IIWBR Karnal | 2.07 | 0.32 | 1.32 | 3.09 | 16.74 | 1.17 | 0.19 |
|  | LS | 2019-20 rabi | IARI NewDelhi | 1.39 | 0.31 | 1.1 | 2.75 | 2.28 | 0.18 | 0.31 ** |
|  | LS | 2020-21 rabi | IARI NewDelhi | 1.68 | 0.3 | 0.95 | 2.9 | 13.52 | 0.32 | 0.02 |
|  | RI | 2019-20 rabi | IARI NewDelhi | 2.49 | 0.56 | 1.14 | 4.54 | 1.84 | 0.12 | 0.1 ** |
|  | RI | 2020-21 rabi | ARI Pune | 1.66 | 0.15 | 1.23 | 2.01 | 7.86 | 0.55 | 0.12 ** |
|  | RI | 2020-21 rabi | IARI Jharkhand | 2.2 | 0.34 | 0.9 | 3.21 | 11.69 | 0.85 | 0.1 |
|  | RI | 2020-21 rabi | IARI NewDelhi | 1.74 | 0.36 | 0.85 | 3.2 | 16.27 | 0.35 | 0.02 |
| 10.BIOMASS | IR | 2020-21 rabi | IARI NewDelhi | 1661.34 | 332.87 | 337.62 | 2775.25 | 15.36 | 622.08 | 103607.6 * |
|  | LS | 2020-21 rabi | IARI NewDelhi | 995.87 | 344.44 | 257 | 2267.6 | 31.53 | 505.65 | 96361.27 ** |
|  | RI | 2020-21 rabi | IARI Jharkhand | 320.49 | 98.54 | 42.75 | 627.75 | 28.11 | 222.66 | 12048.95 ns |
|  | RI | 2020-21 rabi | IARI NewDelhi | 1199.13 | 321.87 | 344 | 1937.15 | 17.05 | 760.91 | 100801.1 ns |
| 11.PLTY | IR | 2019-20 rabi | IARI NewDelhi | 350.96 | 75.79 | 75.12 | 477.56 | 15.47 | 2.83 | 40.17 ** |
|  | IR | 2020-21 rabi | ARI Pune | 186.5 | 45.68 | 41.45 | 321.8 | 14 | 109.96 | 2912.88 |
|  | IR | 2020-21 rabi | IARI NewDelhi | 484.53 | 103.21 | 101.77 | 708.65 | 17.61 | 209.88 | 11844.29 * |
|  | IR | 2020-21 rabi | IIWBR Karnal | 261.68 | 83.68 | 46.55 | 485.27 | 31.62 | 204.87 | 6455.32 |
|  | LS | 2019-20 rabi | IARI NewDelhi | 291.41 | 64.32 | 80.69 | 441.52 | 10.6 | 119.25 | 4139.6 |
|  | LS | 2020-21 rabi | IARI NewDelhi | 340.12 | 110.94 | 53.88 | 597.63 | 28.98 | 135.84 | 6305.63 ** |
|  | RI | 2020-21 rabi | ARI Pune | 92.31 | 33.67 | 19.04 | 215.16 | 33.49 | 107.17 | 4068.58 ** |
|  | RI | 2020-21 rabi | IARI Jharkhand | 163.85 | 48.18 | 13.48 | 286 | 27.2 | 63.38 | 2204.33 ** |
|  | RI | 2020-21 rabi | IARI NewDelhi | 300.78 | 86.3 | 81.5 | 560.21 | 18.38 | 243.84 | 12816.91 |
|  | RI | 2020-21 rabi | IARI RS Indore | 301.16 | 64.6 | 82.73 | 466.83 | 14.61 | 75.37 | 1185.38 |
| 12.TGW | IR | 2020-21 rabi | ARI Pune | 42.99 | 3.52 | 31.83 | 50.36 | 4.68 | 9.64 | 24.03 |
|  | IR | 2020-21 rabi | IARI NewDelhi | 38.82 | 4.43 | 24.16 | 57.28 | 10 | 4.89 | 12.33 ** |
|  | IR | 2020-21 rabi | IIWBR Karnal | 36.83 | 5.03 | 23.63 | 53.33 | 15.31 | 9.46 | 23.73 * |
|  | LS | 2020-21 rabi | IARI NewDelhi | 36.28 | 4.06 | 23.15 | 48.15 | 8.76 | 4.88 | 9.44 ** |
|  | RI | 2020-21 rabi | ARI Pune | 40.85 | 3.45 | 31.13 | 49.01 | 6.19 | 7.73 | 16.71 * |
|  | RI | 2020-21 rabi | IARI Jharkhand | 43.38 | 3.87 | 29.47 | 58.86 | 6.63 | 13.78 | 19.11 |
|  | RI | 2020-21 rabi | IARI NewDelhi | 32.99 | 4.91 | 20.98 | 50.1 | 11.99 | 6.99 | 15.65 ** |
|  | RI | 2020-21 rabi | IARI RS Indore | 44.38 | 3.15 | 36 | 53.62 | 4.52 | 6.15 | 11.12 * |
