# Supplementary material for: Genome-wide association mapping for component traits of drought and heat tolerance in wheat
Source: Front Plant Sci. 2022 Aug 16;13:943033. doi: 10.3389/fpls.2022.943033 (PMC9429996; doi:10.3389/fpls.2022.943033)
Supplement: Supplementary file 1 [file Data_Sheet_1.ZIP › Supp.Table 3.docx]

Supplementary Table 3. Genotypes belong to Population 1, 2 and admixtures population based on STRUCTURE analysis.

| Population | Population 1 | | | | Population 2 | | | | Admixture |
| --- | --- | --- | --- | --- | --- | --- | --- | --- | --- |
| Number of genotypes | **133** | | | | **135** | | | | **14** |
| Genotype name in each population | ADT_144 | ADT_40 | ADT_86 | ADT_138 | ADT_2 | ADT_224 | ADT_85 | ADT_243 | ADT_236 |
|  | ADT_158 | ADT_41 | ADT_87 | ADT_140 | ADT_3 | ADT_247 | ADT_105 | ADT_249 | ADT_259 |
|  | ADT_113 | ADT_43 | ADT_88 | ADT_147 | ADT_4 | ADT_248 | ADT_114 | ADT_261 | ADT_204 |
|  | ADT_46 | ADT_44 | ADT_89 | ADT_149 | ADT_5 | ADT_258 | ADT_119 | ADT_239 | ADT_237 |
|  | ADT_146 | ADT_48 | ADT_90 | ADT_150 | ADT_6 | ADT_260 | ADT_128 | ADT_210 | ADT_203 |
|  | ADT_148 | ADT_50 | ADT_91 | ADT_151 | ADT_8 | ADT_262 | ADT_145 | ADT_197 | ADT_227 |
|  | ADT_154 | ADT_51 | ADT_92 | ADT_153 | ADT_11 | ADT_263 | ADT_157 | ADT_198 | ADT_225 |
|  | ADT_152 | ADT_52 | ADT_93 | ADT_155 | ADT_13 | ADT_264 | ADT_159 | ADT_238 | ADT_228 |
|  | ADT_132 | ADT_53 | ADT_94 | ADT_160 | ADT_14 | ADT_265 | ADT_161 | ADT_208 | ADT_253 |
|  | ADT_39 | ADT_55 | ADT_95 | ADT_163 | ADT_17 | ADT_266 | ADT_169 | ADT_211 | ADT_234 |
|  | ADT_45 | ADT_56 | ADT_96 | ADT_164 | ADT_23 | ADT_268 | ADT_181 | ADT_215 | ADT_207 |
|  | ADT_141 | ADT_59 | ADT_98 | ADT_165 | ADT_24 | ADT_269 | ADT_185 | ADT_231 | ADT_252 |
|  | ADT_173 | ADT_60 | ADT_100 | ADT_166 | ADT_25 | ADT_270 | ADT_193 | ADT_195 | ADT_235 |
|  | ADT_42 | ADT_61 | ADT_101 | ADT_167 | ADT_30 | ADT_271 | ADT_230 | ADT_209 | ADT_250 |
|  | ADT_58 | ADT_63 | ADT_102 | ADT_168 | ADT_49 | ADT_274 | ADT_241 | ADT_255 |  |
|  | ADT_134 | ADT_65 | ADT_103 | ADT_170 | ADT_54 | ADT_276 | ADT_273 | ADT_240 |  |
|  | ADT_156 | ADT_66 | ADT_106 | ADT_171 | ADT_57 | ADT_278 | ADT_275 | ADT_256 |  |
|  | ADT_186 | ADT_67 | ADT_107 | ADT_172 | ADT_97 | ADT_279 | ADT_283 | ADT_216 |  |
|  | ADT_192 | ADT_68 | ADT_108 | ADT_175 | ADT_99 | ADT_280 | ADT_287 | ADT_233 |  |
|  | ADT_7 | ADT_69 | ADT_110 | ADT_176 | ADT_109 | ADT_281 | ADT_290 | ADT_222 |  |
|  | ADT_12 | ADT_70 | ADT_111 | ADT_177 | ADT_117 | ADT_282 | ADT_293 | ADT_200 |  |
|  | ADT_15 | ADT_71 | ADT_115 | ADT_178 | ADT_121 | ADT_286 | ADT_72 | ADT_219 |  |
|  | ADT_16 | ADT_73 | ADT_116 | ADT_180 | ADT_133 | ADT_288 | ADT_246 | ADT_242 |  |
|  | ADT_18 | ADT_74 | ADT_118 | ADT_182 | ADT_135 | ADT_289 | ADT_285 | ADT_251 |  |
|  | ADT_19 | ADT_75 | ADT_122 | ADT_183 | ADT_142 | ADT_292 | ADT_291 | ADT_245 |  |
|  | ADT_26 | ADT_76 | ADT_123 | ADT_184 | ADT_143 | ADT_295 | ADT_112 | ADT_257 |  |
|  | ADT_27 | ADT_77 | ADT_124 | ADT_187 | ADT_162 | ADT_1 | ADT_196 | ADT_201 |  |
|  | ADT_28 | ADT_78 | ADT_126 | ADT_188 | ADT_174 | ADT_20 | ADT_37 | ADT_217 |  |
|  | ADT_29 | ADT_79 | ADT_127 | ADT_189 | ADT_179 | ADT_21 | ADT_226 | ADT_232 |  |
|  | ADT_31 | ADT_80 | ADT_129 | ADT_190 | ADT_194 | ADT_22 | ADT_254 | ADT_244 |  |
|  | ADT_32 | ADT_81 | ADT_130 | ADT_191 | ADT_206 | ADT_33 | ADT_213 | ADT_202 |  |
|  | ADT_34 | ADT_82 | ADT_131 |  | ADT_218 | ADT_47 | ADT_212 | ADT_199 |  |
|  | ADT_35 | ADT_83 | ADT_136 |  | ADT_221 | ADT_62 | ADT_214 | ADT_220 |  |
|  | ADT_38 | ADT_84 | ADT_137 |  | ADT_223 | ADT_64 | ADT_125 |  |  |
