# Supplementary material for: Genome-wide association mapping for component traits of drought and heat tolerance in wheat
Source: Front Plant Sci. 2022 Aug 16;13:943033. doi: 10.3389/fpls.2022.943033 (PMC9429996; doi:10.3389/fpls.2022.943033)
Supplement: Supplementary file 1 [file Data_Sheet_1.ZIP › Supp.Table 4.docx]

Supplementary Table 4: Marker trait associations (MTAs) identified across the traits and environments having *-log(p)* value above 3.0

| Supplementary table 3. All SNPs above cut off p value 0.001 across the environment and traits. | | | | | |  |  |  |
| --- | --- | --- | --- | --- | --- | --- | --- | --- |
| Location | TRAIT | SNP | Chromosome | Position | P.value | maf | nobs | log(p) |
| IR_DL_2020 | BIOMASS | AX-94466450 | 6B | 29849235 | 4.67E-06 | 0.10106383 | 282 | 5.33088964 |
| IR_DL_2020 | BIOMASS | AX-94599608 | 6B | 30011466 | 7.23E-05 | 0.161347518 | 282 | 4.141107535 |
| IR_DL_2020 | BIOMASS | AX-95068836 | 7D | 543603637 | 9.48E-05 | 0.127659574 | 282 | 4.023131439 |
| IR_DL_2020 | BIOMASS | AX-95222290 | 7D | 543394160 | 0.000185694 | 0.226950355 | 282 | 3.731201512 |
| IR_DL_2020 | BIOMASS | AX-94923418 | 7B | 197701844 | 0.000255275 | 0.164893617 | 282 | 3.592991289 |
| IR_DL_2020 | BIOMASS | AX-94683465 | 3D | 98886921 | 0.000299591 | 0.106382979 | 282 | 3.523470817 |
| IR_DL_2020 | BIOMASS | AX-94394420 | 2A | 576067089 | 0.000371126 | 0.067375887 | 282 | 3.430479136 |
| IR_DL_2020 | BIOMASS | AX-94416376 | 4B | 602888633 | 0.000492251 | 0.466312057 | 282 | 3.307813481 |
| IR_DL_2020 | BIOMASS | AX-95008802 | 6D | 455829393 | 0.000771017 | 0.088652482 | 282 | 3.112935806 |
| IR_DL_2020 | BIOMASS | AX-94548288 | 7A | 624688056 | 0.000771107 | 0.242907801 | 282 | 3.112885618 |
| IR_DL_2020 | BIOMASS | AX-94730816 | 5A | 662290692 | 0.000791863 | 0.359929078 | 282 | 3.101349905 |
| IR_DL_2020 | BIOMASS | AX-94948666 | 2B | 412717695 | 0.000795297 | 0.171985816 | 282 | 3.099470794 |
| IR_DL_2020 | BIOMASS | AX-94619302 | 4D | 482707627 | 0.000822893 | 0.260638298 | 282 | 3.084656552 |
| IR_DL_2020 | BIOMASS | AX-94536530 | 4B | 602781575 | 0.000911183 | 0.377659574 | 282 | 3.040394597 |
| IR_DL_2020 | BIOMASS | AX-95249082 | 2B | 554455714 | 0.000968538 | 0.108156028 | 282 | 3.013883239 |
| IR_DL_2020 | BIOMASS | AX-94564902 | 1B | 646172198 | 9.12E-05 | 0.141843972 | 282 | 4.03994022 |
| IR_DL_2020 | BIOMASS | AX-95095643 | 7B | 544139827 | 9.70E-05 | 0.104609929 | 282 | 4.013099652 |
| IR_DL_2020 | BIOMASS | AX-94592974 | 1B | 448742632 | 0.000110709 | 0.469858156 | 282 | 3.955817673 |
| IR_DL_2020 | BIOMASS | AX-95016115 | 1D | 281379625 | 0.000304028 | 0.120567376 | 282 | 3.517086112 |
| IR_DL_2020 | BIOMASS | AX-94859360 | 5A | 544573346 | 0.000503952 | 0.379432624 | 282 | 3.297611011 |
| RI_JR_2020 | BIOMASS | AX-94569444 | 1B | 606190492 | 0.000257227 | 0.216312057 | 282 | 3.58968343 |
| RI_JR_2020 | BIOMASS | AX-94969778 | 1B | 531167036 | 0.000375425 | 0.246453901 | 282 | 3.425477023 |
| RI_JR_2020 | BIOMASS | AX-95008802 | 6D | 455829393 | 0.000744306 | 0.088652482 | 282 | 3.128248629 |
| LS_DL_2019 | CT | AX-94573298 | 7D | 629406870 | 3.01E-06 | 0.19858156 | 282 | 5.521181937 |
| LS_DL_2019 | CT | AX-94888072 | 5A | 442353496 | 4.55E-05 | 0.439716312 | 282 | 4.341630515 |
| LS_DL_2019 | CT | AX-94632287 | 2A | 507019394 | 0.000236678 | 0.113475177 | 282 | 3.625841575 |
| LS_DL_2019 | CT | AX-94827966 | 2A | 697302601 | 0.000326209 | 0.179078014 | 282 | 3.48650435 |
| LS_DL_2019 | CT | AX-94402889 | 2D | 601218303 | 0.000332595 | 0.191489362 | 282 | 3.478084481 |
| LS_DL_2019 | CT | AX-94826520 | 3A | 32252119 | 0.000664842 | 0.180851064 | 282 | 3.177281418 |
| LS_DL_2019 | CT | AX-95158169 | 4A | 584068369 | 0.000800357 | 0.209219858 | 282 | 3.096716007 |
| LS_DL_2019 | CT | AX-95203375 | 7A | 611289106 | 0.000855534 | 0.161347518 | 282 | 3.067762932 |
| LS_DL_2019 | CT | AX-94555026 | 3A | 730178232 | 0.000927152 | 0.274822695 | 282 | 3.032849229 |
| LS_DL_2019 | CT | AX-94524314 | 1B | 656852347 | 0.000994608 | 0.421985816 | 282 | 3.002348177 |
| LS_DL_2020 | CT | AX-95159175 | 6D | 4410712 | 0.000150953 | 0.312056738 | 282 | 3.821157246 |
| LS_DL_2020 | CT | AX-95181312 | 6D | 464649481 | 0.000237813 | 0.232269504 | 282 | 3.623763786 |
| LS_DL_2020 | CT | AX-94570860 | 4A | 277990747 | 0.000828384 | 0.191489362 | 282 | 3.081768317 |
| LS_DL_2020 | CT | AX-95182457 | 7B | 231709756 | 0.000933155 | 0.122340426 | 282 | 3.030046006 |
| RI_DL_2020 | CT | AX-94404276 | 4A | 476985345 | 0.000166824 | 0.384751773 | 282 | 3.777740748 |
| RI_DL_2020 | CT | AX-95196447 | 7A | 258412238 | 0.000279246 | 0.111702128 | 282 | 3.554013506 |
| RI_DL_2020 | CT | AX-94604134 | 2B | 42954039 | 0.000330578 | 0.484042553 | 282 | 3.480725688 |
| RI_DL_2020 | CT | AX-94875965 | 3A | 659040105 | 0.000481113 | 0.081560284 | 282 | 3.317752472 |
| RI_DL_2020 | CT | AX-94460734 | 2D | 354968144 | 0.000484825 | 0.225177305 | 282 | 3.314415207 |
| RI_DL_2020 | CT | AX-94600190 | 7D | 383974136 | 0.000560815 | 0.10106383 | 282 | 3.25118059 |
| RI_DL_2020 | CT | AX-94731563 | 2B | 767022557 | 0.000602217 | 0.30141844 | 282 | 3.220246952 |
| RI_DL_2020 | CT | AX-94598757 | 5B | 316562253 | 0.000625492 | 0.496453901 | 282 | 3.203778009 |
| RI_DL_2020 | CT | AX-94804881 | 2A | 376797767 | 0.000677705 | 0.265957447 | 282 | 3.168959342 |
| RI_DL_2020 | CT | AX-94606832 | 7D | 14881080 | 0.000857334 | 0.457446809 | 282 | 3.066849769 |
| RI_DL_2020 | CT | AX-94815711 | 7D | 203045993 | 0.000963717 | 0.113475177 | 282 | 3.016050579 |
| RI_DL_2019 | DH | AX-94941121 | 3A | 611702554 | 2.34E-09 | 0.070921986 | 282 | 8.630090633 |
| RI_DL_2019 | DH | AX-95119024 | 1D | 9592365 | 1.21E-06 | 0.164893617 | 282 | 5.917904173 |
| RI_DL_2019 | DH | AX-95133267 | 2D | 300437030 | 1.71E-06 | 0.436170213 | 282 | 5.766205235 |
| RI_DL_2019 | DH | AX-94645515 | 6B | 231676214 | 0.000407263 | 0.093971631 | 282 | 3.390125298 |
| RI_DL_2019 | DH | AX-94540968 | 6B | 201433807 | 0.00044644 | 0.095744681 | 282 | 3.350236816 |
| RI_DL_2019 | DH | AX-95177041 | 5A | 505503290 | 0.000514792 | 0.416666667 | 282 | 3.288368253 |
| RI_DL_2019 | DH | AX-94699669 | 3A | 656134941 | 0.000600475 | 0.120567376 | 282 | 3.221504763 |
| RI_DL_2019 | DH | AX-94542706 | 6B | 228976606 | 0.000612873 | 0.108156028 | 282 | 3.212629779 |
| RI_DL_2019 | DH | AX-94466796 | 3B | 487048835 | 0.000709432 | 0.093971631 | 282 | 3.149088994 |
| RI_DL_2019 | DH | AX-95016300 | 6A | 177265961 | 0.000773038 | 0.090425532 | 282 | 3.111799227 |
| RI_DL_2019 | DH | AX-94843879 | 6B | 175719158 | 0.000777733 | 0.079787234 | 282 | 3.109169737 |
| RI_DL_2019 | DH | AX-94669520 | 3D | 3301672 | 0.000897474 | 0.471631206 | 282 | 3.046978041 |
| RI_DL_2019 | DH | AX-95183088 | 6B | 232168465 | 0.000901 | 0.163120567 | 282 | 3.045275029 |
| RI_DL_2019 | DH | AX-94570446 | 6D | 7421603 | 0.000922158 | 0.09751773 | 282 | 3.035194889 |
| RI_DL_2019 | DH | AX-94497226 | 5D | 12990535 | 0.000924543 | 0.106382979 | 282 | 3.034072769 |
| RI_DL_2019 | DH | AX-95108215 | 5D | 552039504 | 0.000976845 | 0.127659574 | 282 | 3.010174224 |
| RI_DL_2019 | DH | AX-95133267 | 2D | 300437030 | 5.91E-06 | 0.436170213 | 282 | 5.228266986 |
| RI_DL_2019 | DH | AX-94442168 | 7A | 471965858 | 1.77E-05 | 0.15070922 | 282 | 4.751639663 |
| RI_DL_2019 | DH | AX-95235622 | 1D | 314574223 | 0.000102991 | 0.058510638 | 282 | 3.98720142 |
| RI_DL_2019 | DH | AX-94425642 | 1D | 258388240 | 0.000133575 | 0.244680851 | 282 | 3.874274608 |
| RI_DL_2019 | DH | AX-94633096 | 1D | 7211883 | 0.000349146 | 0.329787234 | 282 | 3.456993278 |
| RI_DL_2019 | DH | AX-94947434 | 4B | 24051570 | 0.000356054 | 0.079787234 | 282 | 3.448483556 |
| RI_DL_2019 | DH | AX-94733613 | 2B | 768613714 | 0.000486586 | 0.09929078 | 282 | 3.312840497 |
| RI_DL_2019 | DH | AX-94773234 | 1A | 14109176 | 0.0005435 | 0.085106383 | 282 | 3.264800533 |
| RI_DL_2019 | DH | AX-94457937 | 3B | 720217631 | 0.000554374 | 0.274822695 | 282 | 3.256196844 |
| RI_DL_2019 | DH | AX-94654258 | 3B | 720267471 | 0.00065809 | 0.210992908 | 282 | 3.181714868 |
| RI_DL_2019 | DH | AX-94544185 | 4A | 684076698 | 0.000805392 | 0.083333333 | 282 | 3.093992484 |
| RI_DL_2019 | DH | AX-94601848 | 4A | 684269297 | 0.000888437 | 0.088652482 | 282 | 3.051373434 |
| RI_DL_2019 | DH | AX-94521391 | 4B | 650730152 | 0.000934234 | 0.171985816 | 282 | 3.029544256 |
| RI_PUNE_2020 | DH | AX-94941121 | 3A | 611702554 | 2.77E-10 | 0.070921986 | 282 | 9.557035412 |
| RI_PUNE_2020 | DH | AX-94435238 | 5D | 474205125 | 7.68E-10 | 0.113475177 | 282 | 9.114737459 |
| RI_PUNE_2020 | DH | AX-94578563 | 3A | 55743418 | 1.78E-07 | 0.212765957 | 282 | 6.749976139 |
| RI_PUNE_2020 | DH | AX-94631711 | 3A | 26469875 | 0.000138845 | 0.141843972 | 282 | 3.857468618 |
| RI_PUNE_2020 | DH | AX-95112943 | 5B | 437802635 | 0.000293747 | 0.354609929 | 282 | 3.532026836 |
| RI_PUNE_2020 | DH | AX-94913145 | 7A | 36859962 | 0.000313867 | 0.127659574 | 282 | 3.503254466 |
| RI_PUNE_2020 | DH | AX-95174190 | 6A | 51760625 | 0.000340344 | 0.274822695 | 282 | 3.468082257 |
| RI_PUNE_2020 | DH | AX-94985384 | 7D | 580500491 | 0.000355186 | 0.345744681 | 282 | 3.449543676 |
| RI_PUNE_2020 | DH | AX-94587603 | 7D | 102900636 | 0.000372414 | 0.391843972 | 282 | 3.42897354 |
| RI_PUNE_2020 | DH | AX-94749760 | 7A | 670781770 | 0.000621999 | 0.372340426 | 282 | 3.206210192 |
| RI_PUNE_2020 | DH | AX-94961629 | 6A | 75368220 | 0.000645779 | 0.407801418 | 282 | 3.189915754 |
| RI_PUNE_2020 | DH | AX-94449174 | 7A | 36799642 | 0.000652708 | 0.067375887 | 282 | 3.185280757 |
| RI_PUNE_2020 | DH | AX-95143253 | 5B | 54702702 | 0.000822113 | 0.180851064 | 282 | 3.085068458 |
| RI_JR_2020 | DH | AX-95235622 | 1D | 314574223 | 3.25E-10 | 0.058510638 | 282 | 9.488191639 |
| RI_JR_2020 | DH | AX-94607578 | 2A | 759732438 | 8.09E-06 | 0.34929078 | 282 | 5.092239786 |
| RI_JR_2020 | DH | AX-95121939 | 7A | 19421444 | 0.000135891 | 0.161347518 | 282 | 3.86681047 |
| RI_JR_2020 | DH | AX-94569570 | 7B | 152075006 | 0.000136343 | 0.078014184 | 282 | 3.865366852 |
| RI_JR_2020 | DH | AX-94482893 | 3A | 1383538 | 0.000163581 | 0.44858156 | 282 | 3.786267218 |
| RI_JR_2020 | DH | AX-94797710 | 5B | 572591509 | 0.000176423 | 0.092198582 | 282 | 3.753445489 |
| RI_JR_2020 | DH | AX-94405550 | 1D | 488576782 | 0.000256031 | 0.132978723 | 282 | 3.591707132 |
| RI_JR_2020 | DH | AX-94855160 | 2B | 573200485 | 0.000261682 | 0.088652482 | 282 | 3.582225325 |
| RI_JR_2020 | DH | AX-94540157 | 2B | 573594138 | 0.000276207 | 0.083333333 | 282 | 3.558765736 |
| RI_JR_2020 | DH | AX-94623585 | 6B | 26863553 | 0.000340913 | 0.138297872 | 282 | 3.4673563 |
| RI_JR_2020 | DH | AX-94924843 | 5D | 484411118 | 0.000403567 | 0.10106383 | 282 | 3.394084278 |
| RI_JR_2020 | DH | AX-94476859 | 3A | 57568625 | 0.000706574 | 0.308510638 | 282 | 3.150842555 |
| RI_JR_2020 | DH | AX-95092172 | 2D | 649781103 | 0.000733186 | 0.138297872 | 282 | 3.134785805 |
| LS_DL_2019 | DH | AX-94988124 | 5B | 489283456 | 8.49E-05 | 0.25 | 282 | 4.071230551 |
| LS_DL_2019 | DH | AX-94560091 | 2B | 661450110 | 0.00022977 | 0.278368794 | 282 | 3.638707116 |
| LS_DL_2019 | DH | AX-94904781 | 5B | 639737622 | 0.000244067 | 0.290780142 | 282 | 3.612491785 |
| LS_DL_2019 | DH | AX-94894230 | 5D | 403066896 | 0.000245272 | 0.209219858 | 282 | 3.610352342 |
| LS_DL_2019 | DH | AX-94715499 | 2D | 514658801 | 0.000373517 | 0.326241135 | 282 | 3.427689886 |
| LS_DL_2019 | DH | AX-95133267 | 2D | 300437030 | 0.00038371 | 0.436170213 | 282 | 3.415997125 |
| LS_DL_2019 | DH | AX-94738780 | 7A | 709405190 | 0.000390586 | 0.218085106 | 282 | 3.40828341 |
| LS_DL_2019 | DH | AX-95235622 | 1D | 314574223 | 0.000398608 | 0.058510638 | 282 | 3.39945352 |
| LS_DL_2019 | DH | AX-94797710 | 5B | 572591509 | 0.000422296 | 0.092198582 | 282 | 3.374383529 |
| LS_DL_2019 | DH | AX-94414057 | 2B | 648922556 | 0.000439723 | 0.352836879 | 282 | 3.35682067 |
| LS_DL_2019 | DH | AX-94957845 | 1B | 428069284 | 0.000477986 | 0.056737589 | 282 | 3.320585236 |
| LS_DL_2019 | DH | AX-94642295 | 5B | 572547199 | 0.000574386 | 0.09751773 | 282 | 3.240796145 |
| LS_DL_2019 | DH | AX-94637995 | 2D | 62401677 | 0.000609713 | 0.459219858 | 282 | 3.214874408 |
| LS_DL_2019 | DH | AX-94986137 | 7D | 616903248 | 0.000674907 | 0.322695035 | 282 | 3.170755768 |
| LS_DL_2019 | DH | AX-94459800 | 2B | 52911848 | 0.000700798 | 0.480496454 | 282 | 3.154407363 |
| LS_DL_2019 | DH | AX-94733613 | 2B | 768613714 | 0.000738307 | 0.09929078 | 282 | 3.131763066 |
| LS_DL_2019 | DH | AX-95182756 | 2A | 699131127 | 0.000777657 | 0.352836879 | 282 | 3.109212085 |
| LS_DL_2019 | DH | AX-95075429 | 1B | 426072599 | 0.000794665 | 0.060283688 | 282 | 3.099816035 |
| LS_DL_2019 | DH | AX-94490490 | 2B | 759818034 | 0.000842821 | 0.491134752 | 282 | 3.074264906 |
| LS_DL_2019 | DH | AX-94724650 | 2B | 657854248 | 0.000851742 | 0.379432624 | 282 | 3.069691816 |
| LS_DL_2019 | DH | AX-95069434 | 2B | 660156481 | 0.00091366 | 0.326241135 | 282 | 3.039215224 |
| LS_DL_2019 | DH | AX-94699669 | 3A | 656134941 | 0.000945748 | 0.120567376 | 282 | 3.024224378 |
| LS_DL_2020 | DH | AX-94416489 | 3A | 46713296 | 1.51E-06 | 0.161347518 | 282 | 5.820014431 |
| LS_DL_2020 | DH | AX-94940654 | 5D | 367435977 | 4.17E-06 | 0.159574468 | 282 | 5.379682099 |
| LS_DL_2020 | DH | AX-94608035 | 5B | 333027851 | 1.51E-05 | 0.484042553 | 282 | 4.820539181 |
| LS_DL_2020 | DH | AX-94725580 | 5B | 594869106 | 1.90E-05 | 0.237588652 | 282 | 4.722284981 |
| LS_DL_2020 | DH | AX-94423939 | 3B | 721039461 | 0.000156515 | 0.072695035 | 282 | 3.805443248 |
| LS_DL_2020 | DH | AX-94540417 | 1B | 431456792 | 0.000464768 | 0.058510638 | 282 | 3.332763461 |
| LS_DL_2020 | DH | AX-94642374 | 5B | 130914061 | 0.000490508 | 0.195035461 | 282 | 3.30935387 |
| LS_DL_2020 | DH | AX-94442680 | 5B | 142002548 | 0.000499097 | 0.333333333 | 282 | 3.301815252 |
| LS_DL_2020 | DH | AX-95235622 | 1D | 314574223 | 0.000571745 | 0.058510638 | 282 | 3.242797683 |
| LS_DL_2020 | DH | AX-94868639 | 2B | 549038490 | 0.000678602 | 0.118794326 | 282 | 3.168384896 |
| LS_DL_2020 | DH | AX-95124335 | 2D | 52232863 | 0.000703613 | 0.117021277 | 282 | 3.152666082 |
| LS_DL_2020 | DH | AX-94464933 | 5B | 137294096 | 0.000899152 | 0.335106383 | 282 | 3.046166662 |
| LS_DL_2020 | DH | AX-94382472 | 1B | 387306189 | 0.00090466 | 0.106382979 | 282 | 3.043514682 |
| LS_DL_2020 | DH | AX-94536572 | 5A | 116573052 | 0.000905748 | 0.290780142 | 282 | 3.042992767 |
| LS_DL_2020 | DH | AX-94425642 | 1D | 258388240 | 0.000967045 | 0.244680851 | 282 | 3.014553524 |
| IR_DL_2019 | DH | AX-94856367 | 2D | 18232583 | 4.73E-05 | 0.203900709 | 282 | 4.325590945 |
| IR_DL_2019 | DH | AX-94910314 | 7B | 604328137 | 0.000161075 | 0.145390071 | 282 | 3.792970523 |
| IR_DL_2019 | DH | AX-94637066 | 3A | 7279031 | 0.000475644 | 0.212765957 | 282 | 3.322718033 |
| IR_DL_2019 | DH | AX-94703466 | 7D | 550102157 | 0.00062528 | 0.134751773 | 282 | 3.203925772 |
| IR_DL_2019 | DH | AX-94777130 | 3B | 771178846 | 0.00067775 | 0.19858156 | 282 | 3.168930689 |
| IR_DL_2019 | DH | AX-95254965 | 7A | 92799665 | 0.000890479 | 0.159574468 | 282 | 3.050376257 |
| IR_DL_2019 | DH | AX-94734044 | 7D | 535728143 | 0.0009037 | 0.196808511 | 282 | 3.043975644 |
| IR_DL_2019 | DH | AX-94416376 | 4B | 602888633 | 0.000911973 | 0.466312057 | 282 | 3.040018169 |
| IR_DL_2019 | DH | AX-94649275 | 5B | 670117211 | 0.000987951 | 0.088652482 | 282 | 3.005264648 |
| IR_DL_2020 | DH | AX-94689491 | 3A | 618164627 | 9.18E-10 | 0.067375887 | 282 | 9.037011864 |
| IR_DL_2020 | DH | AX-95195332 | 6D | 304116703 | 1.59E-09 | 0.078014184 | 282 | 8.798271085 |
| IR_DL_2020 | DH | AX-95170512 | 2D | 634316706 | 4.74E-07 | 0.44858156 | 282 | 6.323974945 |
| IR_DL_2020 | DH | AX-94637995 | 2D | 62401677 | 6.94E-06 | 0.459219858 | 282 | 5.158629644 |
| IR_DL_2020 | DH | AX-94463626 | 5B | 580840141 | 2.19E-05 | 0.086879433 | 282 | 4.660147884 |
| IR_DL_2020 | DH | AX-94688295 | 4A | 713505714 | 2.41E-05 | 0.157801418 | 282 | 4.617575618 |
| IR_DL_2020 | DH | AX-94797710 | 5B | 572591509 | 5.70E-05 | 0.092198582 | 282 | 4.243966657 |
| IR_DL_2020 | DH | AX-95223242 | 6B | 128550007 | 0.000157843 | 0.129432624 | 282 | 3.801775088 |
| IR_DL_2020 | DH | AX-94830402 | 2D | 620296445 | 0.000257708 | 0.154255319 | 282 | 3.588872527 |
| IR_DL_2020 | DH | AX-94915562 | 5B | 167293601 | 0.000346255 | 0.368794326 | 282 | 3.460604319 |
| IR_DL_2020 | DH | AX-94482893 | 3A | 1383538 | 0.00037249 | 0.44858156 | 282 | 3.428885339 |
| IR_DL_2020 | DH | AX-94454269 | 5B | 223124132 | 0.000462326 | 0.315602837 | 282 | 3.335052108 |
| IR_DL_2020 | DH | AX-94582138 | 5B | 243916628 | 0.000465311 | 0.331560284 | 282 | 3.332256866 |
| IR_DL_2020 | DH | AX-94564844 | 5B | 173858189 | 0.000479473 | 0.320921986 | 282 | 3.31923614 |
| IR_DL_2020 | DH | AX-94460640 | 5D | 220098422 | 0.000581936 | 0.179078014 | 282 | 3.235124571 |
| IR_DL_2020 | DH | AX-94430767 | 5B | 243433888 | 0.000651904 | 0.257092199 | 282 | 3.185816337 |
| IR_DL_2020 | DH | AX-94569846 | 5B | 220813321 | 0.000653994 | 0.331560284 | 282 | 3.184425931 |
| IR_DL_2020 | DH | AX-94415272 | 5B | 208649141 | 0.000690622 | 0.324468085 | 282 | 3.160759669 |
| IR_DL_2020 | DH | AX-94405391 | 1A | 261373106 | 0.000705751 | 0.430851064 | 282 | 3.151348592 |
| IR_DL_2020 | DH | AX-94435091 | 5B | 236258687 | 0.000712869 | 0.267730496 | 282 | 3.146990403 |
| IR_DL_2020 | DH | AX-94661228 | 5B | 243849816 | 0.000767317 | 0.242907801 | 282 | 3.115024916 |
| IR_DL_2020 | DH | AX-94389450 | 1A | 480599314 | 0.000797952 | 0.49822695 | 282 | 3.098023131 |
| IR_DL_2020 | DH | AX-95150920 | 6D | 20311503 | 0.000854138 | 0.216312057 | 282 | 3.06847189 |
| IR_DL_2020 | DH | AX-94490240 | 6D | 462536965 | 0.000902019 | 0.090425532 | 282 | 3.044784074 |
| IR_DL_2020 | DH | AX-94717163 | 2D | 381568187 | 0.000983304 | 0.088652482 | 282 | 3.007312192 |
| IR_DL_2020 | DH | AX-94634768 | 6B | 34340648 | 0.000985711 | 0.209219858 | 282 | 3.006250537 |
| IR_IIWBR_2020 | DH | AX-94546495 | 2B | 671741070 | 1.26E-08 | 0.390070922 | 282 | 7.900782827 |
| IR_IIWBR_2020 | DH | AX-94789937 | 7B | 33533863 | 4.13E-07 | 0.464539007 | 282 | 6.384130696 |
| IR_IIWBR_2020 | DH | AX-94664333 | 6B | 716010011 | 0.000337798 | 0.320921986 | 282 | 3.471342577 |
| IR_IIWBR_2020 | DH | AX-95186991 | 2A | 748537792 | 0.000685344 | 0.466312057 | 282 | 3.164091483 |
| IR_IIWBR_2020 | DH | AX-95223242 | 6B | 128550007 | 0.000862221 | 0.129432624 | 282 | 3.064381477 |
| IR_IIWBR_2020 | DH | AX-94878458 | 1D | 11548335 | 0.000935348 | 0.072695035 | 282 | 3.02902692 |
| IR_IIWBR_2020 | DH | AX-94470747 | 7D | 5277330 | 0.000948305 | 0.072695035 | 282 | 3.02305211 |
| IR_PUNE_2020 | DH | AX-94941121 | 3A | 611702554 | 3.28E-11 | 0.070921986 | 282 | 10.48453217 |
| IR_PUNE_2020 | DH | AX-94435238 | 5D | 474205125 | 3.92E-09 | 0.113475177 | 282 | 8.407156853 |
| IR_PUNE_2020 | DH | AX-94578563 | 3A | 55743418 | 1.40E-08 | 0.212765957 | 282 | 7.852821612 |
| IR_PUNE_2020 | DH | AX-94631711 | 3A | 26469875 | 3.07E-06 | 0.141843972 | 282 | 5.513008848 |
| IR_PUNE_2020 | DH | AX-95143253 | 5B | 54702702 | 0.000222668 | 0.180851064 | 282 | 3.652341738 |
| IR_PUNE_2020 | DH | AX-94749760 | 7A | 670781770 | 0.000297843 | 0.372340426 | 282 | 3.526012126 |
| IR_PUNE_2020 | DH | AX-94622619 | 7B | 24401137 | 0.000886808 | 0.381205674 | 282 | 3.052170276 |
| IR_DL_2020 | DM | AX-94463626 | 5B | 580840141 | 1.66E-10 | 0.086879433 | 282 | 9.780336908 |
| IR_DL_2020 | DM | AX-94490240 | 6D | 462536965 | 9.50E-08 | 0.090425532 | 282 | 7.022311462 |
| IR_DL_2020 | DM | AX-94513007 | 6D | 147239252 | 3.43E-07 | 0.179078014 | 282 | 6.465213492 |
| IR_DL_2020 | DM | AX-95186230 | 2D | 354743665 | 2.43E-06 | 0.125886525 | 282 | 5.615056864 |
| IR_DL_2020 | DM | AX-94634533 | 6A | 25759606 | 0.000247961 | 0.212765957 | 282 | 3.605617345 |
| IR_DL_2020 | DM | AX-94634768 | 6B | 34340648 | 0.000261753 | 0.209219858 | 282 | 3.582107617 |
| IR_DL_2020 | DM | AX-94744525 | 6A | 21519247 | 0.000292661 | 0.232269504 | 282 | 3.533635333 |
| IR_DL_2020 | DM | AX-94556600 | 5A | 33129747 | 0.000457933 | 0.117021277 | 282 | 3.339197871 |
| IR_DL_2020 | DM | AX-94637995 | 2D | 62401677 | 0.000618928 | 0.459219858 | 282 | 3.208359673 |
| IR_DL_2020 | DM | AX-94879742 | 6B | 40456334 | 0.000681027 | 0.269503546 | 282 | 3.166835622 |
| IR_DL_2020 | DM | AX-95172815 | 6A | 18713168 | 0.000822148 | 0.283687943 | 282 | 3.085050129 |
| IR_DL_2020 | DM | AX-94570104 | 1B | 17386404 | 0.000951376 | 0.104609929 | 282 | 3.021647869 |
| LS_DL_2020 | DM | AX-94725580 | 5B | 594869106 | 2.69E-08 | 0.237588652 | 282 | 7.570520583 |
| LS_DL_2020 | DM | AX-94397013 | 4D | 16233130 | 0.000294362 | 0.102836879 | 282 | 3.531118748 |
| LS_DL_2020 | DM | AX-94960755 | 2D | 81968722 | 0.000697238 | 0.196808511 | 282 | 3.156618643 |
| LS_DL_2020 | DM | AX-94664333 | 6B | 716010011 | 0.000730651 | 0.320921986 | 282 | 3.136290016 |
| LS_DL_2020 | DM | AX-95132838 | 5D | 382496233 | 0.000873824 | 0.484042553 | 282 | 3.05857595 |
| RI_DL_2020 | DM | AX-94442168 | 7A | 471965858 | 0.000465301 | 0.15070922 | 282 | 3.332265622 |
| RI_DL_2020 | DM | AX-94945333 | 1B | 198643185 | 0.000842992 | 0.159574468 | 282 | 3.074176628 |
| IR_IIWBR_2020 | DM | AX-94725580 | 5B | 594869106 | 3.20E-08 | 0.237588652 | 282 | 7.494575468 |
| IR_IIWBR_2020 | DM | AX-95133034 | 5A | 1275128 | 0.000316151 | 0.207446809 | 282 | 3.500105218 |
| IR_IIWBR_2020 | DM | AX-94604156 | 2D | 602110005 | 0.000351369 | 0.407801418 | 282 | 3.454235992 |
| IR_IIWBR_2020 | DM | AX-94618336 | 6B | 712174561 | 0.000456211 | 0.170212766 | 282 | 3.340834205 |
| IR_IIWBR_2020 | DM | AX-95219129 | 5D | 538441465 | 0.000529558 | 0.157801418 | 282 | 3.276086424 |
| IR_IIWBR_2020 | DM | AX-94727602 | 5B | 679557585 | 0.000728499 | 0.106382979 | 282 | 3.137571032 |
| IR_IIWBR_2020 | DM | AX-95195991 | 3B | 820139313 | 0.000827121 | 0.134751773 | 282 | 3.082431031 |
| IR_DL_2019 | GWPS | AX-94664052 | 2B | 748152562 | 2.30E-06 | 0.397163121 | 282 | 5.637959208 |
| IR_DL_2019 | GWPS | AX-94988124 | 5B | 489283456 | 4.97E-06 | 0.25 | 282 | 5.303543087 |
| IR_DL_2019 | GWPS | AX-94799208 | 3A | 61308293 | 0.000187613 | 0.157801418 | 282 | 3.726737291 |
| IR_DL_2019 | GWPS | AX-94944372 | 1B | 688283156 | 0.000255736 | 0.280141844 | 282 | 3.592207711 |
| IR_DL_2019 | GWPS | AX-95081630 | 3B | 4325637 | 0.000446574 | 0.085106383 | 282 | 3.350106857 |
| IR_DL_2019 | GWPS | AX-94862432 | 5A | 148831004 | 0.000505769 | 0.074468085 | 282 | 3.296047906 |
| IR_DL_2019 | GWPS | AX-94987428 | 1B | 604147399 | 0.000523815 | 0.147163121 | 282 | 3.28082187 |
| IR_DL_2019 | GWPS | AX-95010150 | 3D | 2909769 | 0.000615764 | 0.163120567 | 282 | 3.210585922 |
| IR_DL_2019 | GWPS | AX-94674294 | 1B | 379387740 | 0.000842771 | 0.120567376 | 282 | 3.074290466 |
| IR_DL_2019 | GWPS | AX-95239994 | 1D | 109980446 | 0.000960572 | 0.338652482 | 282 | 3.017470126 |
| IR_DL_2019 | GWPS | AX-94599278 | 7B | 428491808 | 0.000993319 | 0.063829787 | 282 | 3.002911354 |
| IR_DL_2019 | GWPS | AX-94681274 | 7A | 475804567 | 0.000993319 | 0.063829787 | 282 | 3.002911354 |
| LS_DL_2019 | GWPS | AX-94505180 | 2A | 733091174 | 1.64E-07 | 0.464539007 | 282 | 6.785045945 |
| LS_DL_2019 | GWPS | AX-95118281 | 5A | 624681005 | 7.03E-06 | 0.109929078 | 282 | 5.152777977 |
| LS_DL_2019 | GWPS | AX-94988045 | 7A | 20426852 | 3.67E-05 | 0.180851064 | 282 | 4.435894005 |
| LS_DL_2019 | GWPS | AX-94631122 | 3A | 727224468 | 0.000218087 | 0.453900709 | 282 | 3.661370994 |
| LS_DL_2019 | GWPS | AX-94938436 | 4B | 440328201 | 0.000452577 | 0.170212766 | 282 | 3.344307283 |
| LS_DL_2019 | GWPS | AX-94503208 | 1A | 584478259 | 0.000476131 | 0.489361702 | 282 | 3.322273215 |
| LS_DL_2019 | GWPS | AX-94521760 | 1A | 584469113 | 0.000632983 | 0.457446809 | 282 | 3.198607877 |
| LS_DL_2019 | GWPS | AX-94910420 | 2A | 740070870 | 0.000662515 | 0.317375887 | 282 | 3.178804574 |
| LS_DL_2019 | GWPS | AX-95238241 | 4B | 443453538 | 0.000737186 | 0.140070922 | 282 | 3.13242291 |
| LS_DL_2019 | GWPS | AX-94633968 | 2D | 107401280 | 0.000774448 | 0.117021277 | 282 | 3.111007724 |
| LS_DL_2019 | GWPS | AX-95245878 | 7B | 653894020 | 0.00084183 | 0.122340426 | 282 | 3.074775532 |
| RI_DL_2019 | GWPS | AX-94590453 | 3A | 8325489 | 2.69E-08 | 0.154255319 | 282 | 7.569559354 |
| RI_DL_2019 | GWPS | AX-95150902 | 7B | 200922847 | 2.36E-07 | 0.342198582 | 282 | 6.627890065 |
| RI_DL_2019 | GWPS | AX-94850054 | 3A | 669567947 | 4.95E-06 | 0.060283688 | 282 | 5.305762591 |
| RI_DL_2019 | GWPS | AX-94651230 | 4D | 2769913 | 0.000192224 | 0.257092199 | 282 | 3.716193004 |
| RI_DL_2019 | GWPS | AX-94728078 | 6D | 306203239 | 0.000286942 | 0.15248227 | 282 | 3.542206061 |
| RI_DL_2019 | GWPS | AX-94494832 | 4B | 3735541 | 0.000463453 | 0.368794326 | 282 | 3.333994296 |
| RI_DL_2019 | GWPS | AX-94789404 | 4A | 601357886 | 0.000569857 | 0.269503546 | 282 | 3.244234118 |
| IR_DL_2020 | GWPS | AX-95116004 | 3B | 83883781 | 1.04E-05 | 0.058510638 | 282 | 4.983781355 |
| IR_DL_2020 | GWPS | AX-94842429 | 3A | 749404301 | 2.90E-05 | 0.062056738 | 282 | 4.537712575 |
| IR_DL_2020 | GWPS | AX-94454033 | 3A | 107738831 | 5.23E-05 | 0.063829787 | 282 | 4.281801401 |
| IR_DL_2020 | GWPS | AX-95095284 | 3D | 59294594 | 9.73E-05 | 0.078014184 | 282 | 4.011881123 |
| IR_DL_2020 | GWPS | AX-94527643 | 2A | 97842216 | 0.000140972 | 0.283687943 | 282 | 3.850866639 |
| IR_DL_2020 | GWPS | AX-95119561 | 3D | 25784323 | 0.000205568 | 0.170212766 | 282 | 3.687044832 |
| IR_DL_2020 | GWPS | AX-94492134 | 3A | 106435192 | 0.000214916 | 0.058510638 | 282 | 3.667730546 |
| IR_DL_2020 | GWPS | AX-95119427 | 6B | 698417045 | 0.000223247 | 0.25 | 282 | 3.651214762 |
| IR_DL_2020 | GWPS | AX-94522843 | 4A | 597909728 | 0.000226786 | 0.154255319 | 282 | 3.64438408 |
| IR_DL_2020 | GWPS | AX-94679462 | 3A | 61219767 | 0.000236248 | 0.076241135 | 282 | 3.626631286 |
| IR_DL_2020 | GWPS | AX-94683196 | 4B | 4330618 | 0.000319374 | 0.216312057 | 282 | 3.495700533 |
| IR_DL_2020 | GWPS | AX-94578679 | 3A | 61446196 | 0.000324054 | 0.078014184 | 282 | 3.489382661 |
| IR_DL_2020 | GWPS | AX-94556555 | 2B | 728397561 | 0.000325425 | 0.129432624 | 282 | 3.487549474 |
| IR_DL_2020 | GWPS | AX-94732643 | 4A | 599613464 | 0.000370519 | 0.244680851 | 282 | 3.431189586 |
| IR_DL_2020 | GWPS | AX-94428598 | 4D | 4247242 | 0.000376637 | 0.19858156 | 282 | 3.424076812 |
| IR_DL_2020 | GWPS | AX-94416965 | 2D | 102872670 | 0.000407834 | 0.294326241 | 282 | 3.389516984 |
| IR_DL_2020 | GWPS | AX-94623317 | 4B | 527009135 | 0.000439186 | 0.331560284 | 282 | 3.357351545 |
| IR_DL_2020 | GWPS | AX-94910126 | 2B | 150141335 | 0.000479119 | 0.283687943 | 282 | 3.319556277 |
| IR_DL_2020 | GWPS | AX-94465976 | 3D | 138194078 | 0.00051469 | 0.090425532 | 282 | 3.288453948 |
| IR_DL_2020 | GWPS | AX-94855957 | 5B | 600502639 | 0.000583752 | 0.09929078 | 282 | 3.233771691 |
| IR_DL_2020 | GWPS | AX-94584377 | 4A | 625372103 | 0.000601977 | 0.319148936 | 282 | 3.220419928 |
| IR_DL_2020 | GWPS | AX-94701497 | 3A | 61219665 | 0.000831851 | 0.074468085 | 282 | 3.079954341 |
| LS_DL_2020 | GWPS | AX-94389559 | 5A | 17529329 | 0.000345541 | 0.196808511 | 282 | 3.461499957 |
| LS_DL_2020 | GWPS | AX-95024082 | 1A | 482335926 | 0.000423844 | 0.09751773 | 282 | 3.372793654 |
| LS_DL_2020 | GWPS | AX-94999037 | 5D | 459064274 | 0.000530831 | 0.471631206 | 282 | 3.275043408 |
| LS_DL_2020 | GWPS | AX-94768083 | 5B | 563121382 | 0.000923988 | 0.40248227 | 282 | 3.034333868 |
| LS_DL_2020 | GWPS | AX-94544797 | 1D | 305114395 | 0.000961436 | 0.127659574 | 282 | 3.017079746 |
| RI_DL_2020 | GWPS | AX-94794789 | 7D | 5646692 | 0.00013857 | 0.140070922 | 282 | 3.858331994 |
| RI_DL_2020 | GWPS | AX-94563318 | 5B | 125230671 | 0.000704536 | 0.29787234 | 282 | 3.152096583 |
| RI_DL_2020 | GWPS | AX-94466572 | 6D | 465957530 | 0.000977082 | 0.125886525 | 282 | 3.010068806 |
| IR_IIWBR_2020 | GWPS | AX-95159098 | 6D | 434483212 | 0.000182962 | 0.10106383 | 282 | 3.737639478 |
| IR_IIWBR_2020 | GWPS | AX-95157047 | 7B | 744404624 | 0.000225037 | 0.124113475 | 282 | 3.647746393 |
| IR_IIWBR_2020 | GWPS | AX-94415907 | 5B | 421643627 | 0.000385804 | 0.168439716 | 282 | 3.413633322 |
| IR_IIWBR_2020 | GWPS | AX-95134666 | 4D | 297221718 | 0.000436705 | 0.069148936 | 282 | 3.359812249 |
| IR_IIWBR_2020 | GWPS | AX-94955728 | 5B | 397838009 | 0.000485511 | 0.239361702 | 282 | 3.313801201 |
| IR_IIWBR_2020 | GWPS | AX-94522762 | 5A | 485598127 | 0.000501177 | 0.065602837 | 282 | 3.300008537 |
| IR_IIWBR_2020 | GWPS | AX-94500078 | 5B | 403779997 | 0.00059534 | 0.331560284 | 282 | 3.225235074 |
| IR_IIWBR_2020 | GWPS | AX-94436002 | 5D | 289073944 | 0.000801597 | 0.154255319 | 282 | 3.096043848 |
| IR_IIWBR_2020 | GWPS | AX-95154523 | 5B | 403780173 | 0.000812806 | 0.313829787 | 282 | 3.090012834 |
| IR_IIWBR_2020 | GWPS | AX-95210466 | 6B | 706332789 | 0.000985208 | 0.164893617 | 282 | 3.006472158 |
| RI_JR_2020 | GWPS | AX-94704559 | 3D | 606300512 | 0.000693288 | 0.471631206 | 282 | 3.159086226 |
| RI_JR_2020 | GWPS | AX-94656675 | 5B | 653798260 | 0.000729262 | 0.106382979 | 282 | 3.137116286 |
| IR_PUNE_2020 | GWPS | AX-94660443 | 3B | 727322570 | 0.000406485 | 0.120567376 | 282 | 3.390955887 |
| IR_PUNE_2020 | GWPS | AX-95140494 | 4D | 145507668 | 0.00050033 | 0.115248227 | 282 | 3.300743156 |
| IR_PUNE_2020 | GWPS | AX-94402393 | 3D | 536425356 | 0.000651047 | 0.393617021 | 282 | 3.18638762 |
| IR_PUNE_2020 | GWPS | AX-94685331 | 5B | 480158109 | 0.000733299 | 0.074468085 | 282 | 3.134719165 |
| RI_PUNE_2020 | GWPS | AX-95235626 | 2A | 627513307 | 0.000116851 | 0.328014184 | 282 | 3.932368832 |
| RI_PUNE_2020 | GWPS | AX-95071189 | 5A | 706541345 | 0.000153052 | 0.118794326 | 282 | 3.815161274 |
| RI_PUNE_2020 | GWPS | AX-94676933 | 5A | 482738414 | 0.000634729 | 0.09751773 | 282 | 3.197411365 |
| RI_PUNE_2020 | GWPS | AX-94402388 | 7D | 21081027 | 0.00066123 | 0.166666667 | 282 | 3.179647722 |
| RI_PUNE_2020 | GWPS | AX-94567810 | 5A | 706240306 | 0.000777257 | 0.182624113 | 282 | 3.109435485 |
| RI_PUNE_2020 | GWPS | AX-95020717 | 4A | 662625059 | 0.000793837 | 0.168439716 | 282 | 3.100268566 |
| RI_PUNE_2020 | GWPS | AX-94813910 | 5A | 706541874 | 0.000830265 | 0.122340426 | 282 | 3.08078309 |
| IR_DL_2020 | NDVI | AX-94963273 | 6A | 17534166 | 0.000948782 | 0.484042553 | 282 | 3.022833619 |
| IR_DL_2020 | NDVI | AX-94550967 | 1A | 3381102 | 0.00015476 | 0.113475177 | 282 | 3.810341634 |
| IR_DL_2020 | NDVI | AX-94598030 | 1A | 1159536 | 0.000437939 | 0.113475177 | 282 | 3.358586603 |
| IR_DL_2020 | NDVI | AX-95147357 | 2A | 750565663 | 0.000464658 | 0.195035461 | 282 | 3.332866772 |
| IR_DL_2020 | NDVI | AX-94715499 | 2D | 514658801 | 0.000530741 | 0.326241135 | 282 | 3.275117329 |
| IR_DL_2020 | NDVI | AX-94471623 | 5B | 584069243 | 0.000608662 | 0.108156028 | 282 | 3.215624063 |
| IR_DL_2020 | NDVI | AX-94544614 | 1D | 182039280 | 0.000915431 | 0.108156028 | 282 | 3.038374211 |
| IR_DL_2020 | NDVI | AX-95007159 | 2D | 619598737 | 0.000926482 | 0.418439716 | 282 | 3.033162828 |
| IR_DL_2020 | NDVI | AX-94405391 | 1A | 261373106 | 0.000391217 | 0.430851064 | 282 | 3.407582169 |
| IR_DL_2020 | NDVI | AX-94940145 | 5D | 469501876 | 0.000632615 | 0.234042553 | 282 | 3.198860199 |
| IR_DL_2020 | NDVI | AX-94463626 | 5B | 580840141 | 0.000783735 | 0.086879433 | 282 | 3.105830667 |
| IR_DL_2020 | NDVI | AX-94947326 | 5B | 555179353 | 0.000167492 | 0.20035461 | 282 | 3.776004919 |
| IR_DL_2020 | NDVI | AX-95100010 | 5B | 555943768 | 0.000241475 | 0.205673759 | 282 | 3.617128273 |
| IR_DL_2020 | NDVI | AX-94798973 | 5A | 659457572 | 0.000260403 | 0.166666667 | 282 | 3.584354093 |
| IR_DL_2020 | NDVI | AX-94910126 | 2B | 150141335 | 0.000357784 | 0.283687943 | 282 | 3.446378804 |
| IR_DL_2020 | NDVI | AX-95147357 | 2A | 750565663 | 0.000558491 | 0.195035461 | 282 | 3.252983576 |
| IR_DL_2020 | NDVI | AX-95179939 | 5D | 8546342 | 0.000608335 | 0.129432624 | 282 | 3.215857539 |
| IR_DL_2020 | NDVI | AX-95162278 | 6D | 239048465 | 0.000609827 | 0.093971631 | 282 | 3.214793256 |
| IR_DL_2020 | NDVI | AX-94940145 | 5D | 469501876 | 0.000618929 | 0.234042553 | 282 | 3.208359336 |
| IR_DL_2020 | NDVI | AX-94937975 | 6D | 400075999 | 0.000644431 | 0.384751773 | 282 | 3.190823441 |
| IR_DL_2020 | NDVI | AX-94490702 | 5D | 555528 | 0.000670183 | 0.221631206 | 282 | 3.173806689 |
| IR_DL_2020 | NDVI | AX-94527643 | 2A | 97842216 | 0.000710587 | 0.283687943 | 282 | 3.148383029 |
| IR_DL_2020 | NDVI | AX-95252758 | 6A | 545832250 | 0.000874002 | 0.404255319 | 282 | 3.058487342 |
| IR_DL_2020 | NDVI | AX-94436269 | 2B | 95797357 | 0.000905443 | 0.30141844 | 282 | 3.04313871 |
| IR_DL_2020 | NDVI | AX-94463626 | 5B | 580840141 | 1.90E-08 | 0.086879433 | 282 | 7.720862512 |
| IR_DL_2020 | NDVI | AX-95167887 | 6B | 12675089 | 0.000210114 | 0.054964539 | 282 | 3.677545885 |
| IR_DL_2020 | NDVI | AX-95008467 | 2B | 188719350 | 0.000266406 | 0.058510638 | 282 | 3.574455768 |
| IR_DL_2020 | NDVI | AX-94490240 | 6D | 462536965 | 0.000498317 | 0.090425532 | 282 | 3.302494372 |
| IR_DL_2020 | NDVI | AX-94533409 | 1D | 18576075 | 0.000608327 | 0.475177305 | 282 | 3.215863088 |
| IR_DL_2020 | NDVI | AX-94910126 | 2B | 150141335 | 0.000995188 | 0.283687943 | 282 | 3.002094714 |
| LS_DL_2020 | NDVI | AX-95250945 | 5D | 479583504 | 0.000150375 | 0.20212766 | 282 | 3.822823125 |
| LS_DL_2020 | NDVI | AX-94753708 | 5B | 571231260 | 0.000382861 | 0.054964539 | 282 | 3.416959036 |
| LS_DL_2020 | NDVI | AX-94412376 | 5D | 458907539 | 0.000456882 | 0.058510638 | 282 | 3.340196012 |
| LS_DL_2020 | NDVI | AX-94909292 | 5B | 562226132 | 0.000627778 | 0.062056738 | 282 | 3.202193947 |
| LS_DL_2020 | NDVI | AX-94797710 | 5B | 572591509 | 0.000711435 | 0.092198582 | 282 | 3.147864858 |
| LS_DL_2020 | NDVI | AX-94463626 | 5B | 580840141 | 0.000996181 | 0.086879433 | 282 | 3.001661754 |
| LS_DL_2020 | NDVI | AX-94898331 | 3B | 763067351 | 2.98E-05 | 0.140070922 | 282 | 4.526490229 |
| LS_DL_2020 | NDVI | AX-94765688 | 2B | 752857869 | 6.25E-05 | 0.361702128 | 282 | 4.204399993 |
| LS_DL_2020 | NDVI | AX-94759003 | 2A | 170932926 | 0.000221521 | 0.069148936 | 282 | 3.65458565 |
| LS_DL_2020 | NDVI | AX-94534290 | 2B | 216467652 | 0.000371623 | 0.056737589 | 282 | 3.429897393 |
| LS_DL_2020 | NDVI | AX-94559849 | 2A | 770023173 | 0.00040114 | 0.118794326 | 282 | 3.396703497 |
| LS_DL_2020 | NDVI | AX-94643272 | 3A | 8866518 | 0.000953036 | 0.092198582 | 282 | 3.020890906 |
| LS_DL_2020 | NDVI | AX-94552601 | 4B | 666571858 | 3.51E-08 | 0.113475177 | 282 | 7.45412147 |
| LS_DL_2020 | NDVI | AX-95099367 | 5D | 413073404 | 0.000130769 | 0.070921986 | 282 | 3.883493797 |
| LS_DL_2020 | NDVI | AX-95247247 | 2D | 106321038 | 0.000696423 | 0.09751773 | 282 | 3.157126841 |
| LS_DL_2020 | NDVI | AX-94426211 | 5B | 457128036 | 0.000701115 | 0.328014184 | 282 | 3.154210942 |
| LS_DL_2020 | NDVI | AX-94516158 | 5B | 584886583 | 0.000739678 | 0.239361702 | 282 | 3.130957031 |
| LS_DL_2020 | NDVI | AX-94413923 | 5B | 565928094 | 0.000811296 | 0.118794326 | 282 | 3.090820586 |
| LS_DL_2020 | NDVI | AX-94397362 | 4B | 3420065 | 0.000884958 | 0.069148936 | 282 | 3.053077128 |
| LS_DL_2020 | NDVI | AX-94400346 | 4B | 3420028 | 0.000936462 | 0.074468085 | 282 | 3.028509652 |
| RI_DL_2020 | NDVI | AX-94560091 | 2B | 661450110 | 1.64E-08 | 0.278368794 | 282 | 7.784441488 |
| RI_DL_2020 | NDVI | AX-94658750 | 4A | 27673539 | 3.91E-07 | 0.081560284 | 282 | 6.407471175 |
| RI_DL_2020 | NDVI | AX-94794721 | 5B | 711947092 | 0.000553227 | 0.115248227 | 282 | 3.257096483 |
| RI_DL_2020 | NDVI | AX-94890680 | 5B | 712477386 | 0.000566306 | 0.124113475 | 282 | 3.246948894 |
| RI_DL_2020 | NDVI | AX-95239997 | 1A | 9642399 | 0.000591728 | 0.446808511 | 282 | 3.227877543 |
| RI_DL_2020 | NDVI | AX-94393930 | 5B | 712600724 | 0.000939013 | 0.122340426 | 282 | 3.027328402 |
| RI_DL_2020 | NDVI | AX-95117900 | 3B | 9441866 | 0.000833215 | 0.131205674 | 282 | 3.07924308 |
| RI_DL_2020 | NDVI | AX-94552601 | 4B | 666571858 | 0.000942596 | 0.113475177 | 282 | 3.025674598 |
| RI_DL_2020 | NDVI | AX-94735229 | 7A | 691536158 | 0.000372464 | 0.24822695 | 282 | 3.428915849 |
| RI_DL_2020 | NDVI | AX-94444520 | 5D | 349866325 | 0.000776624 | 0.262411348 | 282 | 3.109789453 |
| RI_DL_2020 | NDVI | AX-94735072 | 2D | 338677300 | 3.35E-07 | 0.460992908 | 282 | 6.474962897 |
| RI_DL_2020 | NDVI | AX-94645515 | 6B | 231676214 | 0.000338044 | 0.093971631 | 282 | 3.471026753 |
| RI_DL_2020 | NDVI | AX-94682249 | 6D | 95723478 | 0.000391252 | 0.102836879 | 282 | 3.407543713 |
| RI_DL_2020 | NDVI | AX-94540968 | 6B | 201433807 | 0.000560605 | 0.095744681 | 282 | 3.251343377 |
| RI_DL_2020 | NDVI | AX-95016300 | 6A | 177265961 | 0.000624891 | 0.090425532 | 282 | 3.204196059 |
| RI_DL_2020 | NDVI | AX-94424491 | 6B | 231692845 | 0.00085945 | 0.058510638 | 282 | 3.065779444 |
| IR_IIWBR_2020 | NDVI | AX-95228815 | 4A | 710665276 | 0.000214228 | 0.377659574 | 282 | 3.669124247 |
| IR_IIWBR_2020 | NDVI | AX-94821409 | 6B | 715750511 | 0.000825367 | 0.127659574 | 282 | 3.083353132 |
| IR_PUNE_2020 | NDVI | AX-94543727 | 3D | 595071692 | 0.000256884 | 0.104609929 | 282 | 3.590263441 |
| IR_PUNE_2020 | NDVI | AX-94623806 | 1B | 600603605 | 0.000326914 | 0.131205674 | 282 | 3.485566372 |
| IR_PUNE_2020 | NDVI | AX-94991264 | 3B | 798506931 | 0.00075154 | 0.111702128 | 282 | 3.124048087 |
| IR_PUNE_2020 | NDVI | AX-94450105 | 7A | 110549821 | 0.000932662 | 0.475177305 | 282 | 3.030275525 |
| IR_PUNE_2020 | NDVI | AX-95075429 | 1B | 426072599 | 0.000959778 | 0.060283688 | 282 | 3.017829095 |
| IR_PUNE_2020 | NDVI | AX-94790358 | 1D | 11409748 | 0.00043196 | 0.095744681 | 282 | 3.364556381 |
| IR_PUNE_2020 | NDVI | AX-94762621 | 2A | 2485186 | 0.000593786 | 0.180851064 | 282 | 3.226370097 |
| IR_PUNE_2020 | NDVI | AX-94756282 | 3D | 1255495 | 0.000786654 | 0.129432624 | 282 | 3.104216006 |
| IR_PUNE_2020 | NDVI | AX-94943657 | 2A | 686172298 | 0.00079682 | 0.124113475 | 282 | 3.098639929 |
| IR_PUNE_2020 | NDVI | AX-94823405 | 2B | 648482734 | 0.000801324 | 0.132978723 | 282 | 3.096191928 |
| IR_PUNE_2020 | NDVI | AX-94454033 | 3A | 107738831 | 0.000914573 | 0.063829787 | 282 | 3.038781393 |
| IR_PUNE_2020 | NDVI | AX-94401833 | 6B | 92569328 | 0.000944739 | 0.281914894 | 282 | 3.024688381 |
| RI_PUNE_2020 | NDVI | AX-94912785 | 7A | 173265797 | 0.000198553 | 0.076241135 | 282 | 3.702123676 |
| RI_PUNE_2020 | NDVI | AX-94721306 | 2A | 724620431 | 0.00028251 | 0.063829787 | 282 | 3.548965862 |
| RI_PUNE_2020 | NDVI | AX-95008466 | 7A | 691535965 | 0.000397231 | 0.095744681 | 282 | 3.400956582 |
| RI_PUNE_2020 | NDVI | AX-94759710 | 3D | 23060884 | 0.00068595 | 0.437943262 | 282 | 3.163707266 |
| RI_PUNE_2020 | NDVI | AX-94681852 | 3A | 584749396 | 0.000755983 | 0.092198582 | 282 | 3.121487847 |
| RI_PUNE_2020 | NDVI | AX-95129652 | 2A | 724375215 | 0.000808324 | 0.074468085 | 282 | 3.092414464 |
| RI_PUNE_2020 | NDVI | AX-95217872 | 2D | 591022287 | 0.000814799 | 0.124113475 | 282 | 3.088949329 |
| RI_PUNE_2020 | NDVI | AX-94535106 | 3B | 582466601 | 0.000830037 | 0.219858156 | 282 | 3.08090277 |
| RI_PUNE_2020 | NDVI | AX-94900690 | 7B | 65303761 | 0.000913055 | 0.054964539 | 282 | 3.039502882 |
| RI_PUNE_2020 | NDVI | AX-94390591 | 5D | 439664240 | 5.03E-05 | 0.154255319 | 282 | 4.298373035 |
| RI_PUNE_2020 | NDVI | AX-95134406 | 2D | 62551264 | 0.000130501 | 0.375886525 | 282 | 3.884386576 |
| RI_PUNE_2020 | NDVI | AX-95126291 | 5A | 581108961 | 0.000328566 | 0.095744681 | 282 | 3.483377848 |
| RI_PUNE_2020 | NDVI | AX-94637995 | 2D | 62401677 | 0.000355187 | 0.459219858 | 282 | 3.449543034 |
| RI_PUNE_2020 | NDVI | AX-94412117 | 7A | 32626994 | 0.000422962 | 0.058510638 | 282 | 3.373698378 |
| RI_PUNE_2020 | NDVI | AX-94736922 | 6D | 72013688 | 0.000553606 | 0.177304965 | 282 | 3.25679886 |
| RI_PUNE_2020 | NDVI | AX-94959258 | 7A | 625743029 | 0.000669417 | 0.34929078 | 282 | 3.174303316 |
| RI_PUNE_2020 | NDVI | AX-94740353 | 7A | 49367757 | 0.000684964 | 0.205673759 | 282 | 3.164332437 |
| RI_PUNE_2020 | NDVI | AX-94410486 | 5A | 582961427 | 0.000879267 | 0.083333333 | 282 | 3.055879464 |
| RI_PUNE_2020 | NDVI | AX-94936257 | 5D | 464068608 | 0.000945464 | 0.095744681 | 282 | 3.024355032 |
| RI_PUNE_2020 | NDVI | AX-94571183 | 7D | 214351136 | 0.000947597 | 0.476950355 | 282 | 3.023376484 |
| RI_PUNE_2020 | NDVI | AX-94405090 | 2A | 709103741 | 0.000973787 | 0.092198582 | 282 | 3.011535854 |
| IR_DL_2019 | NDVI 1 | AX-94720192 | 3B | 417493042 | 1.69E-09 | 0.145390071 | 282 | 8.771123842 |
| IR_DL_2019 | NDVI 1 | AX-94425305 | 2A | 62271435 | 4.79E-08 | 0.067375887 | 282 | 7.319922508 |
| IR_DL_2019 | NDVI 1 | AX-94659413 | 2B | 14049448 | 4.53E-06 | 0.196808511 | 282 | 5.343982803 |
| IR_DL_2019 | NDVI 1 | AX-94519254 | 1A | 575614144 | 6.06E-05 | 0.058510638 | 282 | 4.2174806 |
| IR_DL_2019 | NDVI 1 | AX-94510777 | 4A | 736519193 | 0.000129416 | 0.085106383 | 282 | 3.888011767 |
| IR_DL_2019 | NDVI 1 | AX-95011117 | 2B | 416872893 | 0.000234422 | 0.145390071 | 282 | 3.630001752 |
| IR_DL_2019 | NDVI 1 | AX-94686966 | 5A | 624739660 | 0.000617987 | 0.40070922 | 282 | 3.209020839 |
| IR_DL_2019 | NDVI 1 | AX-94889222 | 6A | 146742347 | 0.000673336 | 0.083333333 | 282 | 3.171768341 |
| IR_DL_2019 | NDVI 1 | AX-95021774 | 3D | 569783736 | 0.000706378 | 0.484042553 | 282 | 3.150962727 |
| IR_DL_2019 | NDVI 1 | AX-94411411 | 3B | 430936438 | 0.000752486 | 0.122340426 | 282 | 3.123501419 |
| LS_DL_2019 | NDVI 1 | AX-94762983 | 1D | 170224720 | 1.57E-11 | 0.20035461 | 282 | 10.80411346 |
| LS_DL_2019 | NDVI 1 | AX-95110974 | 1A | 463290351 | 2.06E-08 | 0.079787234 | 282 | 7.685398294 |
| LS_DL_2019 | NDVI 1 | AX-95155574 | 5B | 692564991 | 1.31E-06 | 0.145390071 | 282 | 5.883923925 |
| LS_DL_2019 | NDVI 1 | AX-94556975 | 1D | 110009099 | 9.76E-06 | 0.19858156 | 282 | 5.010654429 |
| LS_DL_2019 | NDVI 1 | AX-95196898 | 5B | 17979638 | 2.10E-05 | 0.090425532 | 282 | 4.677534951 |
| LS_DL_2019 | NDVI 1 | AX-95105199 | 1D | 241227312 | 0.000177883 | 0.113475177 | 282 | 3.749865701 |
| LS_DL_2019 | NDVI 1 | AX-94775416 | 1D | 240588877 | 0.000182436 | 0.092198582 | 282 | 3.738889388 |
| LS_DL_2019 | NDVI 1 | AX-94426451 | 2B | 447361842 | 0.000191004 | 0.067375887 | 282 | 3.718956987 |
| LS_DL_2019 | NDVI 1 | AX-95024082 | 1A | 482335926 | 0.000222913 | 0.09751773 | 282 | 3.651865239 |
| LS_DL_2019 | NDVI 1 | AX-94491854 | 3B | 815511495 | 0.000242684 | 0.117021277 | 282 | 3.614959218 |
| LS_DL_2019 | NDVI 1 | AX-95246631 | 4B | 24554733 | 0.000248694 | 0.069148936 | 282 | 3.604335139 |
| LS_DL_2019 | NDVI 1 | AX-94477167 | 7D | 451050673 | 0.000261542 | 0.25 | 282 | 3.582457776 |
| LS_DL_2019 | NDVI 1 | AX-94983068 | 1A | 308813558 | 0.000272857 | 0.079787234 | 282 | 3.564065592 |
| LS_DL_2019 | NDVI 1 | AX-94577064 | 2A | 87892339 | 0.000282409 | 0.067375887 | 282 | 3.54912156 |
| LS_DL_2019 | NDVI 1 | AX-94436556 | 3B | 8243819 | 0.000290302 | 0.352836879 | 282 | 3.537150301 |
| LS_DL_2019 | NDVI 1 | AX-95238138 | 1A | 301782334 | 0.000325483 | 0.092198582 | 282 | 3.487471473 |
| LS_DL_2019 | NDVI 1 | AX-95242713 | 1B | 510980080 | 0.000336414 | 0.109929078 | 282 | 3.473125706 |
| LS_DL_2019 | NDVI 1 | AX-95107580 | 2D | 186963464 | 0.000367947 | 0.076241135 | 282 | 3.434215138 |
| LS_DL_2019 | NDVI 1 | AX-94464122 | 1B | 335973804 | 0.000410402 | 0.109929078 | 282 | 3.386791026 |
| LS_DL_2019 | NDVI 1 | AX-94495092 | 1B | 223799152 | 0.00041184 | 0.062056738 | 282 | 3.385271435 |
| LS_DL_2019 | NDVI 1 | AX-94592612 | 4B | 24554735 | 0.000438775 | 0.074468085 | 282 | 3.357757903 |
| LS_DL_2019 | NDVI 1 | AX-94826839 | 1A | 513624126 | 0.00045176 | 0.15070922 | 282 | 3.345091871 |
| LS_DL_2019 | NDVI 1 | AX-94992565 | 1B | 110607725 | 0.000480919 | 0.113475177 | 282 | 3.317928503 |
| LS_DL_2019 | NDVI 1 | AX-94503076 | 1A | 474192439 | 0.000486885 | 0.115248227 | 282 | 3.312573636 |
| LS_DL_2019 | NDVI 1 | AX-94989774 | 5B | 283024857 | 0.000532294 | 0.069148936 | 282 | 3.273848303 |
| LS_DL_2019 | NDVI 1 | AX-95186944 | 5A | 328543240 | 0.000582908 | 0.115248227 | 282 | 3.234399655 |
| LS_DL_2019 | NDVI 1 | AX-95016647 | 7D | 58504216 | 0.000636183 | 0.39893617 | 282 | 3.196417602 |
| LS_DL_2019 | NDVI 1 | AX-94650901 | 1B | 104771363 | 0.000653696 | 0.062056738 | 282 | 3.184623998 |
| LS_DL_2019 | NDVI 1 | AX-94907052 | 1A | 320224045 | 0.000689393 | 0.111702128 | 282 | 3.161533108 |
| LS_DL_2019 | NDVI 1 | AX-94548980 | 5D | 479620076 | 0.00070699 | 0.109929078 | 282 | 3.150586567 |
| LS_DL_2019 | NDVI 1 | AX-95230140 | 2B | 553624227 | 0.000740145 | 0.095744681 | 282 | 3.130683342 |
| LS_DL_2019 | NDVI 1 | AX-95193898 | 2B | 5663685 | 0.000768368 | 0.076241135 | 282 | 3.114430816 |
| LS_DL_2019 | NDVI 1 | AX-94745564 | 1D | 110497408 | 0.000799314 | 0.060283688 | 282 | 3.097282648 |
| LS_DL_2019 | NDVI 1 | AX-94682476 | 1B | 69906174 | 0.000843603 | 0.108156028 | 282 | 3.073862067 |
| LS_DL_2019 | NDVI 1 | AX-94970016 | 1D | 97538509 | 0.000858227 | 0.136524823 | 282 | 3.066397872 |
| IR_DL_2019 | NDVI 2 | AX-94570573 | 1B | 673743649 | 0.000550667 | 0.118794326 | 282 | 3.259111172 |
| IR_DL_2019 | NDVI 2 | AX-94401688 | 7D | 58258719 | 0.000562637 | 0.39893617 | 282 | 3.249771371 |
| IR_DL_2019 | NDVI 2 | AX-95167887 | 6B | 12675089 | 0.000826329 | 0.054964539 | 282 | 3.082847094 |
| LS_DL_2019 | NDVI 2 | AX-94826520 | 3A | 32252119 | 0.000264389 | 0.180851064 | 282 | 3.577757062 |
| LS_DL_2019 | NDVI 2 | AX-94497226 | 5D | 12990535 | 0.000270112 | 0.106382979 | 282 | 3.56845557 |
| LS_DL_2019 | NDVI 2 | AX-94555026 | 3A | 730178232 | 0.00035016 | 0.274822695 | 282 | 3.455733709 |
| LS_DL_2019 | NDVI 2 | AX-94920868 | 5D | 8033845 | 0.000511541 | 0.170212766 | 282 | 3.291119174 |
| LS_DL_2019 | NDVI 2 | AX-94473027 | 5A | 606397172 | 0.000915778 | 0.45035461 | 282 | 3.038209732 |
| LS_DL_2019 | NDVI 2 | AX-94689704 | 7B | 581795502 | 0.000960447 | 0.228723404 | 282 | 3.017526802 |
| RI_DL_2019 | NDVI 2 | AX-94436269 | 2B | 95797357 | 6.06E-10 | 0.30141844 | 282 | 9.217250124 |
| RI_DL_2019 | NDVI 2 | AX-94569570 | 7B | 152075006 | 0.000160888 | 0.078014184 | 282 | 3.793476742 |
| RI_DL_2019 | NDVI 2 | AX-95018763 | 7B | 595797466 | 0.00021203 | 0.214539007 | 282 | 3.673602603 |
| RI_DL_2019 | NDVI 2 | AX-94540157 | 2B | 573594138 | 0.000371732 | 0.083333333 | 282 | 3.429770495 |
| RI_DL_2019 | NDVI 2 | AX-95182450 | 4B | 415125817 | 0.000376581 | 0.156028369 | 282 | 3.424141022 |
| RI_DL_2019 | NDVI 2 | AX-94869965 | 7A | 112265416 | 0.000395632 | 0.191489362 | 282 | 3.402708565 |
| RI_DL_2019 | NDVI 2 | AX-94568269 | 2D | 159336297 | 0.000446305 | 0.088652482 | 282 | 3.350367828 |
| RI_DL_2019 | NDVI 2 | AX-94621634 | 2B | 615886779 | 0.000555461 | 0.175531915 | 282 | 3.255346383 |
| RI_DL_2019 | NDVI 2 | AX-95174190 | 6A | 51760625 | 0.000578259 | 0.274822695 | 282 | 3.237877964 |
| RI_DL_2019 | NDVI 2 | AX-94705334 | 7D | 55430382 | 0.000645283 | 0.407801418 | 282 | 3.190249791 |
| RI_DL_2019 | NDVI 2 | AX-95175956 | 2D | 650963421 | 0.000685135 | 0.095744681 | 282 | 3.164223912 |
| RI_DL_2019 | NDVI 2 | AX-94763185 | 1B | 686933092 | 0.000756426 | 0.487588652 | 282 | 3.121233564 |
| RI_DL_2019 | NDVI 2 | AX-94606541 | 4B | 373780768 | 0.000786832 | 0.196808511 | 282 | 3.104118156 |
| RI_DL_2019 | NDVI 2 | AX-94975607 | 4A | 86853368 | 0.00082566 | 0.15248227 | 282 | 3.083198939 |
| RI_DL_2019 | NDVI 2 | AX-94388520 | 3B | 89068518 | 0.000831556 | 0.379432624 | 282 | 3.080108551 |
| RI_DL_2019 | NDVI 2 | AX-94895903 | 3D | 614366842 | 0.00086295 | 0.111702128 | 282 | 3.064014505 |
| RI_DL_2019 | NDVI 2 | AX-94969584 | 4D | 341390351 | 0.00086676 | 0.092198582 | 282 | 3.062101374 |
| RI_DL_2019 | NDVI 2 | AX-94422468 | 7A | 12309453 | 0.000986321 | 0.393617021 | 282 | 3.005981509 |
| RI_DL_2019 | NDVI 2 | AX-95105278 | 2B | 104832888 | 0.000999456 | 0.363475177 | 282 | 3.000236212 |
| IR_DL_2019 | NDVI 3 | AX-94959586 | 6B | 711235419 | 0.000581564 | 0.20035461 | 282 | 3.235402204 |
| IR_DL_2019 | NDVI 3 | AX-95133813 | 5B | 23322511 | 0.000588899 | 0.308510638 | 282 | 3.229958895 |
| IR_DL_2019 | NDVI 3 | AX-94517684 | 1B | 642678709 | 0.00069967 | 0.296099291 | 282 | 3.155106861 |
| IR_DL_2019 | NDVI 3 | AX-94596987 | 2B | 785271449 | 0.000909795 | 0.118794326 | 282 | 3.041056565 |
| LS_DL_2019 | NDVI 3 | AX-94885543 | 7B | 588288588 | 0.000121881 | 0.065602837 | 282 | 3.914062741 |
| LS_DL_2019 | NDVI 3 | AX-94425134 | 7B | 588290106 | 0.000167315 | 0.143617021 | 282 | 3.776463838 |
| LS_DL_2019 | NDVI 3 | AX-94731327 | 7A | 102056851 | 0.000406519 | 0.138297872 | 282 | 3.390919337 |
| LS_DL_2019 | NDVI 3 | AX-94499340 | 3D | 415157877 | 0.000555852 | 0.147163121 | 282 | 3.255040622 |
| LS_DL_2019 | NDVI 3 | AX-94485699 | 7A | 1704477 | 0.000615132 | 0.388297872 | 282 | 3.21103166 |
| LS_DL_2019 | NDVI 3 | AX-94993081 | 7D | 93782941 | 0.000716828 | 0.104609929 | 282 | 3.144585277 |
| LS_DL_2019 | NDVI 3 | AX-94957884 | 3D | 17778458 | 0.000960362 | 0.163120567 | 282 | 3.017565255 |
| LS_DL_2019 | NDVI 4 | AX-94433353 | 5A | 460518493 | 0.000186785 | 0.187943262 | 282 | 3.728658623 |
| LS_DL_2019 | NDVI 4 | AX-94723735 | 5D | 545214915 | 0.000191322 | 0.29787234 | 282 | 3.718236122 |
| LS_DL_2019 | NDVI 4 | AX-94814880 | 2D | 17606841 | 0.000416069 | 0.163120567 | 282 | 3.380834376 |
| LS_DL_2019 | NDVI 4 | AX-94527390 | 5D | 544615079 | 0.000514355 | 0.283687943 | 282 | 3.288737088 |
| LS_DL_2019 | NDVI 4 | AX-95098966 | 2D | 18234286 | 0.000573967 | 0.134751773 | 282 | 3.241113423 |
| LS_DL_2019 | NDVI 4 | AX-94721501 | 4A | 617946108 | 0.000575909 | 0.29787234 | 282 | 3.239646288 |
| LS_DL_2019 | NDVI 4 | AX-94928539 | 5D | 544697840 | 0.000582991 | 0.285460993 | 282 | 3.23433809 |
| LS_DL_2019 | NDVI 4 | AX-94670668 | 6A | 24420235 | 0.000613455 | 0.45035461 | 282 | 3.212217211 |
| LS_DL_2019 | NDVI 4 | AX-94814470 | 2B | 782147488 | 0.000671585 | 0.10106383 | 282 | 3.172898887 |
| LS_DL_2019 | NDVI 4 | AX-94724080 | 5B | 689929224 | 0.000693441 | 0.203900709 | 282 | 3.158990761 |
| LS_DL_2019 | NDVI 4 | AX-95155574 | 5B | 692564991 | 0.000720348 | 0.145390071 | 282 | 3.142457713 |
| LS_DL_2019 | NDVI 4 | AX-94979447 | 4B | 649605746 | 0.000727603 | 0.069148936 | 282 | 3.138105674 |
| LS_DL_2019 | NDVI 4 | AX-95110488 | 2D | 64190278 | 0.000755506 | 0.414893617 | 282 | 3.12176185 |
| LS_DL_2019 | NDVI 4 | AX-94510777 | 4A | 736519193 | 0.000804012 | 0.085106383 | 282 | 3.094737625 |
| LS_DL_2019 | NDVI 4 | AX-94409923 | 5B | 551499591 | 0.000938663 | 0.20212766 | 282 | 3.027490309 |
| LS_DL_2019 | NDVI 5 | AX-94433353 | 5A | 460518493 | 5.61E-10 | 0.187943262 | 282 | 9.251103489 |
| LS_DL_2019 | NDVI 5 | AX-94935560 | 7A | 63389458 | 9.13E-09 | 0.494680851 | 282 | 8.039646274 |
| LS_DL_2019 | NDVI 5 | AX-95214458 | 1B | 4634343 | 8.89E-05 | 0.34751773 | 282 | 4.050955886 |
| LS_DL_2019 | NDVI 5 | AX-94731327 | 7A | 102056851 | 0.000104139 | 0.138297872 | 282 | 3.98238509 |
| LS_DL_2019 | NDVI 5 | AX-94961429 | 7B | 3795391 | 0.000112106 | 0.179078014 | 282 | 3.950369399 |
| LS_DL_2019 | NDVI 5 | AX-94683572 | 2B | 749418797 | 0.000178 | 0.175531915 | 282 | 3.749580733 |
| LS_DL_2019 | NDVI 5 | AX-94979447 | 4B | 649605746 | 0.00034115 | 0.069148936 | 282 | 3.467054912 |
| LS_DL_2019 | NDVI 5 | AX-94635493 | 3B | 68465490 | 0.000407226 | 0.095744681 | 282 | 3.390164823 |
| LS_DL_2019 | NDVI 5 | AX-94457603 | 7A | 159557314 | 0.000420414 | 0.478723404 | 282 | 3.376323294 |
| LS_DL_2019 | NDVI 5 | AX-94386946 | 1D | 474377044 | 0.000509426 | 0.359929078 | 282 | 3.292918537 |
| LS_DL_2019 | NDVI 5 | AX-94765688 | 2B | 752857869 | 0.000675046 | 0.361702128 | 282 | 3.170666754 |
| LS_DL_2019 | NDVI 5 | AX-94637232 | 3B | 68464206 | 0.000712856 | 0.093971631 | 282 | 3.146998289 |
| LS_DL_2019 | NDVI 5 | AX-94520064 | 4D | 131041602 | 0.000730522 | 0.189716312 | 282 | 3.136366489 |
| LS_DL_2019 | NDVI 5 | AX-95070249 | 4B | 545448054 | 0.000740086 | 0.173758865 | 282 | 3.130717726 |
| LS_DL_2019 | NDVI 5 | AX-94777395 | 7D | 54998295 | 0.000745741 | 0.365248227 | 282 | 3.127412125 |
| LS_DL_2019 | NDVI 5 | AX-95235622 | 1D | 314574223 | 0.000839557 | 0.058510638 | 282 | 3.075950009 |
| LS_DL_2019 | NDVI 5 | AX-95200970 | 7B | 48457248 | 0.000864878 | 0.170212766 | 282 | 3.063044933 |
| LS_DL_2019 | NDVI 5 | AX-94555122 | 3B | 141816638 | 0.000908464 | 0.085106383 | 282 | 3.041692039 |
| LS_DL_2019 | NDVI 5 | AX-94957845 | 1B | 428069284 | 0.000999952 | 0.056737589 | 282 | 3.000020985 |
| LS_DL_2019 | NDVI 5 | DL.2019.LS.NDVI.6 |  |  |  |  |  |  |
| LS_DL_2019 | NDVI 5 | AX-94433353 | 5A | 460518493 | 6.31E-05 | 0.187943262 | 282 | 4.200015306 |
| LS_DL_2019 | NDVI 5 | AX-94637995 | 2D | 62401677 | 0.000423767 | 0.459219858 | 282 | 3.372872643 |
| LS_DL_2019 | NDVI 5 | AX-94449757 | 7A | 215111202 | 0.000760173 | 0.468085106 | 282 | 3.119087663 |
| LS_DL_2019 | NDVI 5 | AX-94723872 | 7D | 94507780 | 0.000942309 | 0.088652482 | 282 | 3.025806576 |
| LS_DL_2019 | NDVI 5 | AX-94653345 | 5D | 198467821 | 1.26E-05 | 0.49822695 | 282 | 4.898517521 |
| LS_DL_2019 | NDVI 5 | AX-94562843 | 6A | 611713192 | 2.13E-05 | 0.095744681 | 282 | 4.672134229 |
| LS_DL_2019 | NDVI 5 | AX-95205723 | 7A | 85906439 | 2.51E-05 | 0.406028369 | 282 | 4.601124969 |
| LS_DL_2019 | NDVI 5 | AX-94420604 | 6A | 613245745 | 0.000185902 | 0.260638298 | 282 | 3.730716135 |
| LS_DL_2019 | NDVI 5 | AX-94644557 | 2D | 34040 | 0.000229115 | 0.203900709 | 282 | 3.639945745 |
| LS_DL_2019 | NDVI 5 | AX-94488136 | 6B | 711764749 | 0.000299273 | 0.255319149 | 282 | 3.523932598 |
| LS_DL_2019 | NDVI 5 | AX-94868061 | 6A | 46974886 | 0.000349762 | 0.145390071 | 282 | 3.456227767 |
| LS_DL_2019 | NDVI 5 | AX-94905285 | 7A | 85438274 | 0.000376277 | 0.393617021 | 282 | 3.42449186 |
| LS_DL_2019 | NDVI 5 | AX-94653845 | 2D | 36435362 | 0.000400597 | 0.163120567 | 282 | 3.397292513 |
| LS_DL_2019 | NDVI 5 | AX-95124010 | 1D | 37220945 | 0.000440314 | 0.147163121 | 282 | 3.356237161 |
| LS_DL_2019 | NDVI 5 | AX-94533562 | 7A | 85912049 | 0.000446695 | 0.427304965 | 282 | 3.349988585 |
| LS_DL_2019 | NDVI 5 | AX-94733613 | 2B | 768613714 | 0.00061501 | 0.09929078 | 282 | 3.21111777 |
| LS_DL_2019 | NDVI 5 | AX-94663391 | 4B | 667413219 | 0.000656996 | 0.230496454 | 282 | 3.18243699 |
| LS_DL_2019 | NDVI 5 | AX-94882811 | 3D | 6019174 | 0.000779198 | 0.485815603 | 282 | 3.108352427 |
| LS_DL_2019 | NDVI 5 | AX-94423424 | 6A | 612189373 | 0.000884272 | 0.359929078 | 282 | 3.053414362 |
| LS_DL_2019 | NDVI 5 | AX-95215256 | 5A | 538757186 | 0.00092777 | 0.496453901 | 282 | 3.032559761 |
| LS_DL_2019 | NDVI 5 | AX-94762983 | 1D | 170224720 | 0.000982552 | 0.20035461 | 282 | 3.007644643 |
| IR_DL_2019 | PH | AX-94544520 | 5B | 569546008 | 4.53E-05 | 0.271276596 | 282 | 4.344297977 |
| IR_DL_2019 | PH | AX-94904992 | 2D | 650327865 | 0.000153765 | 0.09929078 | 282 | 3.813143378 |
| IR_DL_2019 | PH | AX-94424092 | 1D | 290444762 | 0.00015496 | 0.193262411 | 282 | 3.809781465 |
| IR_DL_2019 | PH | AX-95209088 | 1A | 185145571 | 0.00019368 | 0.182624113 | 282 | 3.712914623 |
| IR_DL_2019 | PH | AX-95069114 | 2D | 645934109 | 0.000381627 | 0.234042553 | 282 | 3.418360425 |
| IR_DL_2019 | PH | AX-94986625 | 7A | 666989702 | 0.000392875 | 0.086879433 | 282 | 3.405745613 |
| IR_DL_2019 | PH | AX-94751829 | 1B | 106680653 | 0.000727035 | 0.079787234 | 282 | 3.138444697 |
| IR_DL_2019 | PH | AX-94821666 | 3A | 51475258 | 0.000923165 | 0.186170213 | 282 | 3.034720448 |
| IR_DL_2019 | PH | AX-94470714 | 3A | 53204808 | 0.00095413 | 0.179078014 | 282 | 3.020392393 |
| IR_DL_2020 | PH | AX-95154523 | 5B | 403780173 | 0.000254851 | 0.313829787 | 282 | 3.593714117 |
| IR_DL_2020 | PH | AX-95259052 | 5A | 466615052 | 0.000338824 | 0.303191489 | 282 | 3.470025911 |
| IR_DL_2020 | PH | AX-94500078 | 5B | 403779997 | 0.000487703 | 0.331560284 | 282 | 3.31184493 |
| IR_DL_2020 | PH | AX-94475718 | 6A | 252818652 | 0.000618891 | 0.109929078 | 282 | 3.208385708 |
| IR_DL_2020 | PH | AX-94456270 | 6B | 581868833 | 0.000733439 | 0.067375887 | 282 | 3.134636271 |
| IR_DL_2020 | PH | AX-94575241 | 6B | 645533269 | 0.000834299 | 0.361702128 | 282 | 3.078678498 |
| IR_DL_2020 | PH | AX-94493694 | 6A | 229787559 | 0.00093891 | 0.117021277 | 282 | 3.027375834 |
| IR_DL_2020 | PH | AX-95211139 | 6A | 399879102 | 0.000994685 | 0.090425532 | 282 | 3.002314395 |
| LS_DL_2020 | PH | AX-94635917 | 7B | 582266823 | 8.18E-06 | 0.310283688 | 282 | 5.087290103 |
| LS_DL_2020 | PH | AX-94861052 | 5B | 462144261 | 1.87E-05 | 0.262411348 | 282 | 4.72851356 |
| LS_DL_2020 | PH | AX-94422189 | 7D | 58644172 | 0.000155641 | 0.308510638 | 282 | 3.807875183 |
| LS_DL_2020 | PH | AX-94943835 | 7A | 63703930 | 0.000220629 | 0.381205674 | 282 | 3.656337956 |
| LS_DL_2020 | PH | AX-94747939 | 7D | 58869341 | 0.00026422 | 0.414893617 | 282 | 3.578034044 |
| LS_DL_2020 | PH | AX-94436996 | 7D | 59873376 | 0.000280251 | 0.281914894 | 282 | 3.552452782 |
| LS_DL_2020 | PH | AX-95084909 | 5D | 447141196 | 0.000337715 | 0.466312057 | 282 | 3.471449649 |
| LS_DL_2020 | PH | AX-94404280 | 2A | 649004889 | 0.000495029 | 0.464539007 | 282 | 3.305369624 |
| LS_DL_2020 | PH | AX-95246731 | 6A | 77895799 | 0.000665026 | 0.125886525 | 282 | 3.177161675 |
| LS_DL_2020 | PH | AX-94465558 | 5D | 366748926 | 0.000837259 | 0.413120567 | 282 | 3.077140084 |
| LS_DL_2020 | PH | AX-94468582 | 7A | 134556149 | 0.00097882 | 0.131205674 | 282 | 3.00929721 |
| RI_DL_2020 | PH | AX-94599469 | 6B | 644431633 | 3.15E-07 | 0.132978723 | 282 | 6.501721678 |
| RI_DL_2020 | PH | AX-94453454 | 7D | 371137330 | 0.000106263 | 0.147163121 | 282 | 3.97361798 |
| RI_DL_2020 | PH | AX-94469556 | 2A | 31085631 | 0.000167289 | 0.189716312 | 282 | 3.776531682 |
| RI_DL_2020 | PH | AX-94497886 | 6B | 423084252 | 0.00017572 | 0.118794326 | 282 | 3.755178686 |
| RI_DL_2020 | PH | AX-94767203 | 3A | 41625512 | 0.000191191 | 0.336879433 | 282 | 3.718531723 |
| RI_DL_2020 | PH | AX-95162559 | 7A | 34032637 | 0.000388165 | 0.292553191 | 282 | 3.410983318 |
| RI_DL_2020 | PH | AX-94574902 | 4A | 744309745 | 0.000424876 | 0.076241135 | 282 | 3.371737461 |
| RI_DL_2020 | PH | AX-94754601 | 6A | 578062274 | 0.000736702 | 0.129432624 | 282 | 3.132708213 |
| RI_DL_2020 | PH | AX-94413608 | 5D | 502880989 | 0.000739158 | 0.209219858 | 282 | 3.131262555 |
| RI_DL_2020 | PH | AX-94646010 | 2B | 589879389 | 0.000812394 | 0.359929078 | 282 | 3.090233342 |
| RI_JR_2020 | PH | AX-94415907 | 5B | 421643627 | 2.25E-07 | 0.168439716 | 282 | 6.648635158 |
| RI_JR_2020 | PH | AX-95004450 | 7B | 121890521 | 0.000319993 | 0.095744681 | 282 | 3.49485922 |
| RI_JR_2020 | PH | AX-95166268 | 2D | 601183623 | 0.000423619 | 0.203900709 | 282 | 3.373024093 |
| RI_JR_2020 | PH | AX-94495566 | 2B | 762502999 | 0.000443525 | 0.437943262 | 282 | 3.353082338 |
| RI_JR_2020 | PH | AX-95183918 | 5D | 372297234 | 0.000642289 | 0.475177305 | 282 | 3.192269239 |
| RI_JR_2020 | PH | AX-94816029 | 5D | 405439684 | 0.000774218 | 0.086879433 | 282 | 3.111136944 |
| IR_DL_2019 | PLTY | AX-94721306 | 2A | 724620431 | 0.000415879 | 0.063829787 | 282 | 3.38103299 |
| IR_DL_2019 | PLTY | AX-94812864 | 3A | 102879907 | 0.000594025 | 0.285460993 | 282 | 3.226195576 |
| IR_DL_2019 | PLTY | AX-95218850 | 1D | 10383022 | 0.000658929 | 0.129432624 | 282 | 3.181161681 |
| IR_DL_2019 | PLTY | AX-94918833 | 2A | 740873736 | 0.000927687 | 0.092198582 | 282 | 3.032598648 |
| LS_DL_2019 | PLTY | AX-94997258 | 1D | 385805134 | 5.22E-10 | 0.078014184 | 282 | 9.282206223 |
| LS_DL_2019 | PLTY | AX-94390275 | 1D | 246649492 | 1.32E-08 | 0.184397163 | 282 | 7.878685495 |
| LS_DL_2019 | PLTY | AX-94916490 | 2B | 214282744 | 4.97E-08 | 0.088652482 | 282 | 7.303397996 |
| LS_DL_2019 | PLTY | AX-95257885 | 2A | 16259626 | 1.86E-07 | 0.113475177 | 282 | 6.731364943 |
| LS_DL_2019 | PLTY | AX-94833043 | 7B | 682886515 | 6.12E-07 | 0.111702128 | 282 | 6.212957363 |
| LS_DL_2019 | PLTY | AX-95252161 | 3D | 7244189 | 6.83E-06 | 0.469858156 | 282 | 5.165606438 |
| LS_DL_2019 | PLTY | AX-95103231 | 2D | 600318281 | 0.000117038 | 0.421985816 | 282 | 3.931673345 |
| LS_DL_2019 | PLTY | AX-94897757 | 6B | 719675688 | 0.000207111 | 0.354609929 | 282 | 3.683796703 |
| LS_DL_2019 | PLTY | AX-94664333 | 6B | 716010011 | 0.000256565 | 0.320921986 | 282 | 3.590803259 |
| LS_DL_2019 | PLTY | AX-94937664 | 3A | 663580260 | 0.000312174 | 0.09929078 | 282 | 3.505603407 |
| LS_DL_2019 | PLTY | AX-94486694 | 6D | 461316254 | 0.000390892 | 0.058510638 | 282 | 3.407942778 |
| LS_DL_2019 | PLTY | AX-95161595 | 5D | 421849463 | 0.000620969 | 0.420212766 | 282 | 3.206929953 |
| LS_DL_2019 | PLTY | AX-95005082 | 2A | 16487399 | 0.000731916 | 0.15070922 | 282 | 3.135538953 |
| LS_DL_2019 | PLTY | AX-94600877 | 3A | 660268768 | 0.000732524 | 0.065602837 | 282 | 3.135178295 |
| LS_DL_2019 | PLTY | AX-95111244 | 5A | 698214002 | 0.000740846 | 0.15248227 | 282 | 3.13027187 |
| LS_DL_2019 | PLTY | AX-95092904 | 3B | 701408224 | 0.000815378 | 0.117021277 | 282 | 3.088641031 |
| IR_DL_2020 | PLTY | AX-95222290 | 7D | 543394160 | 2.36E-05 | 0.226950355 | 282 | 4.627311975 |
| IR_DL_2020 | PLTY | AX-94466450 | 6B | 29849235 | 3.44E-05 | 0.10106383 | 282 | 4.463345398 |
| IR_DL_2020 | PLTY | AX-95068836 | 7D | 543603637 | 0.000137145 | 0.127659574 | 282 | 3.862820595 |
| IR_DL_2020 | PLTY | AX-94530943 | 7B | 587910543 | 0.000157911 | 0.320921986 | 282 | 3.801588133 |
| IR_DL_2020 | PLTY | AX-94959258 | 7A | 625743029 | 0.000194988 | 0.34929078 | 282 | 3.709991406 |
| IR_DL_2020 | PLTY | AX-94653468 | 7D | 541242166 | 0.000247817 | 0.117021277 | 282 | 3.605868991 |
| IR_DL_2020 | PLTY | AX-95021378 | 4A | 622188320 | 0.000271331 | 0.157801418 | 282 | 3.566499919 |
| IR_DL_2020 | PLTY | AX-94451613 | 6B | 694679336 | 0.00030175 | 0.345744681 | 282 | 3.520352299 |
| IR_DL_2020 | PLTY | AX-94548288 | 7A | 624688056 | 0.000330622 | 0.242907801 | 282 | 3.4806685 |
| IR_DL_2020 | PLTY | AX-94689704 | 7B | 581795502 | 0.000421298 | 0.228723404 | 282 | 3.375410165 |
| IR_DL_2020 | PLTY | AX-94428406 | 5B | 491583061 | 0.000483734 | 0.090425532 | 282 | 3.315393378 |
| IR_DL_2020 | PLTY | AX-94510387 | 7B | 584806254 | 0.000624666 | 0.161347518 | 282 | 3.204351927 |
| IR_DL_2020 | PLTY | AX-94681771 | 7B | 623975607 | 0.000650067 | 0.237588652 | 282 | 3.187041571 |
| IR_DL_2020 | PLTY | AX-95147208 | 1D | 30777836 | 0.000667056 | 0.189716312 | 282 | 3.175837625 |
| IR_DL_2020 | PLTY | AX-94599608 | 6B | 30011466 | 0.000922657 | 0.161347518 | 282 | 3.034959823 |
| IR_DL_2020 | PLTY | AX-94437579 | 7D | 540714499 | 0.000991726 | 0.136524823 | 282 | 3.003608162 |
| RI_DL_2020 | PLTY | AX-94500245 | 3D | 4968780 | 0.000866628 | 0.489361702 | 282 | 3.062167464 |
| RI_DL_2020 | PLTY | AX-94681852 | 3A | 584749396 | 0.000877658 | 0.092198582 | 282 | 3.056674827 |
| IR_IIWBR_2020 | PLTY | AX-94959965 | 3A | 727990434 | 0.000369488 | 0.088652482 | 282 | 3.432399572 |
| RI_IND_2020 | PLTY | AX-94874038 | 5A | 611560369 | 0.000115435 | 0.060283688 | 282 | 3.937664169 |
| RI_IND_2020 | PLTY | AX-94747224 | 5A | 613477789 | 0.000251043 | 0.062056738 | 282 | 3.600251722 |
| RI_IND_2020 | PLTY | AX-95251638 | 4B | 654592653 | 0.00027166 | 0.129432624 | 282 | 3.565973561 |
| RI_IND_2020 | PLTY | AX-95003465 | 1D | 342070118 | 0.000314201 | 0.078014184 | 282 | 3.502792148 |
| RI_IND_2020 | PLTY | AX-94468582 | 7A | 134556149 | 0.000651542 | 0.131205674 | 282 | 3.18605761 |
| RI_IND_2020 | PLTY | AX-94570560 | 4B | 619224779 | 0.000747109 | 0.228723404 | 282 | 3.126615998 |
| RI_IND_2020 | PLTY | AX-95222044 | 4B | 598261950 | 0.000977921 | 0.09751773 | 282 | 3.009696345 |
| RI_JR_2020 | PLTY | AX-94460140 | 1B | 565336836 | 0.00025948 | 0.446808511 | 282 | 3.585896303 |
| RI_JR_2020 | PLTY | AX-94635180 | 4B | 90504836 | 0.000535837 | 0.09751773 | 282 | 3.270967186 |
| RI_JR_2020 | PLTY | AX-94969778 | 1B | 531167036 | 0.000592335 | 0.246453901 | 282 | 3.227432489 |
| RI_JR_2020 | PLTY | AX-94882125 | 3B | 94320948 | 0.000649474 | 0.062056738 | 282 | 3.187438354 |
| RI_JR_2020 | PLTY | AX-94830773 | 4B | 95374239 | 0.000700375 | 0.070921986 | 282 | 3.154669476 |
| RI_JR_2020 | PLTY | AX-94941069 | 5B | 546830055 | 0.000812656 | 0.290780142 | 282 | 3.090093276 |
| RI_JR_2020 | PLTY | AX-95169563 | 7D | 62280926 | 0.000907631 | 0.143617021 | 282 | 3.042090896 |
| RI_JR_2020 | PLTY | AX-94847486 | 7A | 65975884 | 0.00099056 | 0.109929078 | 282 | 3.00411914 |
| RI_JR_2020 | PLTY | AX-94397040 | 4B | 414799611 | 0.00099626 | 0.086879433 | 282 | 3.001627095 |
| RI_PUNE_2020 | PLTY | AX-94391038 | 3B | 232605210 | 5.24E-05 | 0.138297872 | 282 | 4.280355373 |
| RI_PUNE_2020 | PLTY | AX-95173587 | 2B | 12076610 | 0.000109046 | 0.294326241 | 282 | 3.962389682 |
| RI_PUNE_2020 | PLTY | AX-95220232 | 7D | 64891768 | 0.00015968 | 0.111702128 | 282 | 3.796748738 |
| RI_PUNE_2020 | PLTY | AX-94640059 | 7A | 693816164 | 0.000179623 | 0.10106383 | 282 | 3.745637811 |
| RI_PUNE_2020 | PLTY | AX-94923418 | 7B | 197701844 | 0.000469823 | 0.164893617 | 282 | 3.328066154 |
| RI_PUNE_2020 | PLTY | AX-94502699 | 2D | 56203334 | 0.000841509 | 0.186170213 | 282 | 3.074941017 |
| RI_PUNE_2020 | PLTY | AX-94449146 | 1B | 606189561 | 0.00087598 | 0.113475177 | 282 | 3.057505792 |
| RI_PUNE_2020 | PLTY | AX-94676714 | 5A | 675958158 | 0.000899919 | 0.175531915 | 282 | 3.04579663 |
| RI_PUNE_2020 | PLTY | AX-95115419 | 5B | 684042182 | 0.000919977 | 0.14893617 | 282 | 3.036223217 |
| IR_DL_2020 | SPAD | AX-95192108 | 1A | 510373143 | 0.000182635 | 0.124113475 | 282 | 3.738414907 |
| IR_DL_2020 | SPAD | AX-94536777 | 3A | 690193735 | 0.00042569 | 0.154255319 | 282 | 3.370906191 |
| IR_DL_2020 | SPAD | AX-95194962 | 6D | 16235670 | 0.000476178 | 0.134751773 | 282 | 3.322231077 |
| IR_DL_2020 | SPAD | AX-95257633 | 4B | 38282744 | 0.000484286 | 0.379432624 | 282 | 3.314898047 |
| IR_DL_2020 | SPAD | AX-94399803 | 5D | 545943255 | 0.000829437 | 0.180851064 | 282 | 3.081216623 |
| LS_DL_2020 | SPAD | AX-94548900 | 6B | 340304616 | 0.000621261 | 0.294326241 | 282 | 3.206726049 |
| RI_DL_2020 | SPAD | AX-94777981 | 6B | 712324036 | 6.63E-05 | 0.184397163 | 282 | 4.178614631 |
| RI_DL_2020 | SPAD | AX-94749511 | 6B | 707244736 | 7.47E-05 | 0.175531915 | 282 | 4.126899013 |
| RI_DL_2020 | SPAD | AX-94451862 | 6B | 710149895 | 0.000183277 | 0.25177305 | 282 | 3.736891722 |
| RI_DL_2020 | SPAD | AX-95173340 | 6B | 711235327 | 0.000215177 | 0.074468085 | 282 | 3.667204942 |
| RI_DL_2020 | SPAD | AX-94423828 | 6B | 710200300 | 0.000305342 | 0.241134752 | 282 | 3.515213305 |
| RI_DL_2020 | SPAD | AX-94466511 | 6D | 463716132 | 0.000474113 | 0.090425532 | 282 | 3.324118414 |
| RI_DL_2020 | SPAD | AX-94804853 | 4A | 718836214 | 0.000665703 | 0.390070922 | 282 | 3.176719569 |
| RI_DL_2020 | SPAD | AX-94451660 | 5A | 679138016 | 0.000719099 | 0.329787234 | 282 | 3.143211156 |
| RI_DL_2020 | SPAD | AX-95148946 | 3A | 575493217 | 0.000840197 | 0.25 | 282 | 3.075618975 |
| LS_DL_2020 | SPAD | AX-94404301 | 4D | 340980889 | 0.000424772 | 0.476950355 | 282 | 3.371844081 |
| LS_DL_2020 | SPAD | AX-95177058 | 5D | 538657406 | 0.000682406 | 0.108156028 | 282 | 3.165957378 |
| LS_DL_2020 | SPAD | AX-95192108 | 1A | 510373143 | 0.000733329 | 0.124113475 | 282 | 3.13470125 |
| LS_DL_2020 | SPAD | AX-94862187 | 6A | 23410200 | 0.000998888 | 0.177304965 | 282 | 3.000483284 |
| IR_DL_2020 | SPKLNTH | AX-94527740 | 2B | 51930364 | 9.16E-06 | 0.285460993 | 282 | 5.038034949 |
| IR_DL_2020 | SPKLNTH | AX-94810283 | 2A | 96248691 | 4.97E-05 | 0.196808511 | 282 | 4.30377911 |
| IR_DL_2020 | SPKLNTH | AX-94417491 | 3B | 127929564 | 0.000186973 | 0.09751773 | 282 | 3.728220835 |
| IR_DL_2020 | SPKLNTH | AX-94753708 | 5B | 571231260 | 0.000429108 | 0.054964539 | 282 | 3.367433365 |
| IR_DL_2020 | SPKLNTH | AX-95207464 | 7B | 37854834 | 0.000518041 | 0.062056738 | 282 | 3.285635886 |
| IR_DL_2020 | SPKLNTH | AX-94923774 | 4A | 733460243 | 0.000638724 | 0.218085106 | 282 | 3.194686724 |
| IR_DL_2020 | SPKLNTH | AX-94677709 | 2B | 786230752 | 0.000664942 | 0.141843972 | 282 | 3.17721649 |
| IR_DL_2020 | SPKLNTH | AX-94465976 | 3D | 138194078 | 0.000782567 | 0.090425532 | 282 | 3.10647827 |
| LS_DL_2020 | SPKLNTH | AX-94474207 | 5D | 488428762 | 2.67E-09 | 0.171985816 | 282 | 8.573871872 |
| LS_DL_2020 | SPKLNTH | AX-94514616 | 7A | 674272470 | 1.85E-07 | 0.434397163 | 282 | 6.732687405 |
| LS_DL_2020 | SPKLNTH | AX-94428141 | 5B | 128854481 | 3.18E-05 | 0.370567376 | 282 | 4.497289906 |
| LS_DL_2020 | SPKLNTH | AX-94780255 | 4B | 672237311 | 5.52E-05 | 0.15070922 | 282 | 4.257939159 |
| LS_DL_2020 | SPKLNTH | AX-95188181 | 1A | 502804981 | 0.00016731 | 0.218085106 | 282 | 3.776477678 |
| LS_DL_2020 | SPKLNTH | AX-94680240 | 6A | 585428558 | 0.000232854 | 0.175531915 | 282 | 3.6329154 |
| LS_DL_2020 | SPKLNTH | AX-94527403 | 3B | 802600552 | 0.000292961 | 0.072695035 | 282 | 3.533190394 |
| LS_DL_2020 | SPKLNTH | AX-94647273 | 3B | 775830708 | 0.000537552 | 0.180851064 | 282 | 3.269579446 |
| LS_DL_2020 | SPKLNTH | AX-95072131 | 2B | 491022596 | 0.000686706 | 0.115248227 | 282 | 3.163229452 |
| LS_DL_2020 | SPKLNTH | AX-94637777 | 6A | 585343407 | 0.000867061 | 0.143617021 | 282 | 3.061950583 |
| LS_DL_2020 | SPKLNTH | AX-94475816 | 3B | 776120883 | 0.000924415 | 0.138297872 | 282 | 3.034133173 |
| LS_DL_2020 | SPKLNTH | AX-94674699 | 2A | 569205887 | 0.000962768 | 0.109929078 | 282 | 3.01647851 |
| LS_DL_2020 | TGW | AX-94385515 | 5B | 10444933 | 0.000110336 | 0.180851064 | 282 | 3.957284676 |
| LS_DL_2020 | TGW | AX-94791055 | 2B | 749138678 | 0.000289208 | 0.324468085 | 282 | 3.53878923 |
| LS_DL_2020 | TGW | AX-94912807 | 6A | 467046581 | 0.000859514 | 0.432624113 | 282 | 3.065746919 |
| RI_DL_2020 | TGW | AX-94579198 | 1D | 409496915 | 0.000772213 | 0.15248227 | 282 | 3.112262952 |
| IR_IIWBR_2020 | TGW | AX-94700391 | 3A | 700422029 | 6.70E-08 | 0.074468085 | 282 | 7.173683629 |
| IR_IIWBR_2020 | TGW | AX-94744025 | 4B | 28069543 | 0.000147737 | 0.127659574 | 282 | 3.830511495 |
| IR_IIWBR_2020 | TGW | AX-94400545 | 4A | 584124176 | 0.000206119 | 0.138297872 | 282 | 3.685881515 |
| IR_IIWBR_2020 | TGW | AX-94400451 | 4A | 584271661 | 0.000314642 | 0.237588652 | 282 | 3.502183707 |
| IR_IIWBR_2020 | TGW | AX-95102079 | 6B | 462036168 | 0.000333112 | 0.14893617 | 282 | 3.477409294 |
| IR_IIWBR_2020 | TGW | AX-95200618 | 6A | 258142746 | 0.000337335 | 0.106382979 | 282 | 3.471938151 |
| IR_IIWBR_2020 | TGW | AX-95204989 | 6A | 435064234 | 0.000378165 | 0.179078014 | 282 | 3.422318655 |
| IR_IIWBR_2020 | TGW | AX-94404431 | 6B | 476415045 | 0.000503087 | 0.157801418 | 282 | 3.298357228 |
| IR_IIWBR_2020 | TGW | AX-94496337 | 3B | 250465776 | 0.000525341 | 0.086879433 | 282 | 3.279558746 |
| IR_IIWBR_2020 | TGW | AX-94424491 | 6B | 231692845 | 0.000533734 | 0.058510638 | 282 | 3.272675533 |
| IR_IIWBR_2020 | TGW | AX-94819074 | 7A | 734539011 | 0.000568835 | 0.092198582 | 282 | 3.245013319 |
| IR_IIWBR_2020 | TGW | AX-94513007 | 6D | 147239252 | 0.000621244 | 0.179078014 | 282 | 3.206737908 |
| IR_IIWBR_2020 | TGW | AX-95183088 | 6B | 232168465 | 0.000654485 | 0.163120567 | 282 | 3.184100304 |
| IR_IIWBR_2020 | TGW | AX-95007288 | 6B | 333171198 | 0.000674946 | 0.156028369 | 282 | 3.170731222 |
| IR_IIWBR_2020 | TGW | AX-94727885 | 5B | 488107976 | 0.000686571 | 0.393617021 | 282 | 3.163314548 |
| IR_IIWBR_2020 | TGW | AX-94508847 | 4A | 583974873 | 0.000744937 | 0.260638298 | 282 | 3.127880248 |
| IR_IIWBR_2020 | TGW | AX-94467818 | 6B | 122679800 | 0.000763773 | 0.157801418 | 282 | 3.117035837 |
| IR_IIWBR_2020 | TGW | AX-95127885 | 6D | 157450680 | 0.000773316 | 0.166666667 | 282 | 3.111643059 |
| IR_IIWBR_2020 | TGW | AX-95101481 | 3D | 170681650 | 0.00078967 | 0.079787234 | 282 | 3.102554443 |
| IR_IIWBR_2020 | TGW | AX-94525446 | 4A | 584349479 | 0.000826736 | 0.244680851 | 282 | 3.082633029 |
| IR_IIWBR_2020 | TGW | AX-94563364 | 4A | 583909391 | 0.000875253 | 0.264184397 | 282 | 3.057866451 |
| IR_IIWBR_2020 | TGW | AX-94407690 | 4A | 583953494 | 0.000876431 | 0.187943262 | 282 | 3.057282362 |
| IR_IIWBR_2020 | TGW | AX-94462774 | 6B | 309782948 | 0.000932342 | 0.132978723 | 282 | 3.030424531 |
| IR_IIWBR_2020 | TGW | AX-94383985 | 3B | 147683153 | 0.00093283 | 0.125886525 | 282 | 3.030197358 |
| IR_IIWBR_2020 | TGW | AX-94806637 | 7B | 401546360 | 0.00099812 | 0.14893617 | 282 | 3.000817102 |
| RI_IND_2020 | TGW | AX-95195224 | 7A | 321653330 | 0.000237576 | 0.358156028 | 282 | 3.624198144 |
| RI_IND_2020 | TGW | AX-94989647 | 6A | 581976194 | 0.00066659 | 0.156028369 | 282 | 3.176141346 |
| RI_IND_2020 | TGW | AX-94692255 | 7B | 586748774 | 0.000695012 | 0.159574468 | 282 | 3.158007411 |
| RI_JR_2020 | TGW | AX-94505686 | 1D | 80445861 | 5.32E-09 | 0.076241135 | 282 | 8.273984384 |
| RI_JR_2020 | TGW | AX-95107567 | 7D | 406896330 | 4.32E-07 | 0.086879433 | 282 | 6.364985553 |
| RI_JR_2020 | TGW | AX-94598030 | 1A | 1159536 | 1.51E-06 | 0.113475177 | 282 | 5.822309408 |
| RI_JR_2020 | TGW | AX-95231147 | 2A | 24301168 | 0.000159317 | 0.331560284 | 282 | 3.797737087 |
| RI_JR_2020 | TGW | AX-95085884 | 2D | 8568937 | 0.000378486 | 0.324468085 | 282 | 3.421949844 |
| RI_JR_2020 | TGW | AX-95159844 | 1A | 534565904 | 0.000472132 | 0.09751773 | 282 | 3.325936435 |
| RI_JR_2020 | TGW | AX-94891248 | 2A | 19125996 | 0.000494799 | 0.343971631 | 282 | 3.305571465 |
| RI_JR_2020 | TGW | AX-94692252 | 2B | 21952451 | 0.000519064 | 0.361702128 | 282 | 3.284779404 |
| RI_JR_2020 | TGW | AX-94585962 | 2B | 25248991 | 0.00052748 | 0.136524823 | 282 | 3.277793728 |
| RI_JR_2020 | TGW | AX-95211013 | 2B | 5670138 | 0.000532035 | 0.329787234 | 282 | 3.274059825 |
| RI_JR_2020 | TGW | AX-94688290 | 2A | 15236993 | 0.000541656 | 0.218085106 | 282 | 3.266276429 |
| RI_JR_2020 | TGW | AX-95005223 | 2A | 11548803 | 0.000631131 | 0.335106383 | 282 | 3.199880235 |
| RI_JR_2020 | TGW | AX-94448314 | 2A | 17659007 | 0.000726325 | 0.179078014 | 282 | 3.138869074 |
| RI_JR_2020 | TGW | AX-95115244 | 2D | 168644036 | 0.000732395 | 0.104609929 | 282 | 3.135254538 |
| RI_JR_2020 | TGW | AX-94690771 | 2A | 4171296 | 0.000912463 | 0.210992908 | 282 | 3.039784938 |
| IR_PUNE_2020 | TGW | AX-94634468 | 2B | 767374289 | 1.52E-07 | 0.343971631 | 282 | 6.817670955 |
| IR_PUNE_2020 | TGW | AX-94433091 | 2D | 279866 | 0.000174138 | 0.274822695 | 282 | 3.759106121 |
| IR_PUNE_2020 | TGW | AX-95073175 | 7D | 55007731 | 0.00049316 | 0.35106383 | 282 | 3.307012343 |
| IR_PUNE_2020 | TGW | AX-95154971 | 2B | 16268725 | 0.000563463 | 0.462765957 | 282 | 3.249134379 |
| IR_PUNE_2020 | TGW | AX-94416982 | 1A | 47578129 | 0.000705103 | 0.459219858 | 282 | 3.151747618 |
| IR_PUNE_2020 | TGW | AX-94509279 | 1B | 670180628 | 0.000791406 | 0.478723404 | 282 | 3.101600653 |
| IR_PUNE_2020 | TGW | AX-95078224 | 1A | 407632565 | 0.000857754 | 0.19858156 | 282 | 3.06663717 |
| IR_PUNE_2020 | TGW | AX-94621372 | 1B | 4478877 | 0.000902646 | 0.124113475 | 282 | 3.044482543 |
| IR_PUNE_2020 | TGW | AX-94638908 | 7B | 721220492 | 0.000913076 | 0.093971631 | 282 | 3.039493034 |
| IR_PUNE_2020 | TGW | AX-94885849 | 7B | 310411816 | 0.000977126 | 0.492907801 | 282 | 3.010049257 |
| RI_PUNE_2020 | TGW | AX-94759710 | 3D | 23060884 | 5.06E-06 | 0.437943262 | 282 | 5.29595929 |
| RI_PUNE_2020 | TGW | AX-94853252 | 3D | 22281272 | 3.61E-05 | 0.416666667 | 282 | 4.442252752 |
| RI_PUNE_2020 | TGW | AX-95179399 | 3A | 32118994 | 7.01E-05 | 0.423758865 | 282 | 4.154164168 |
| RI_PUNE_2020 | TGW | AX-94601768 | 7A | 44447779 | 7.67E-05 | 0.166666667 | 282 | 4.115366822 |
| RI_PUNE_2020 | TGW | AX-95206732 | 3B | 32278221 | 9.83E-05 | 0.484042553 | 282 | 4.007520257 |
| RI_PUNE_2020 | TGW | AX-94548313 | 3D | 19959968 | 0.000178758 | 0.466312057 | 282 | 3.747733411 |
| RI_PUNE_2020 | TGW | AX-94714854 | 7A | 27096999 | 0.000240381 | 0.312056738 | 282 | 3.619100519 |
| RI_PUNE_2020 | TGW | AX-95021295 | 7A | 36859529 | 0.00026157 | 0.381205674 | 282 | 3.582412462 |
| RI_PUNE_2020 | TGW | AX-95229410 | 5D | 475693779 | 0.000452943 | 0.45212766 | 282 | 3.343956593 |
| RI_PUNE_2020 | TGW | AX-94464303 | 7A | 44443938 | 0.000479618 | 0.122340426 | 282 | 3.319104709 |
| RI_PUNE_2020 | TGW | AX-95219561 | 3B | 805339961 | 0.000499856 | 0.120567376 | 282 | 3.301155154 |
| RI_PUNE_2020 | TGW | AX-94752880 | 3B | 23026686 | 0.000517168 | 0.067375887 | 282 | 3.286368223 |
| RI_PUNE_2020 | TGW | AX-94440483 | 1A | 532815403 | 0.000571982 | 0.320921986 | 282 | 3.242617747 |
| RI_PUNE_2020 | TGW | AX-94536530 | 4B | 602781575 | 0.000677931 | 0.377659574 | 282 | 3.168814247 |
| RI_PUNE_2020 | TGW | AX-94561124 | 7B | 498970935 | 0.000821454 | 0.267730496 | 282 | 3.08541674 |
| RI_PUNE_2020 | TGW | AX-94528523 | 5B | 700520244 | 0.0009123 | 0.271276596 | 282 | 3.039862418 |
| RI_PUNE_2020 | TGW | AX-95101284 | 4B | 612253957 | 0.000925607 | 0.283687943 | 282 | 3.033573326 |
