# Supplementary material for: Genome-wide association mapping for component traits of drought and heat tolerance in wheat
Source: Front Plant Sci. 2022 Aug 16;13:943033. doi: 10.3389/fpls.2022.943033 (PMC9429996; doi:10.3389/fpls.2022.943033)

Supplementary Fig 1: Trait wise Frequency distribution of all the studied traits across the environments

GWPS


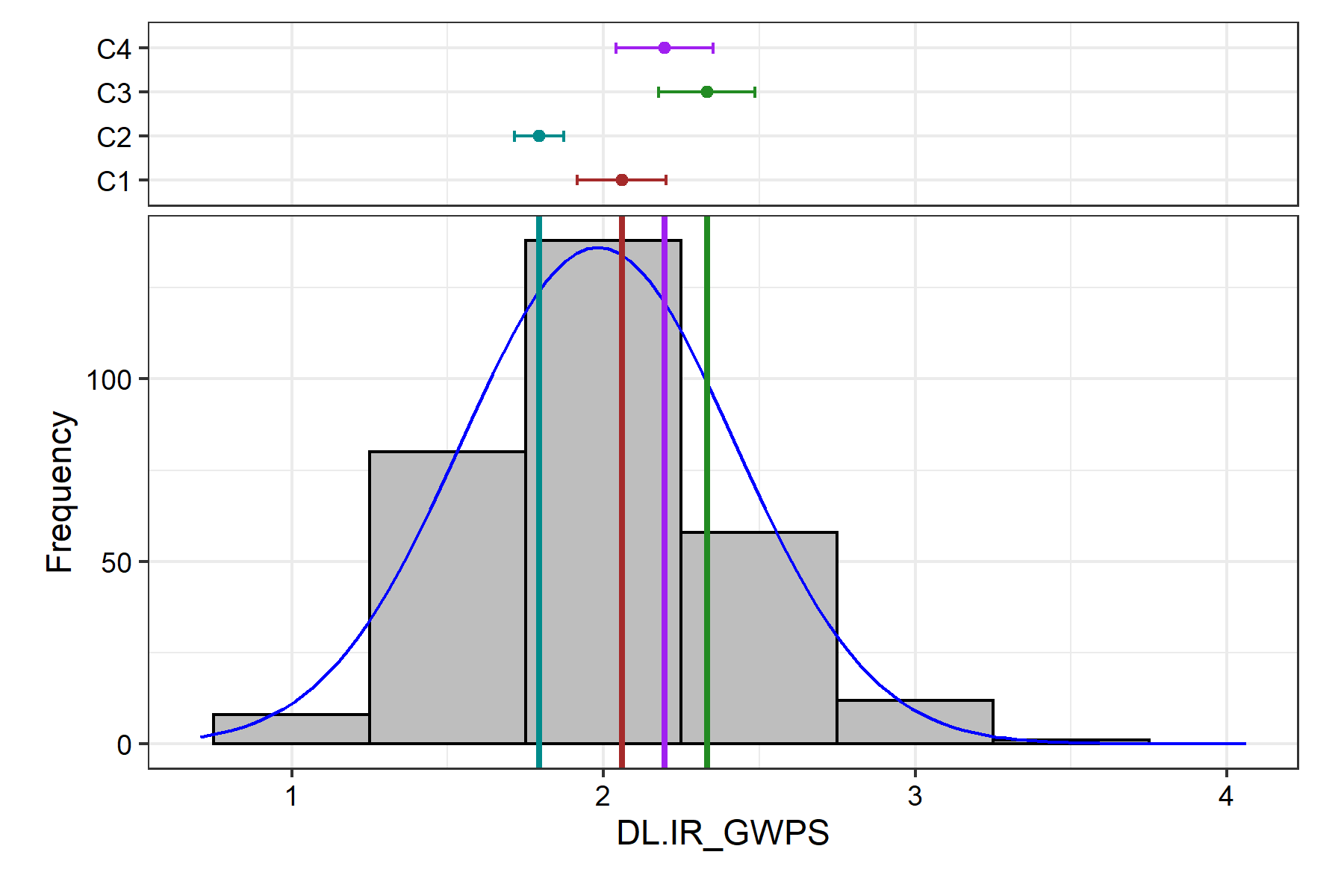

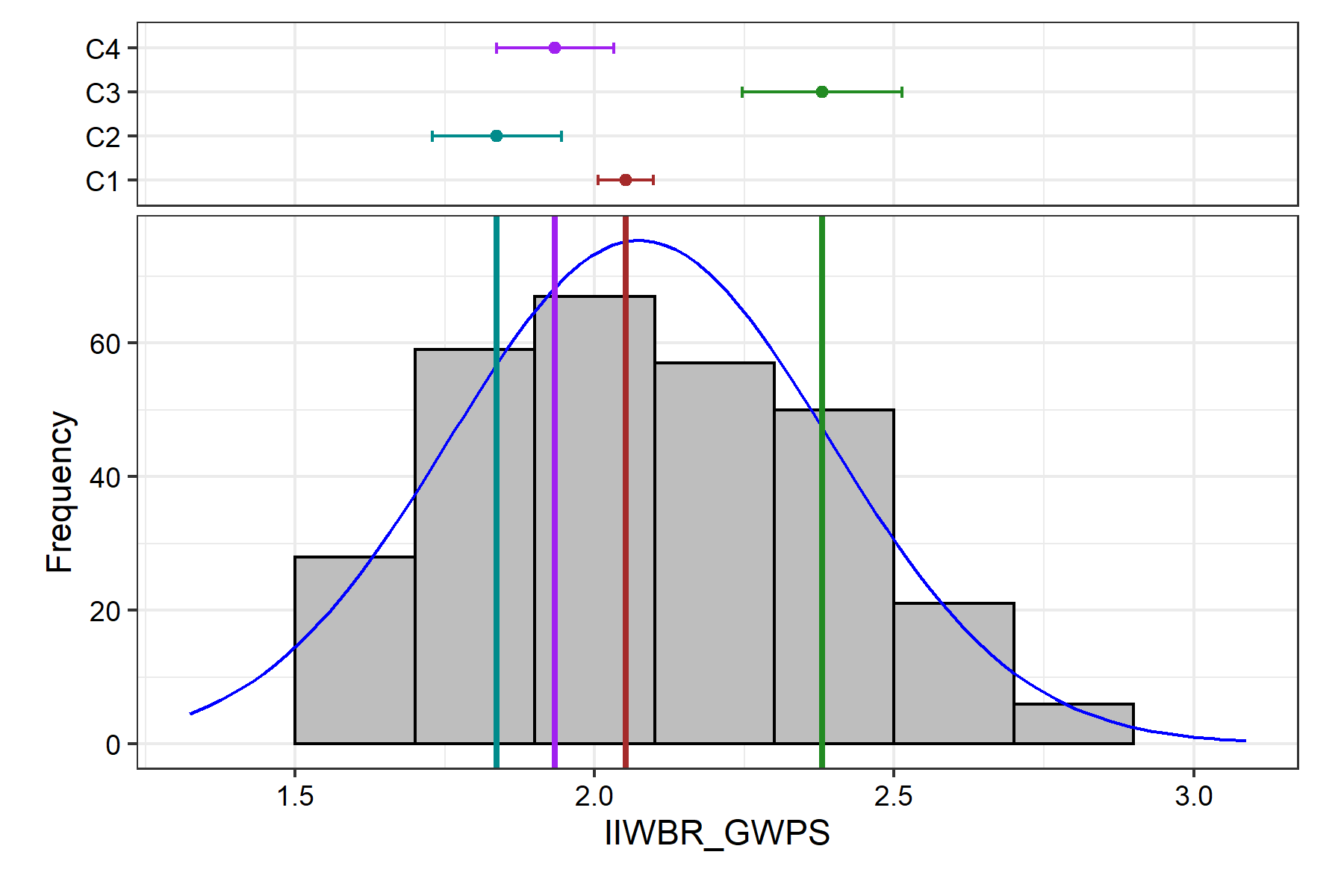

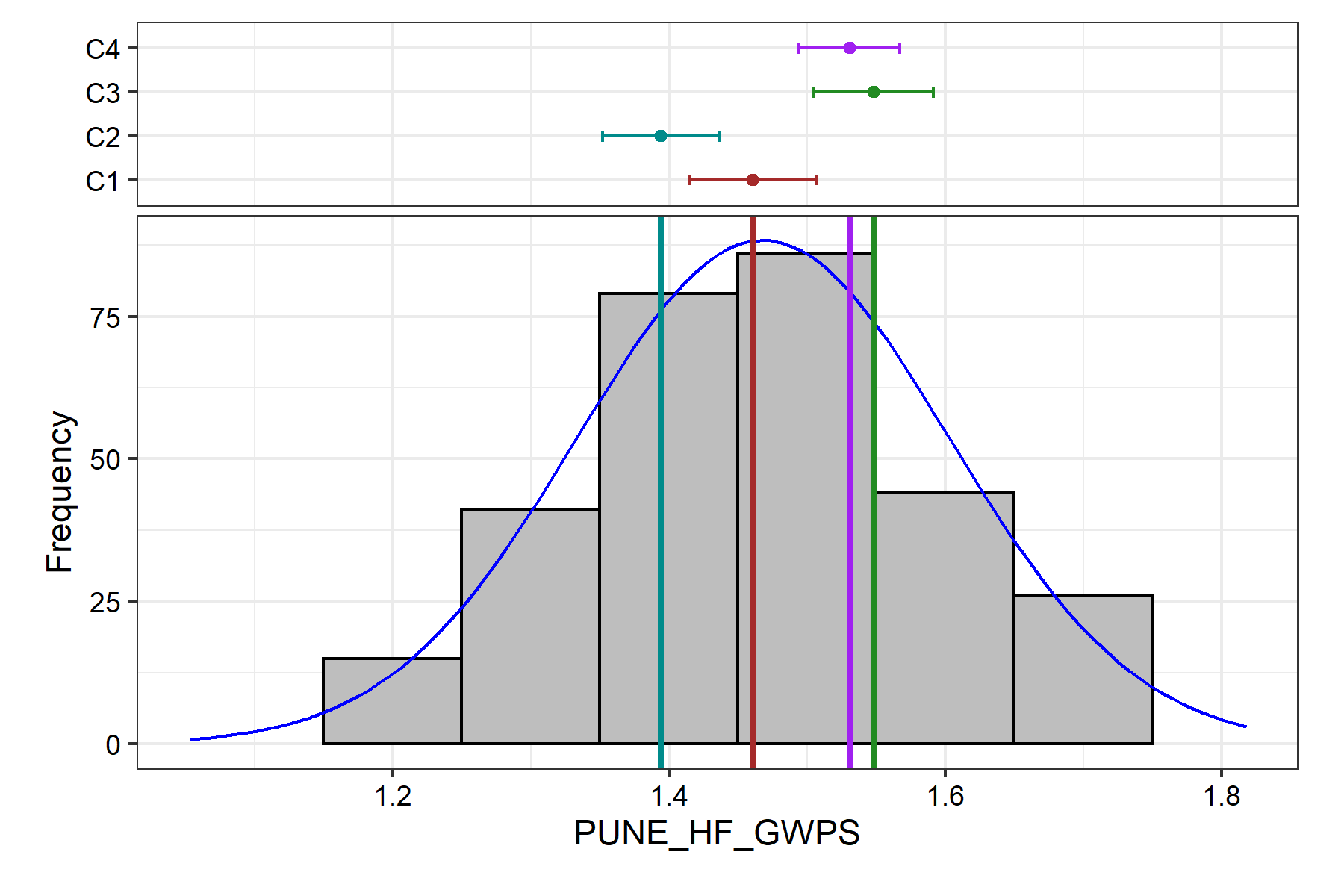

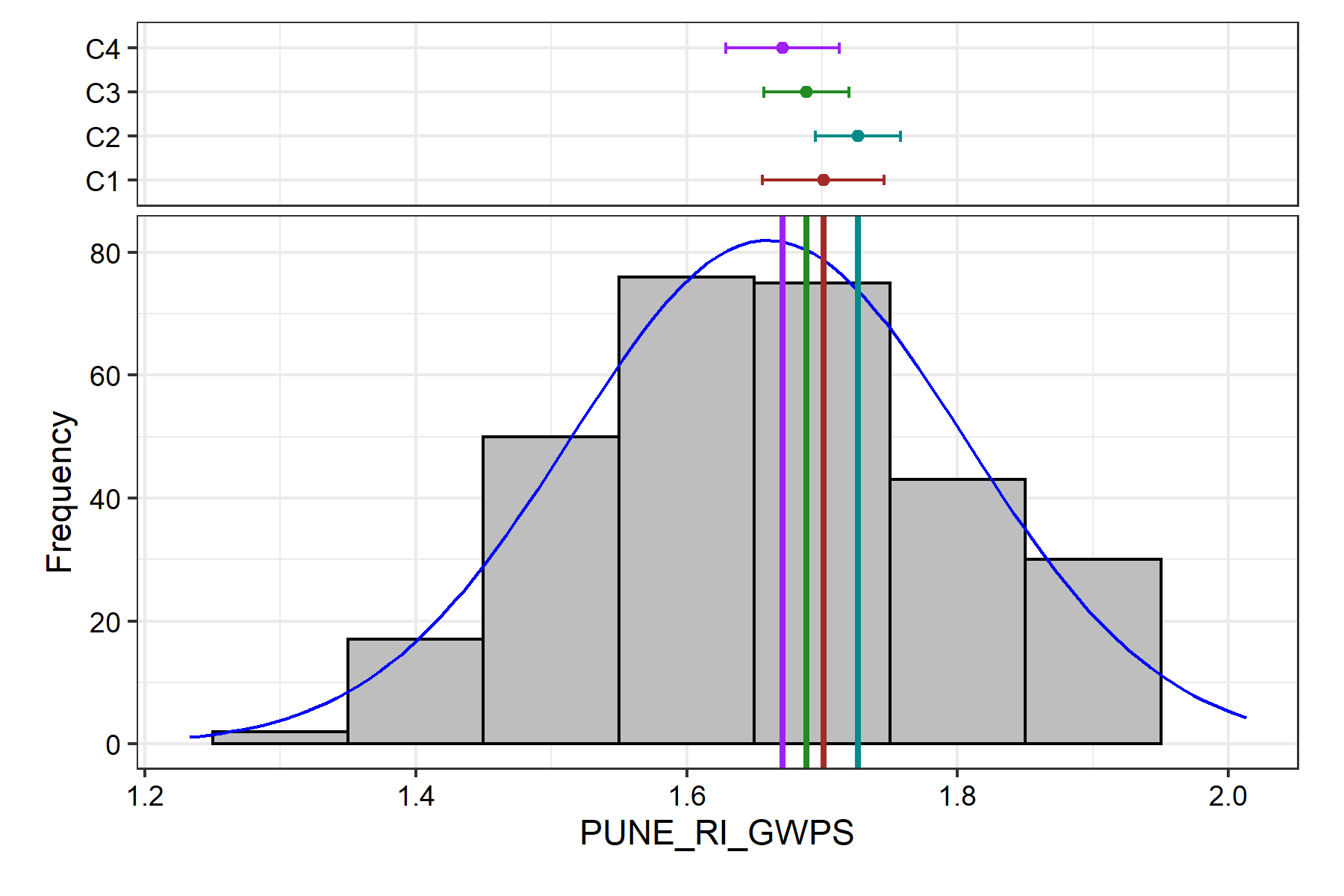

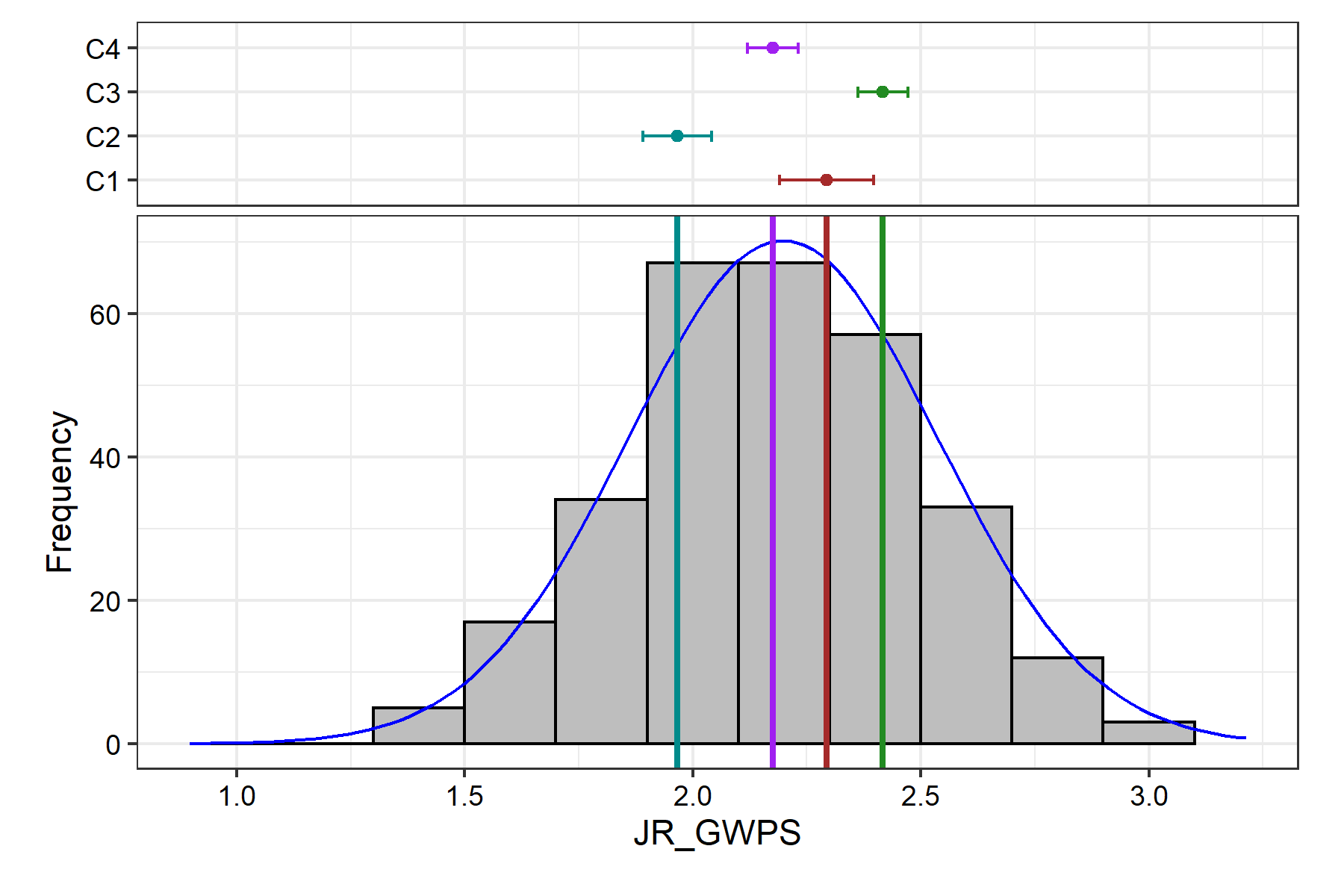

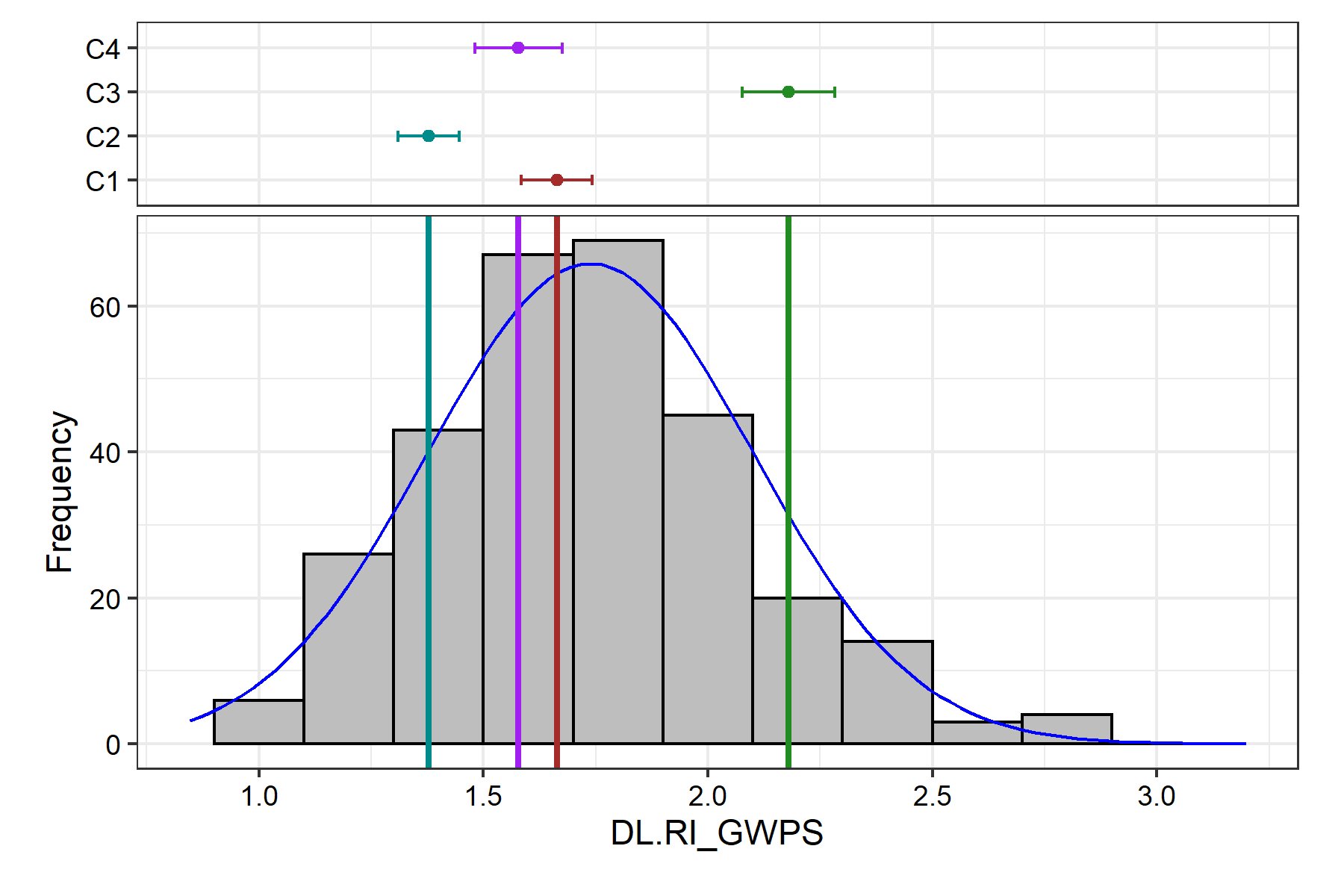

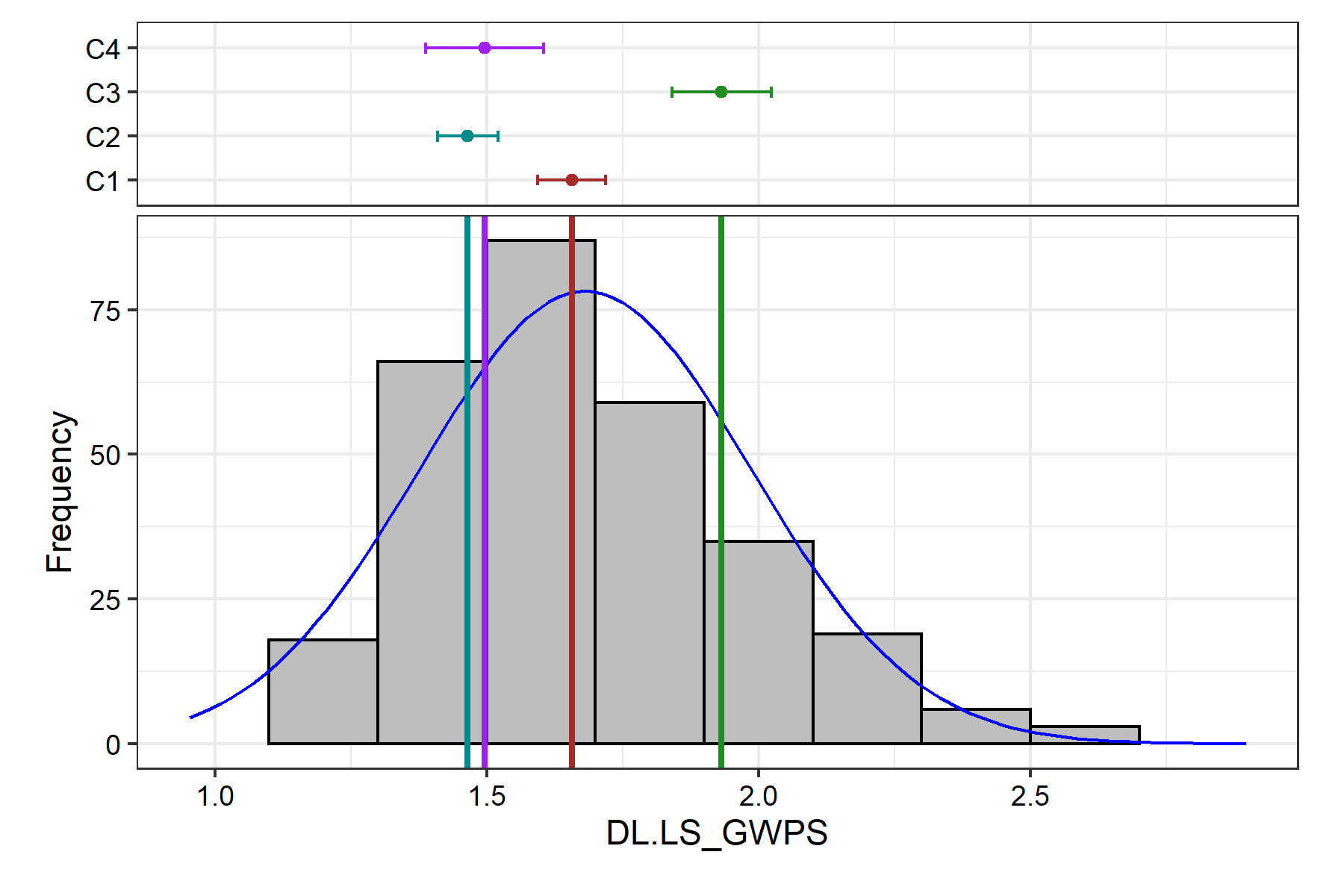

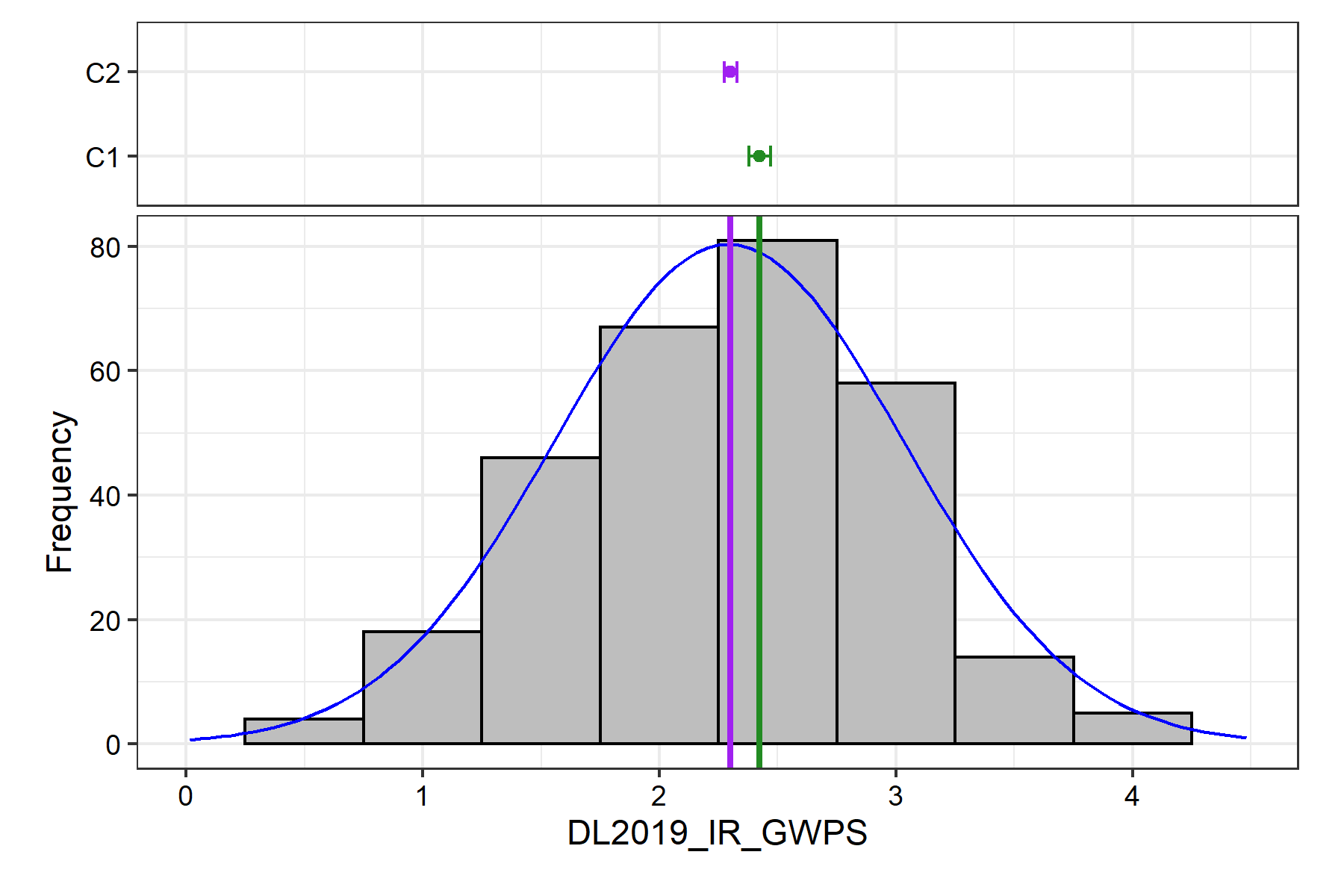

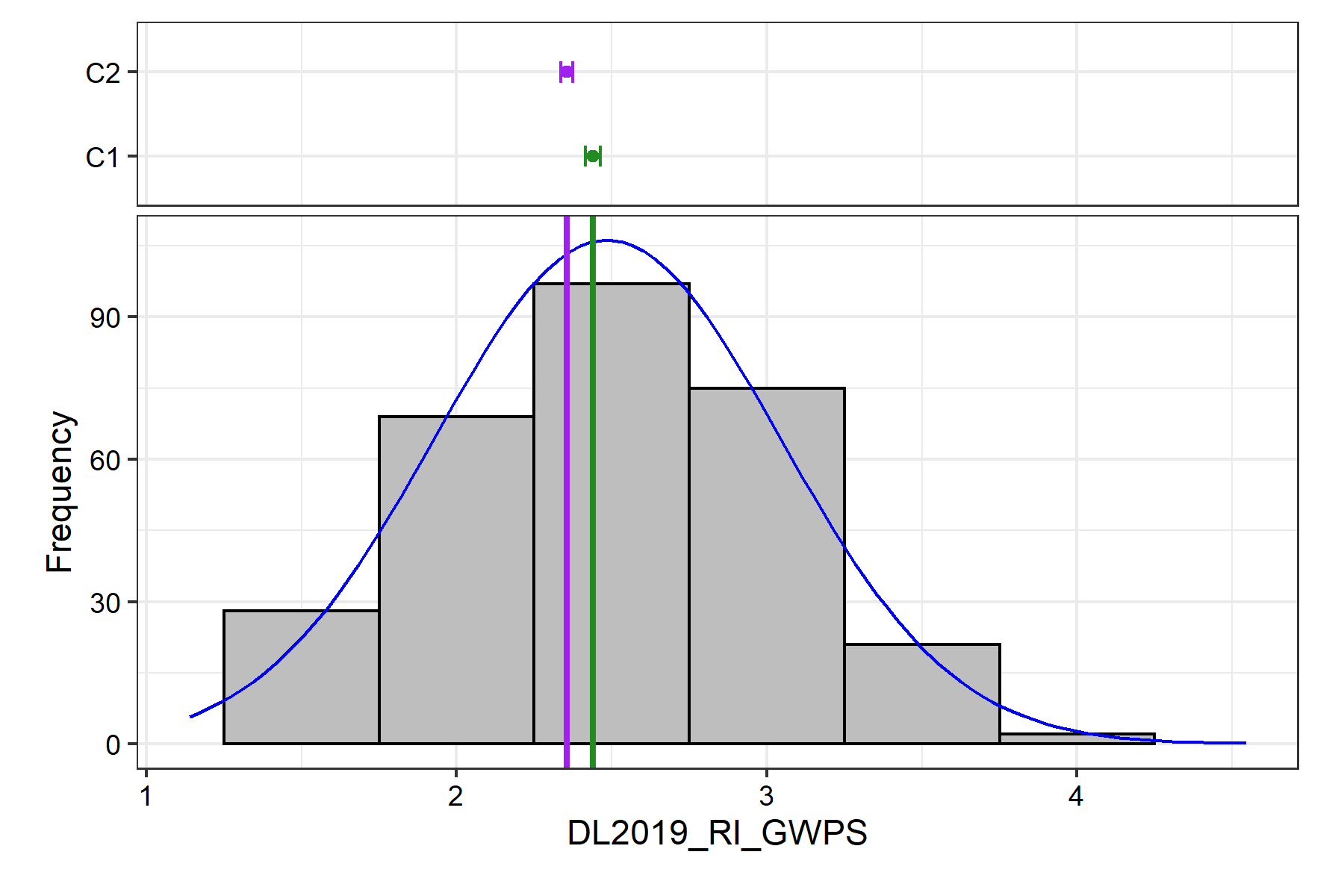

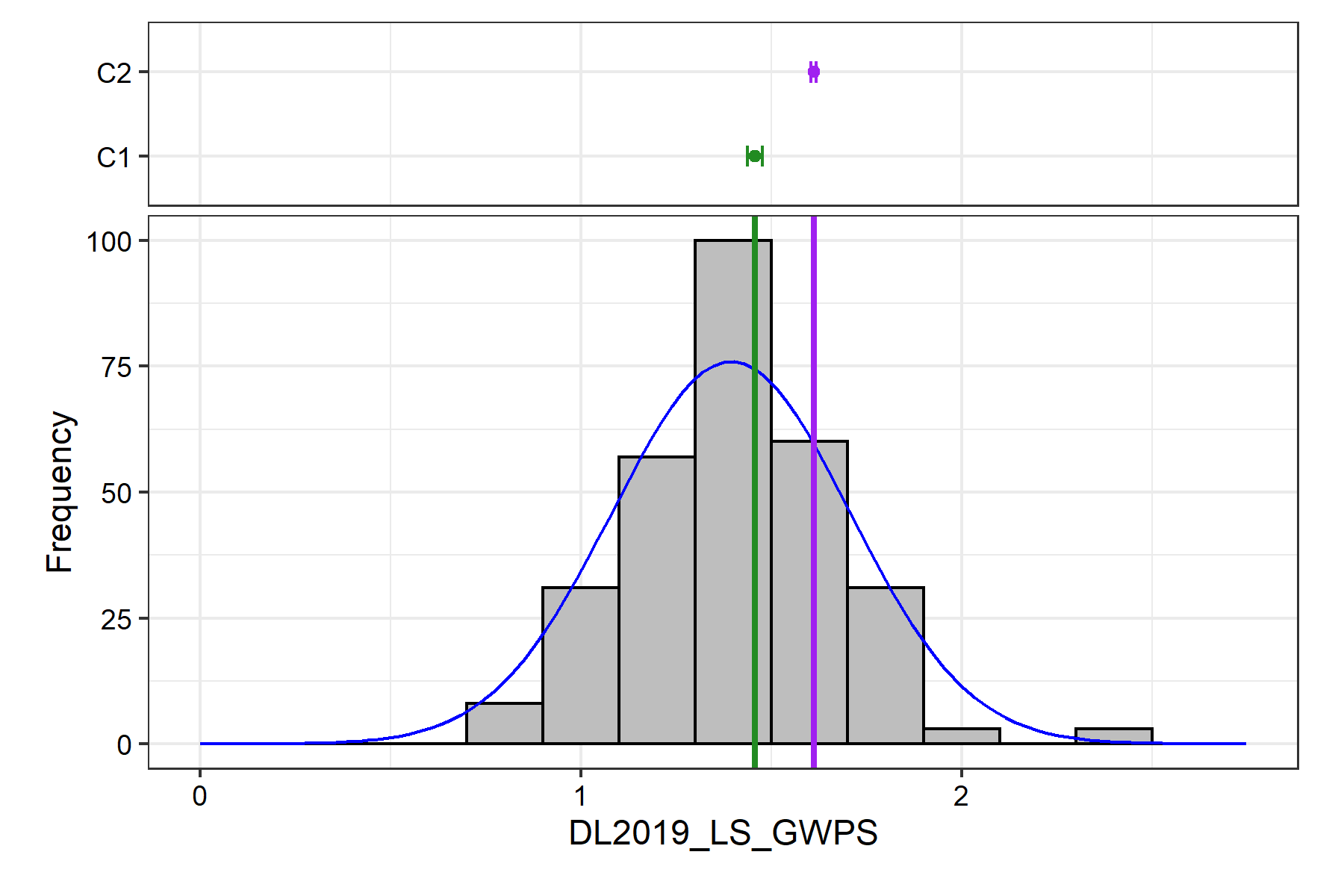


DH


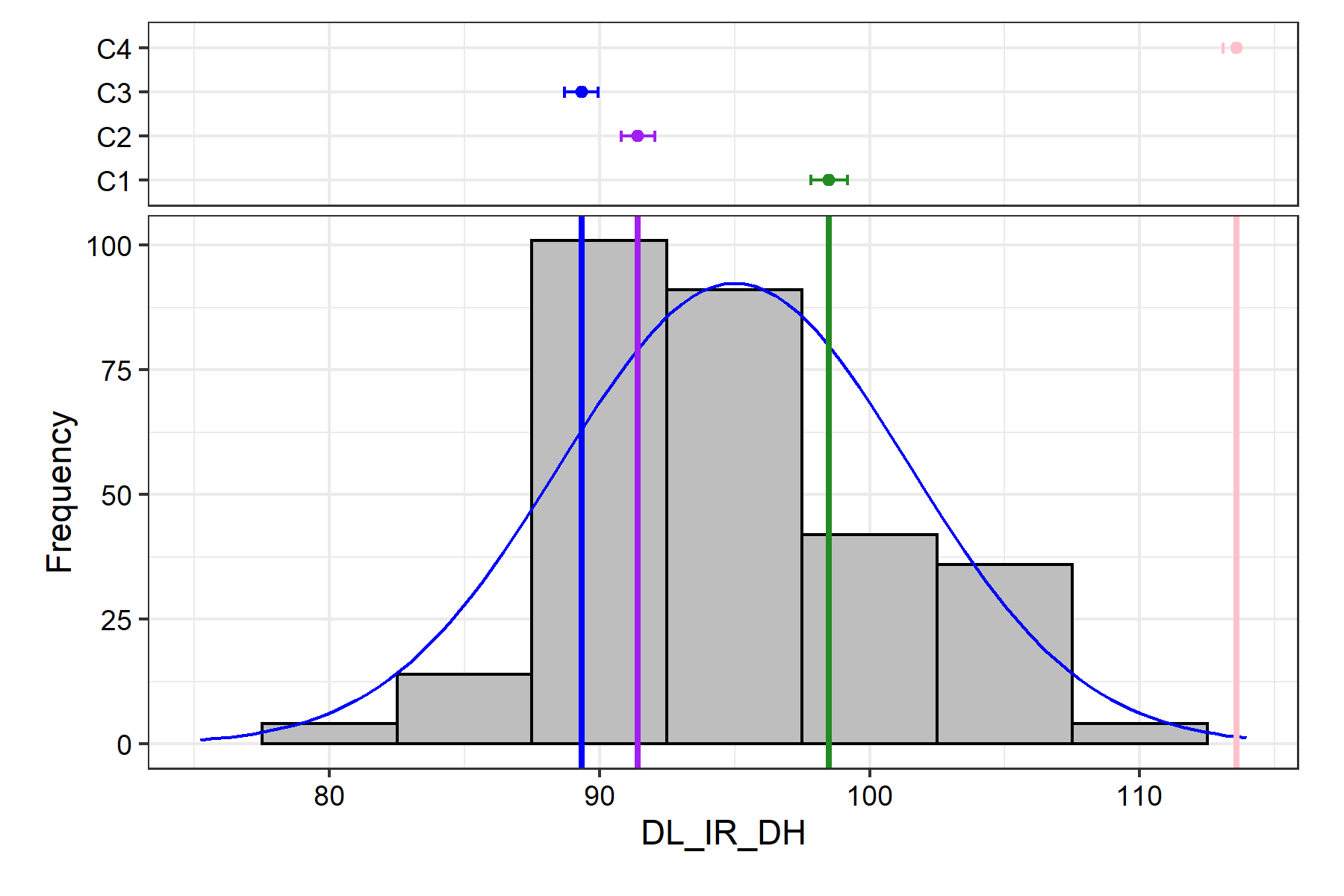

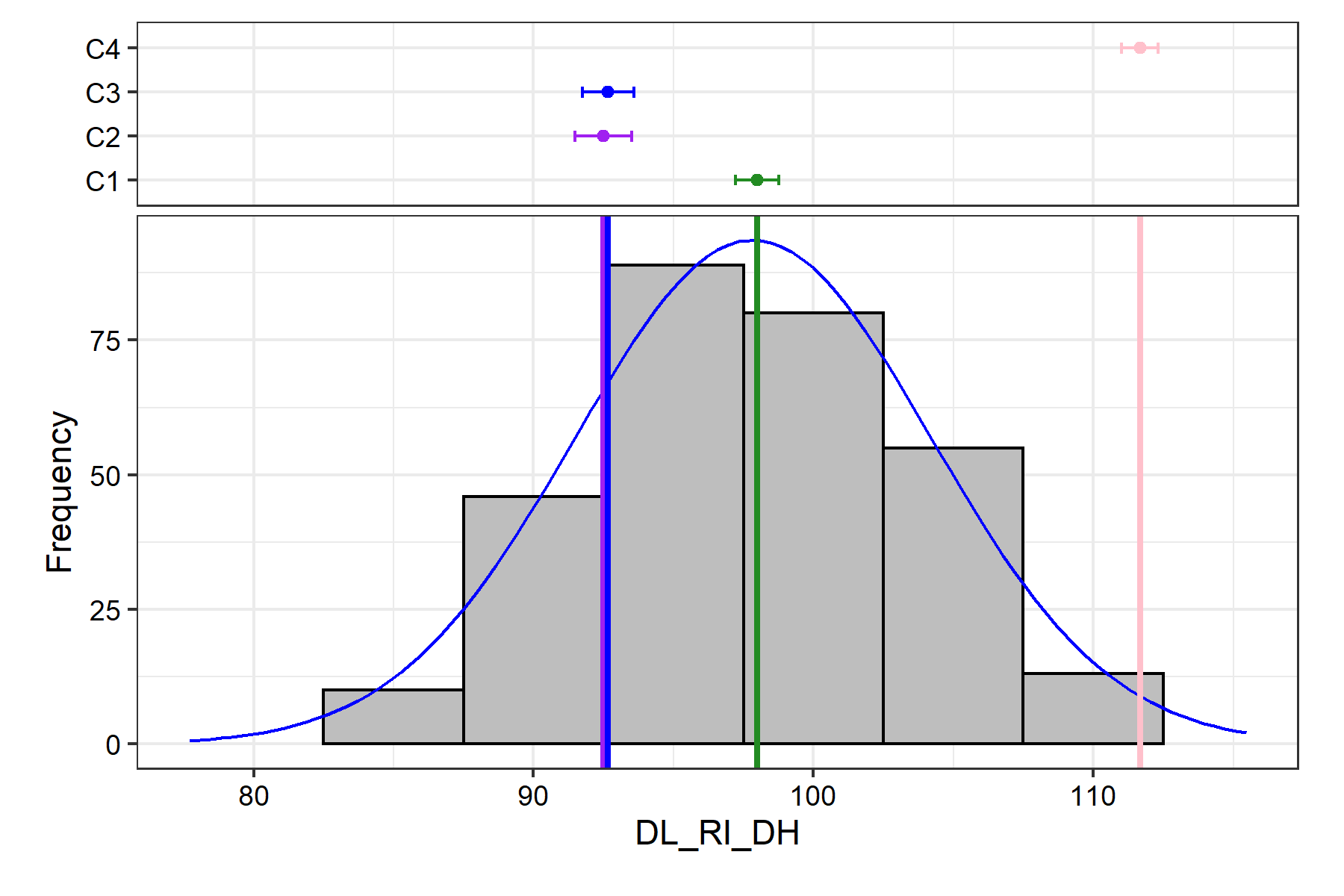

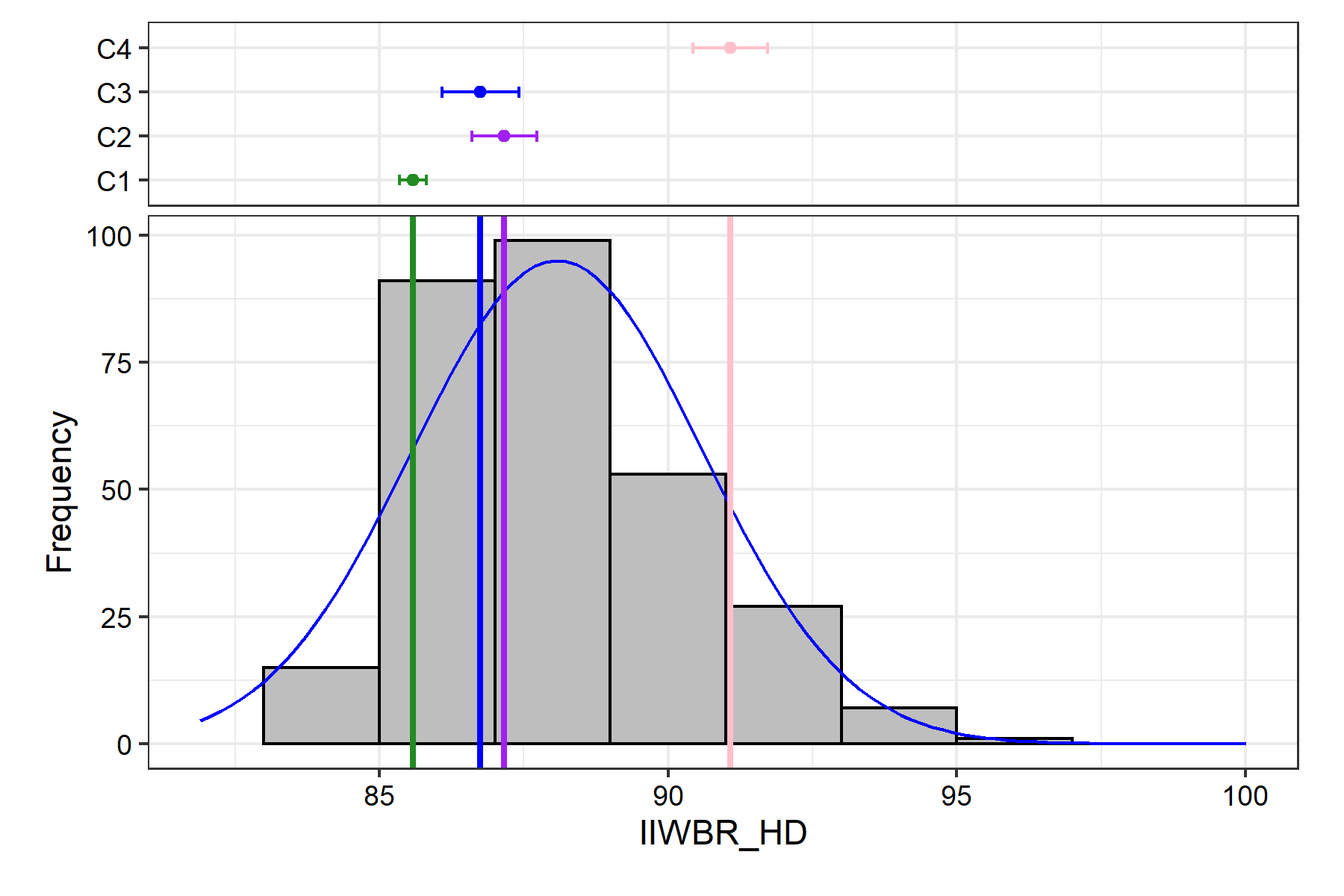

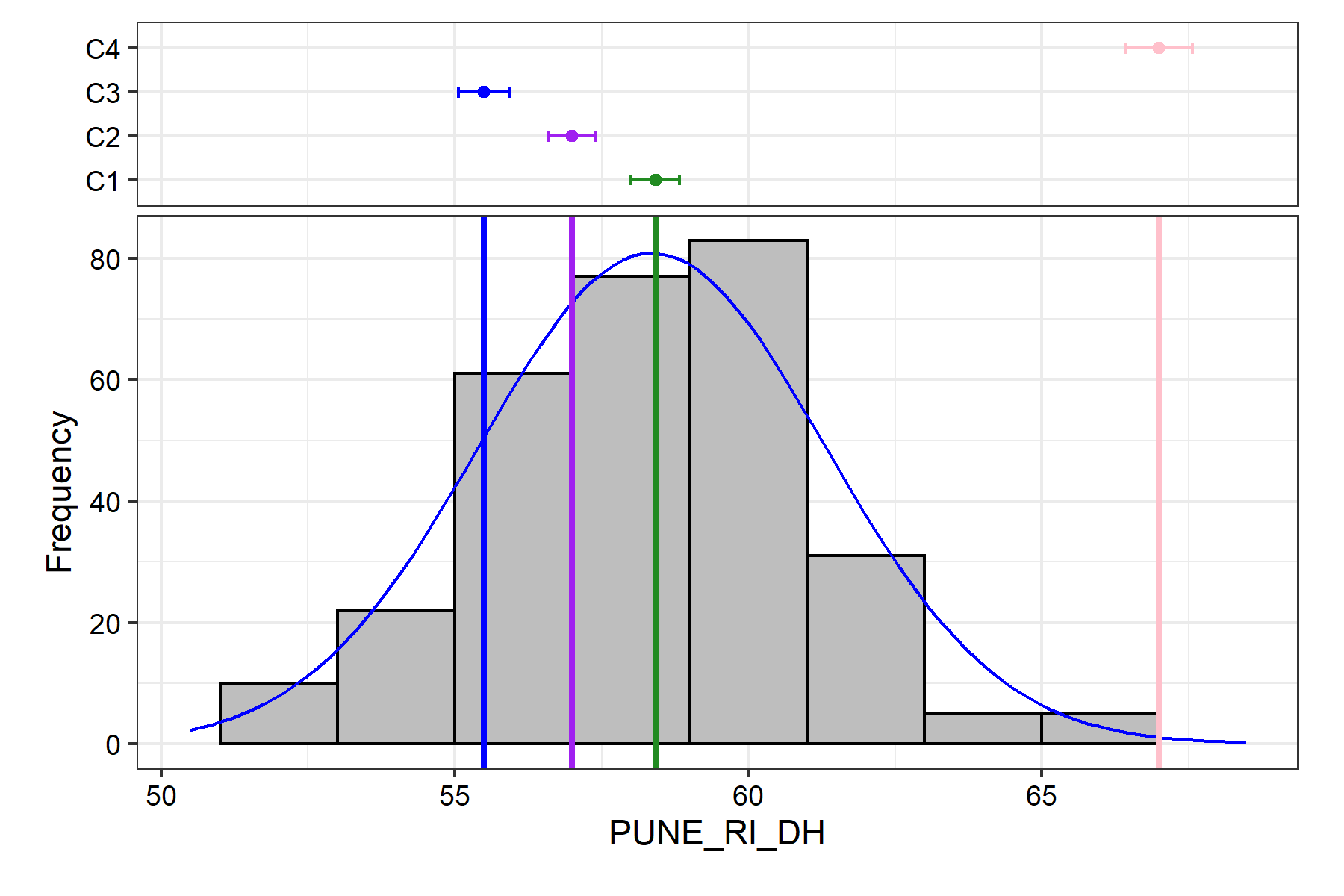

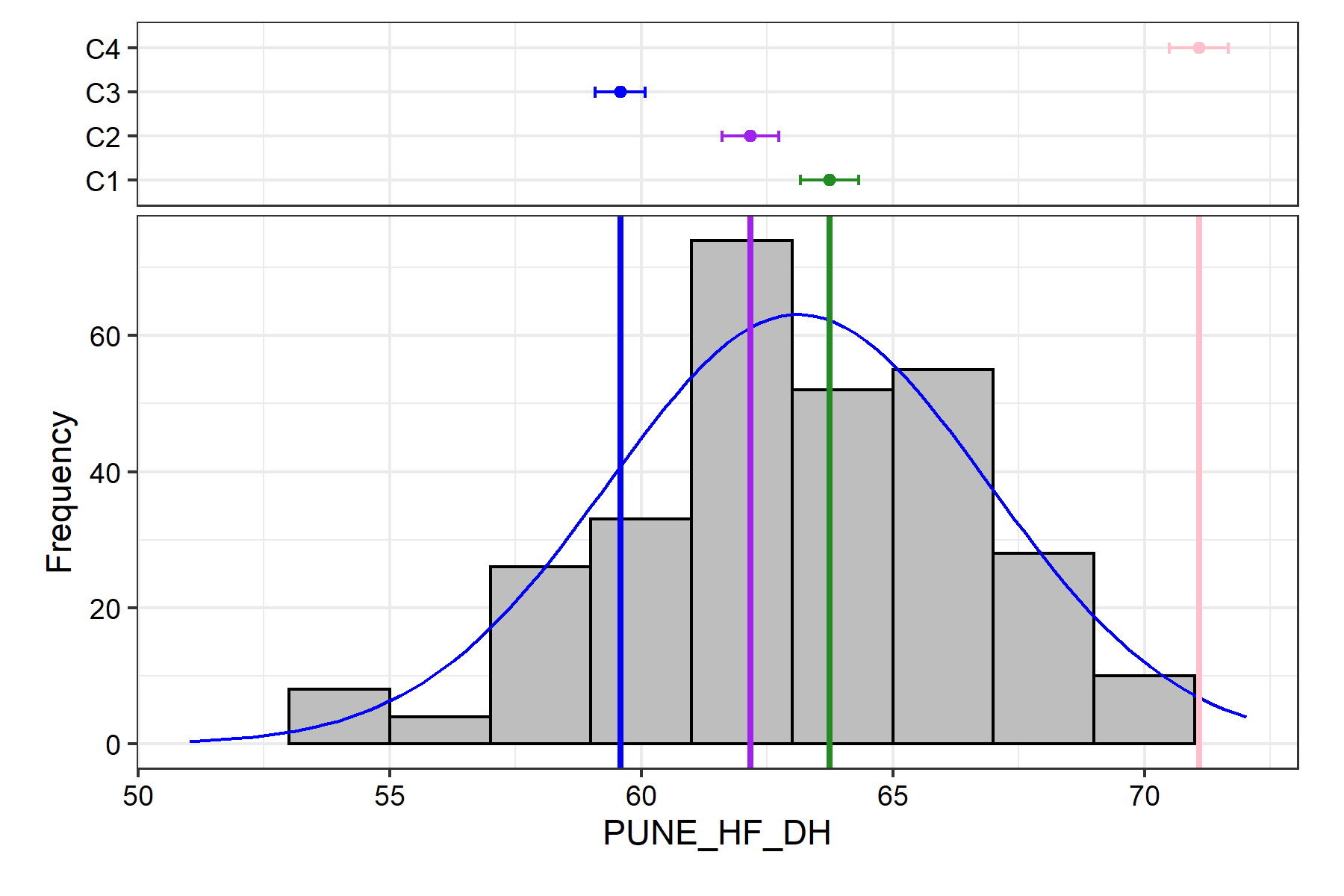

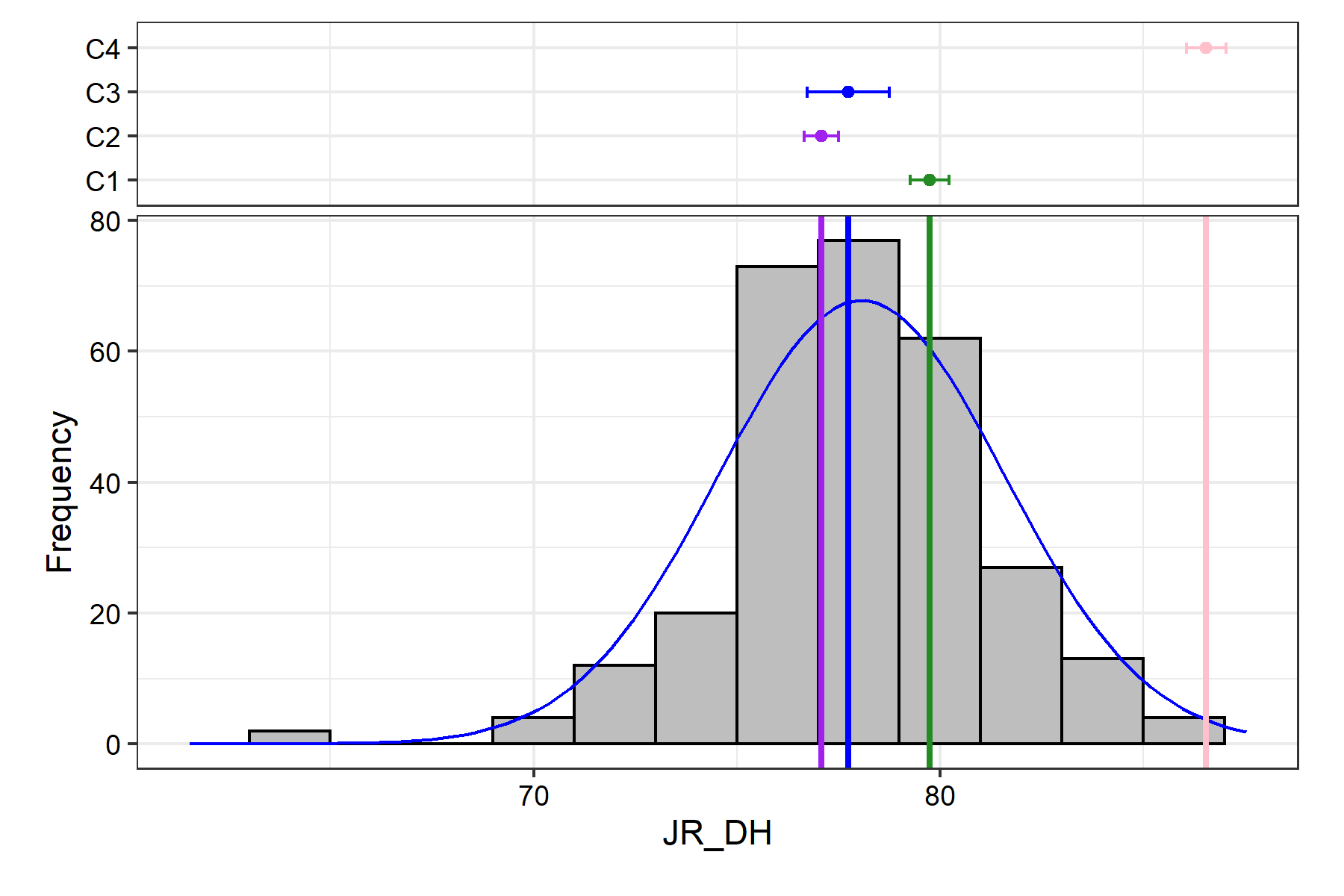

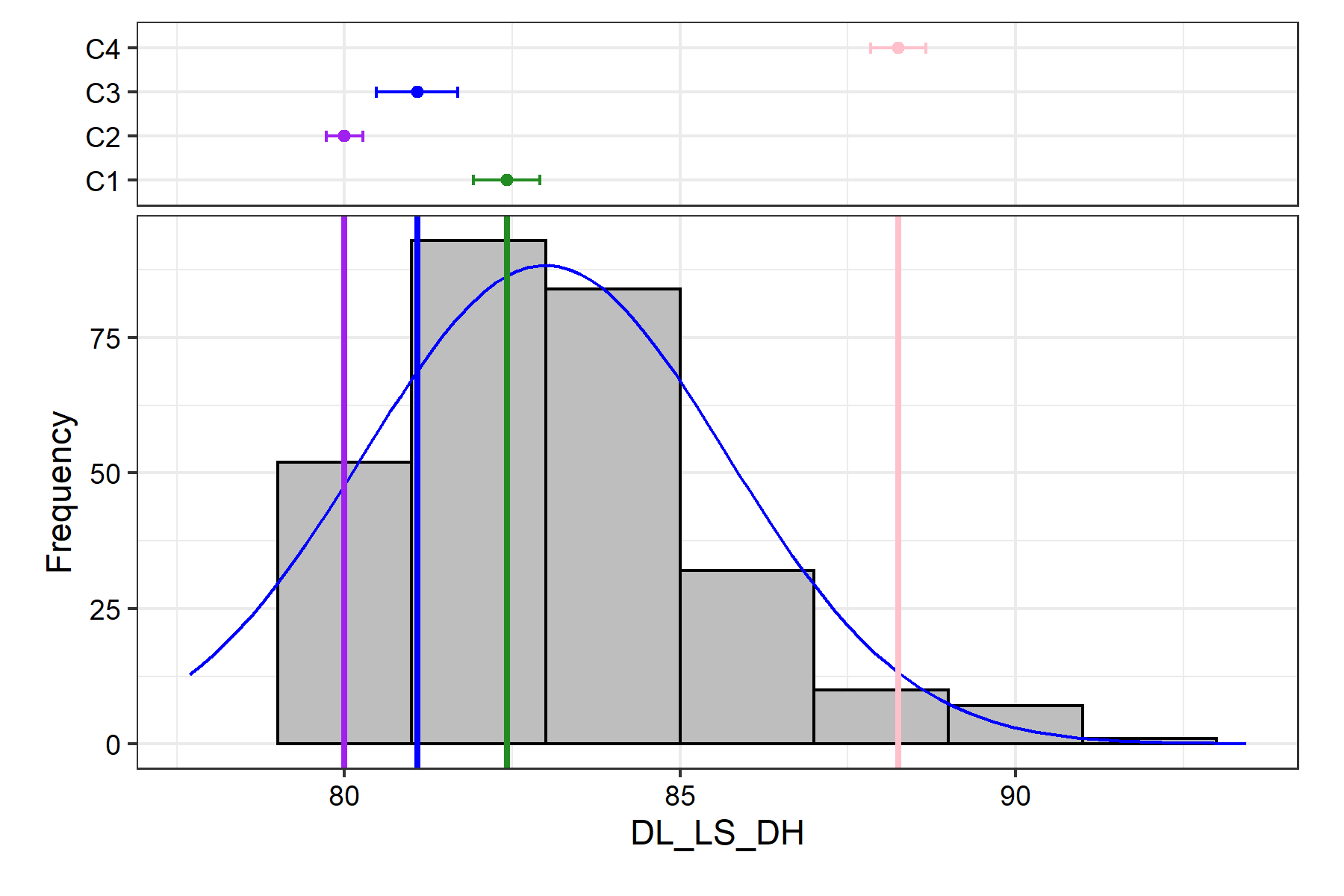

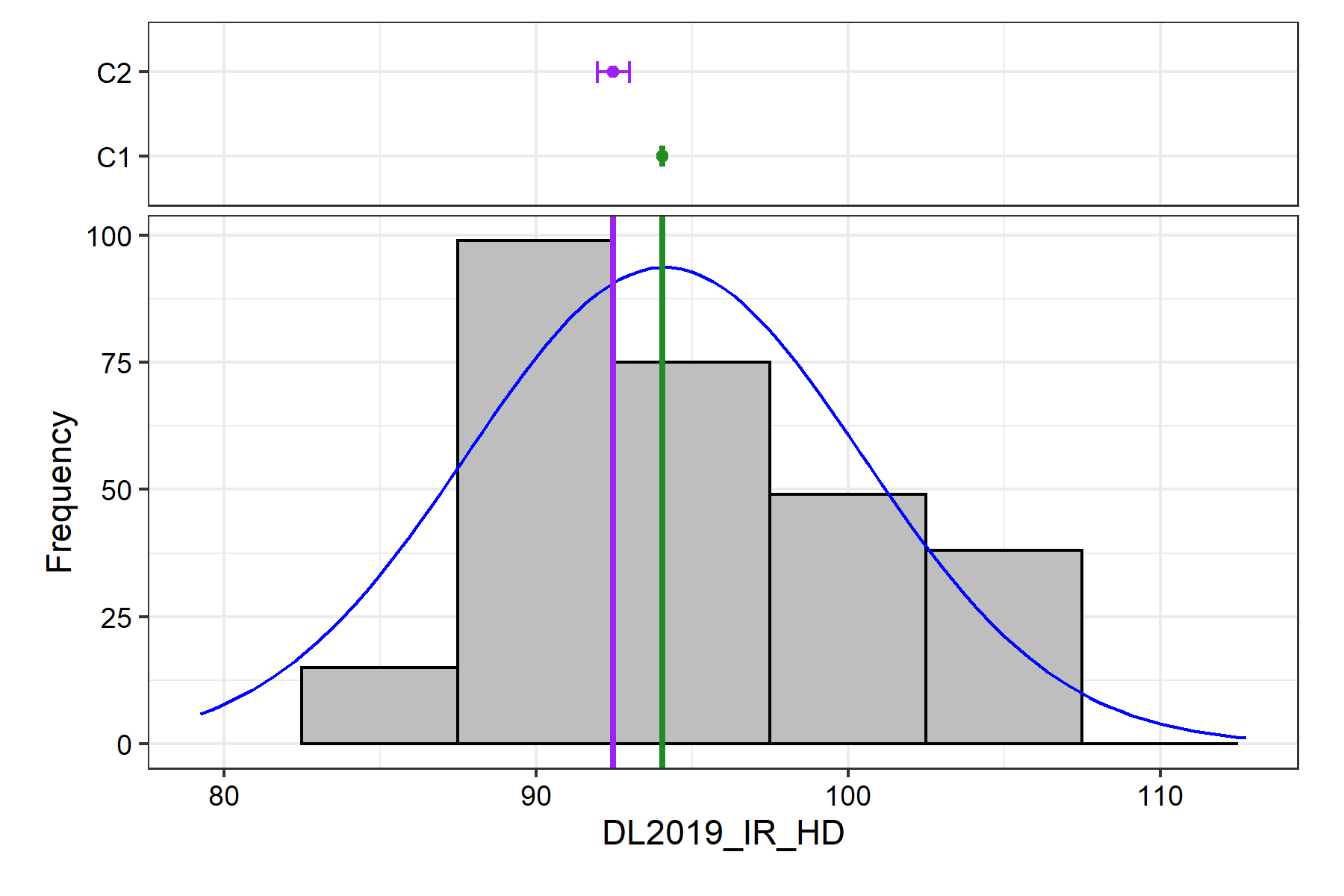

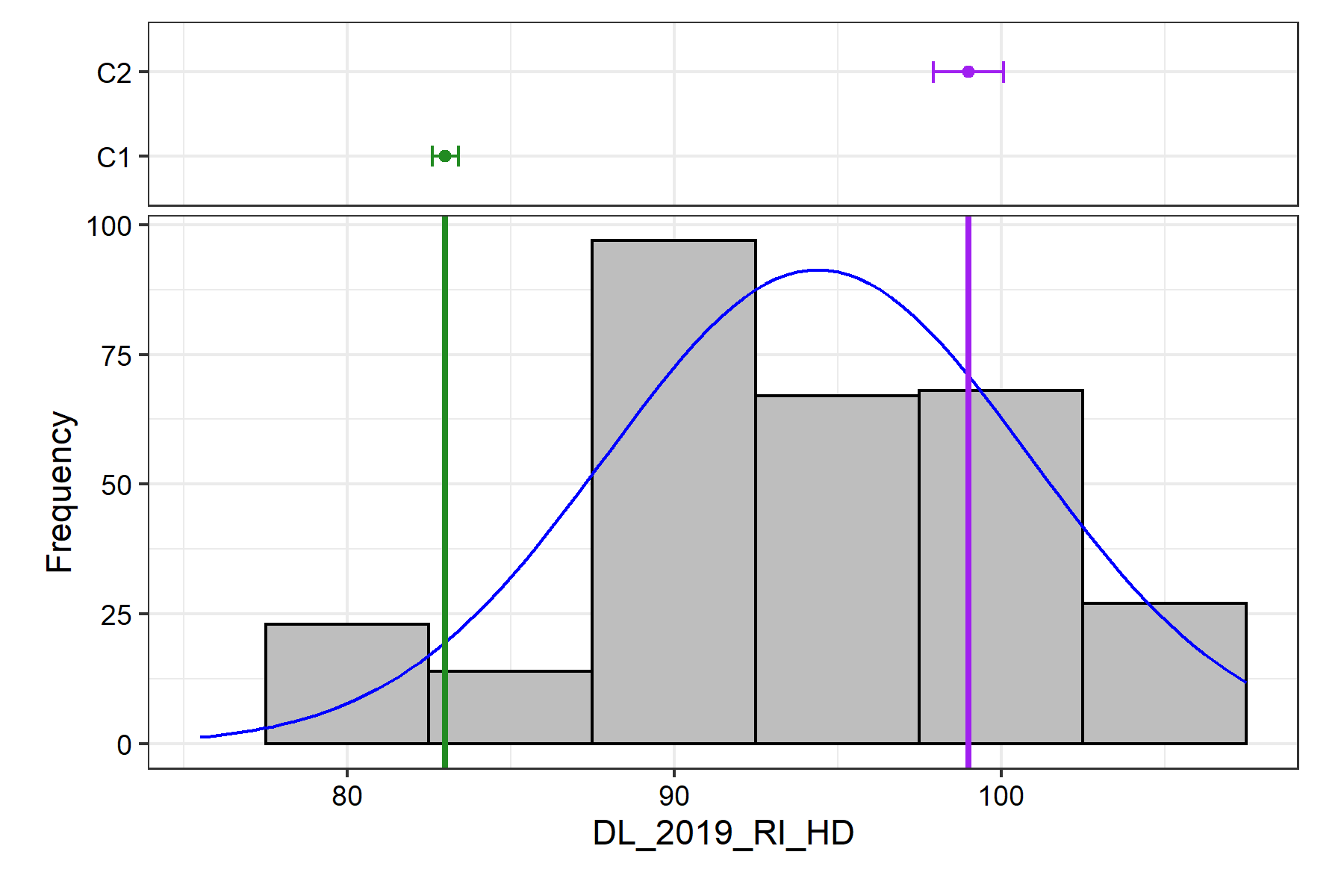

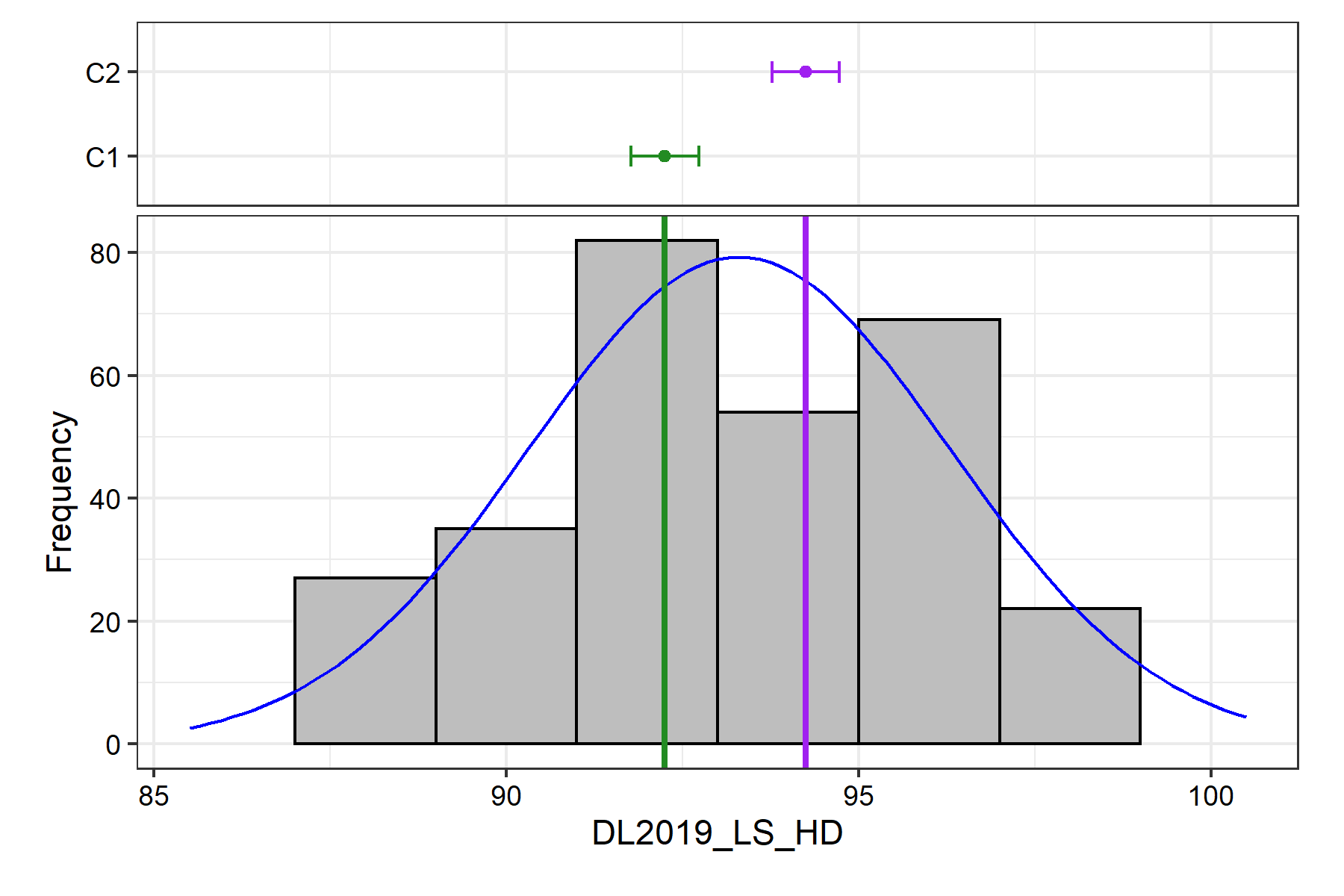


PH


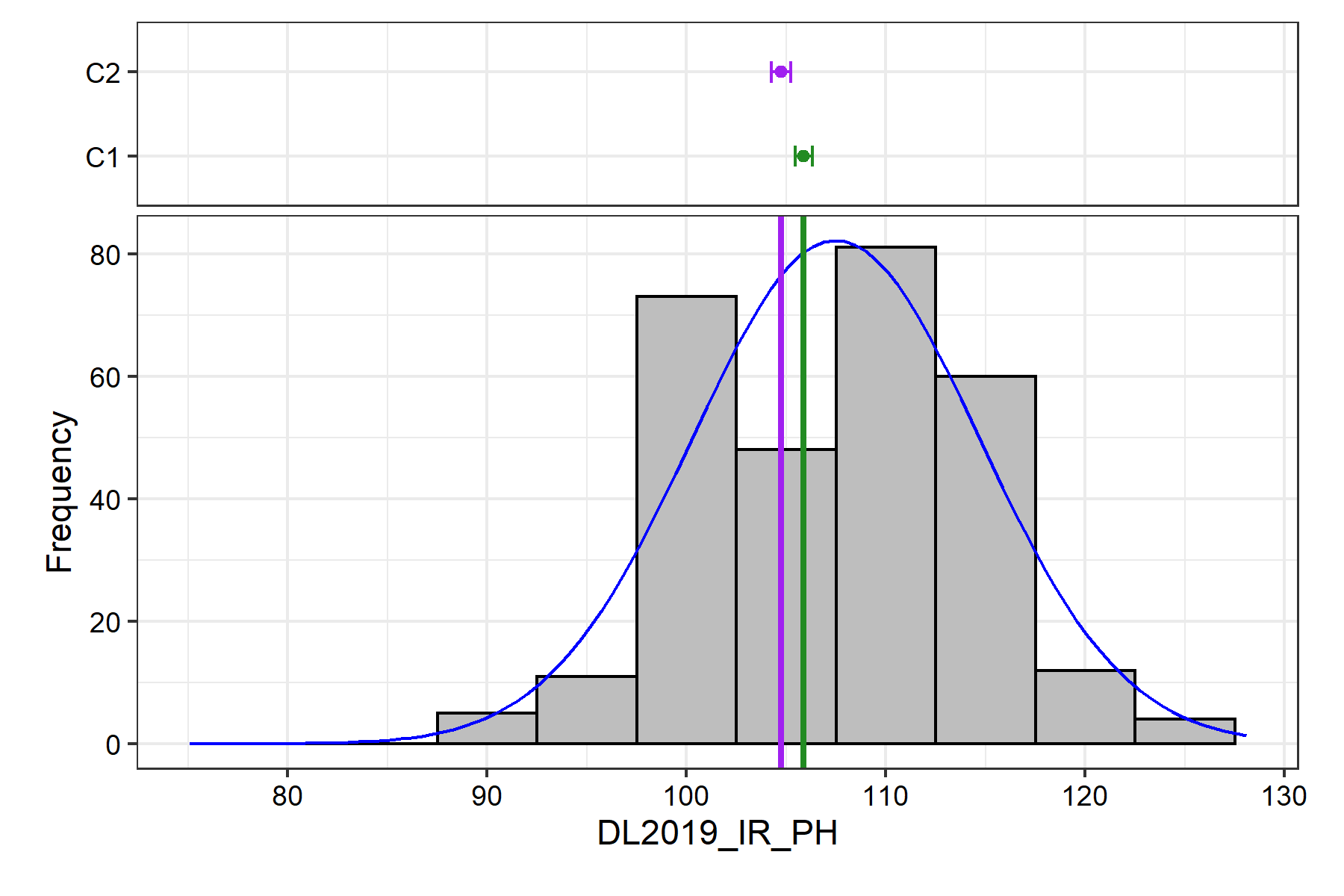

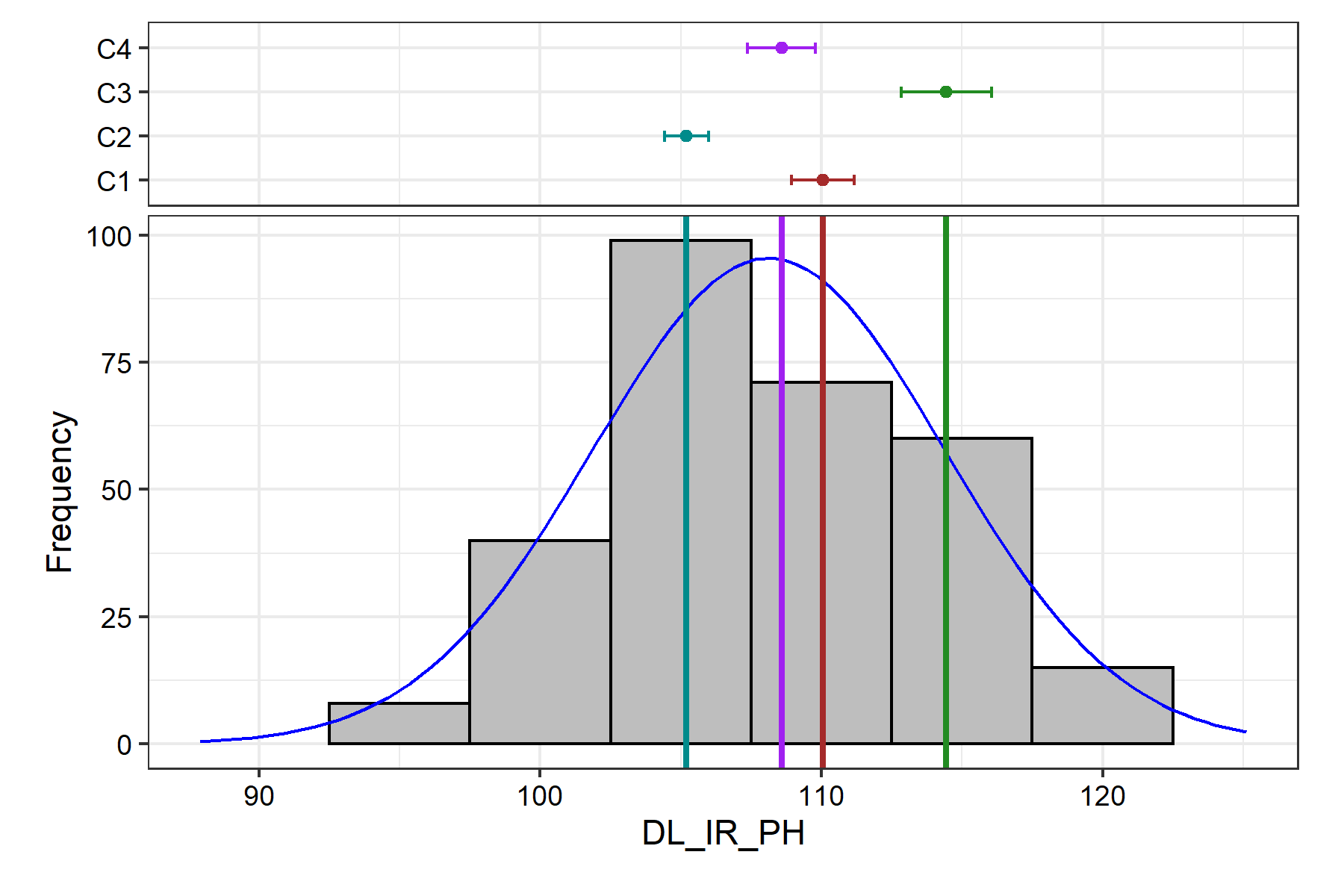

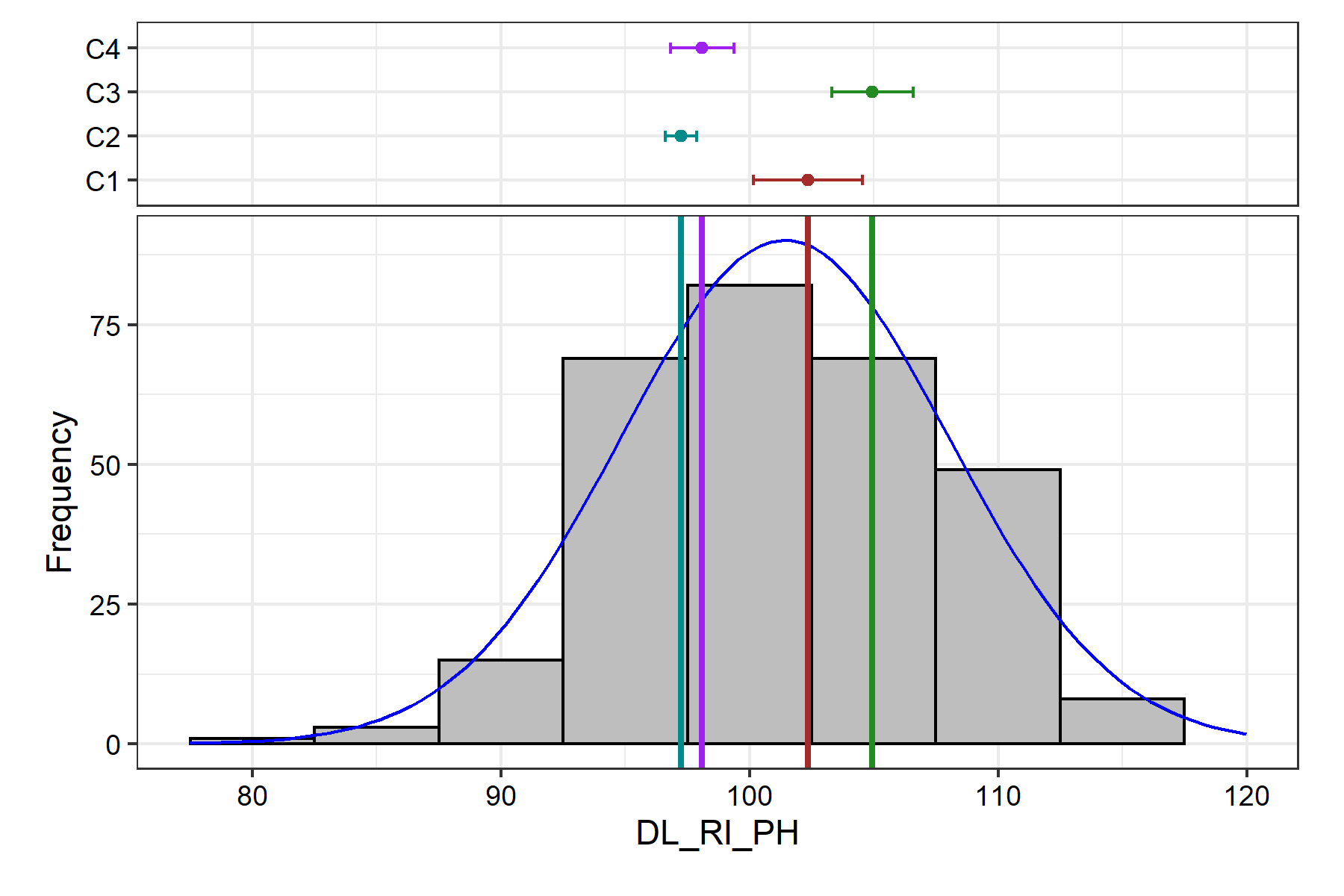

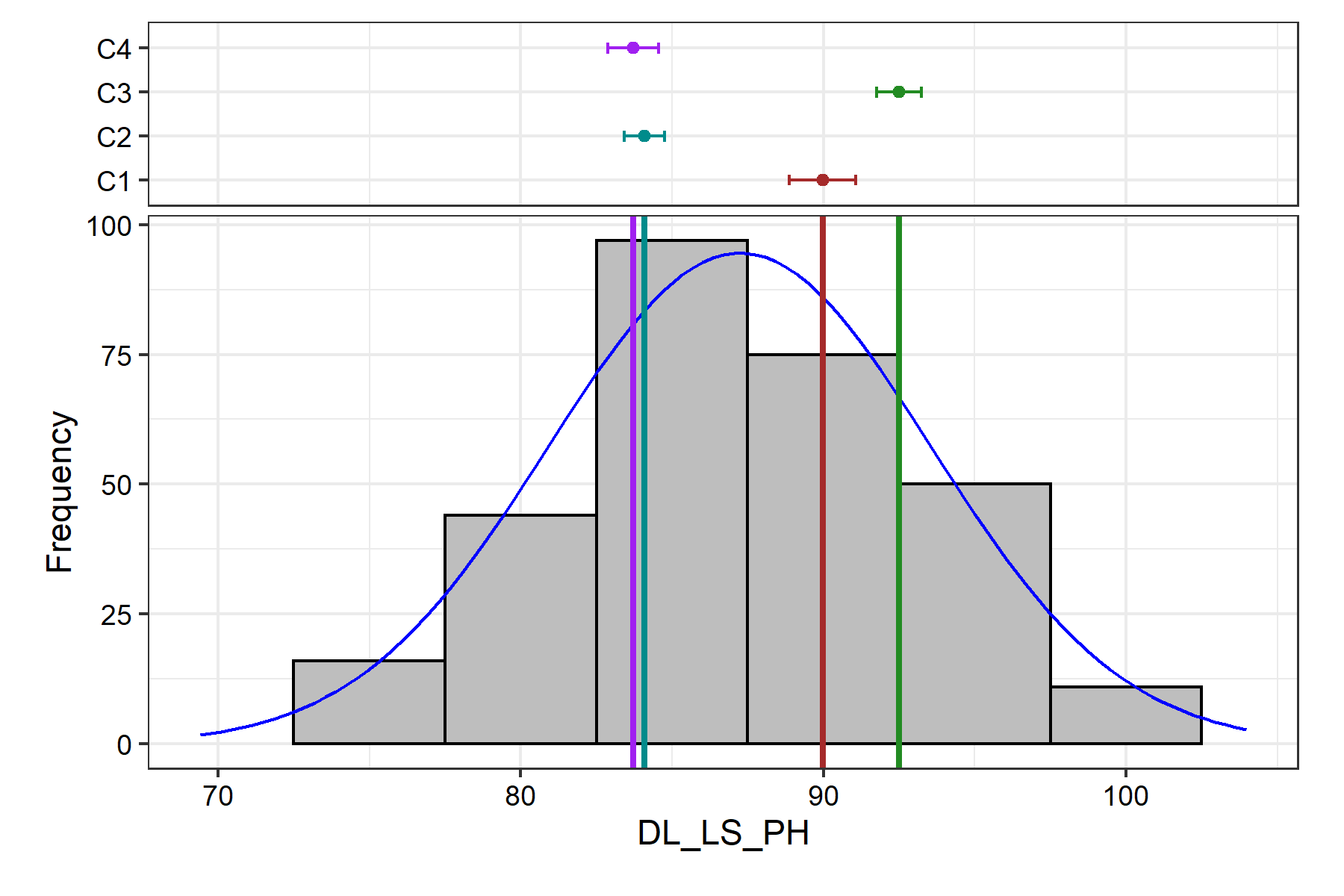

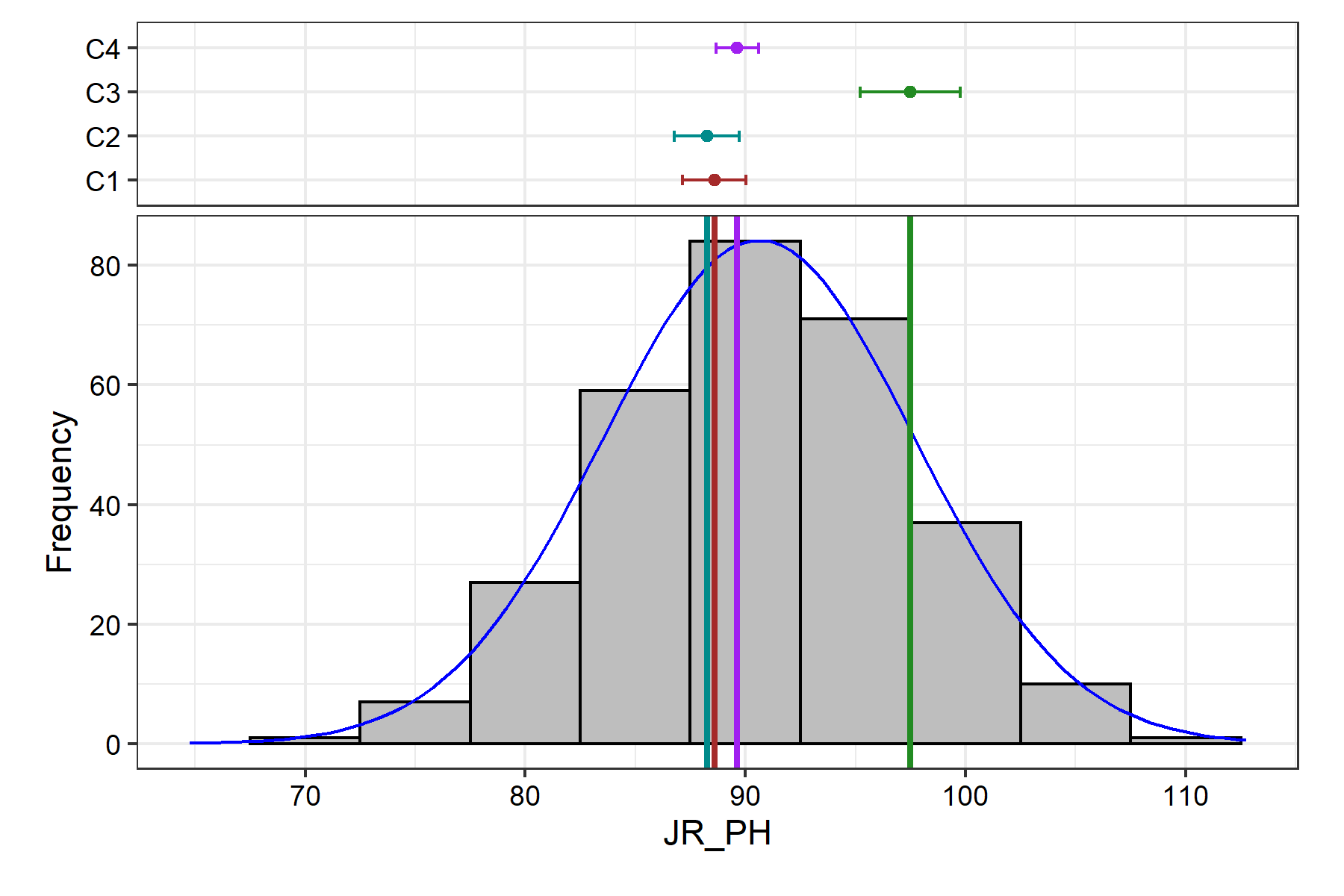


TGW


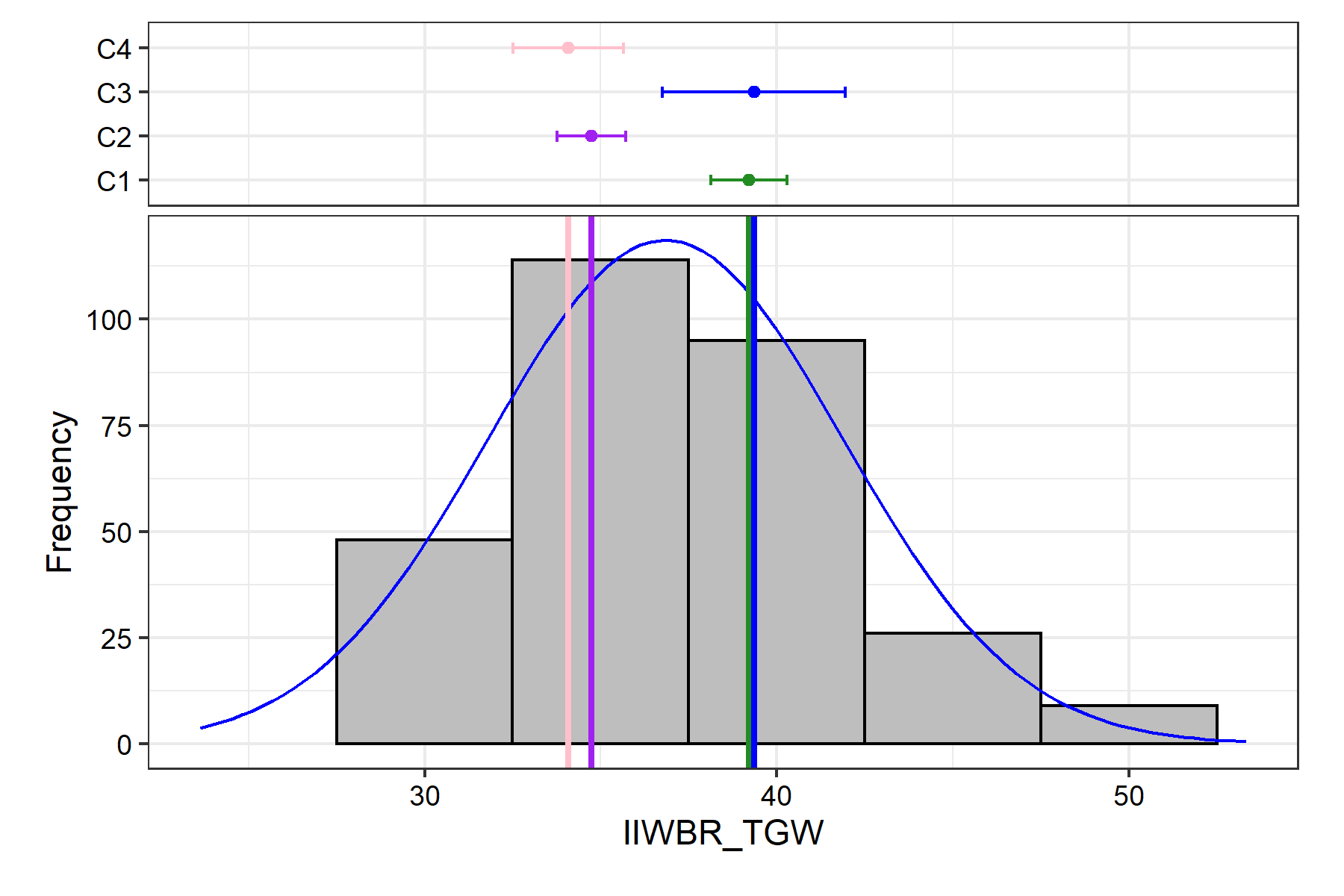

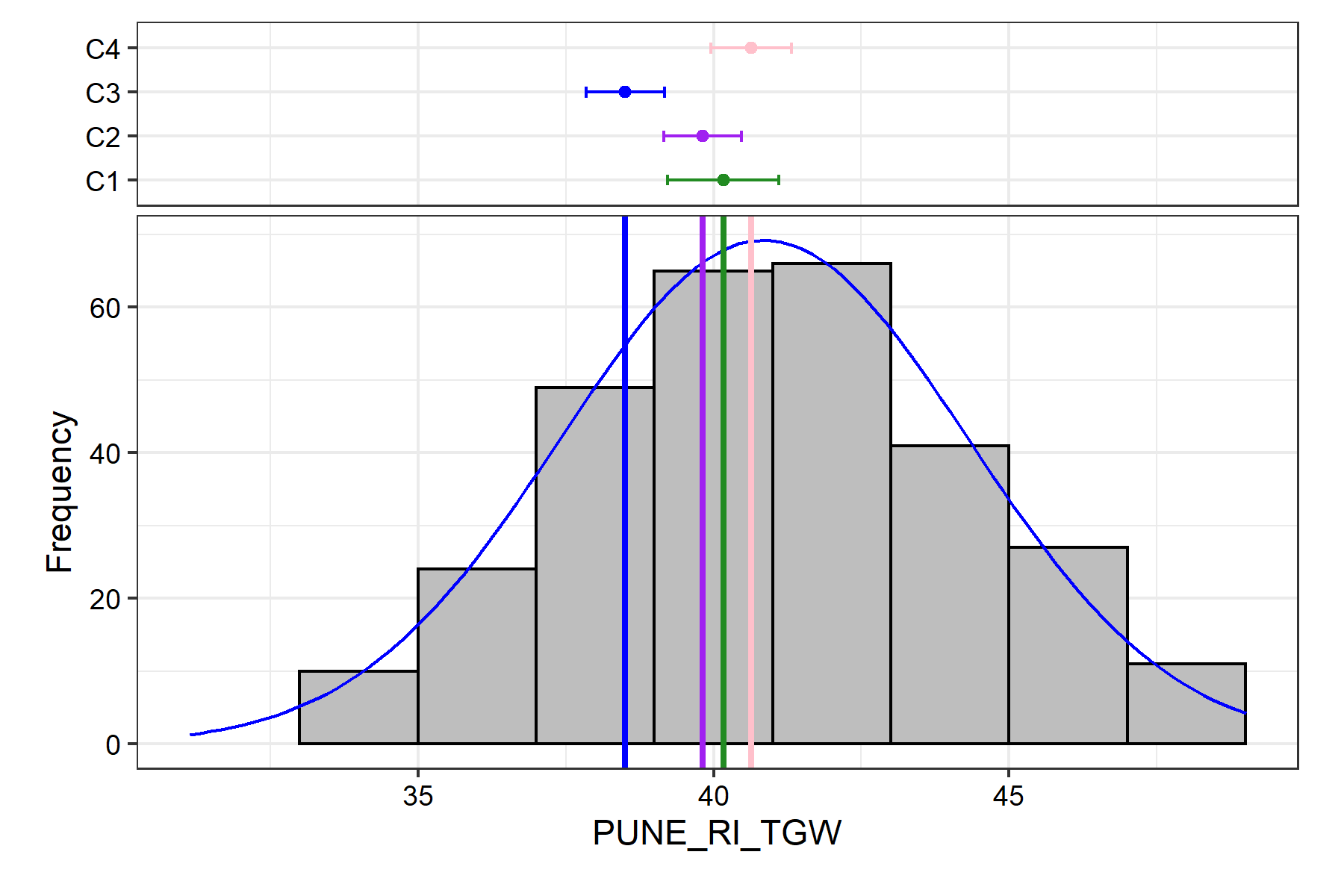

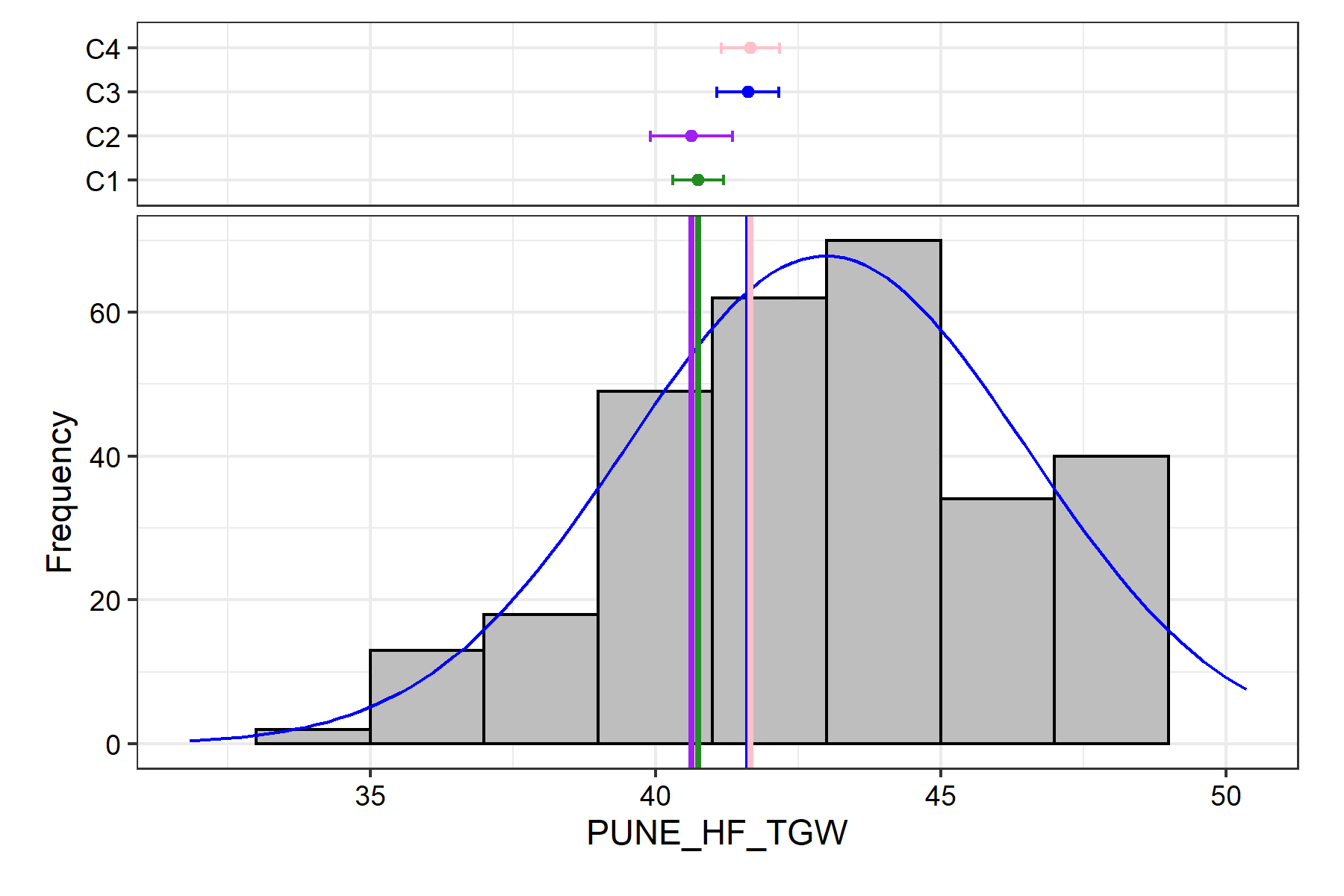

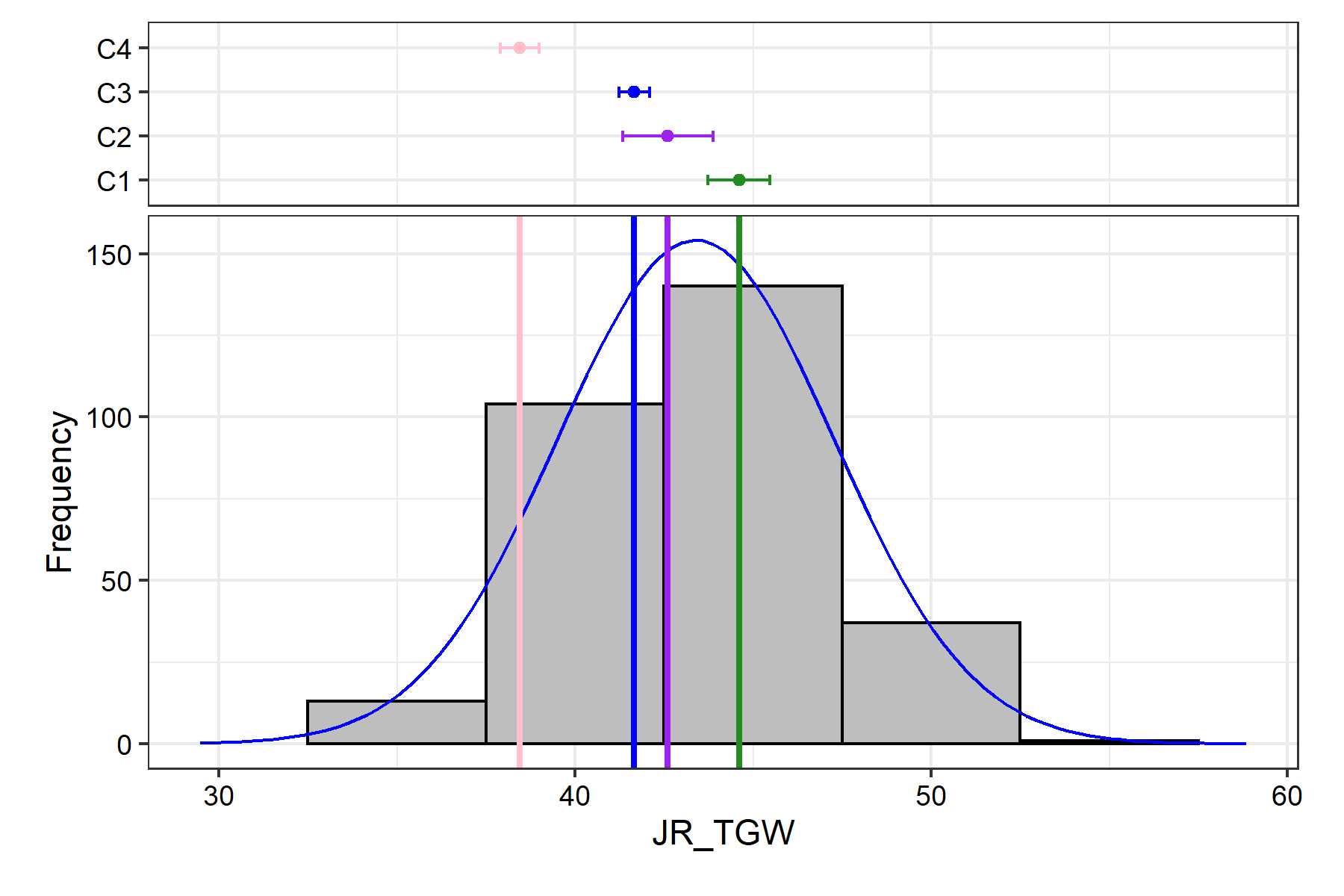

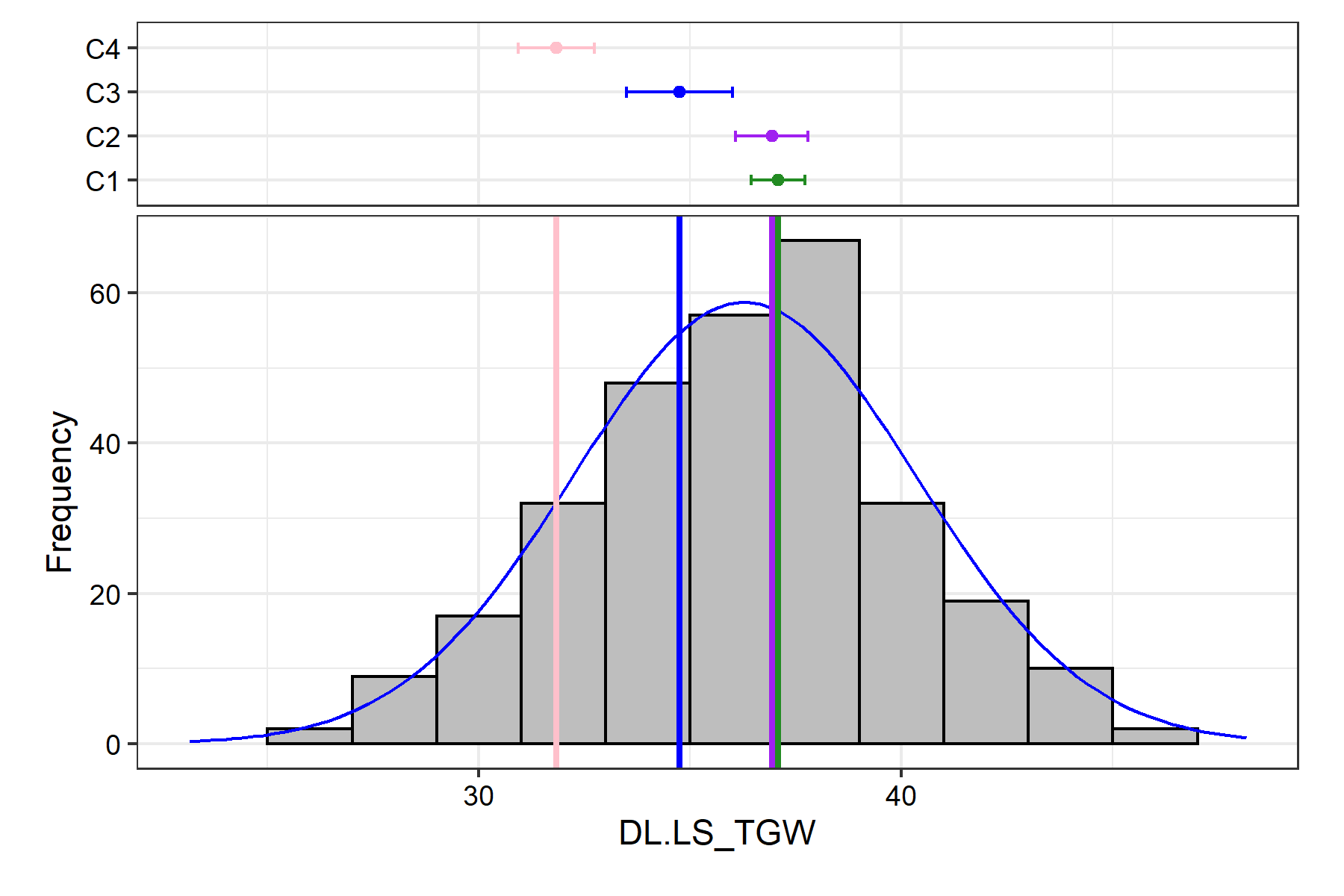

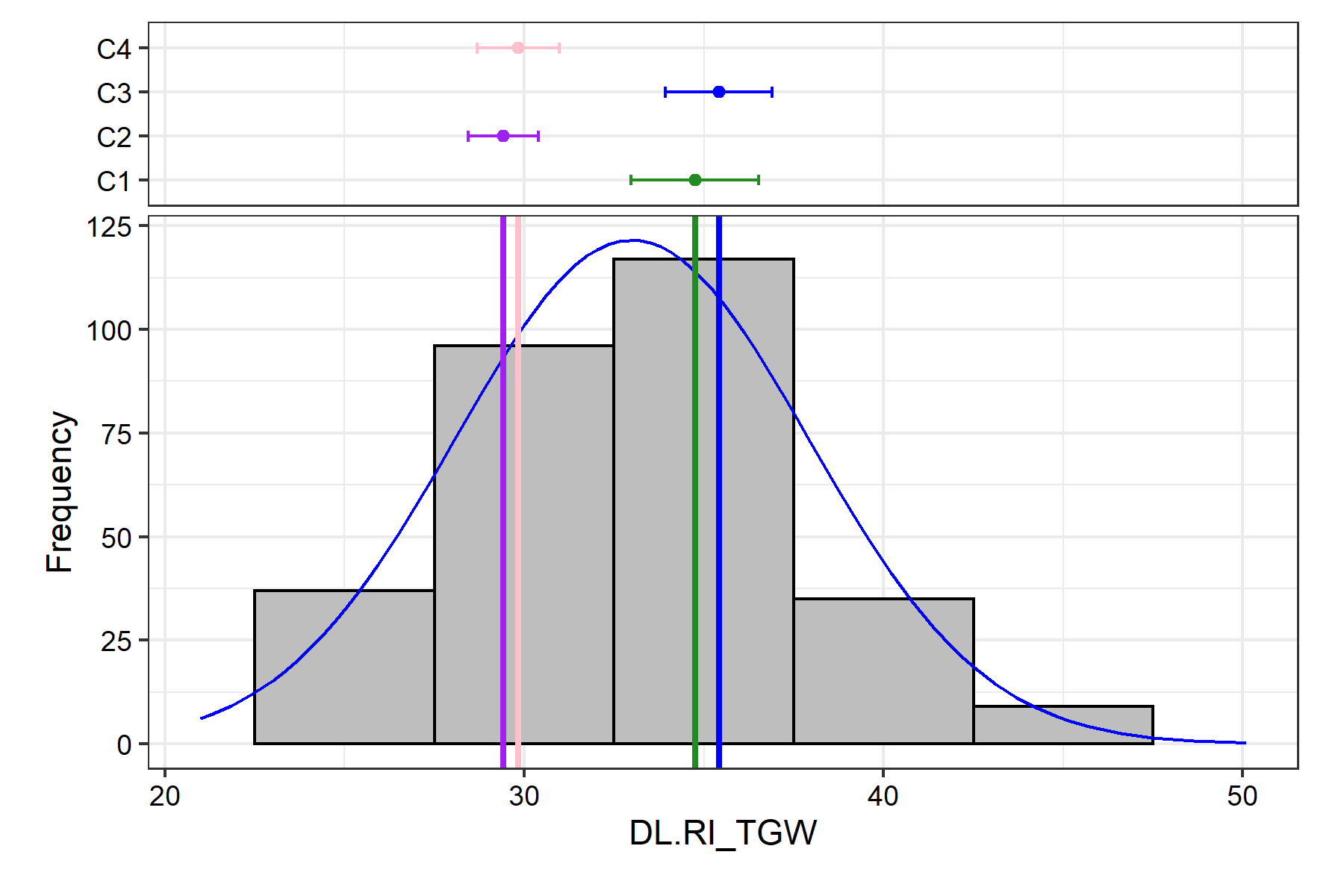

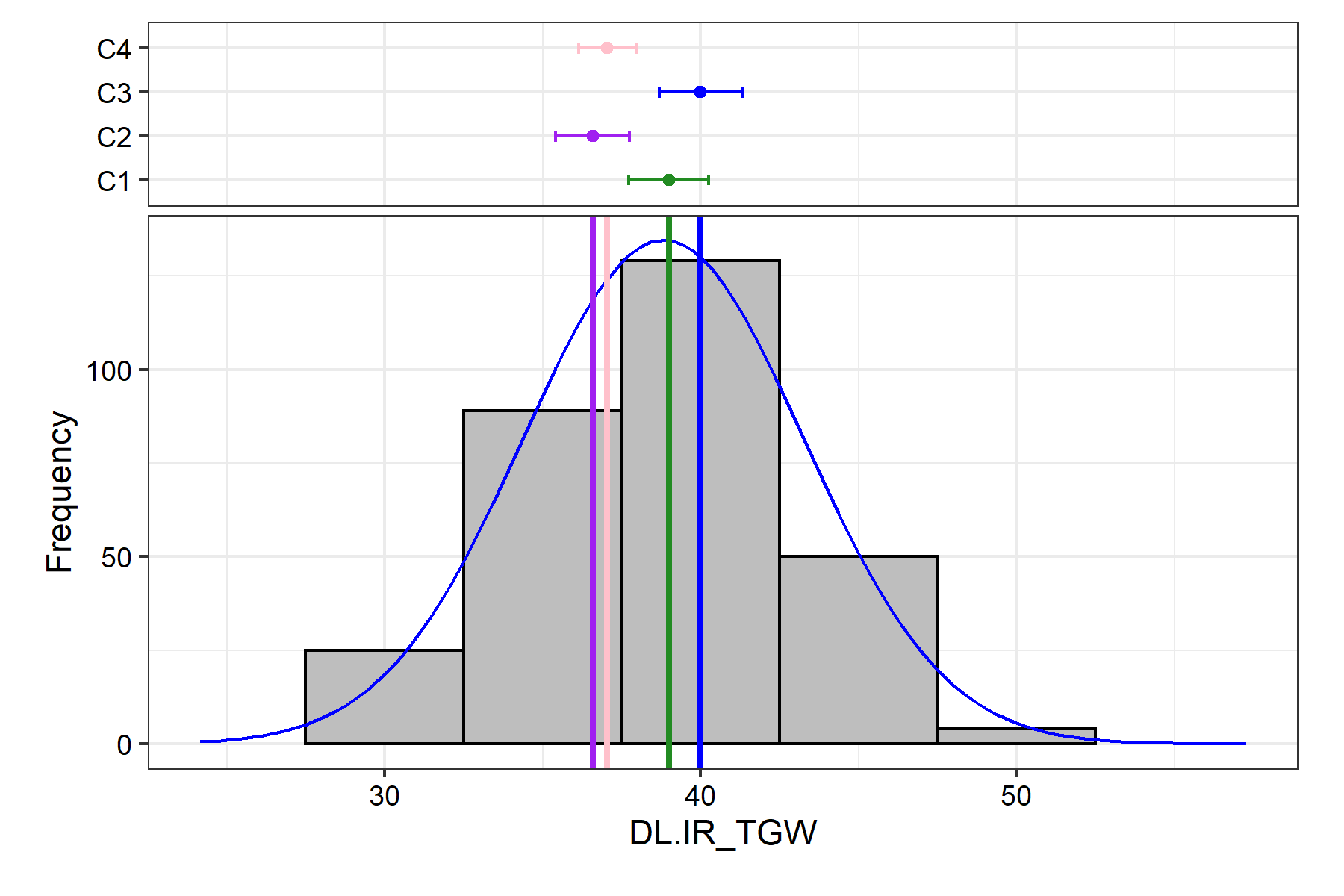

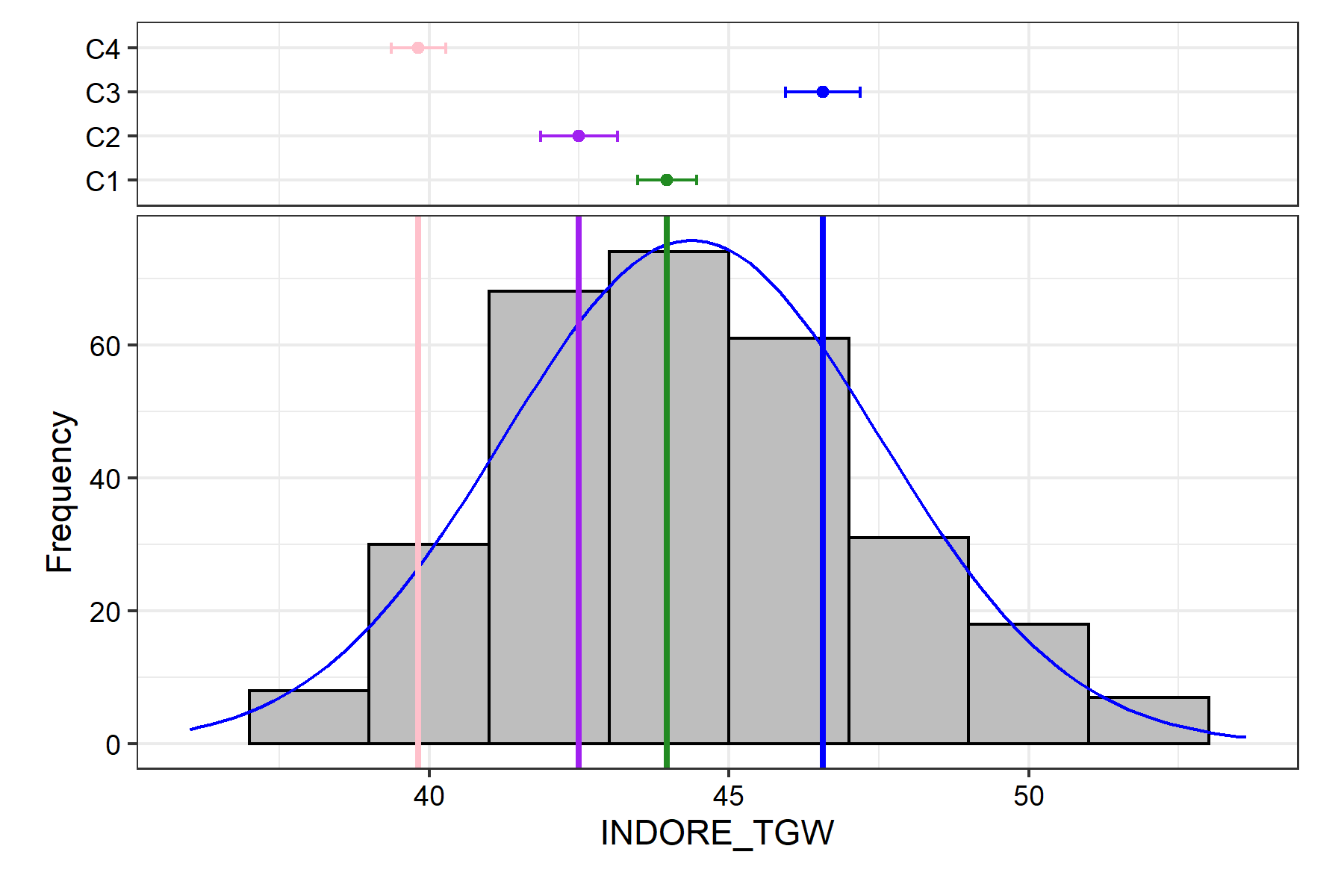


PLTY


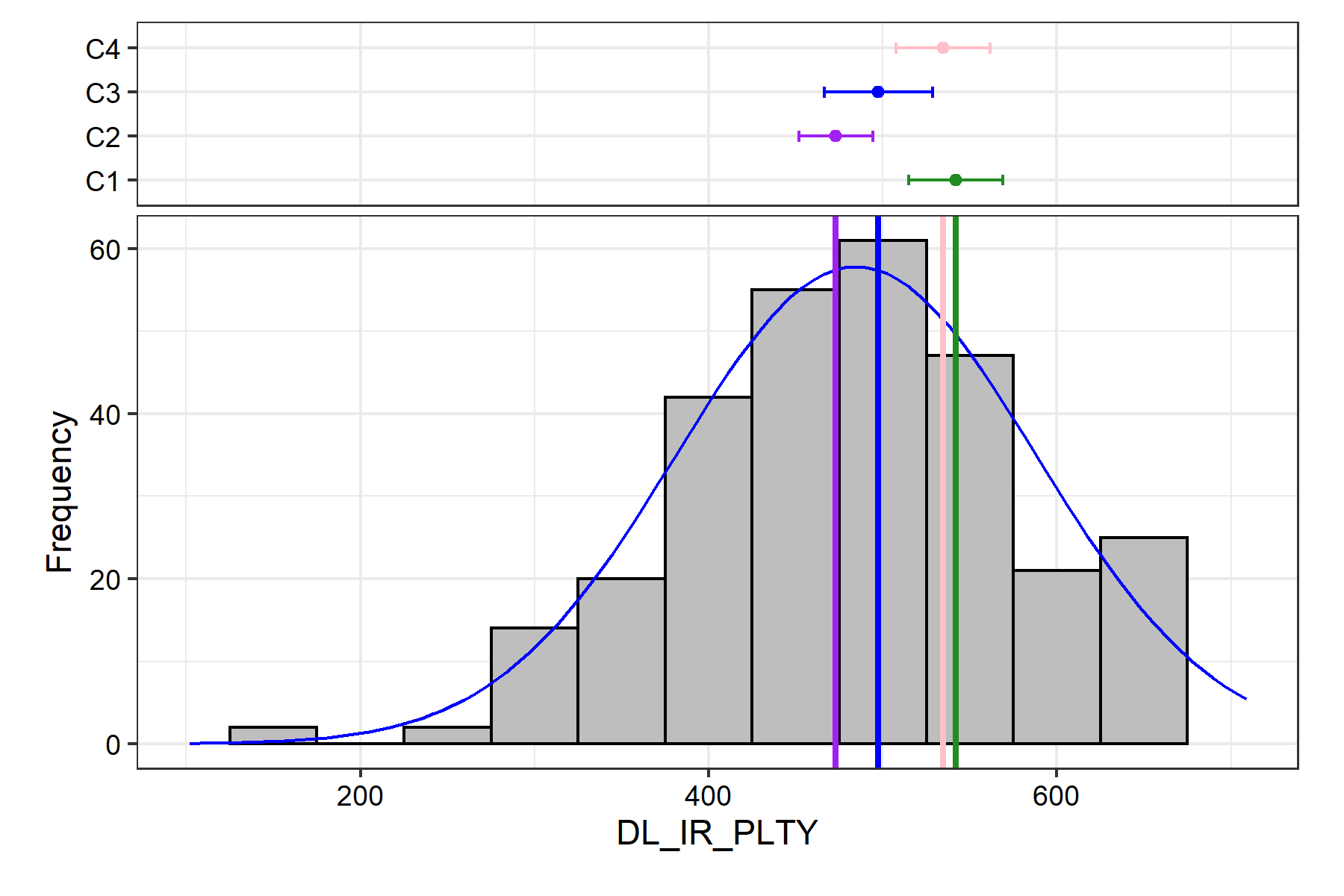

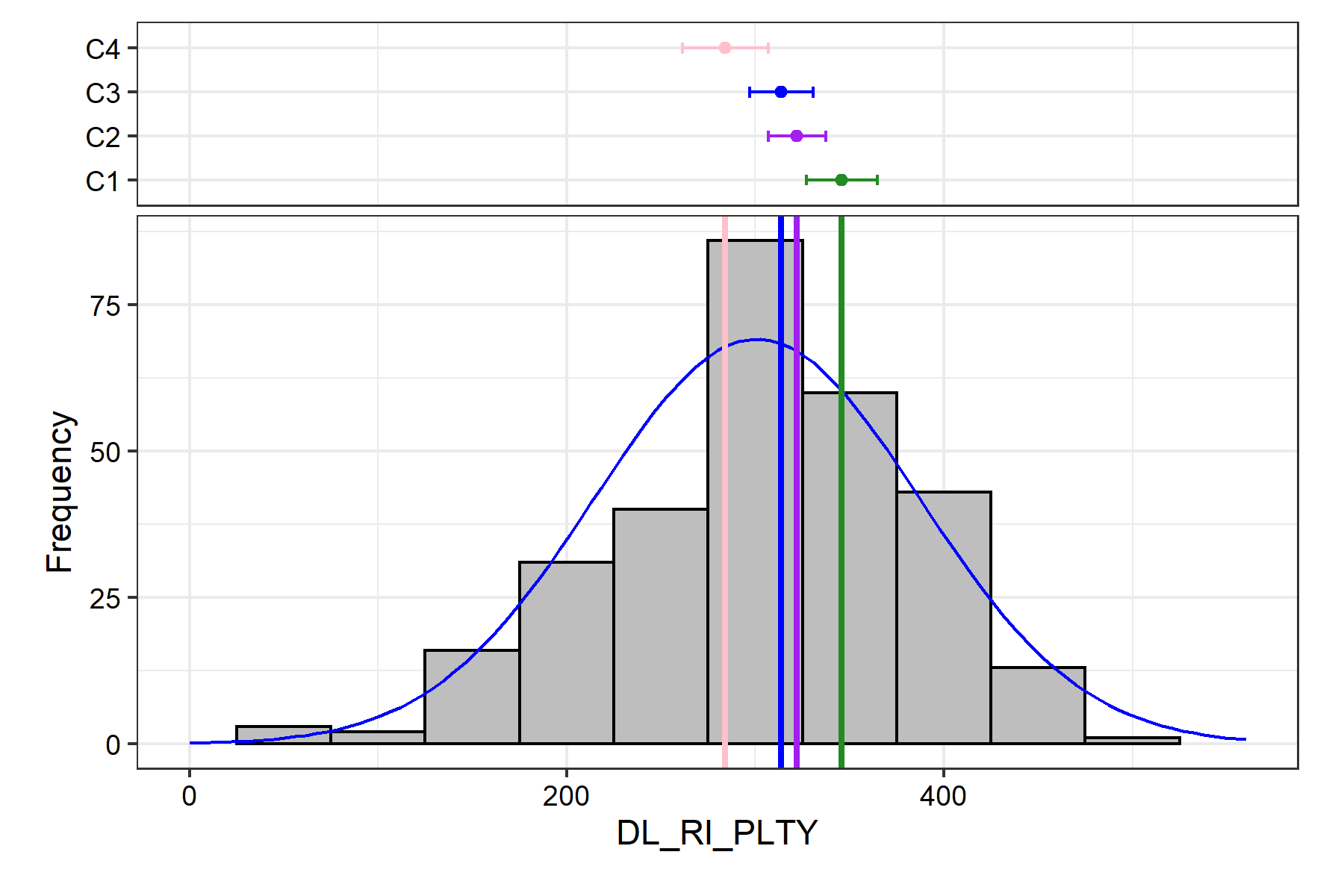

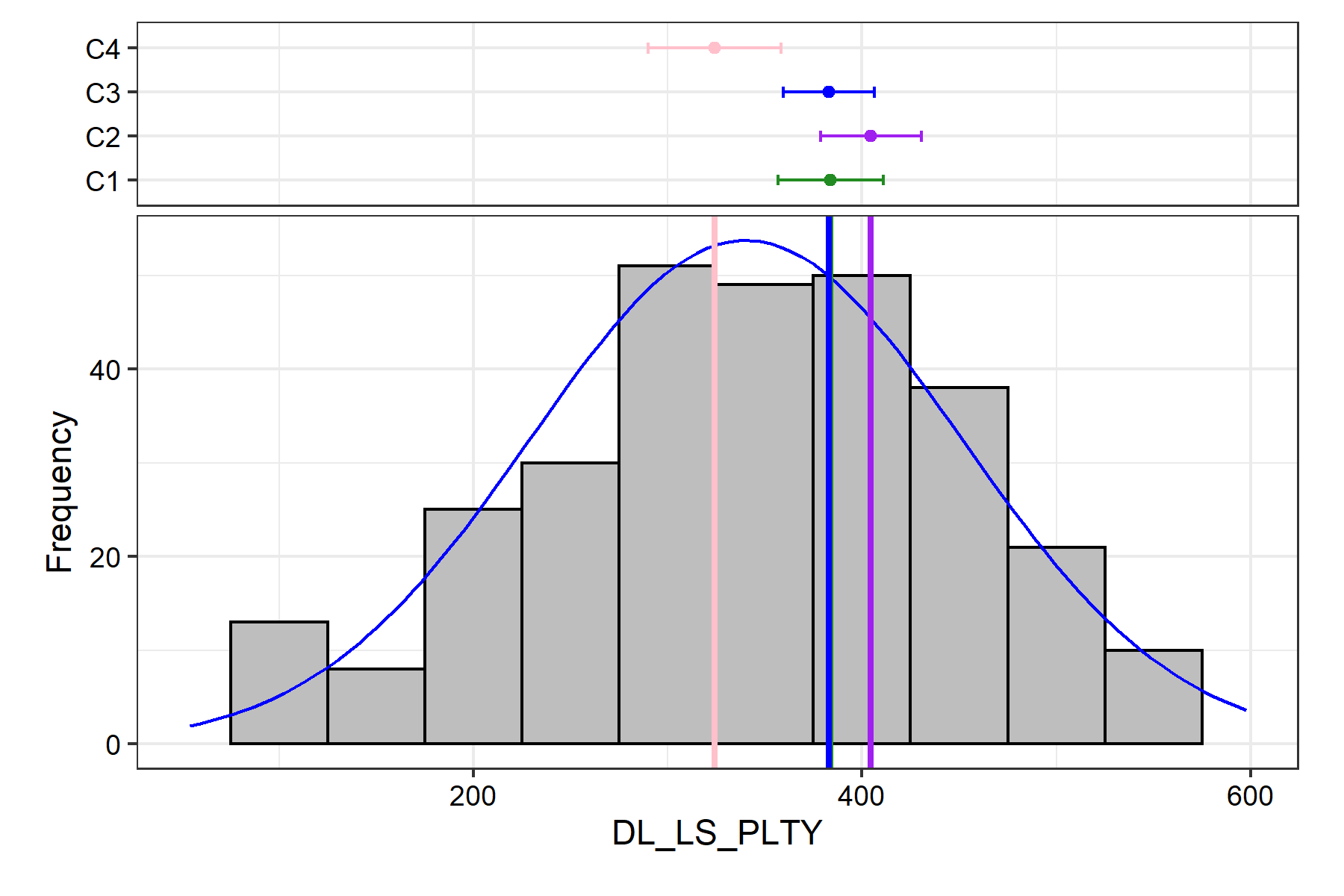

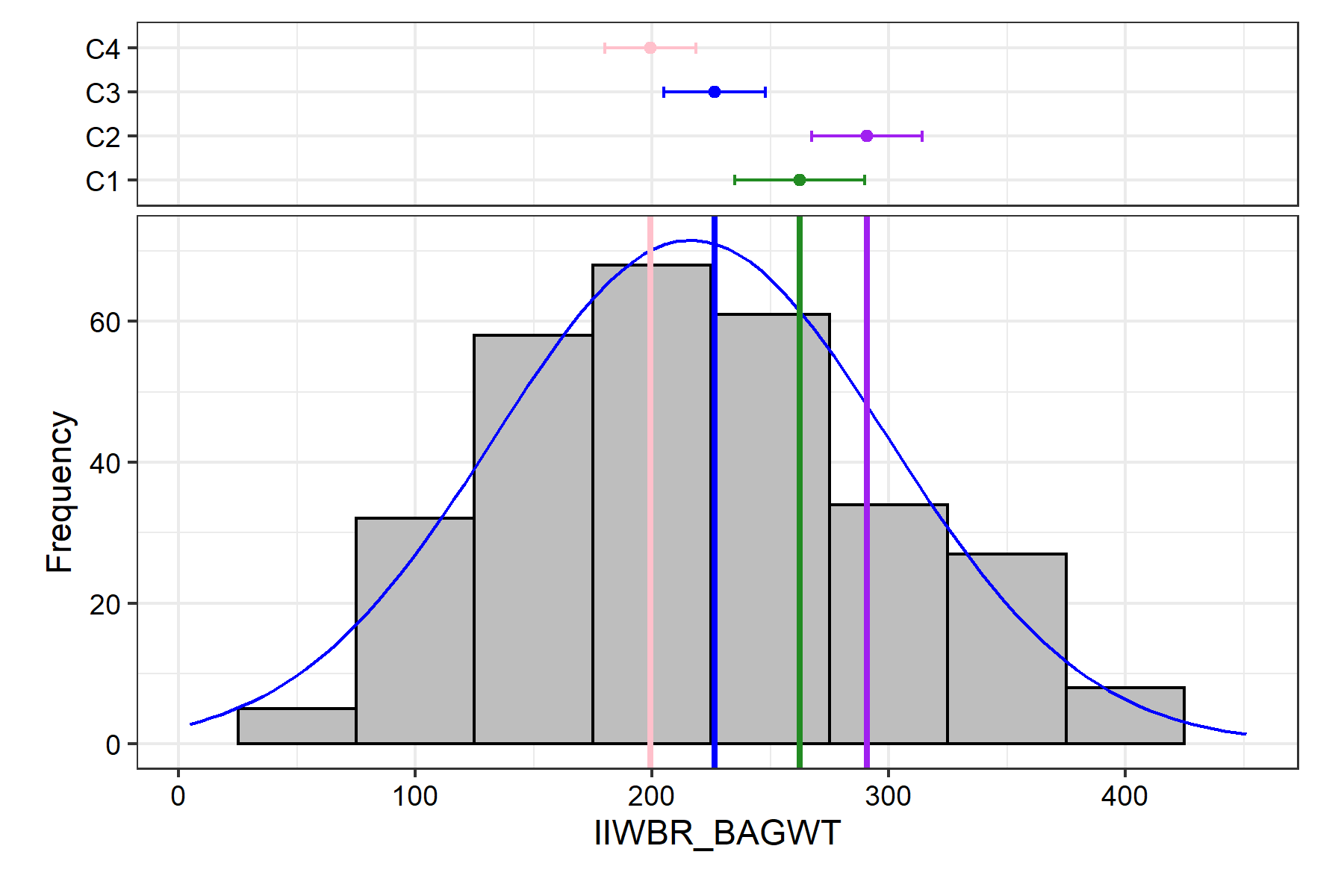

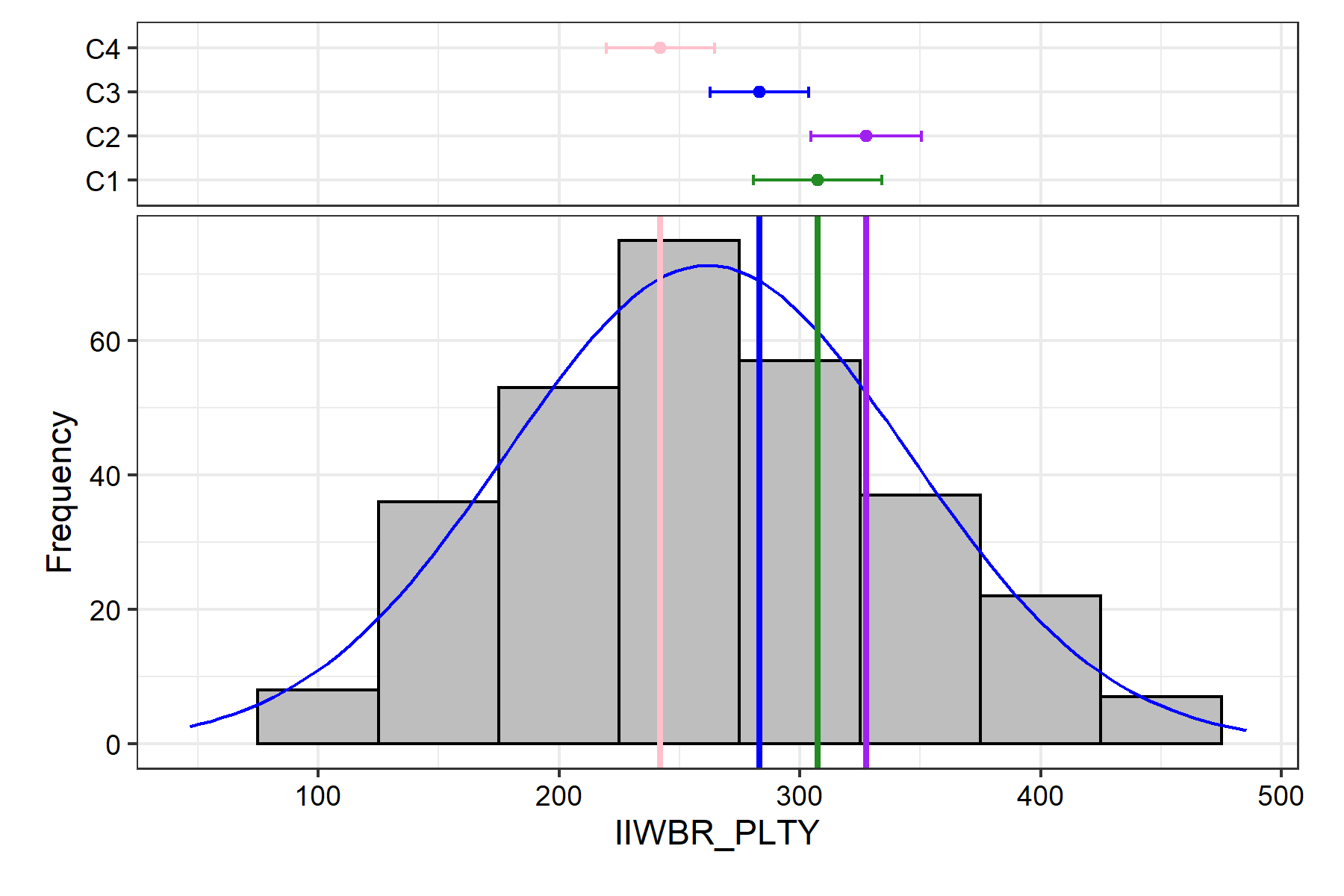

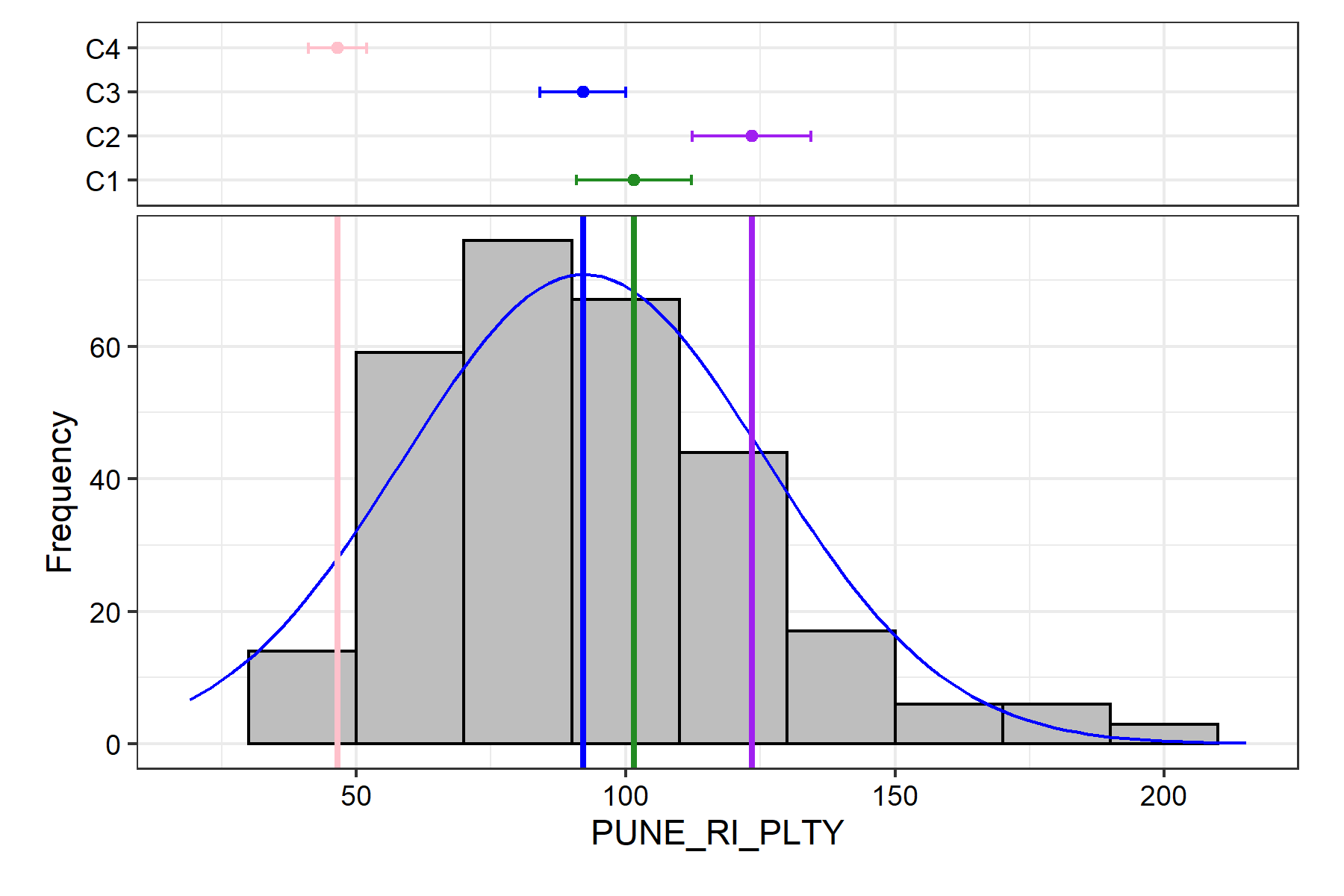

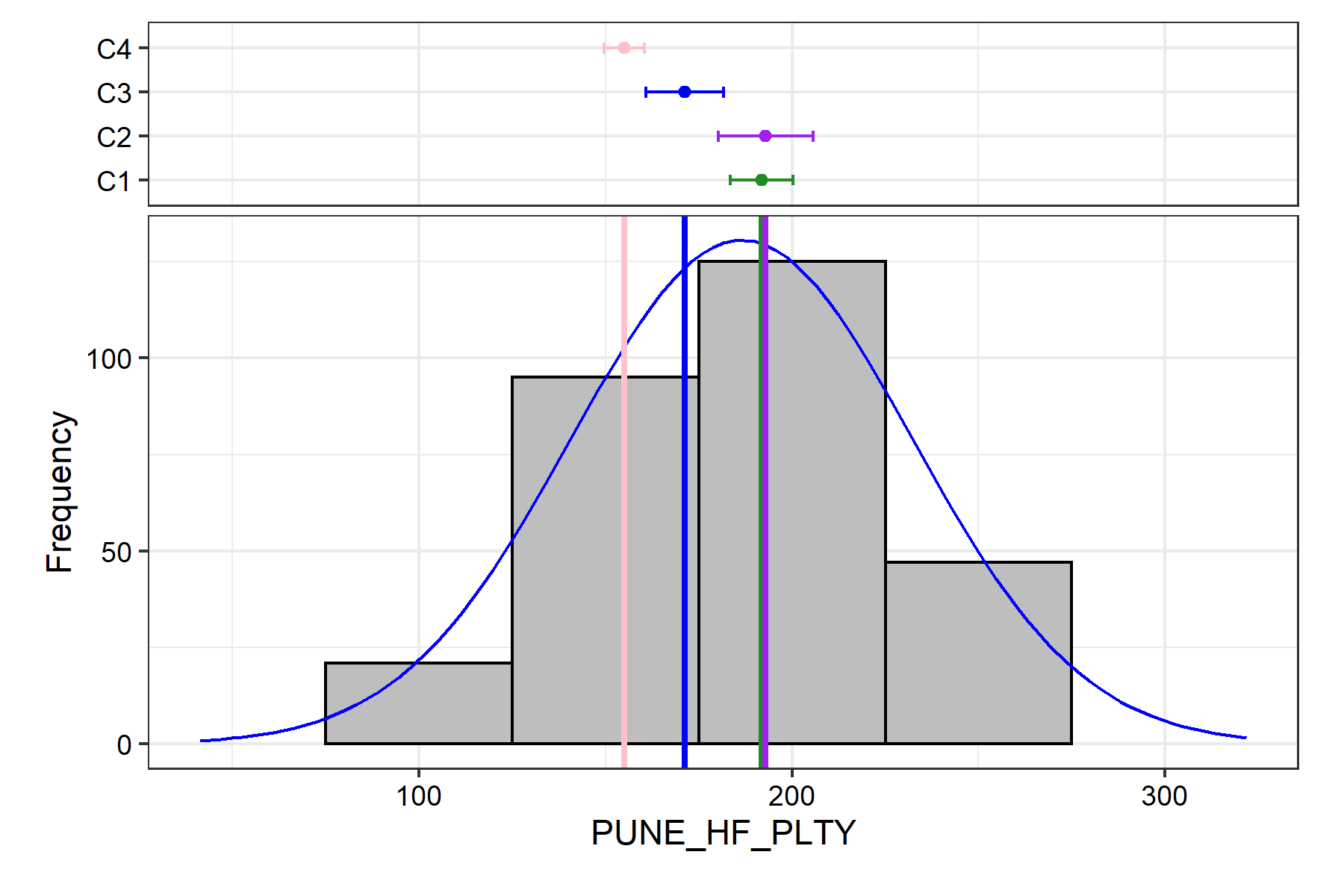

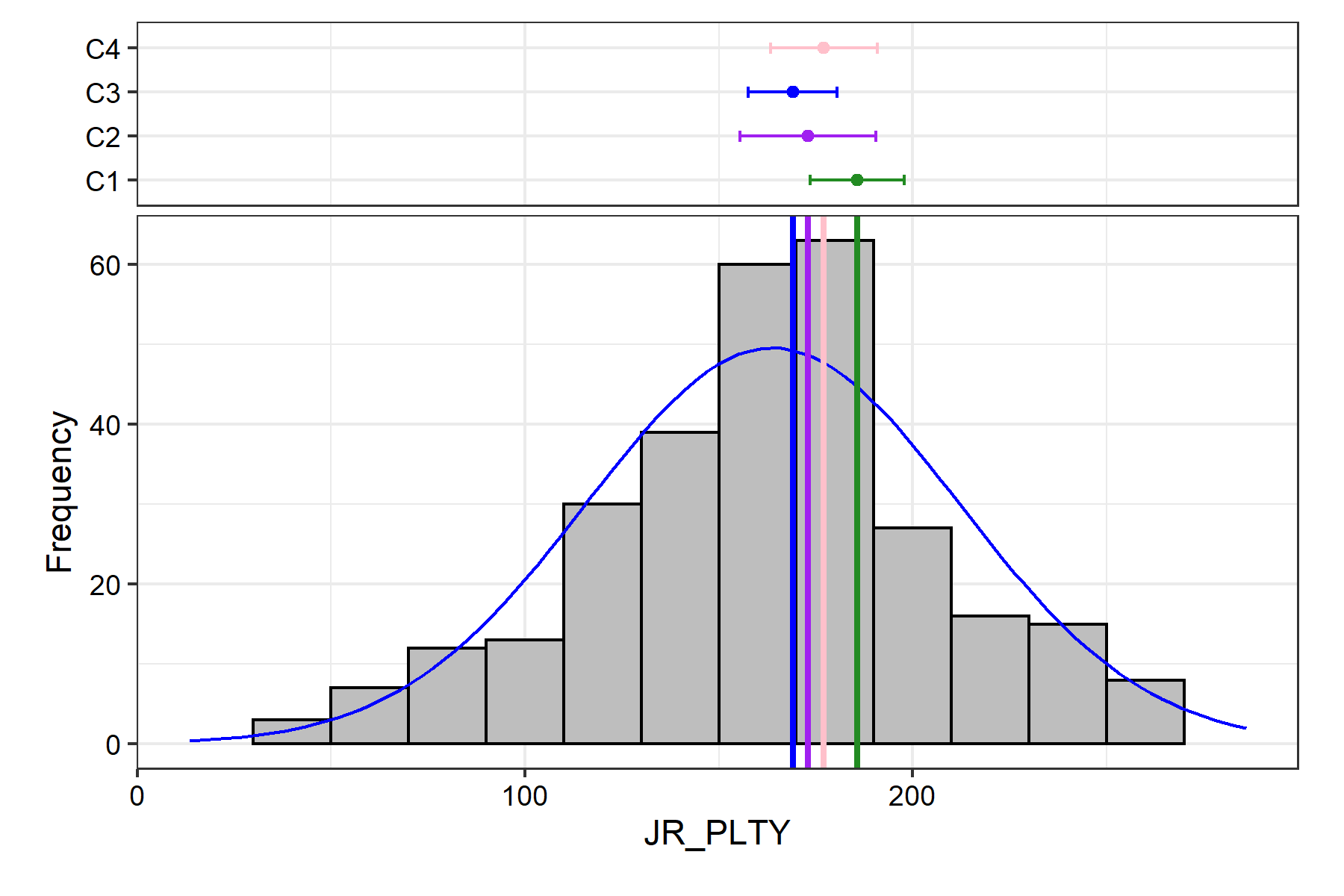

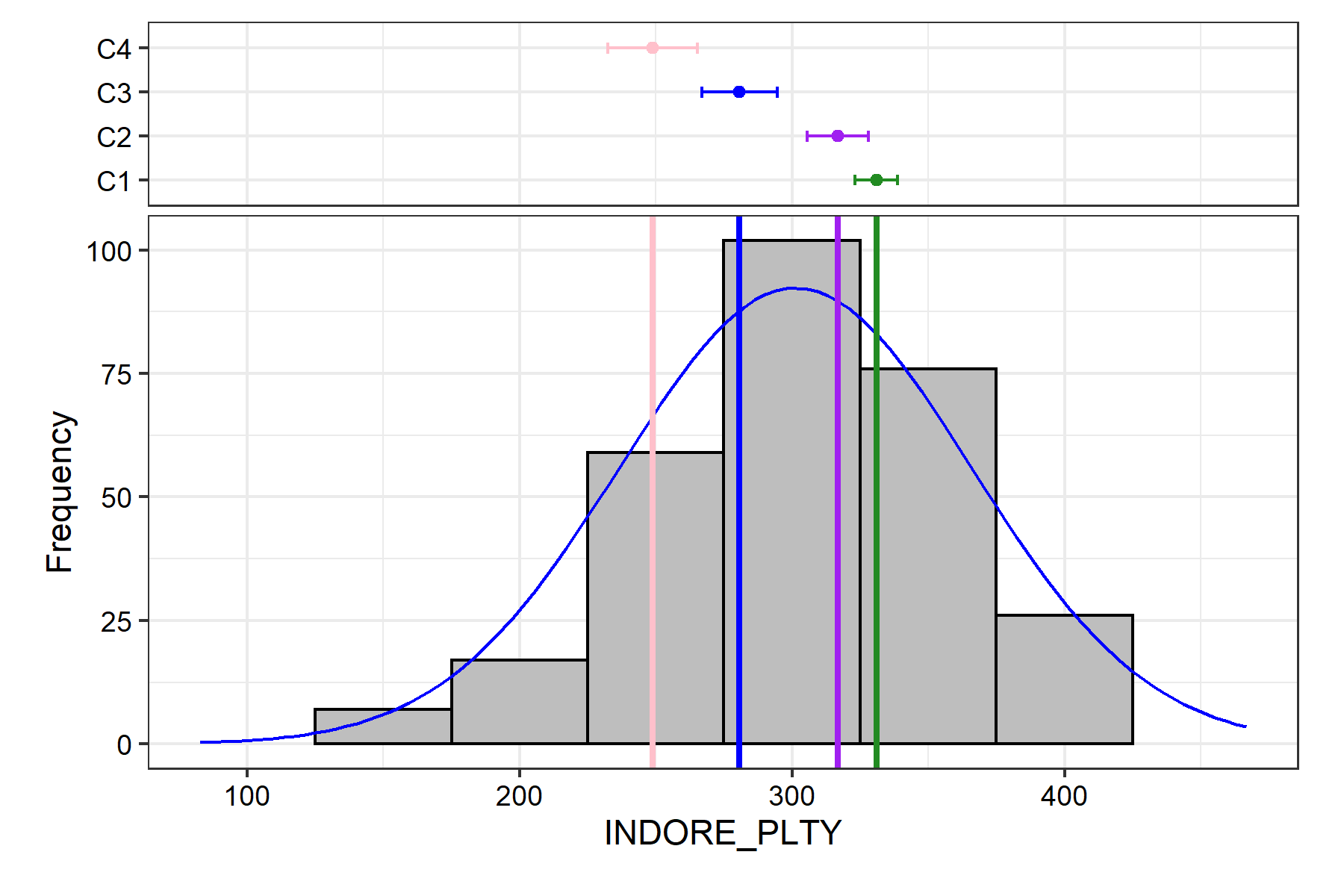

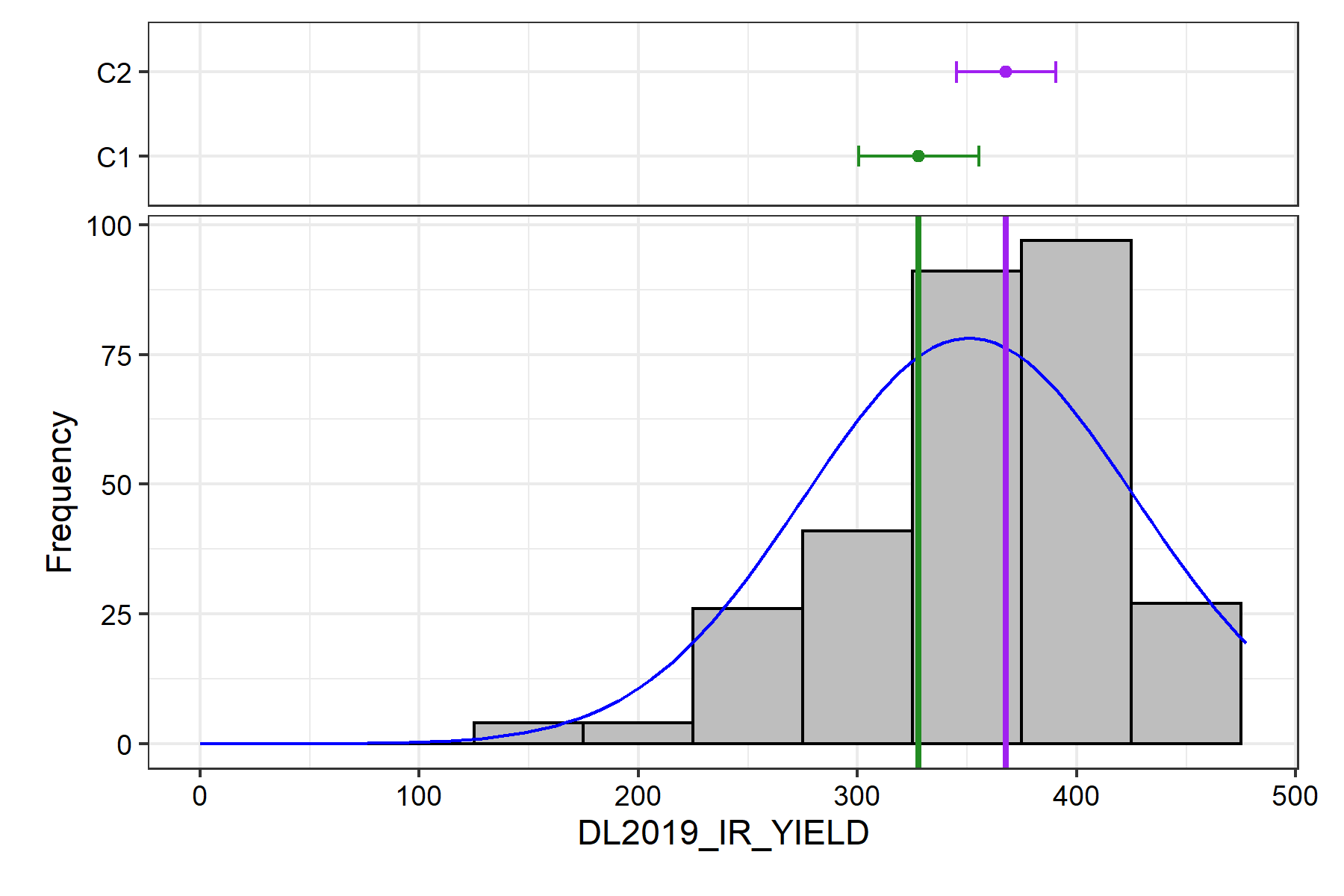

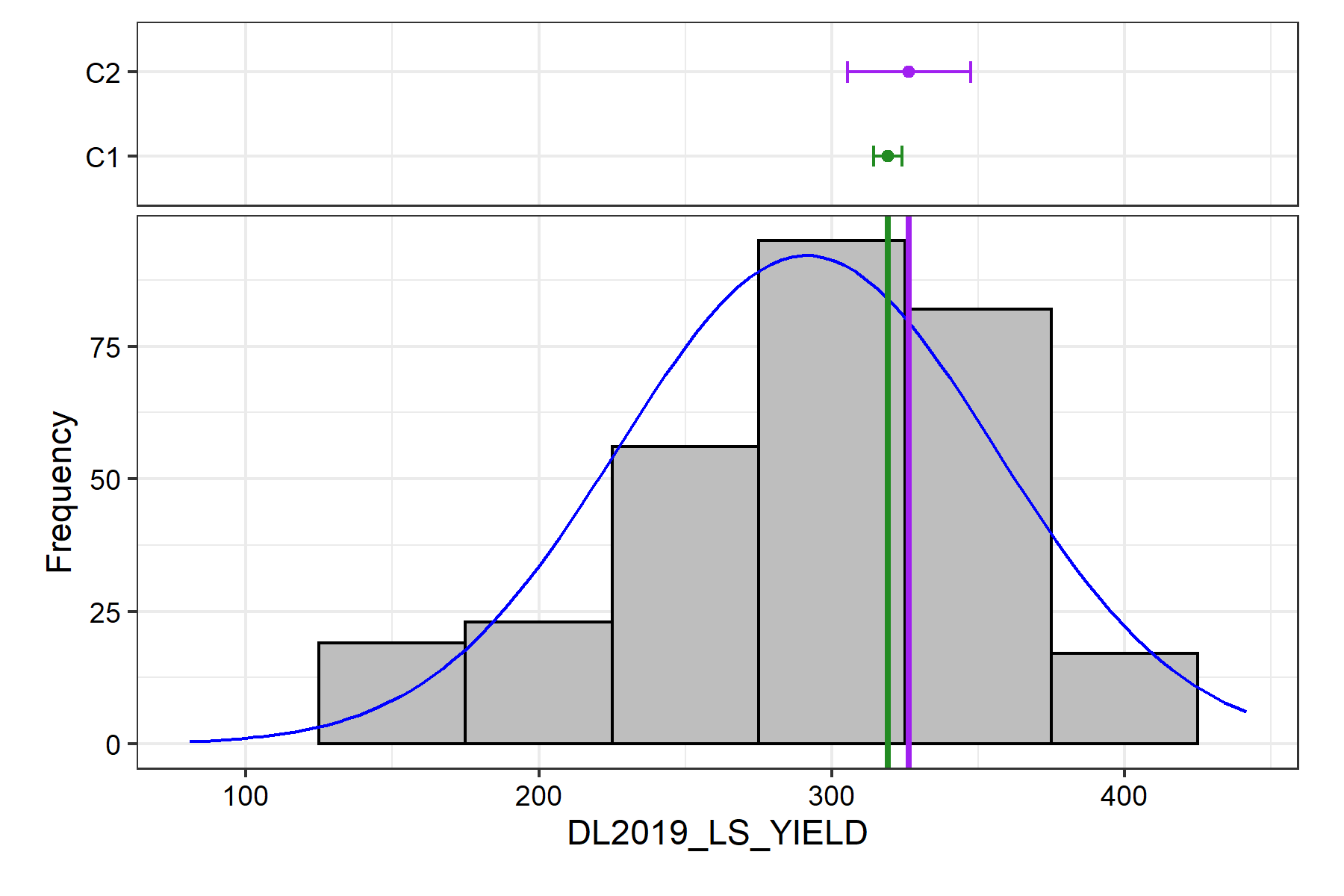


BMS


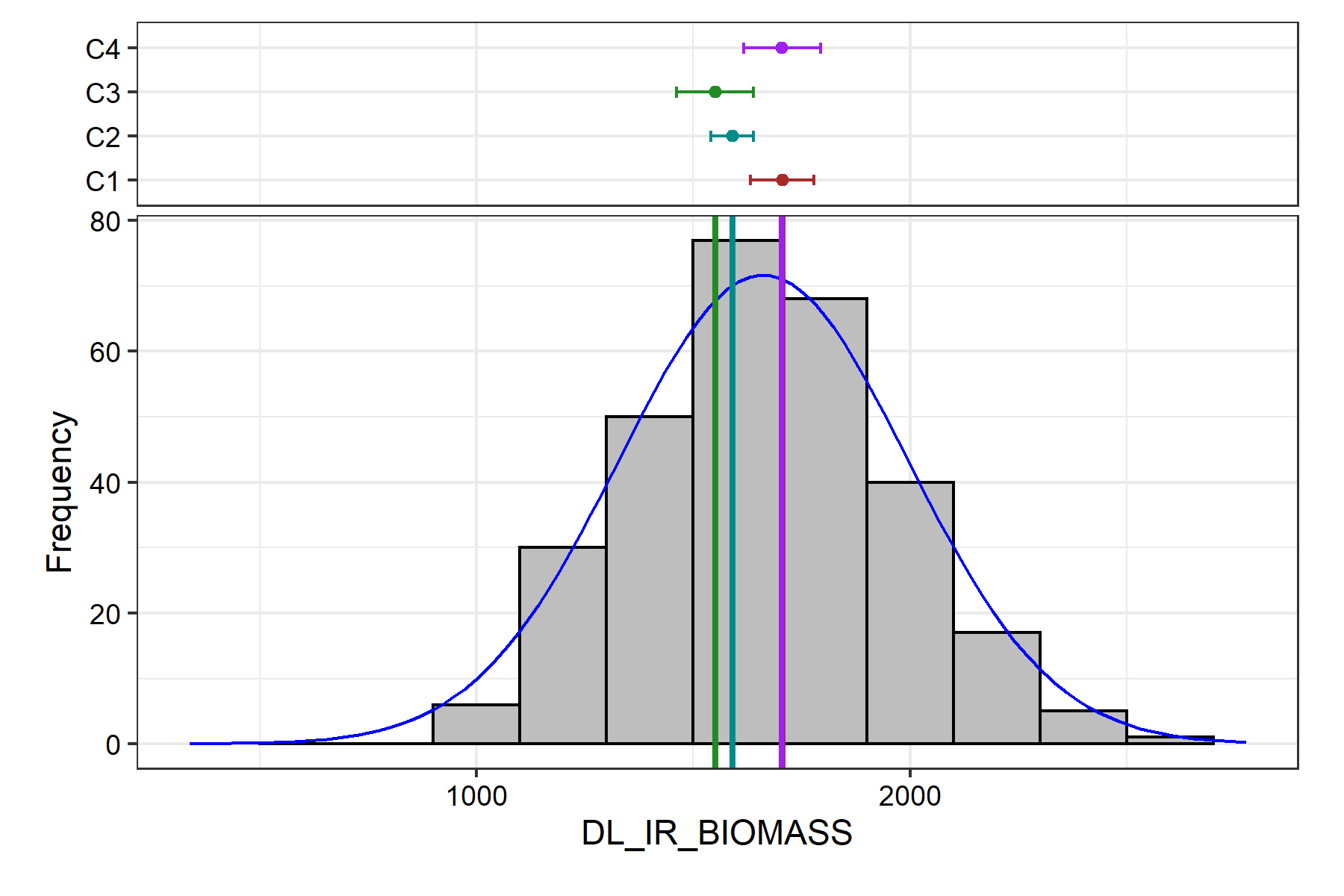

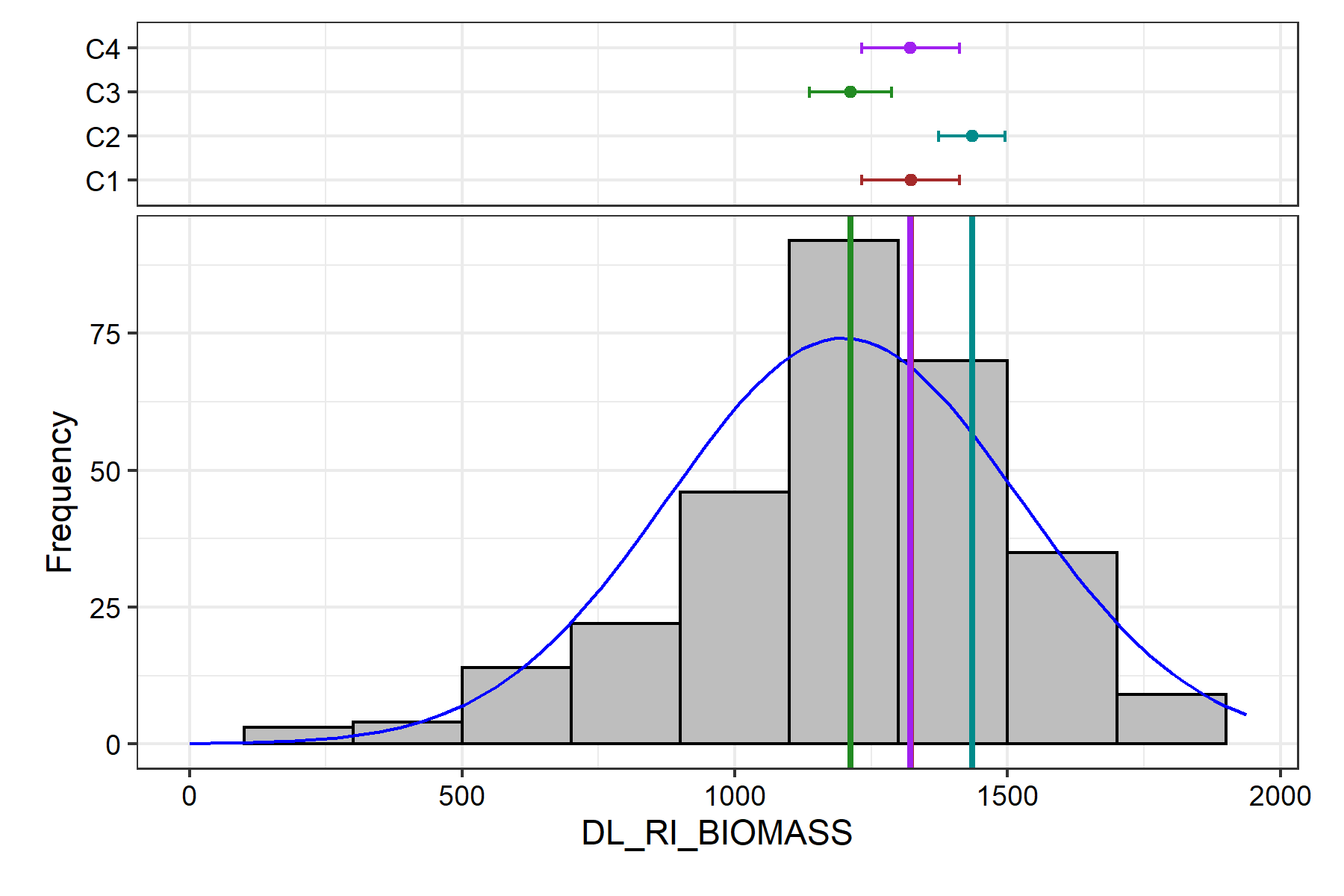

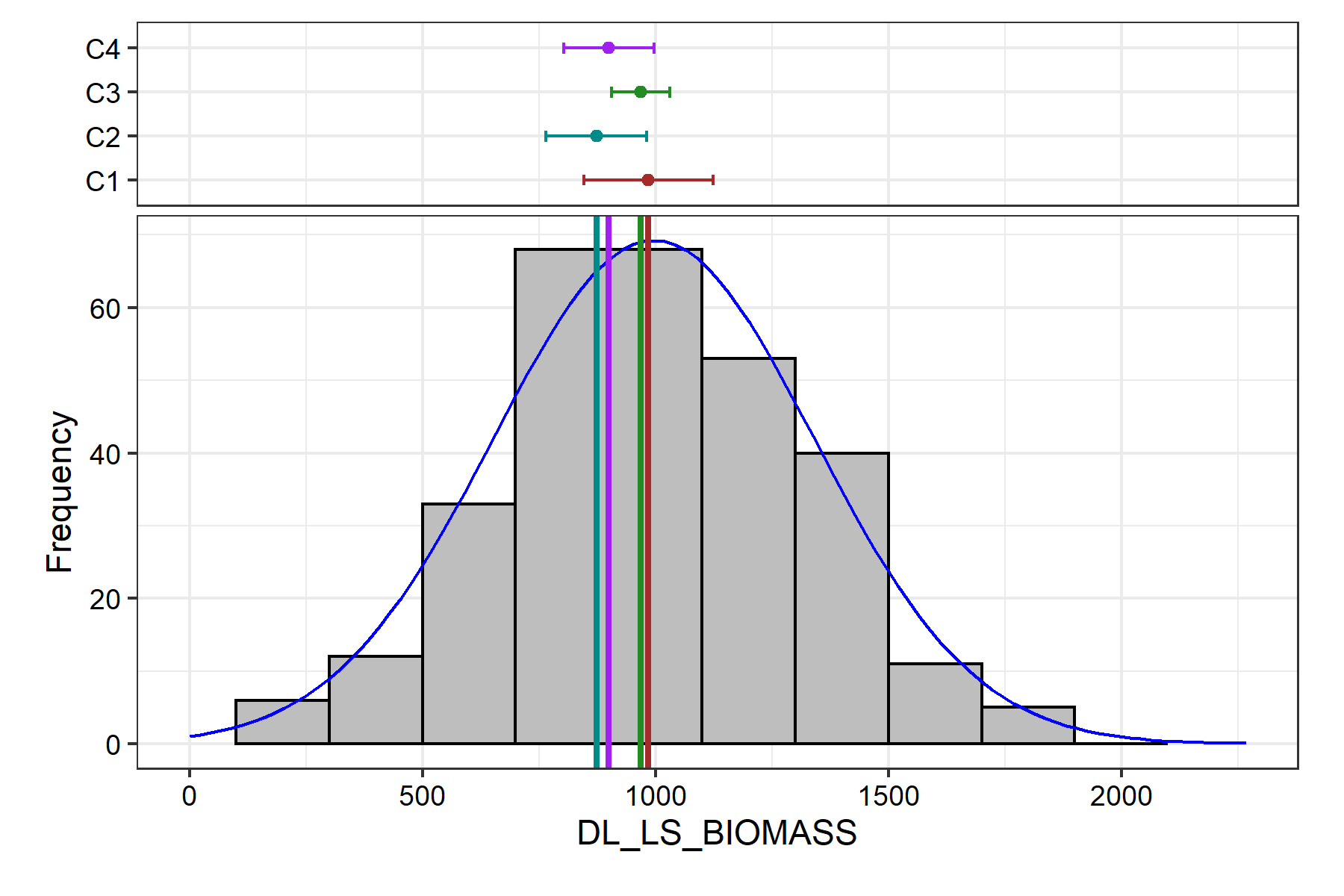

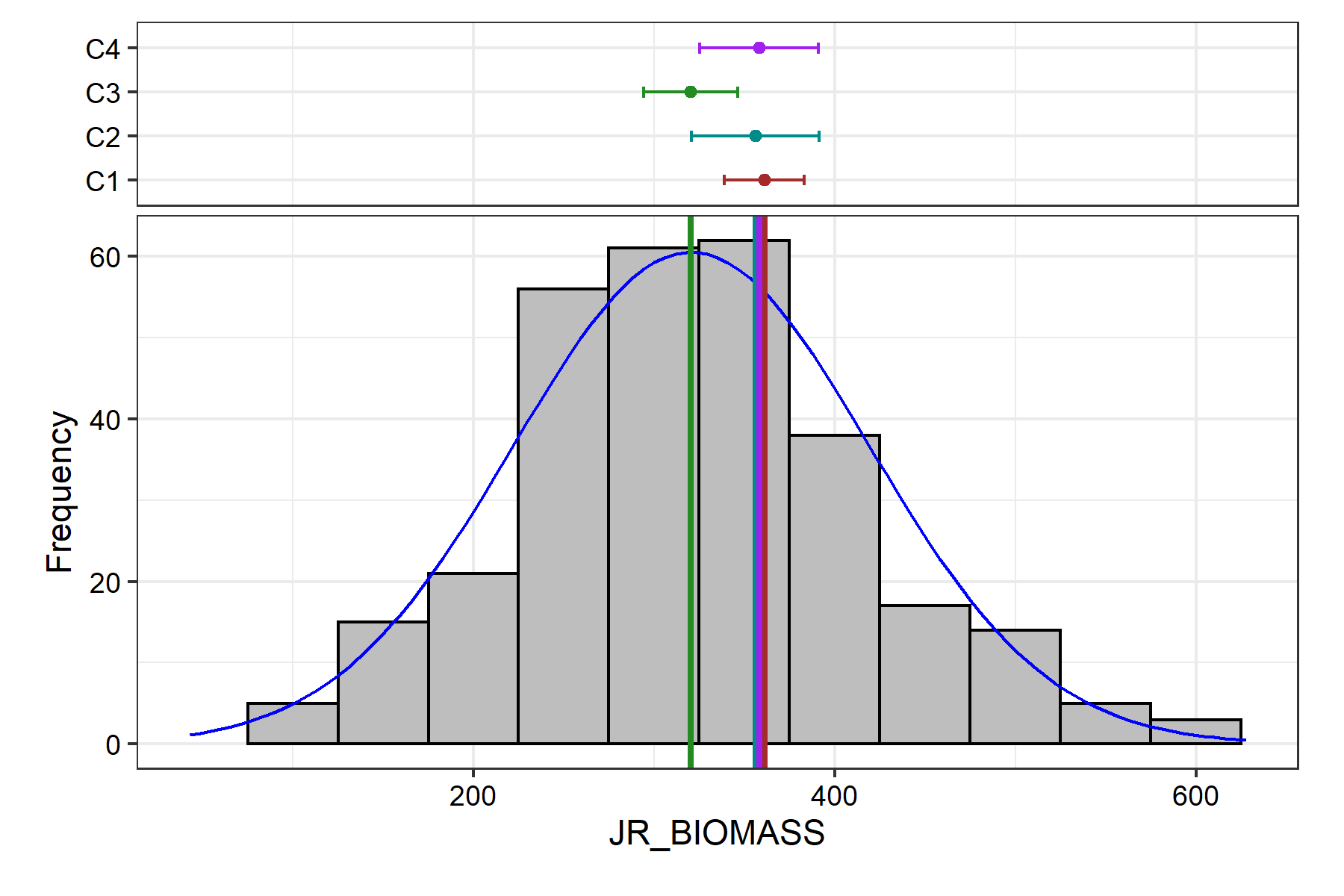


DM


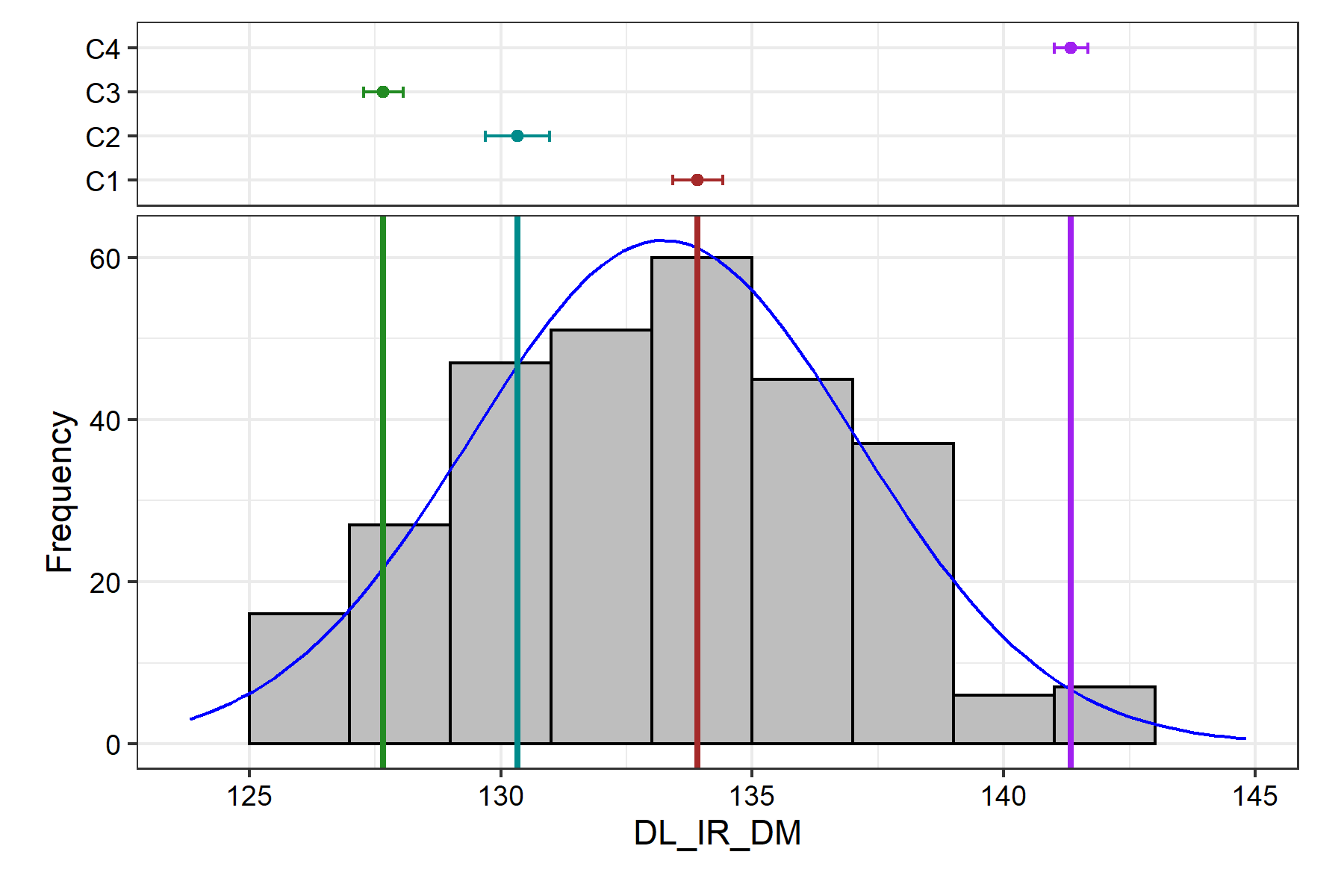

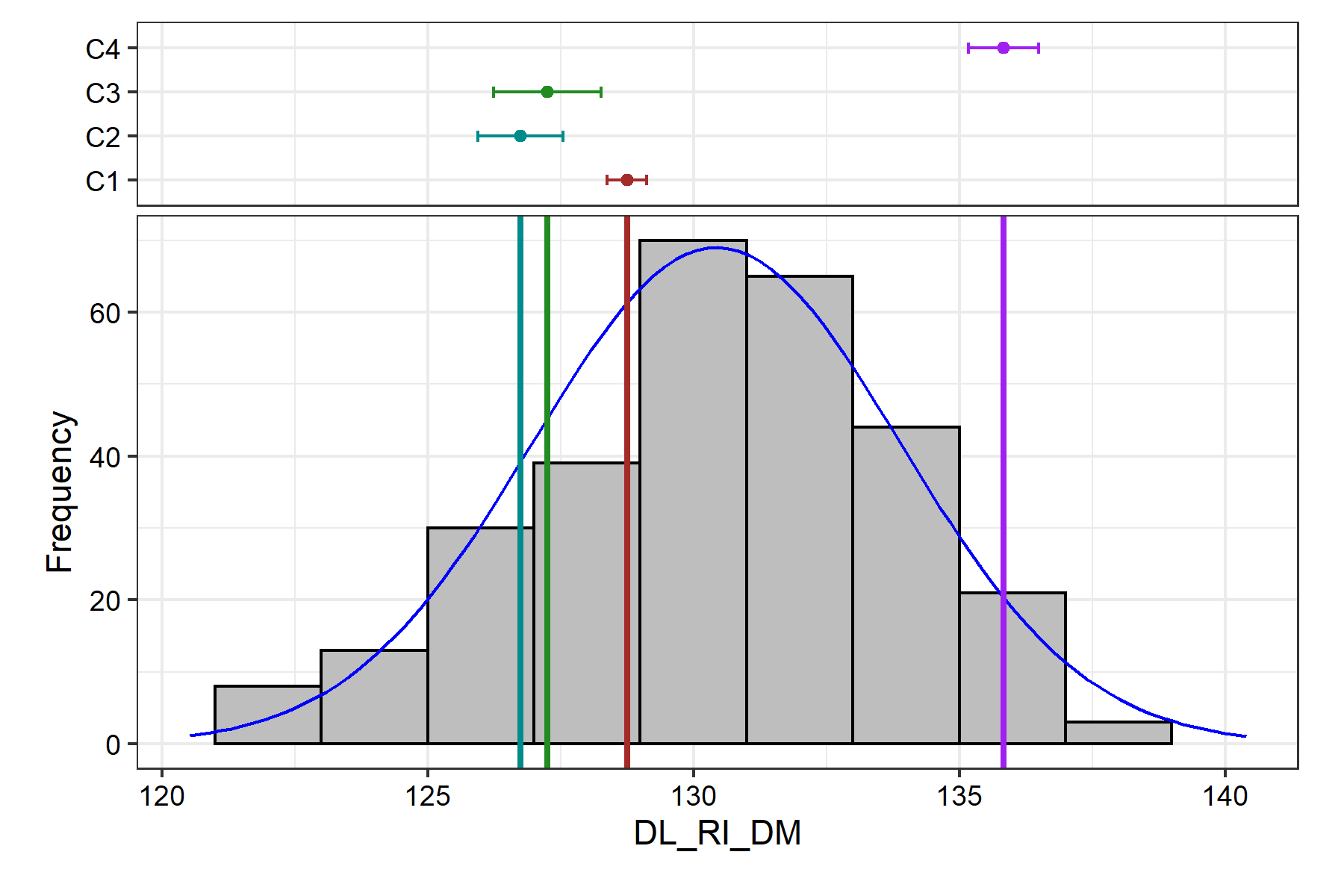

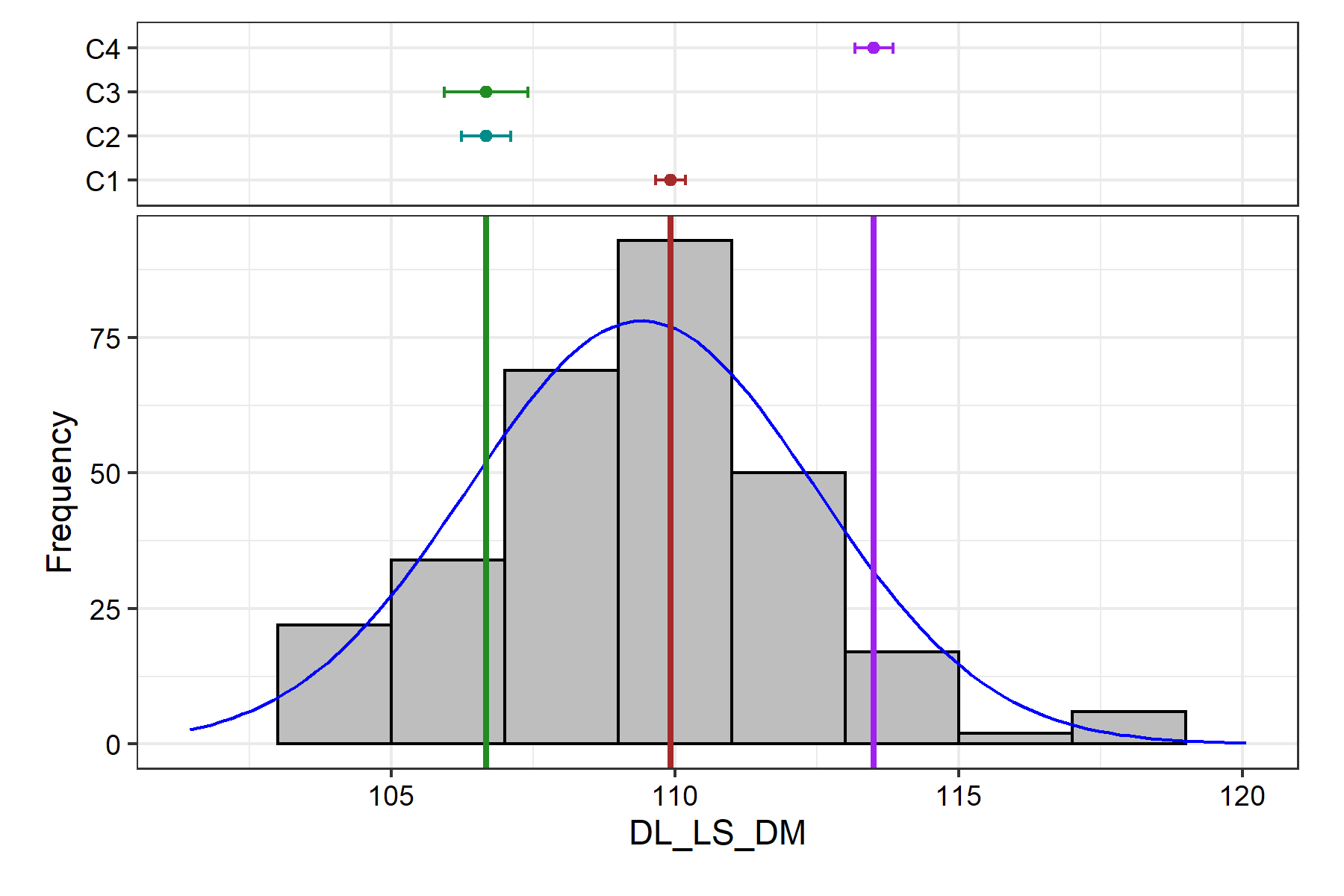

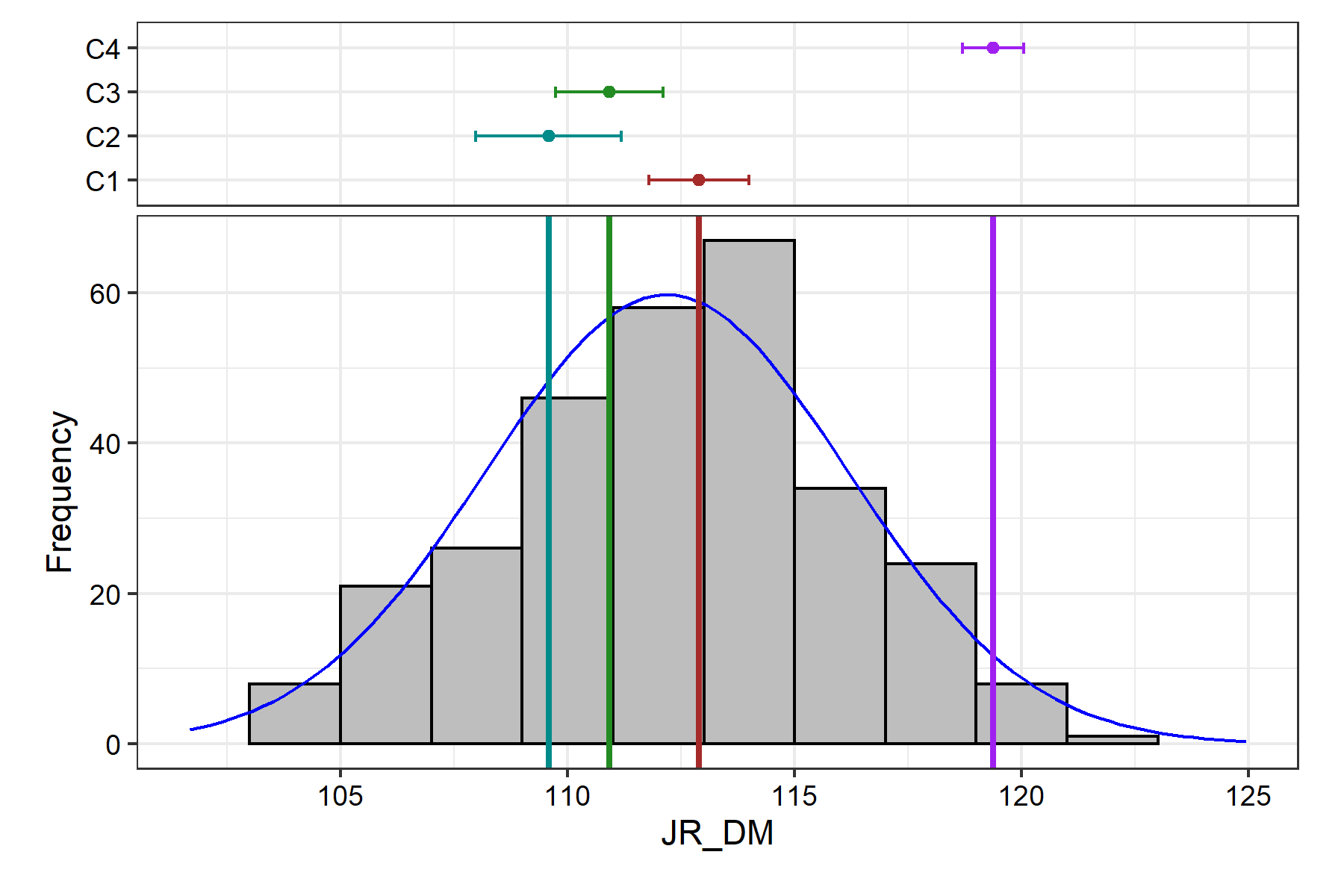

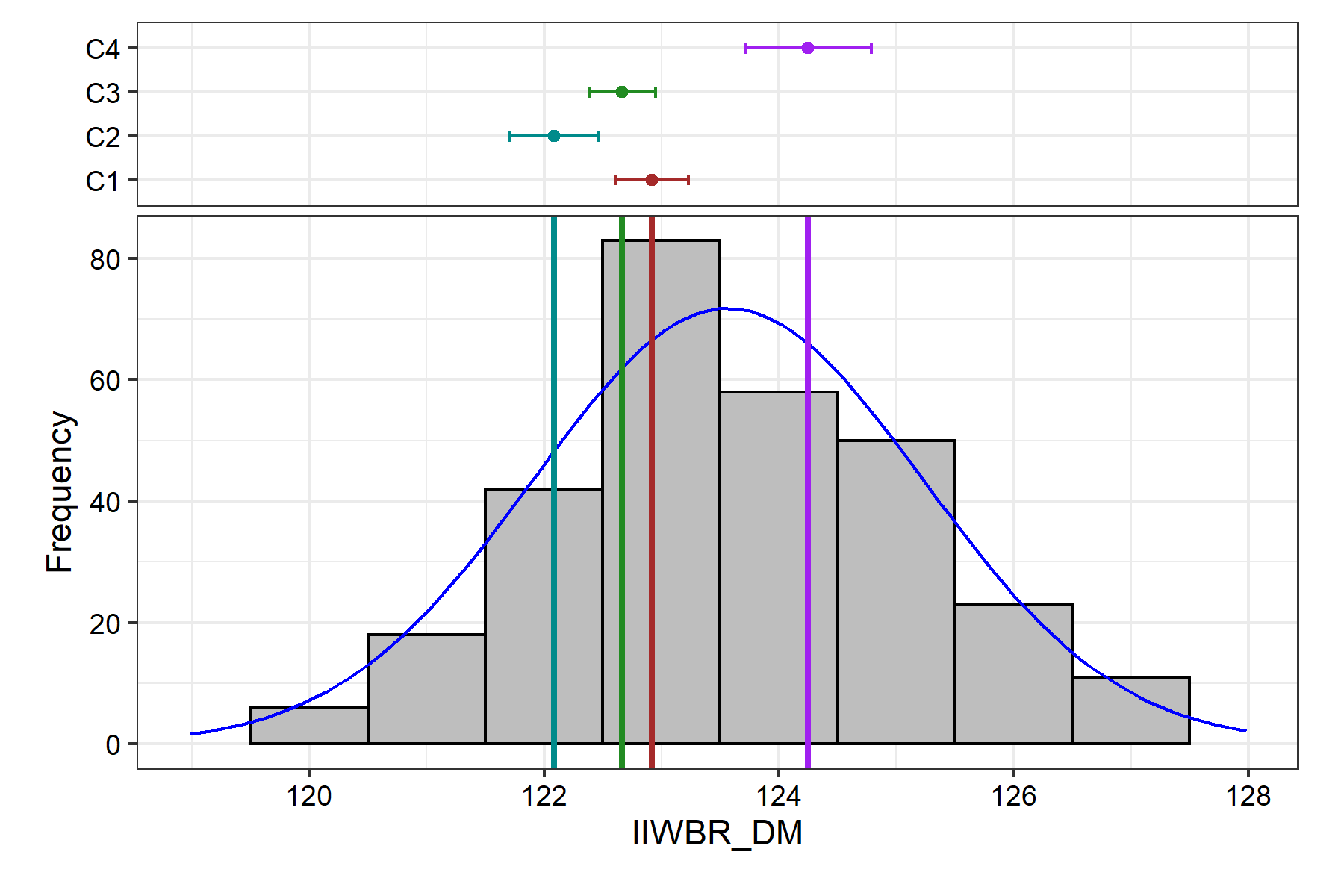


NDVI


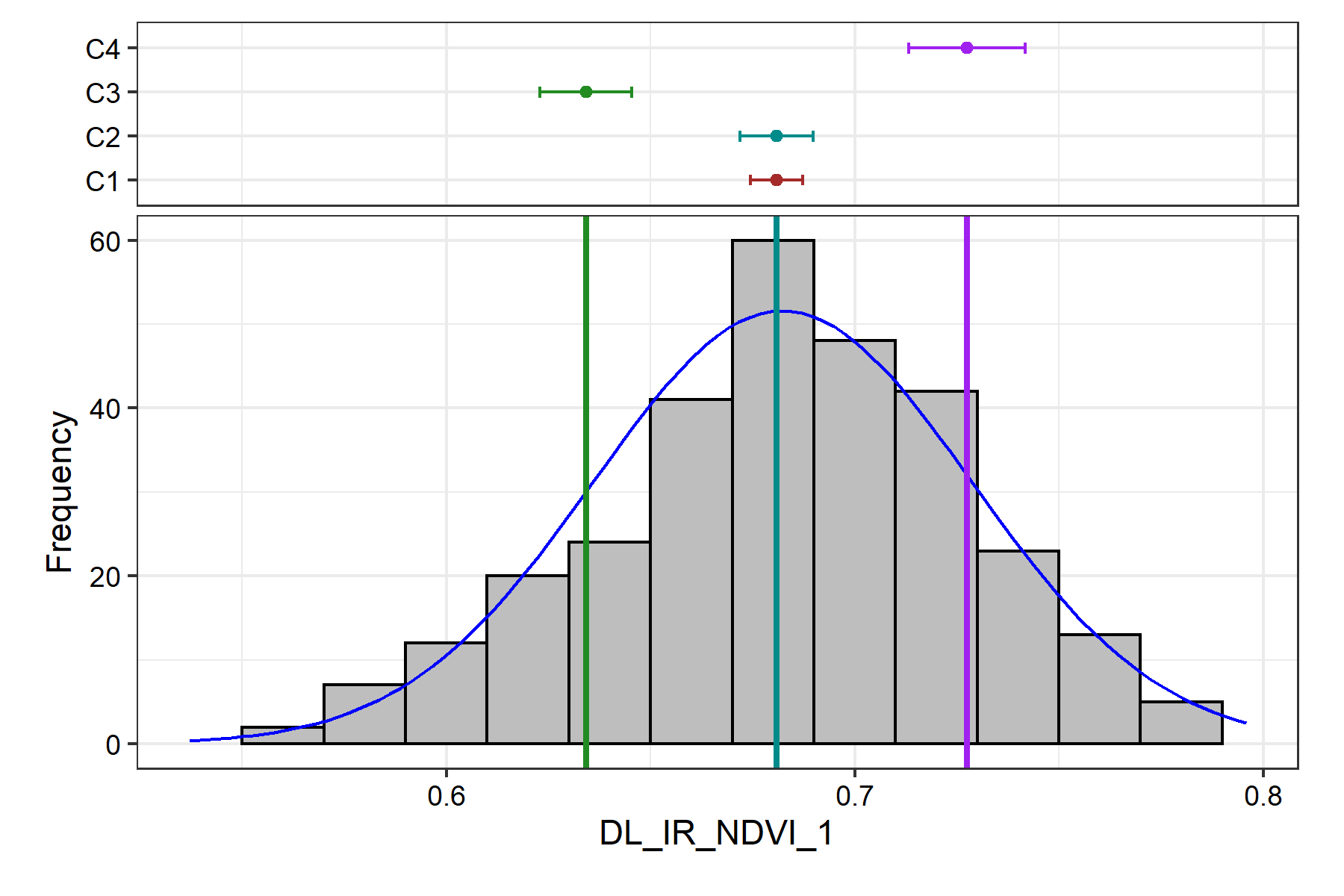

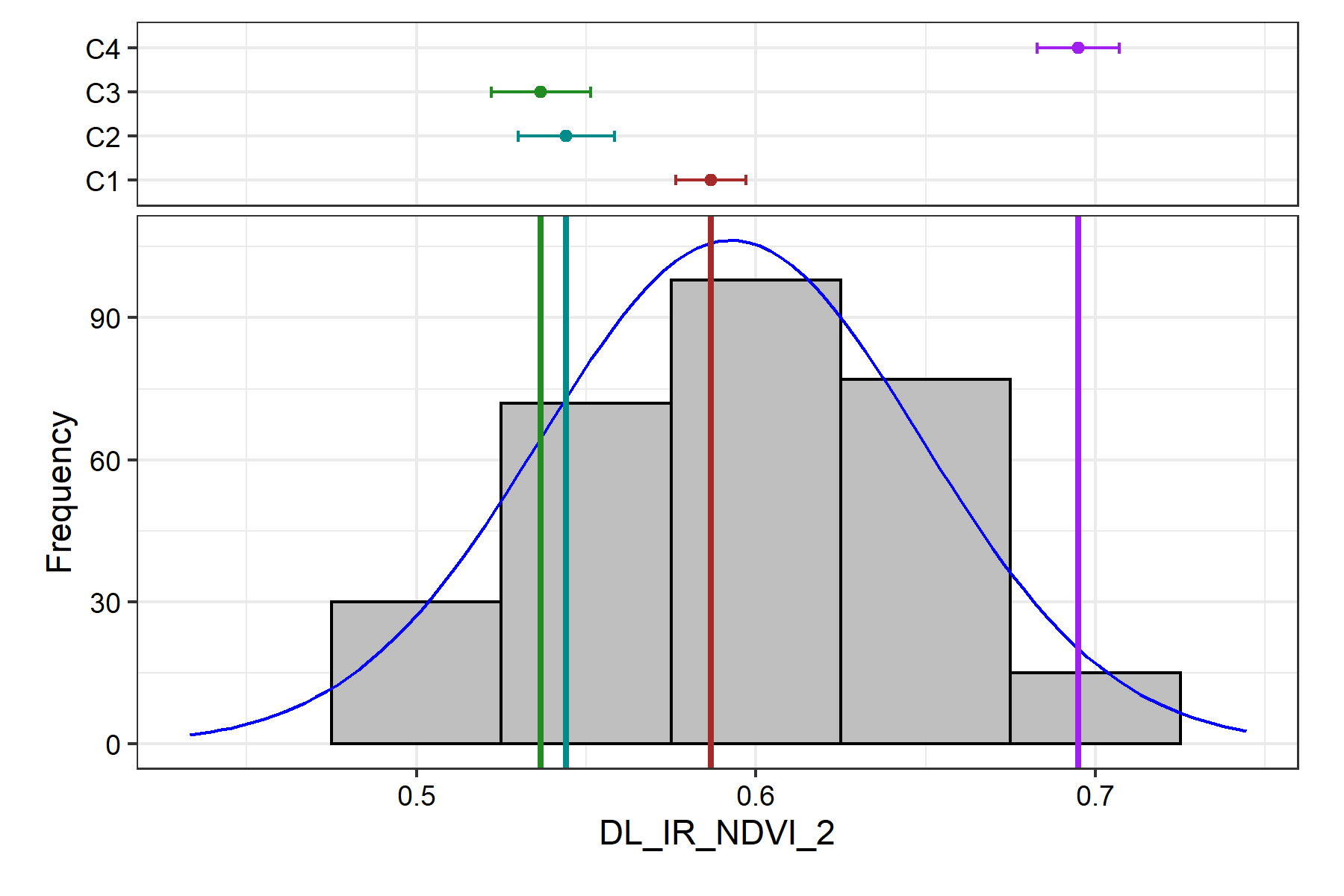

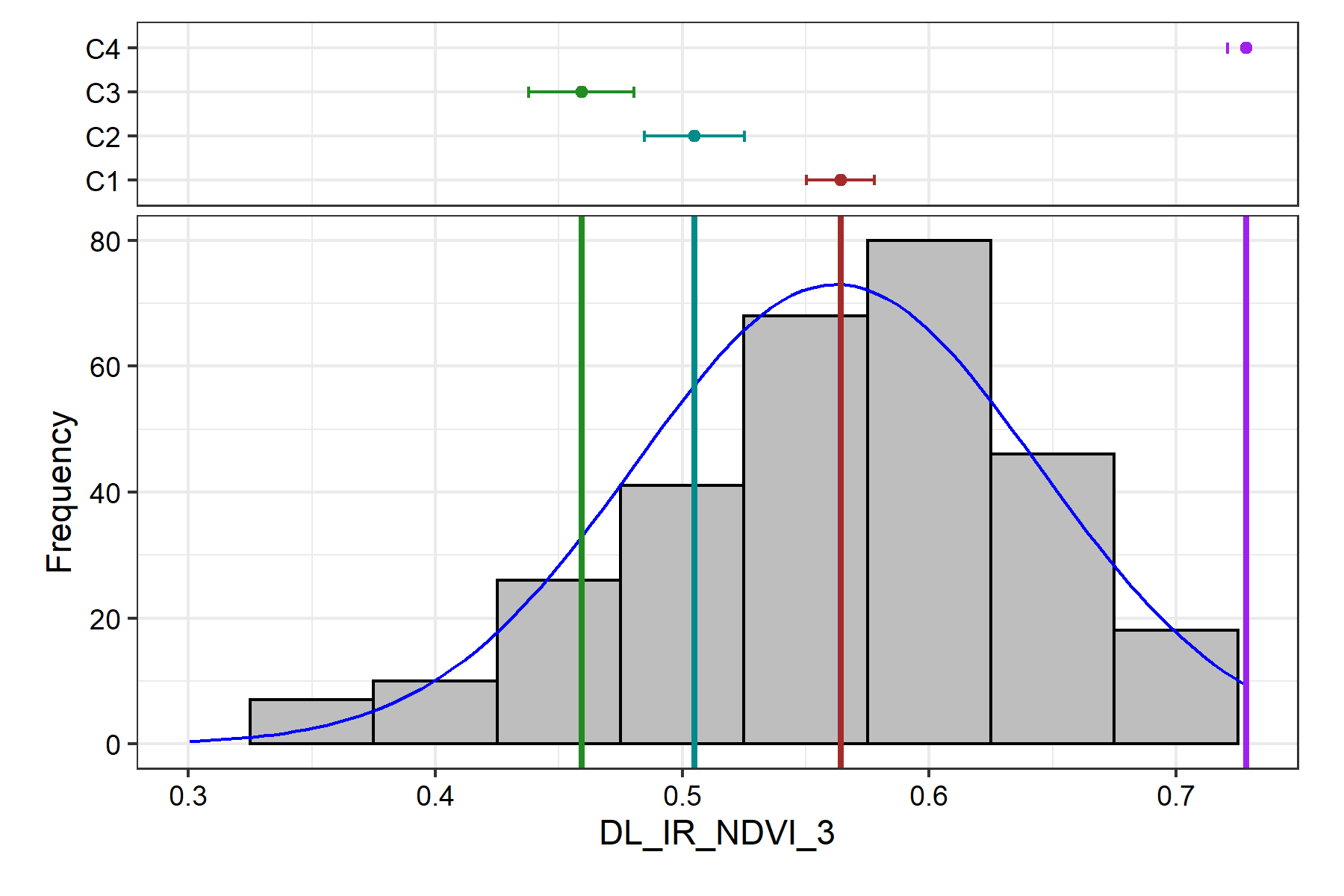

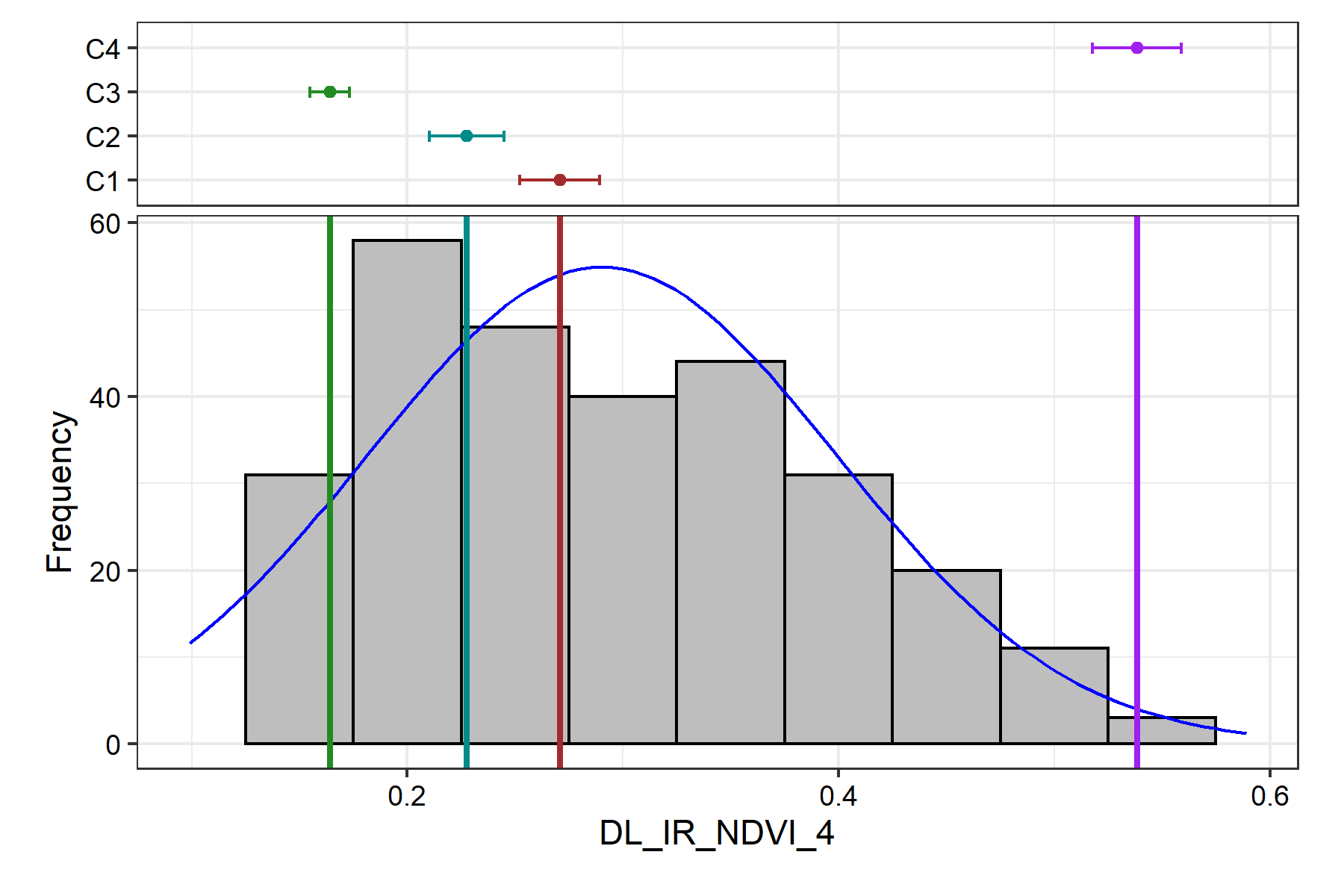

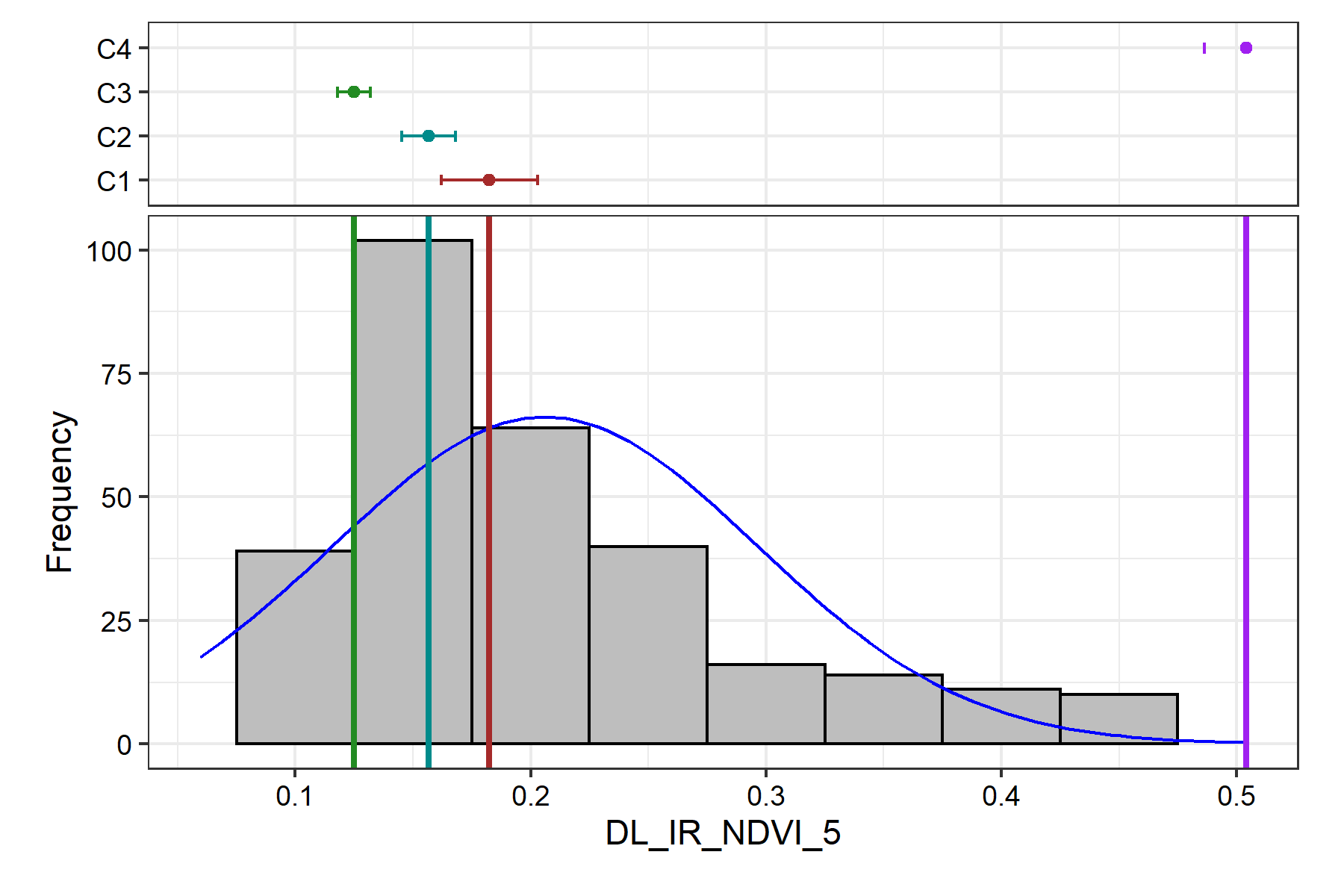

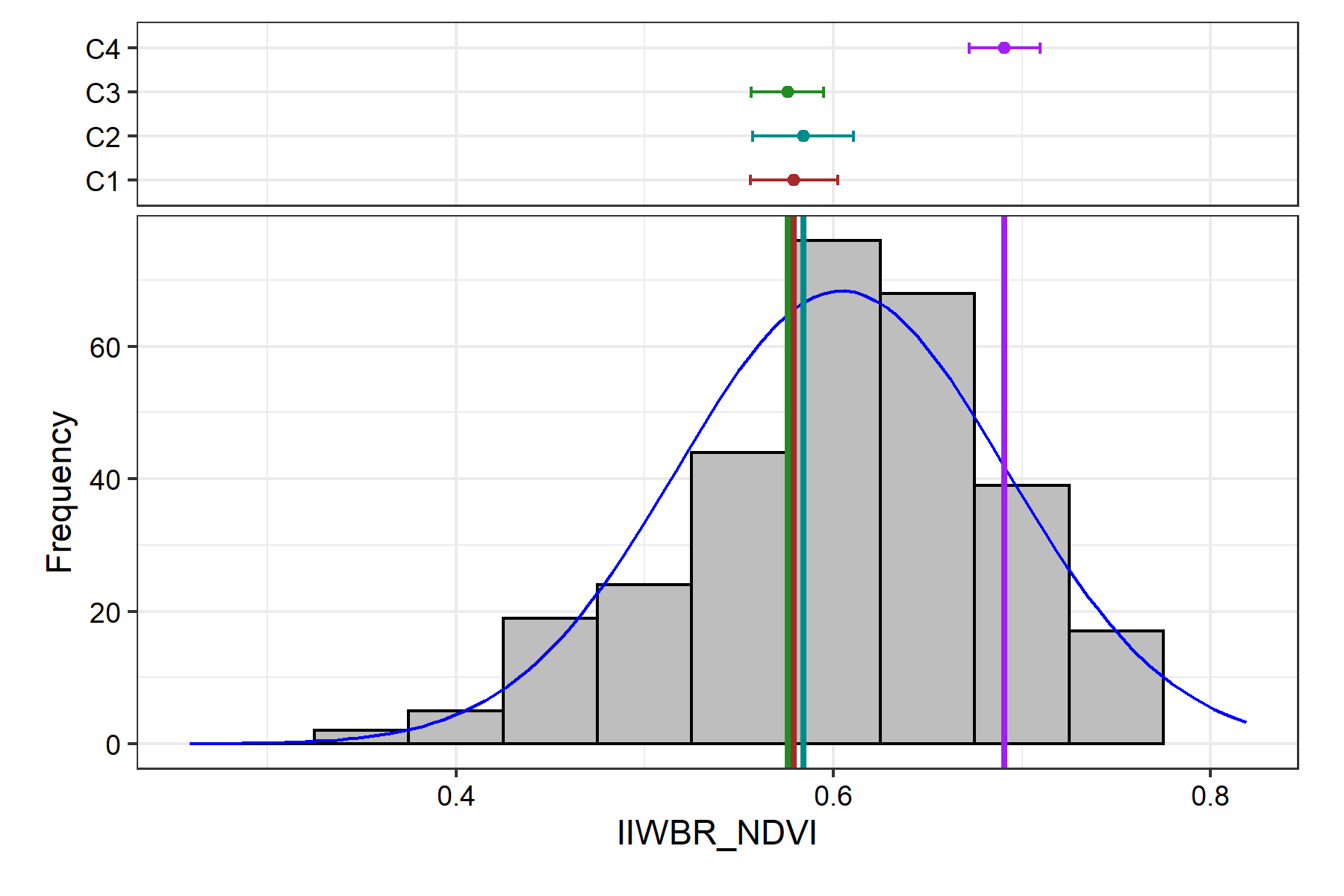


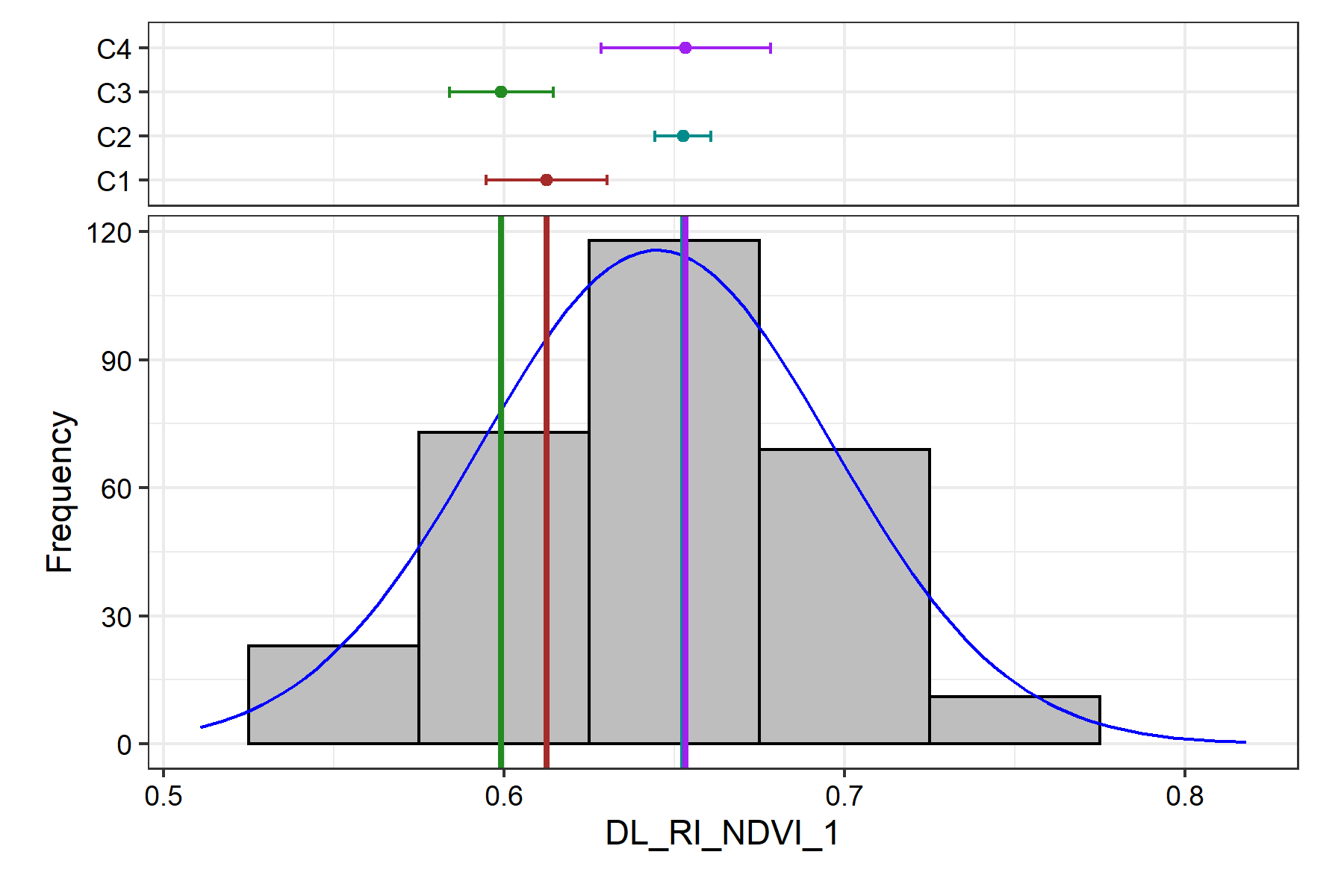

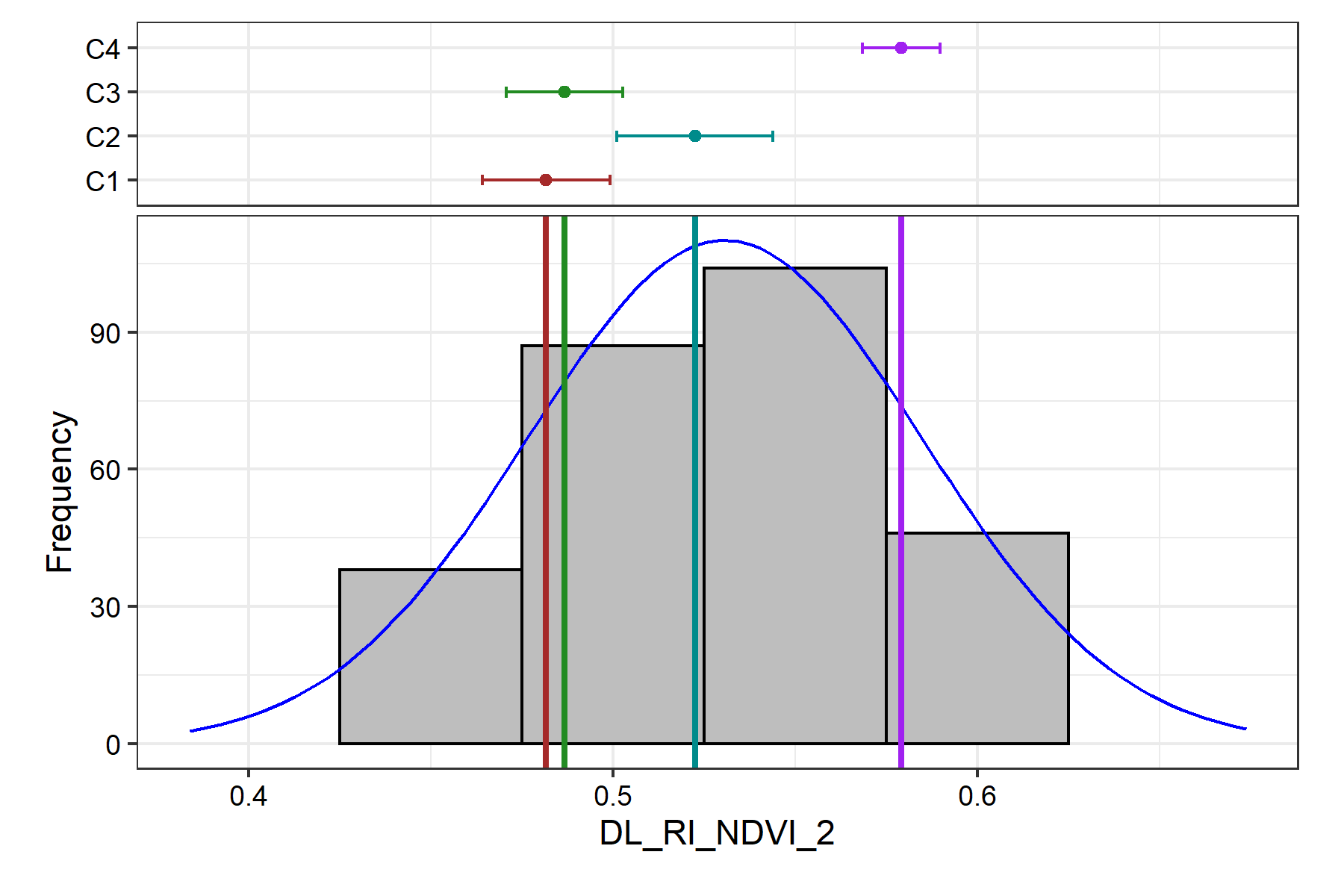

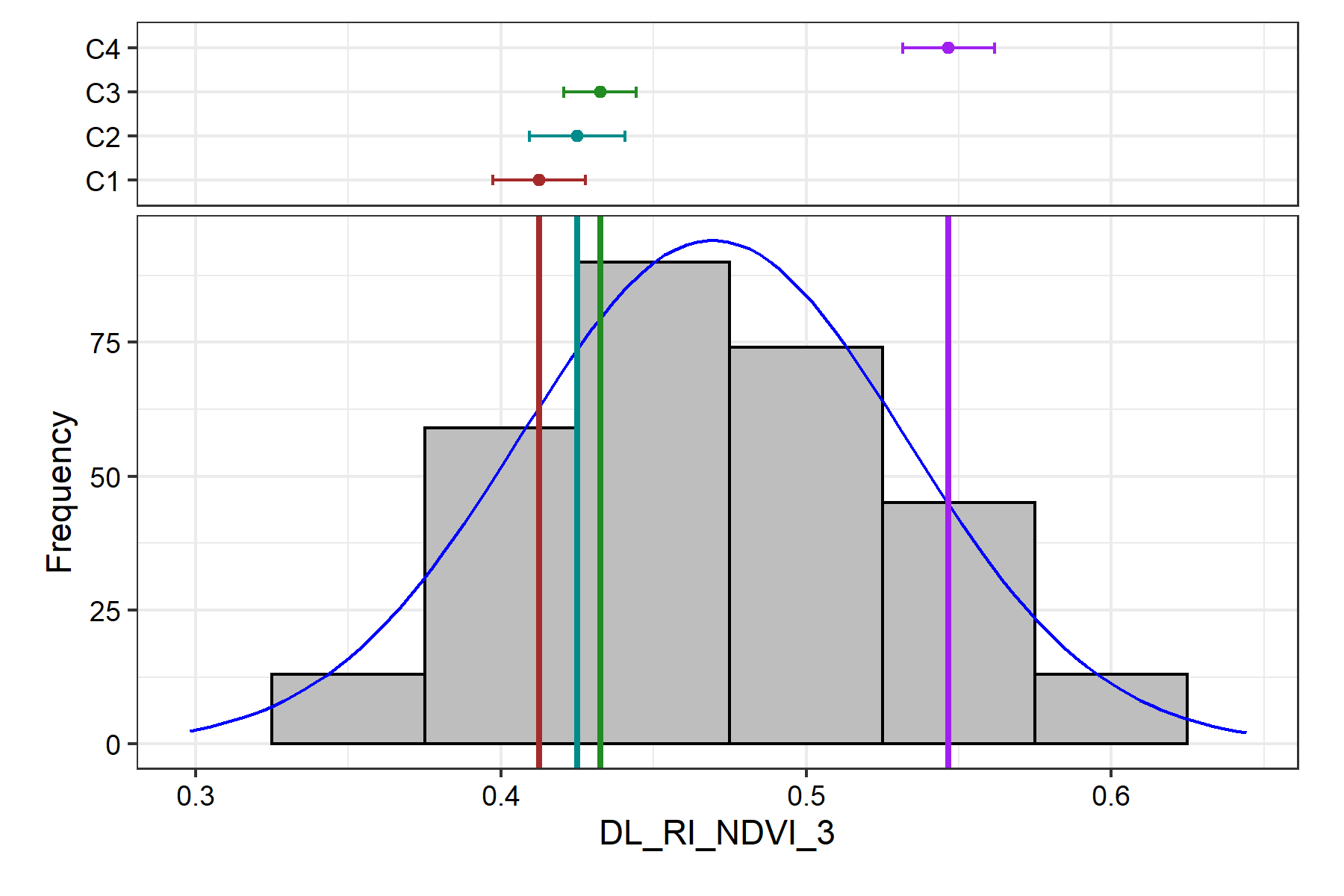

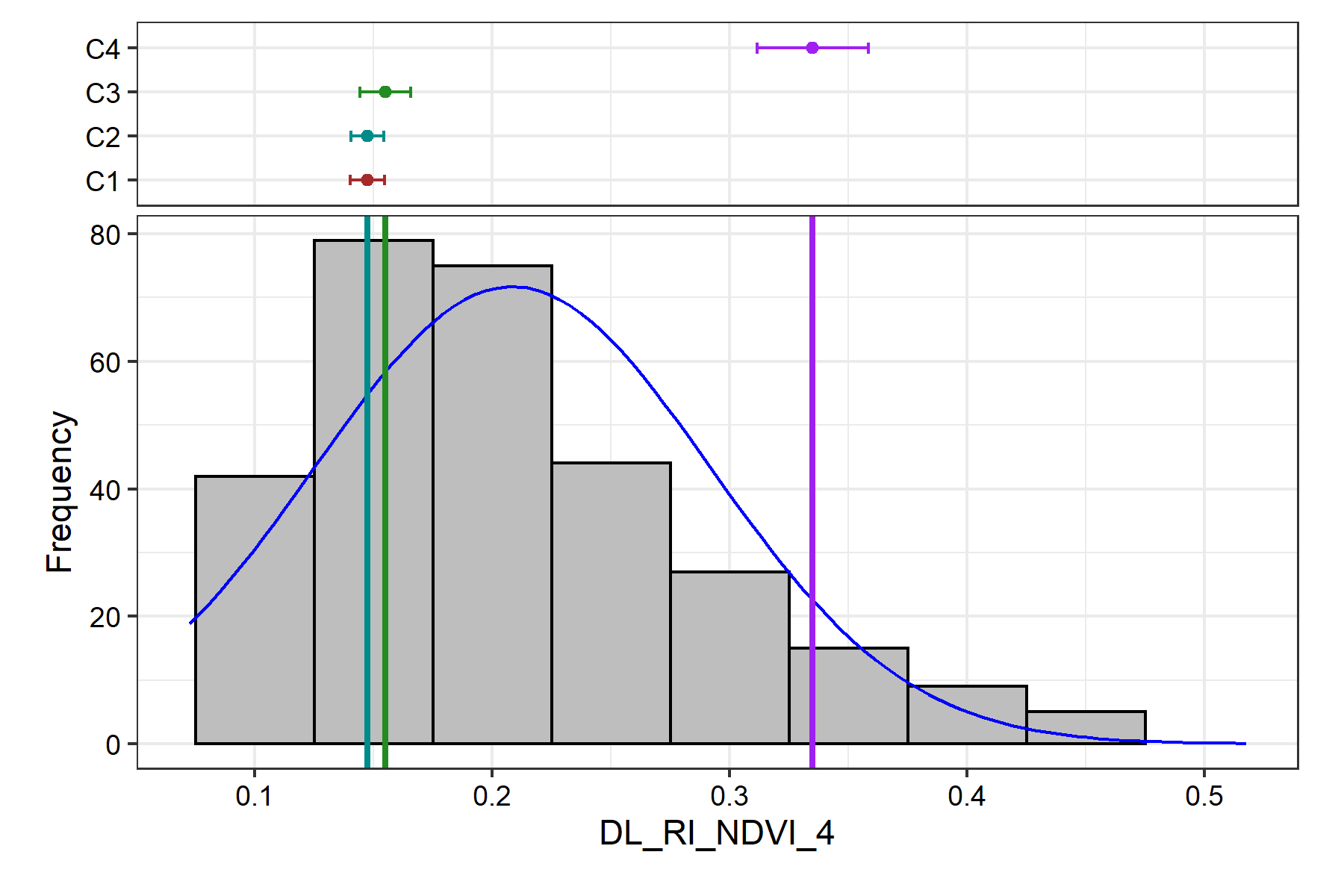

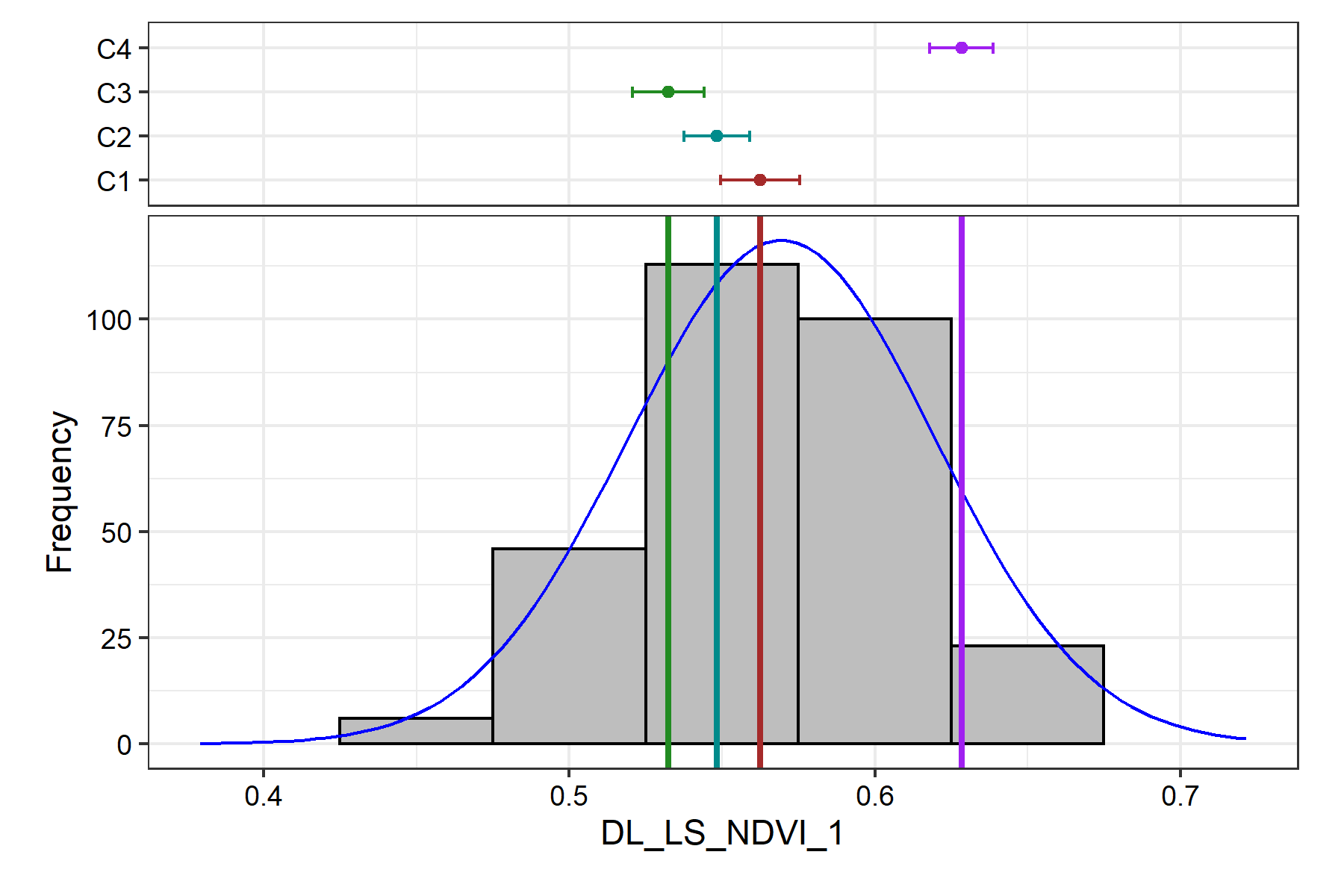

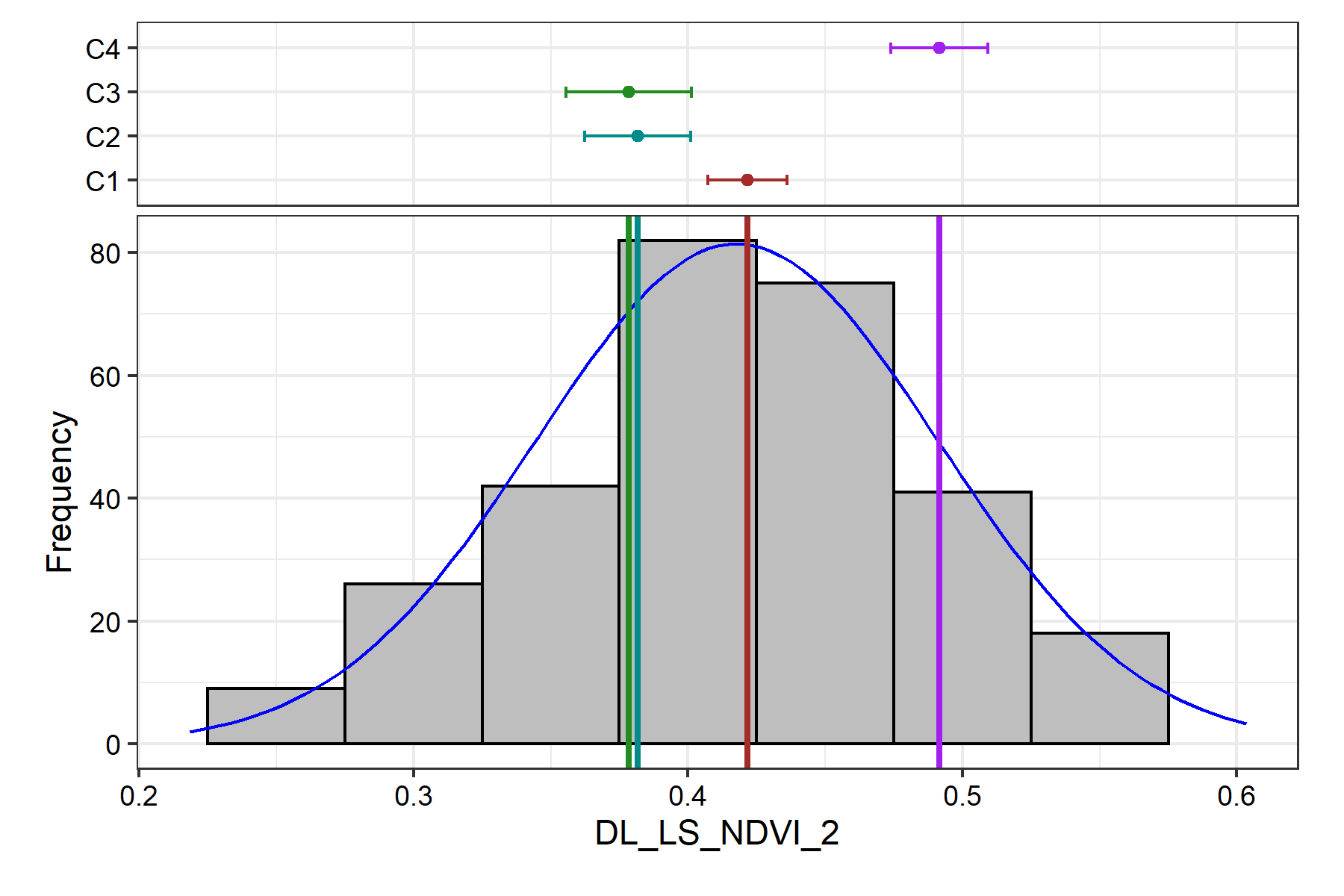

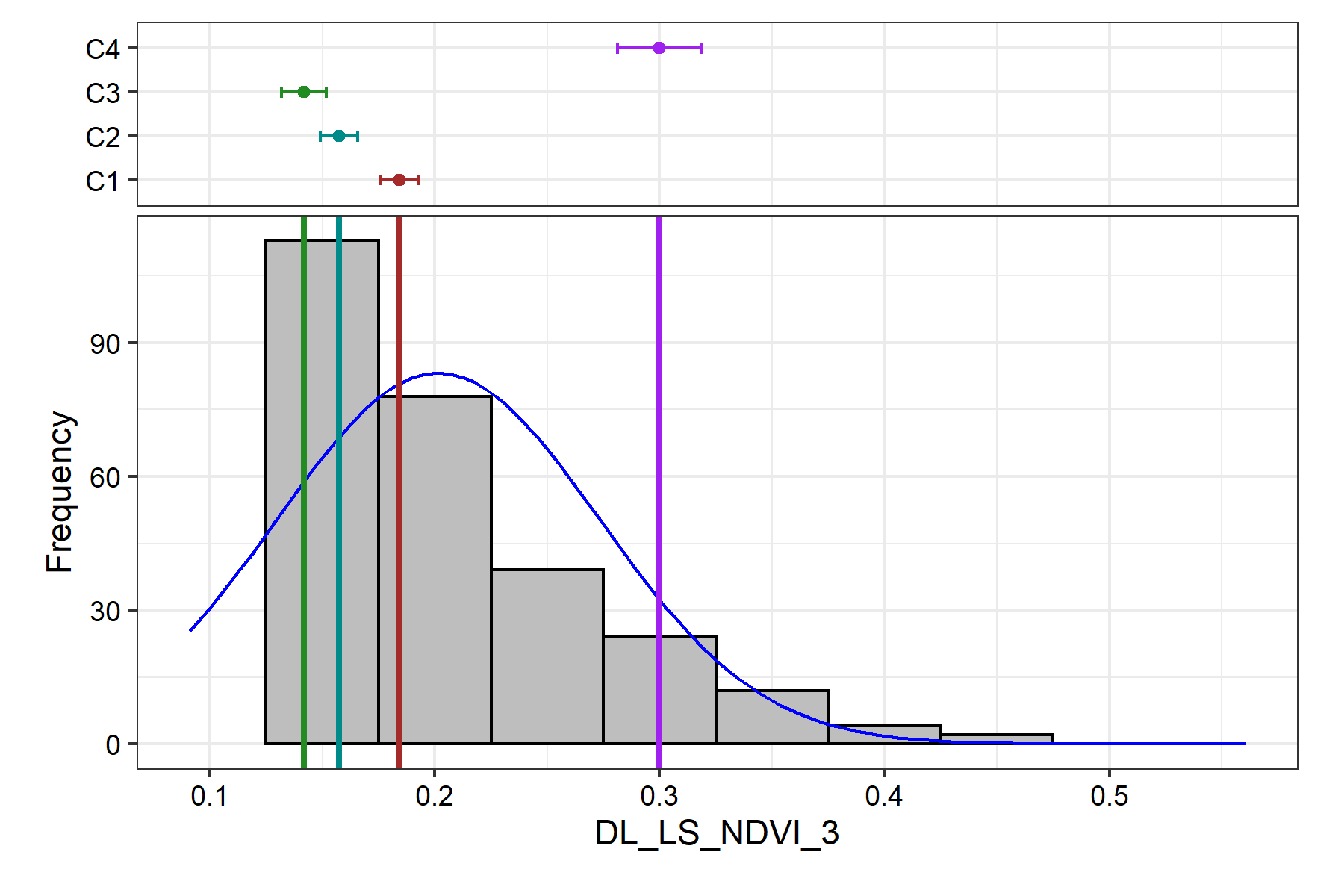

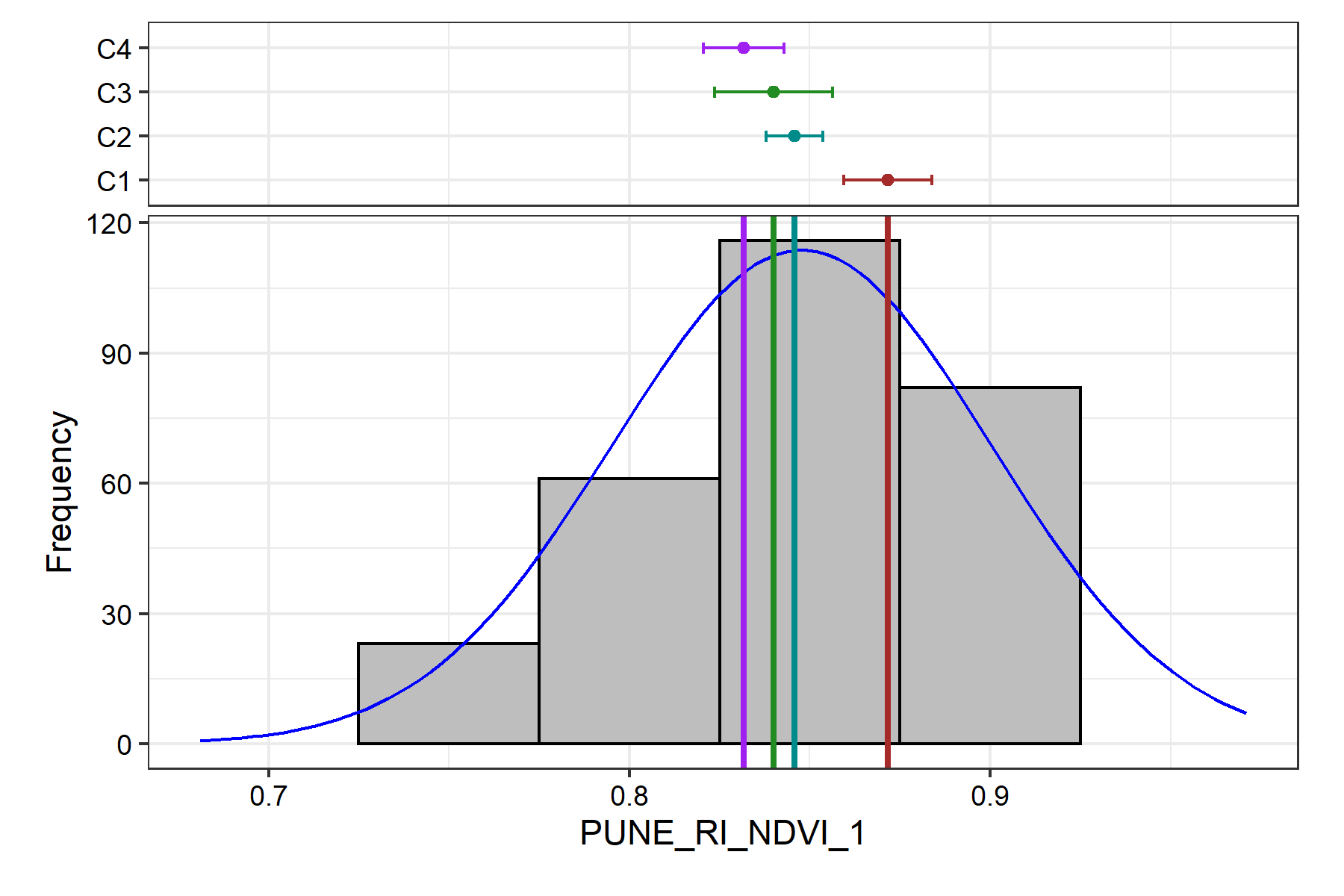

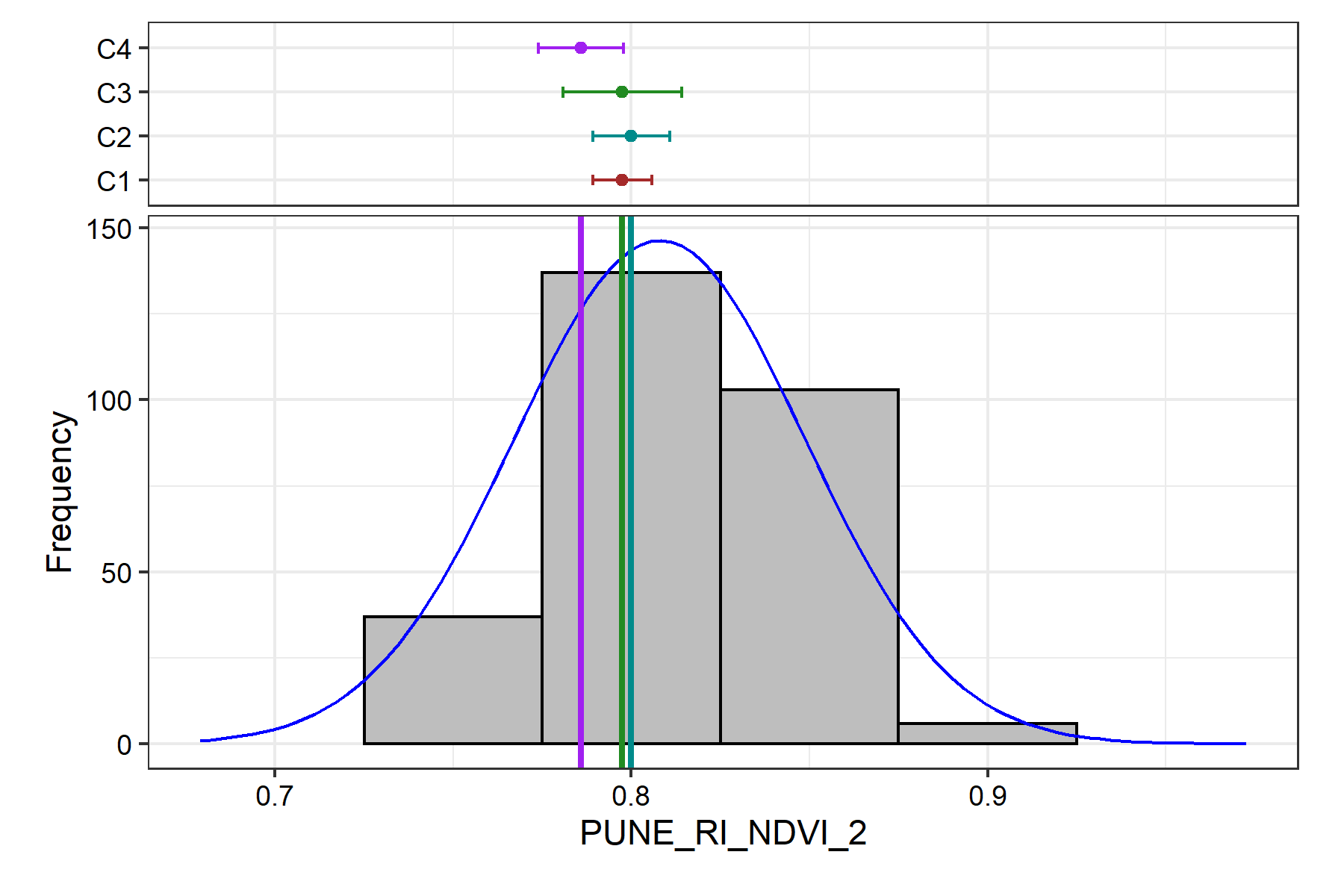

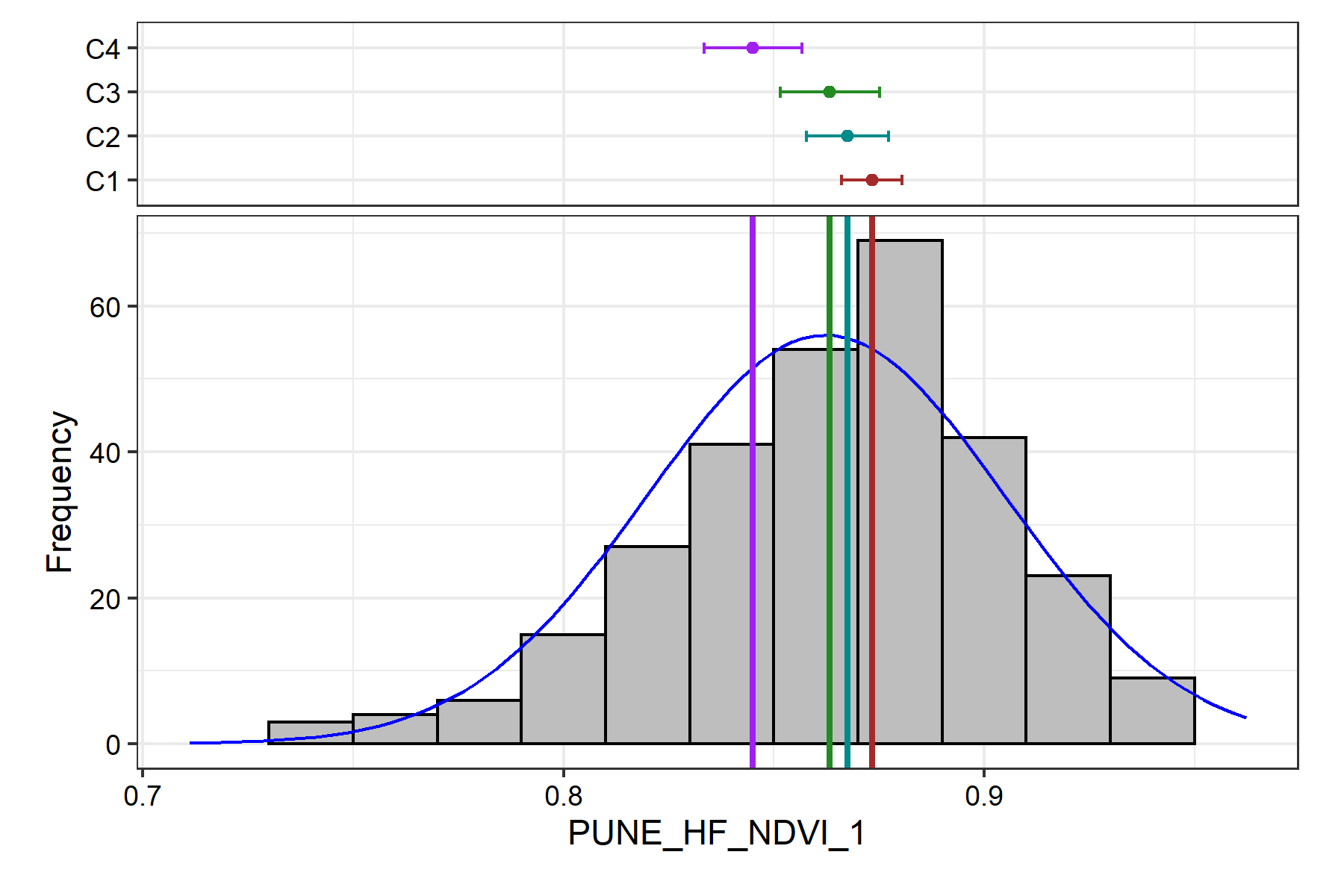

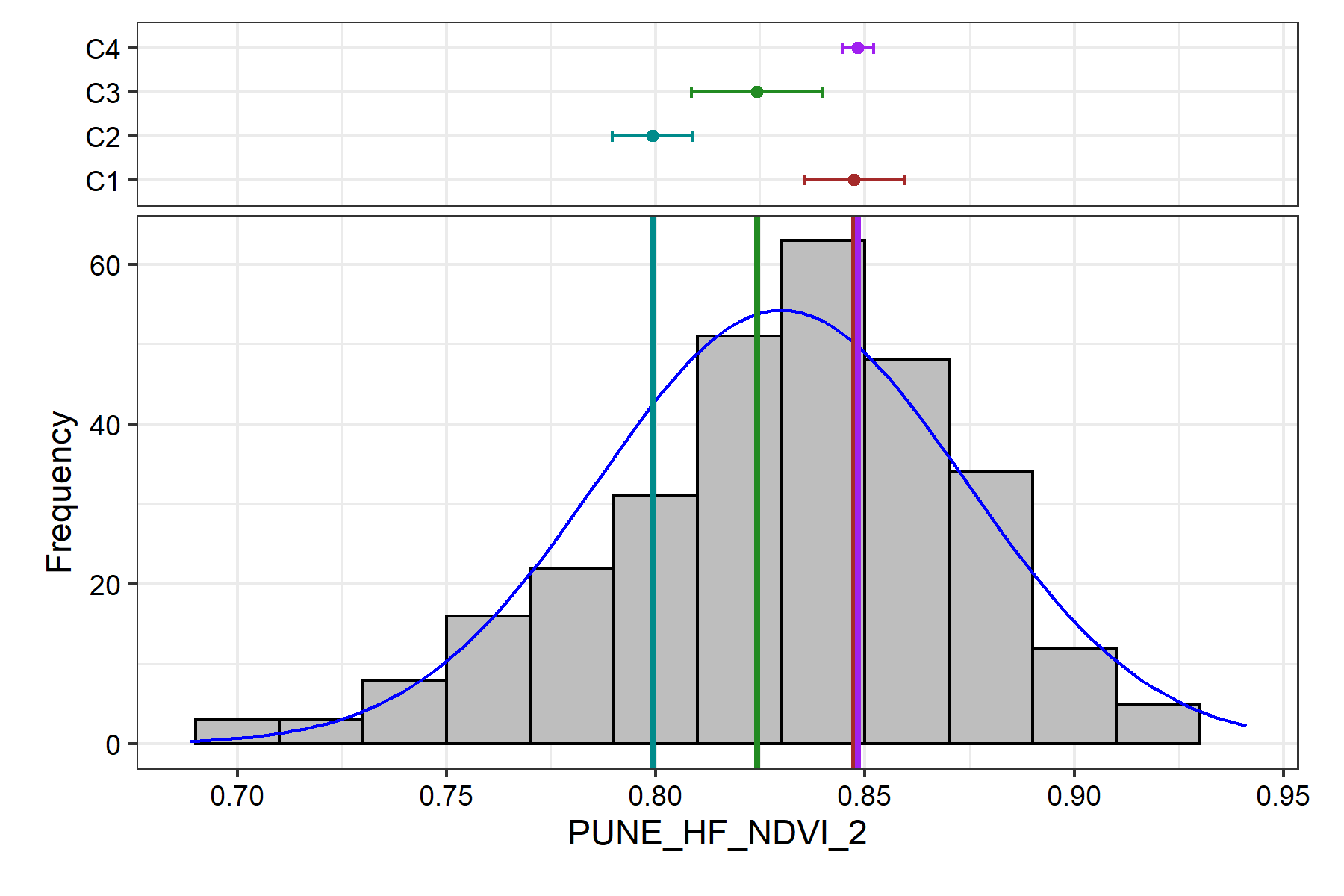


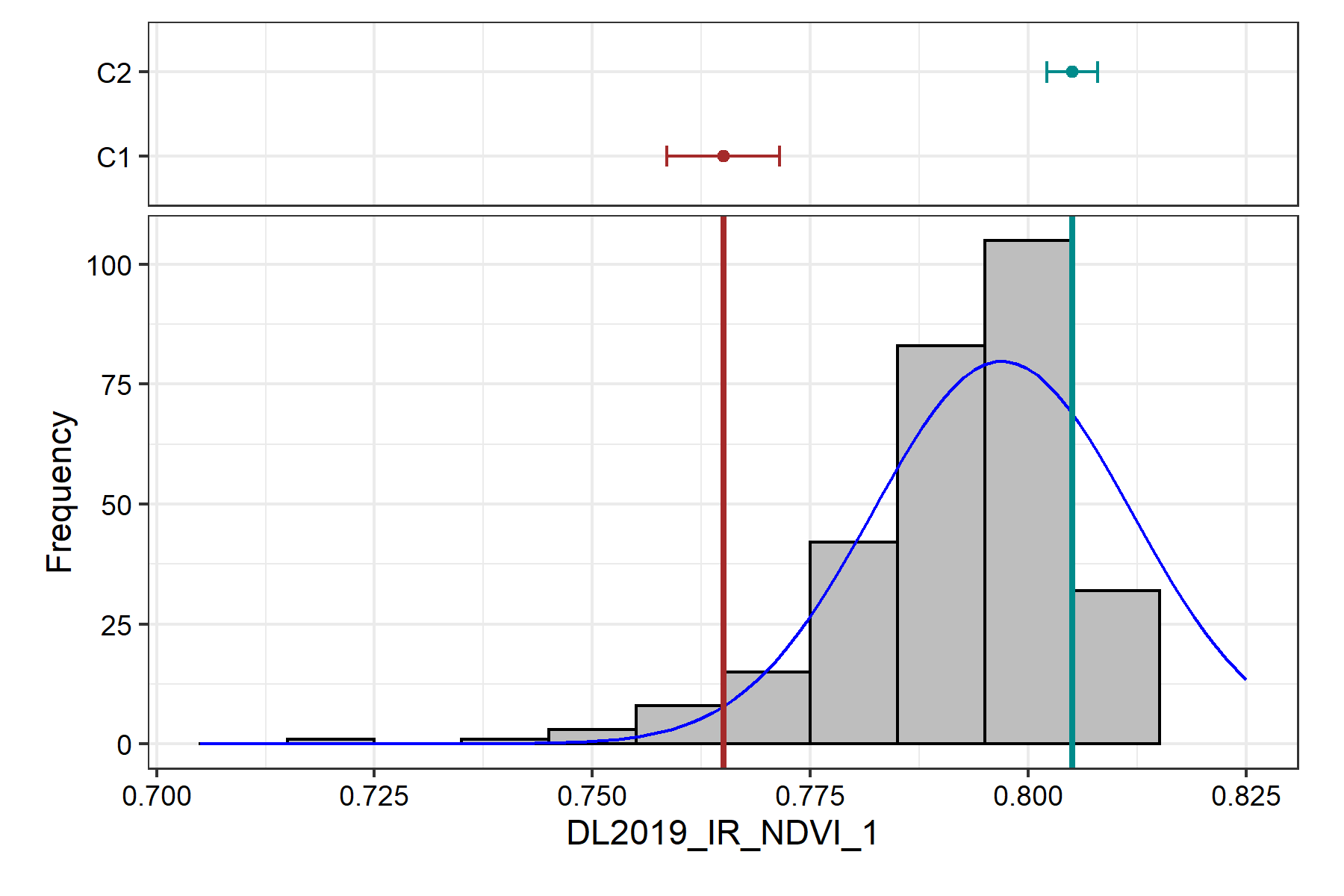

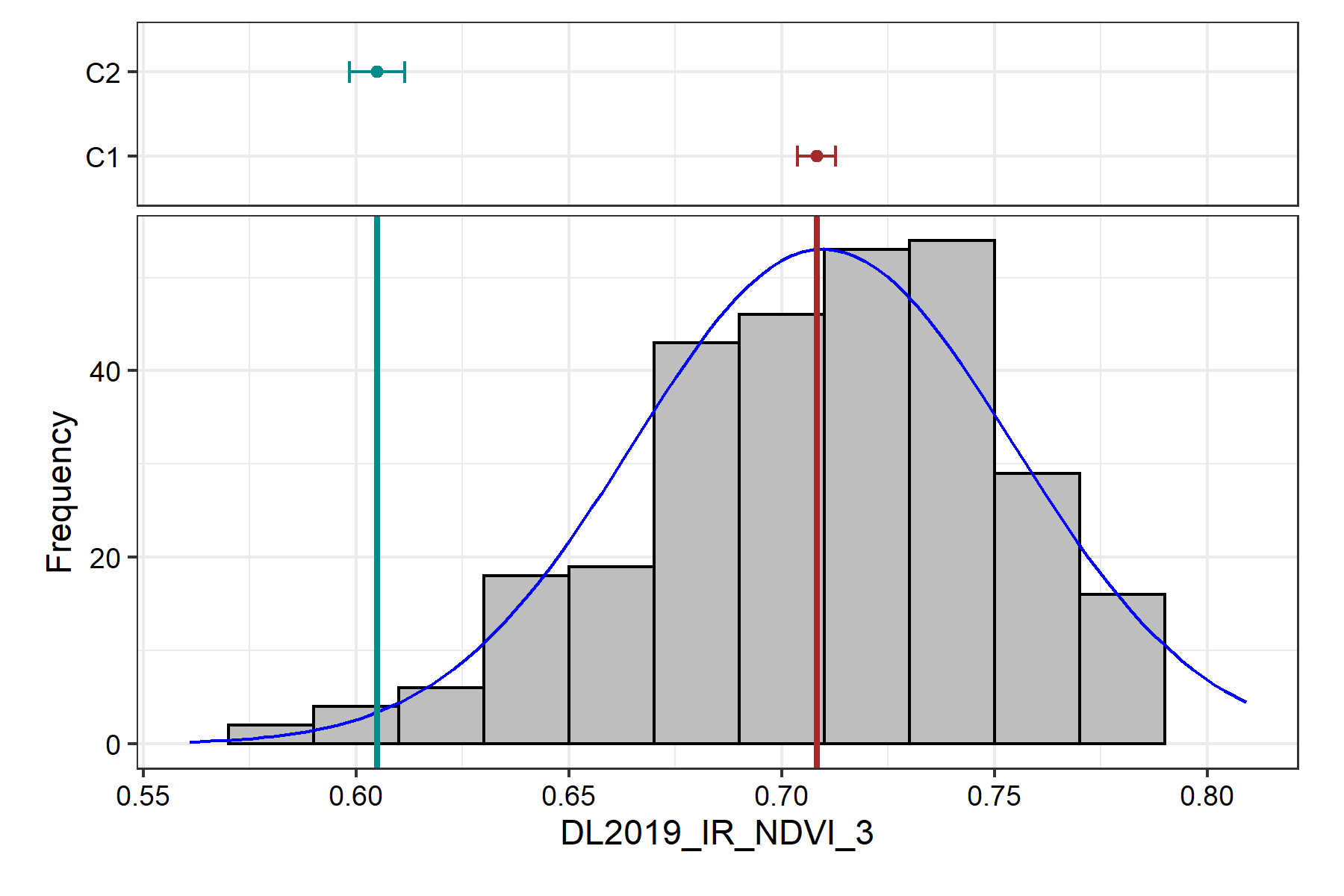

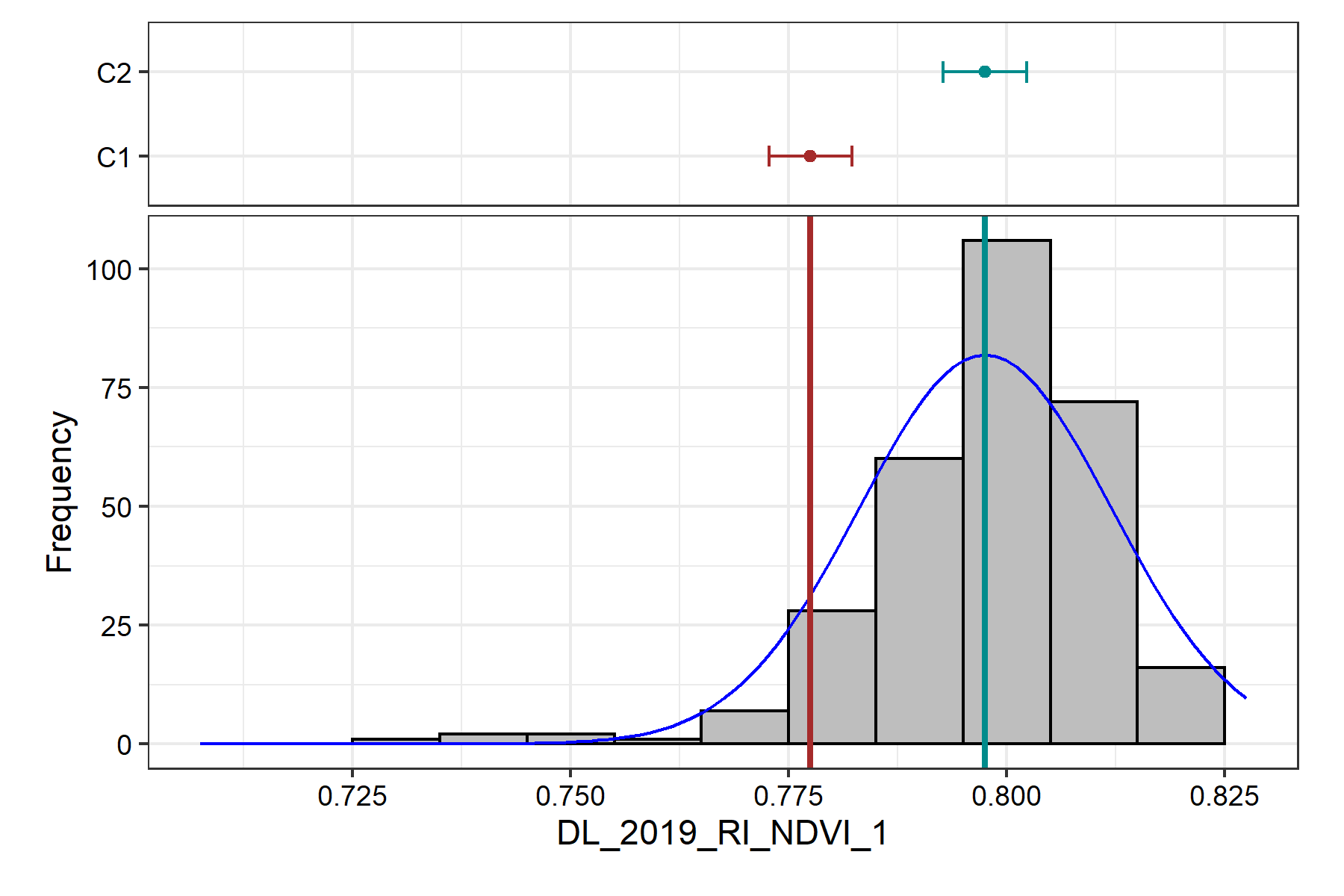

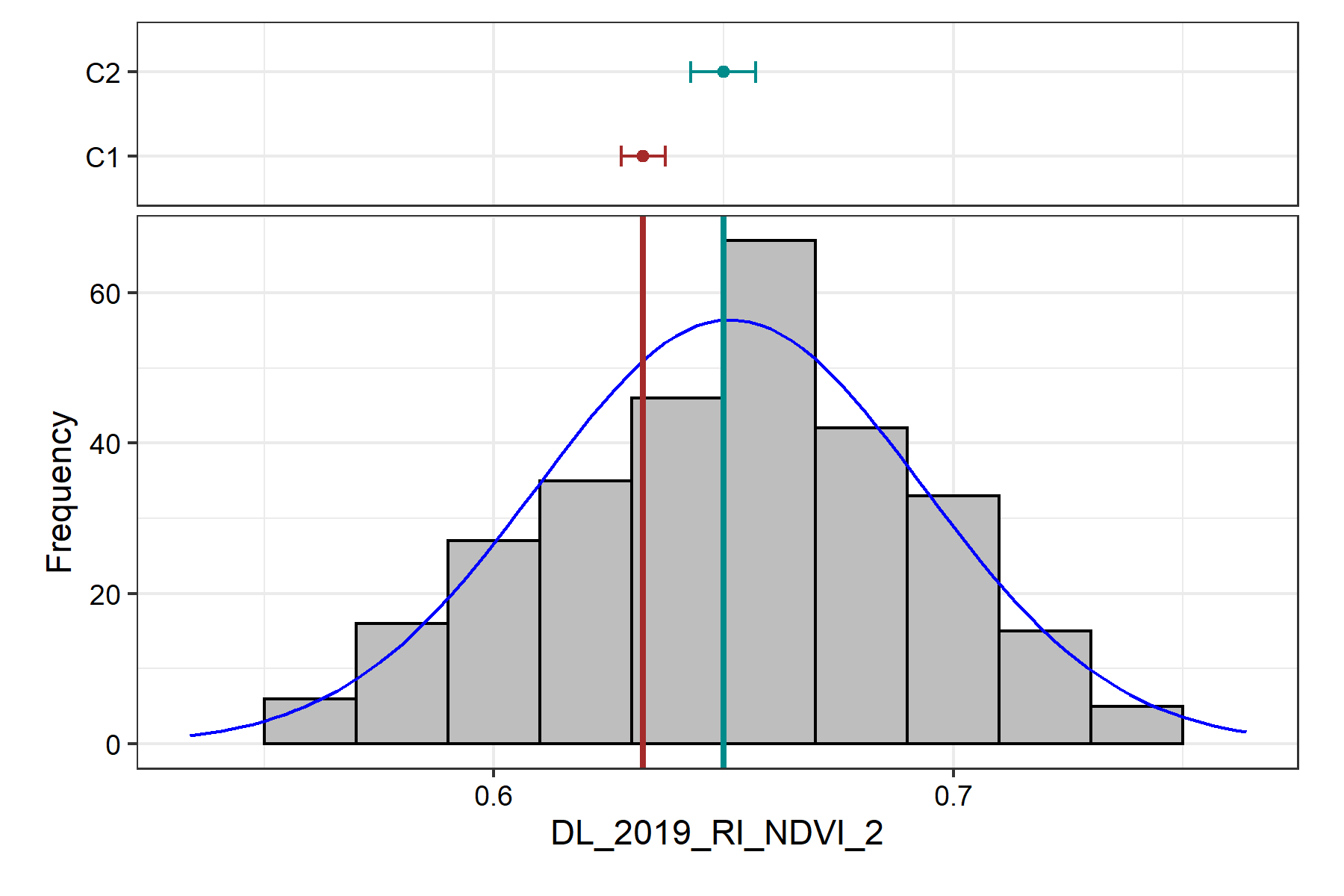


SPAD


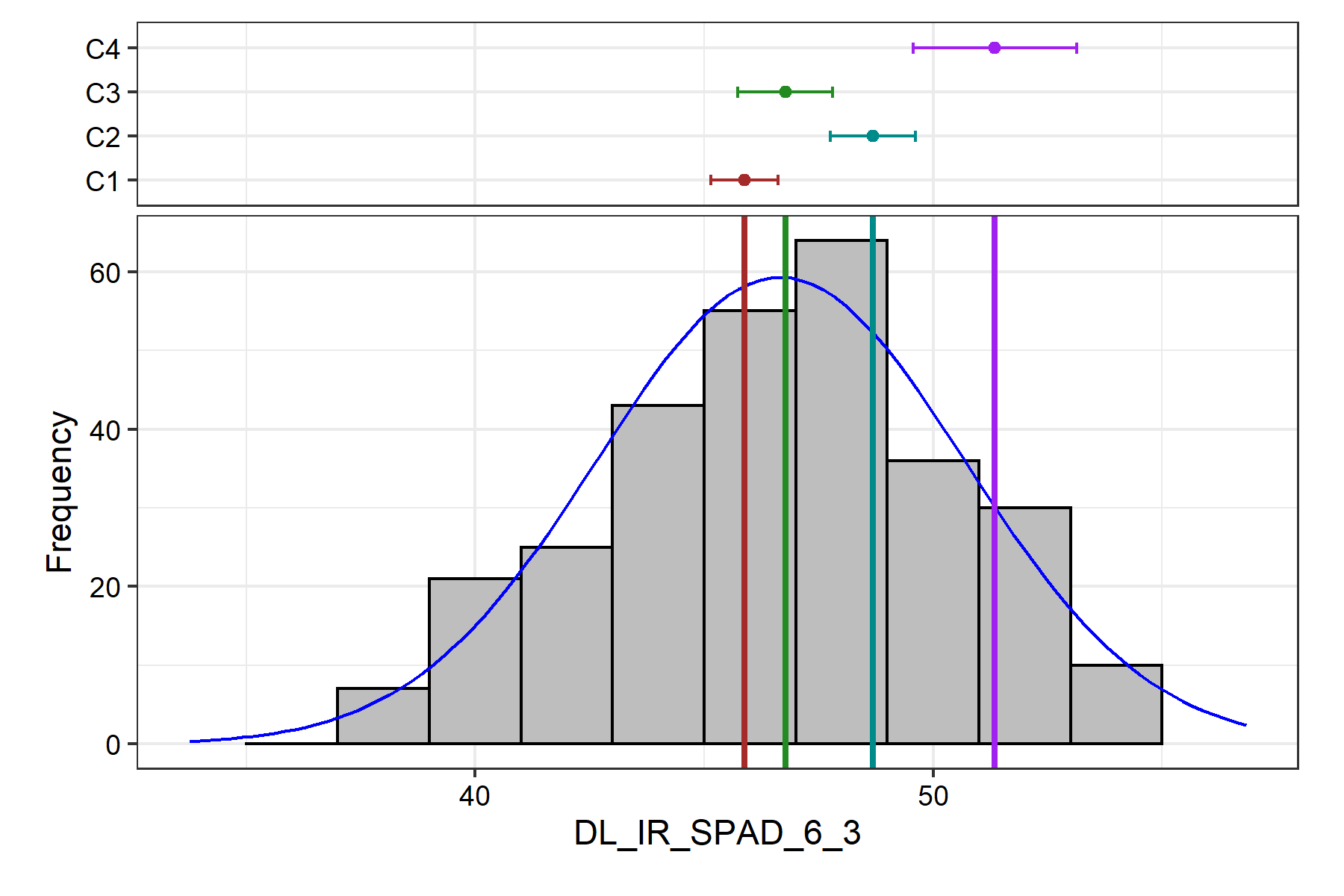

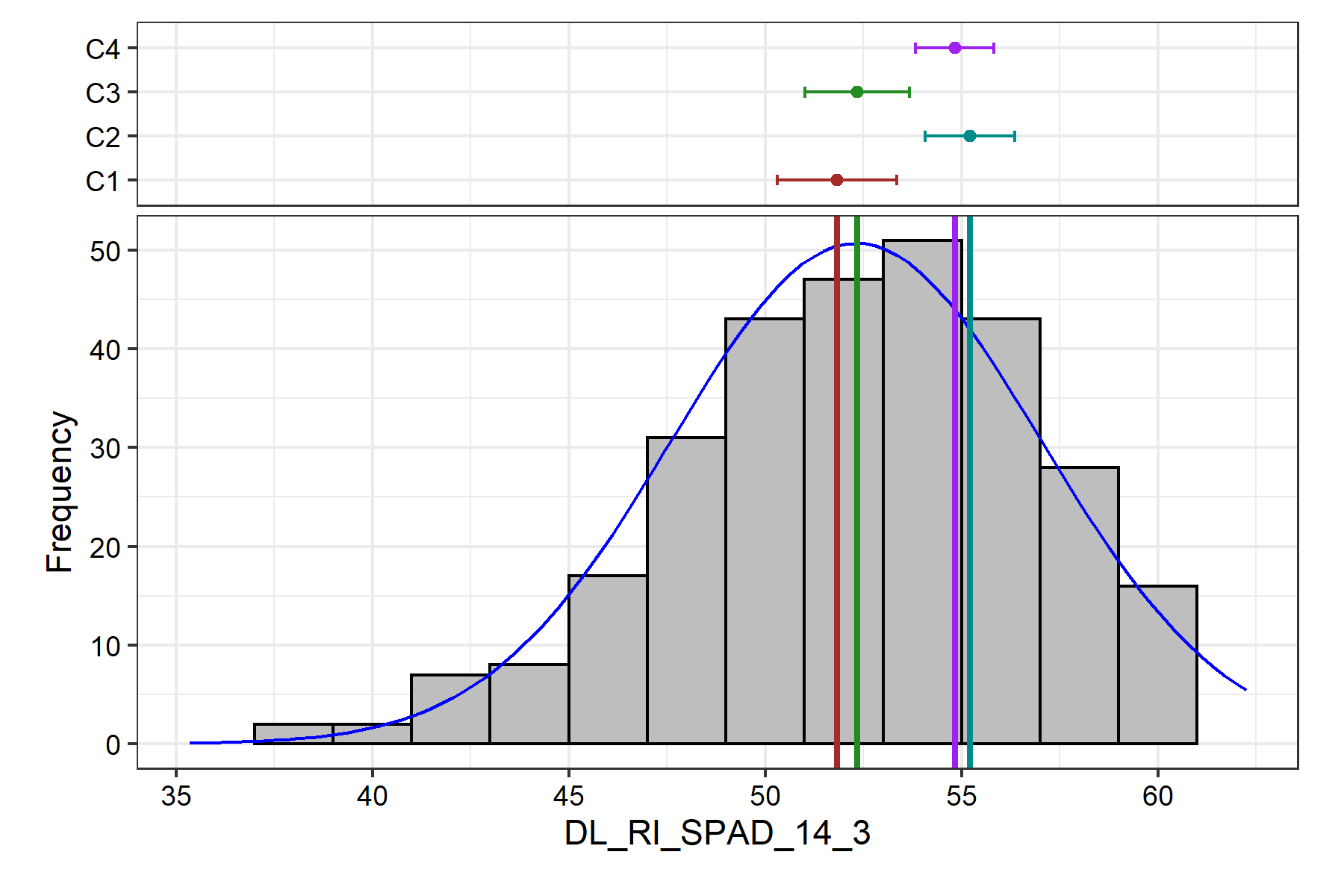

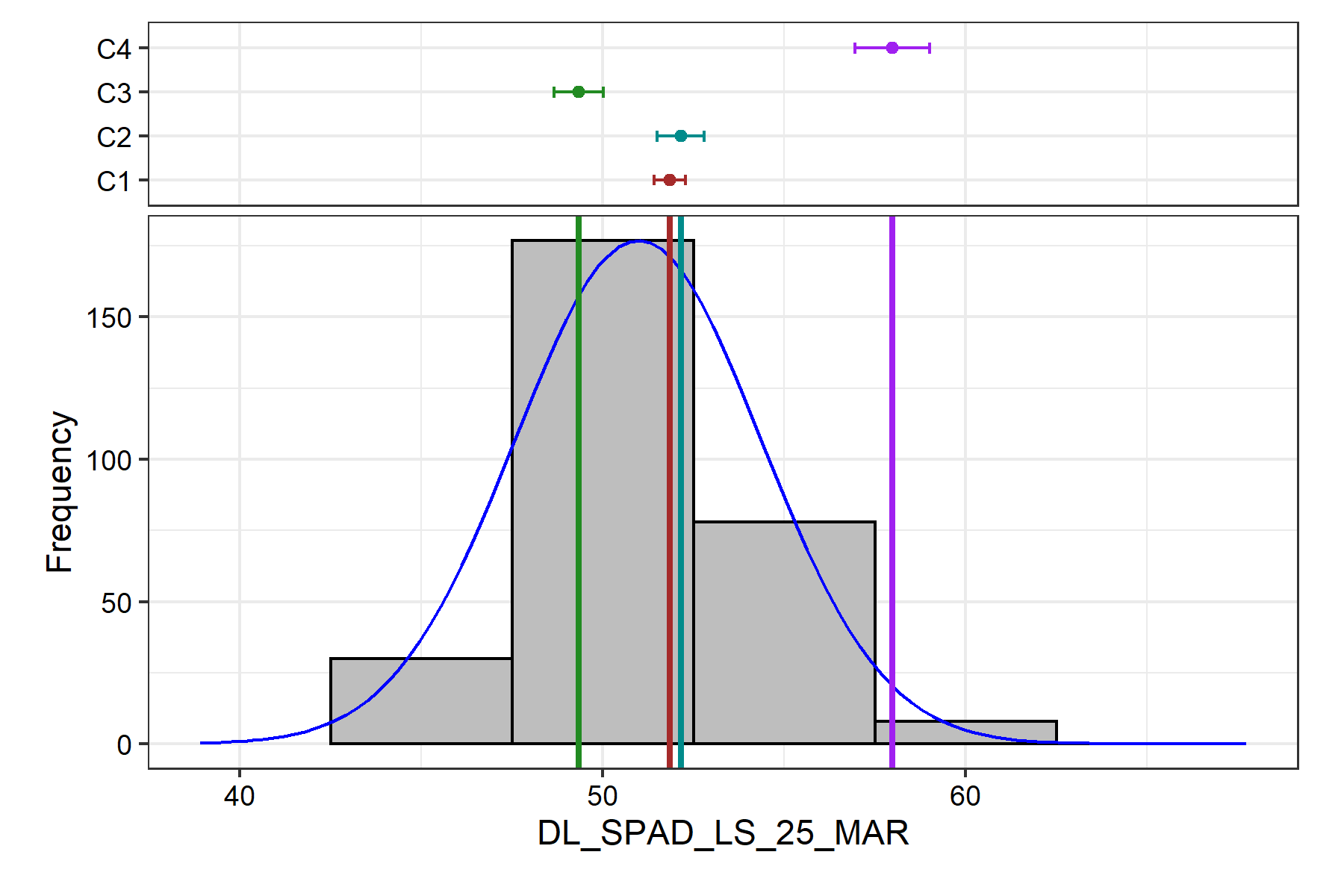

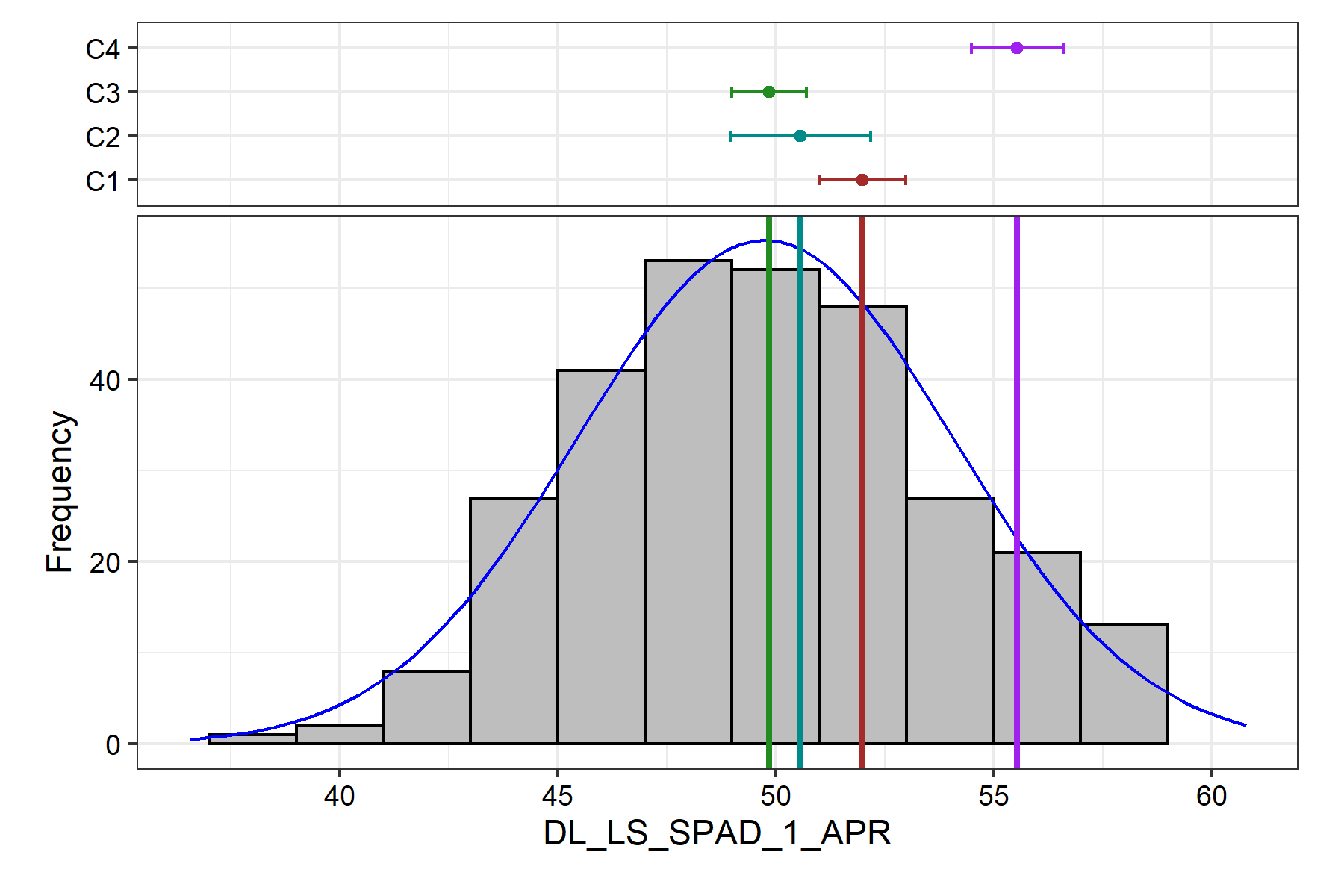


CT


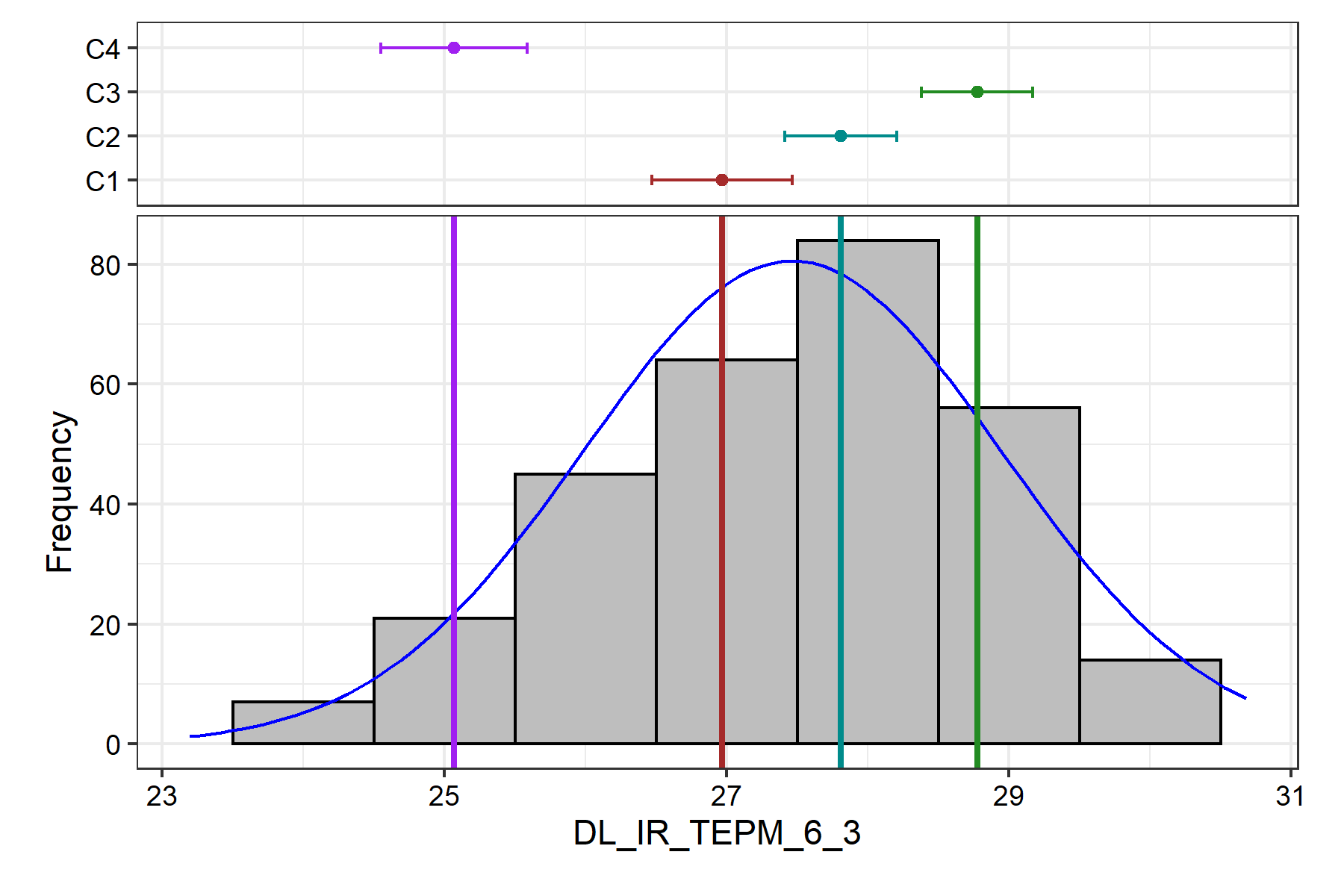

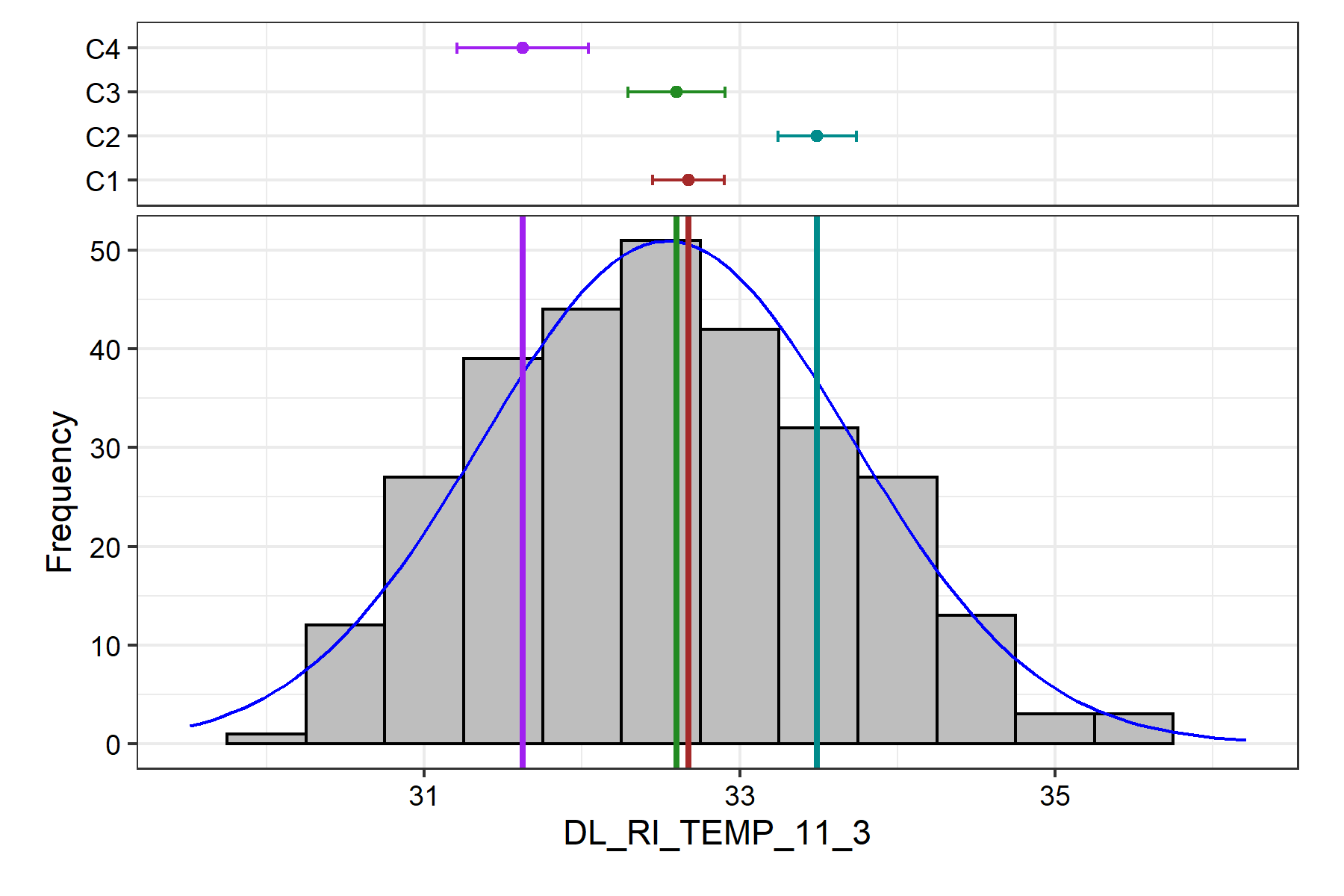

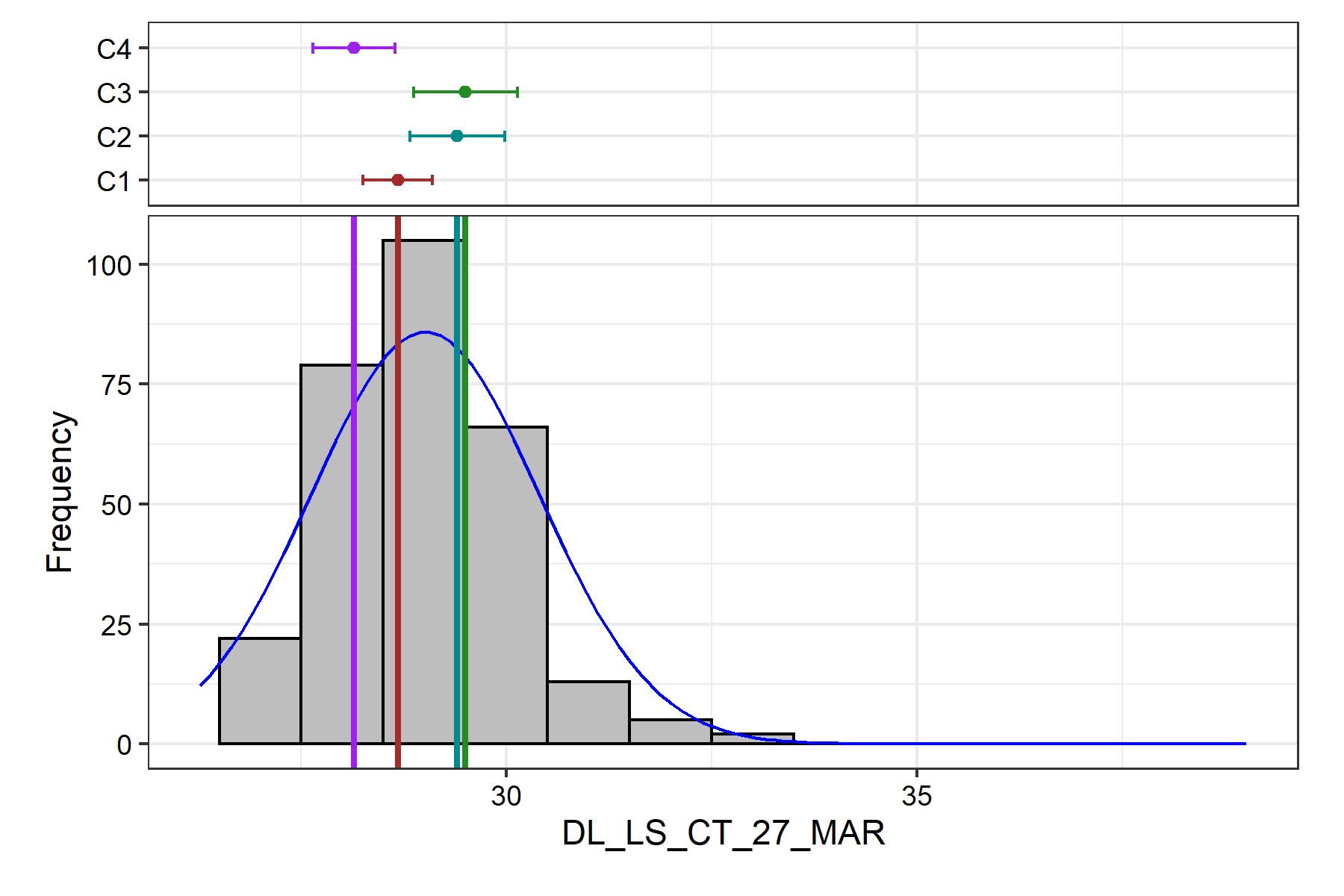

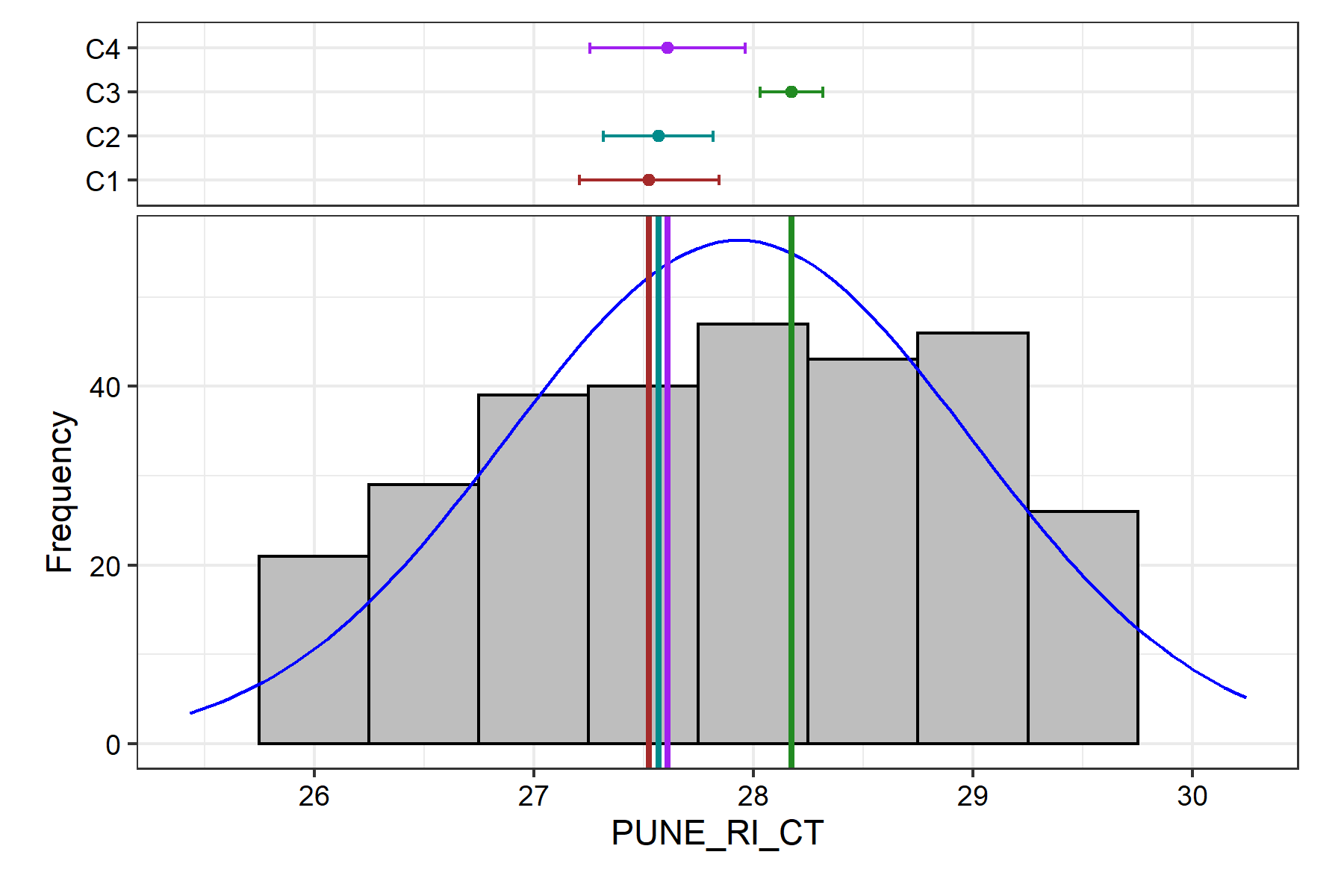

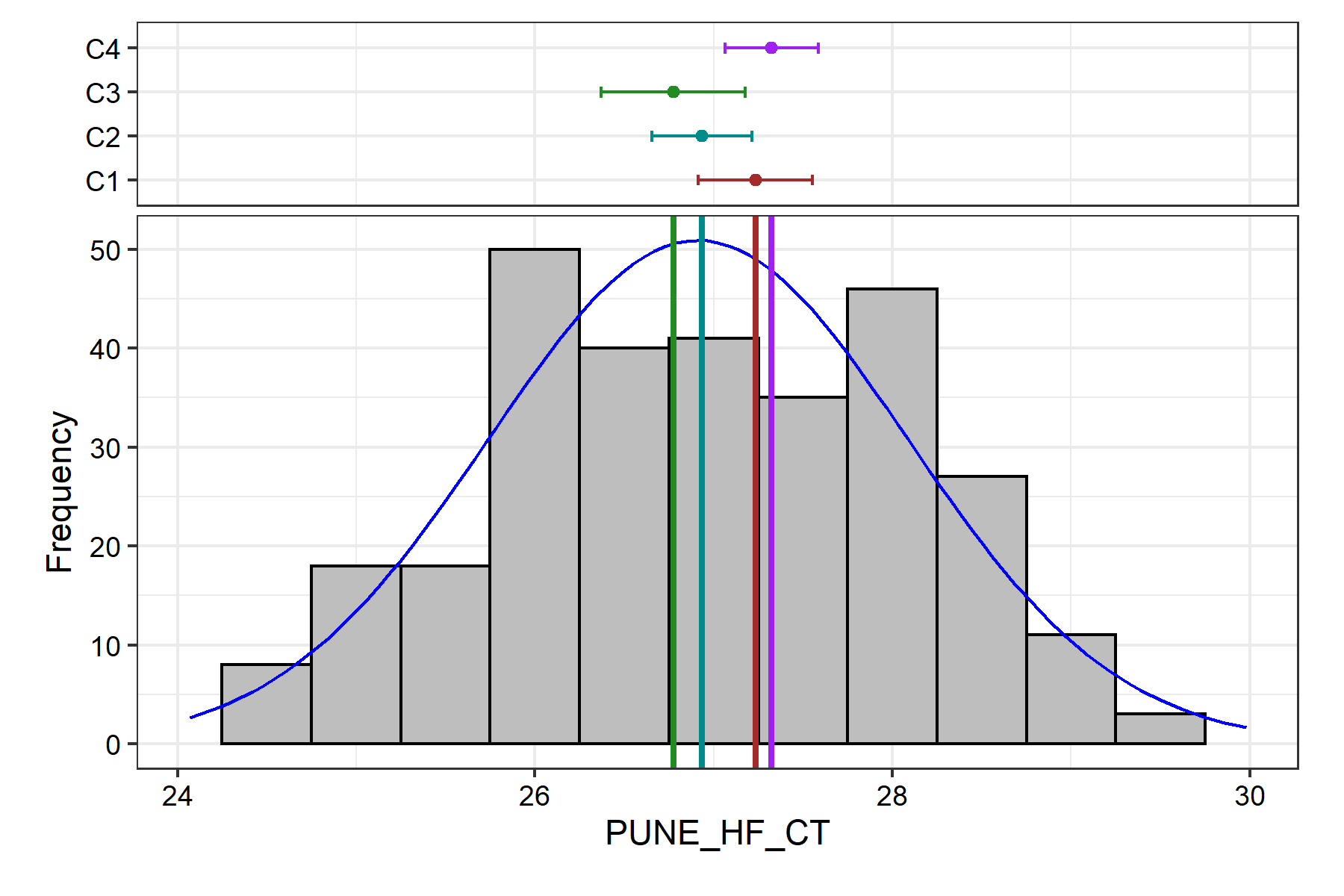

Supplement: Supplementary file 2 [file Data_Sheet_2.ZIP › Supp.Figure 1.docx]
