# Supplementary material for: Genome-wide association mapping for component traits of drought and heat tolerance in wheat
Source: Front Plant Sci. 2022 Aug 16;13:943033. doi: 10.3389/fpls.2022.943033 (PMC9429996; doi:10.3389/fpls.2022.943033)

Supplementary Fig 3. Environment wise PCA Biplots from the traits under investigation

DL_RI_2020


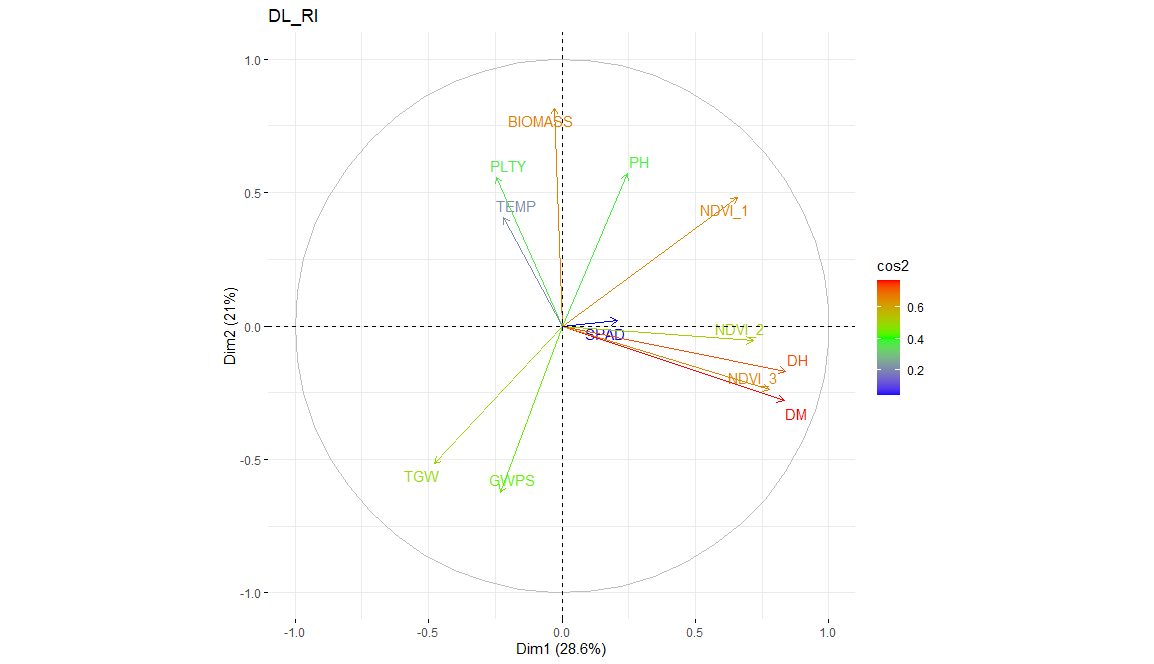


DL_LS_2020


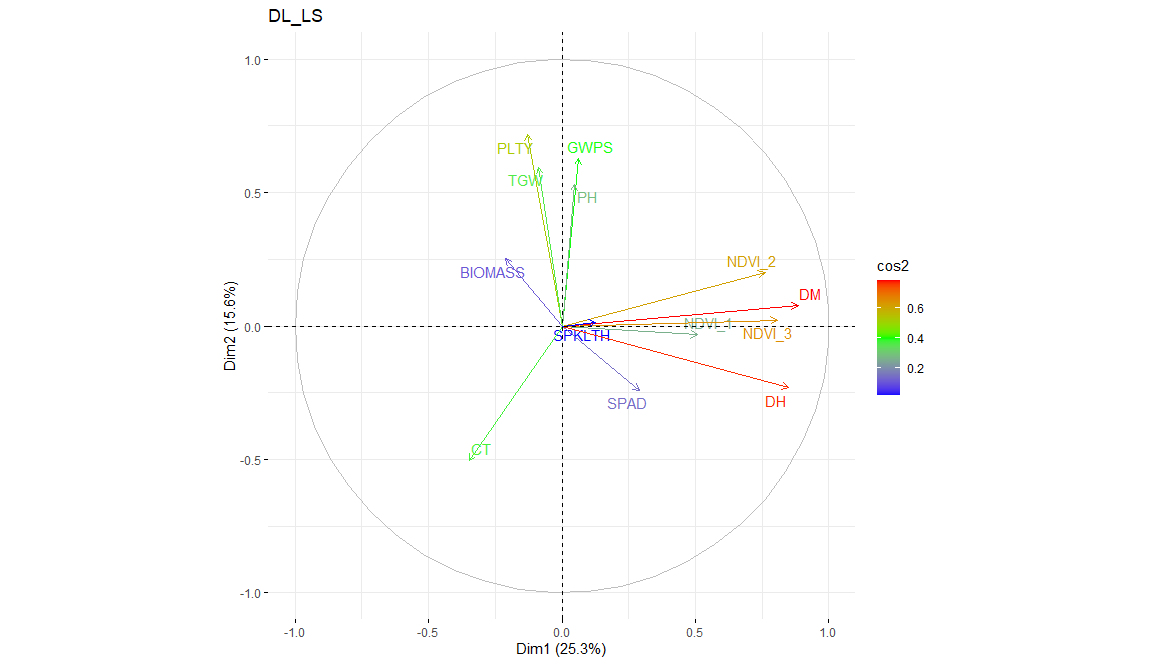


IIWBR_2020


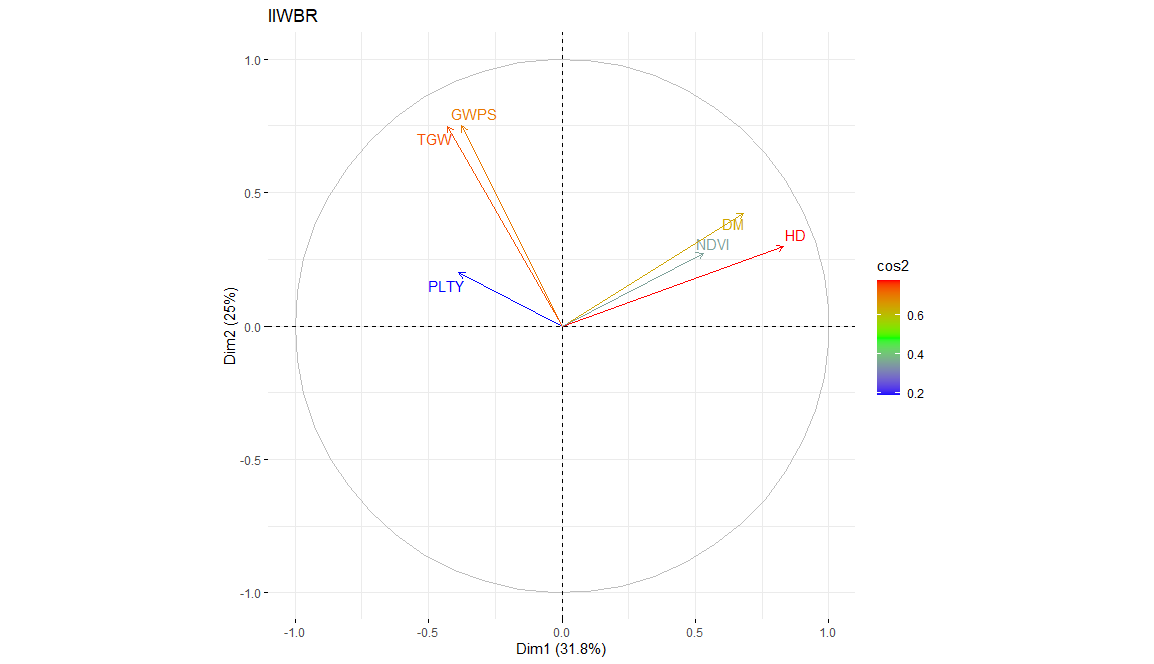


JR_2020


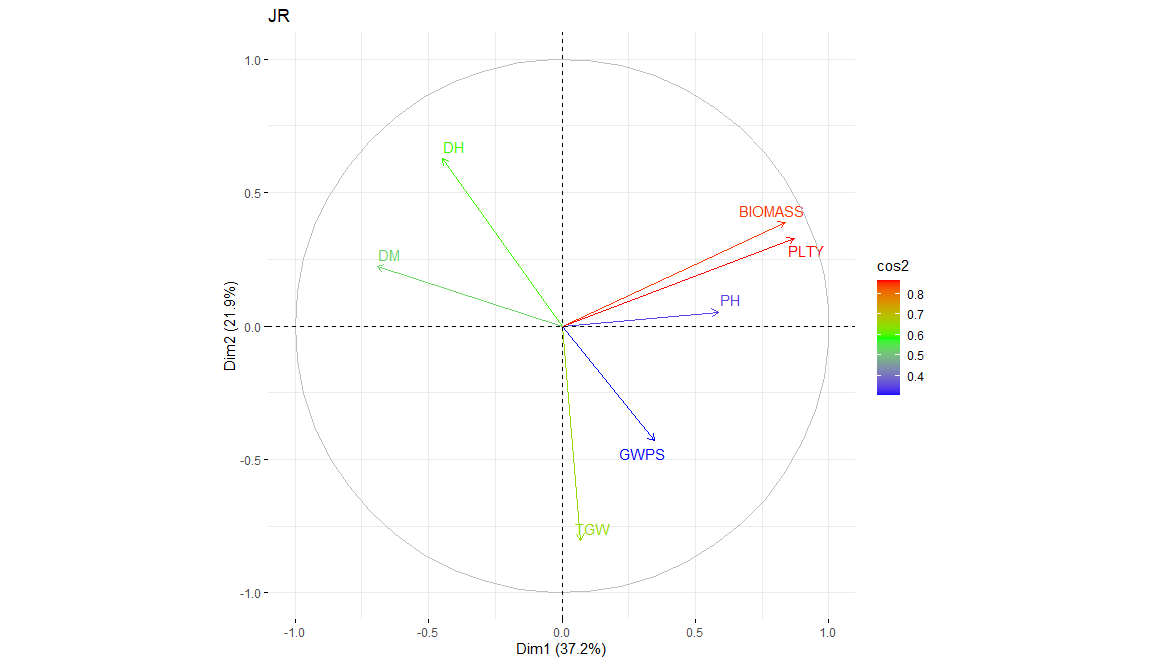


PUNE_IR_2020


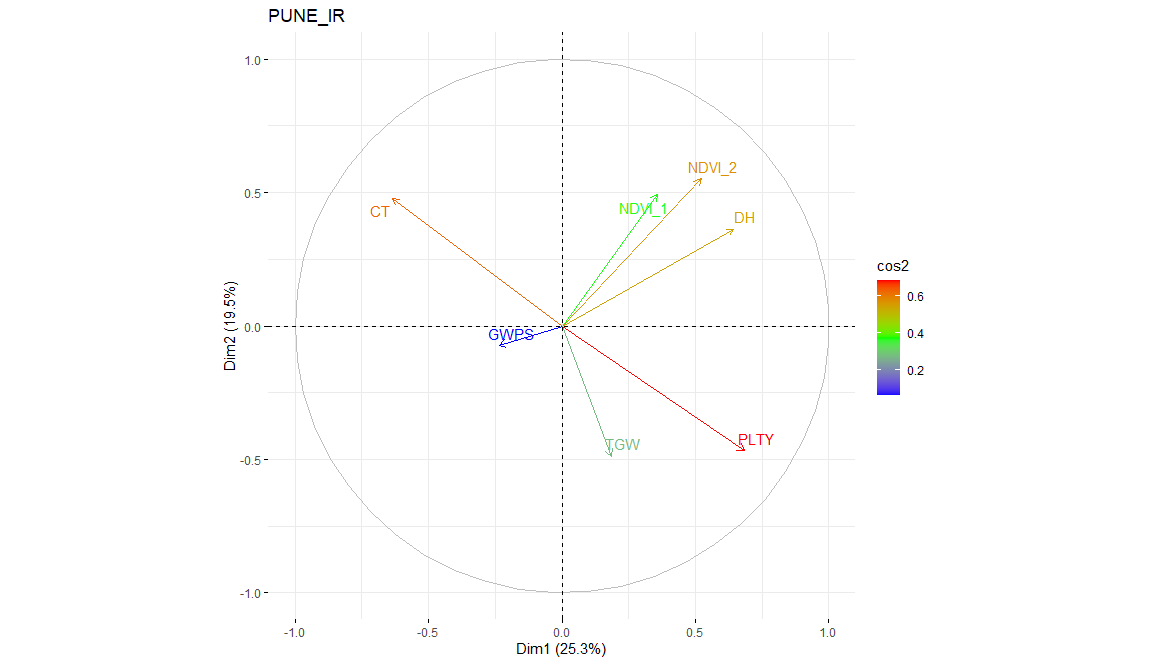


PUNE_RI_2020


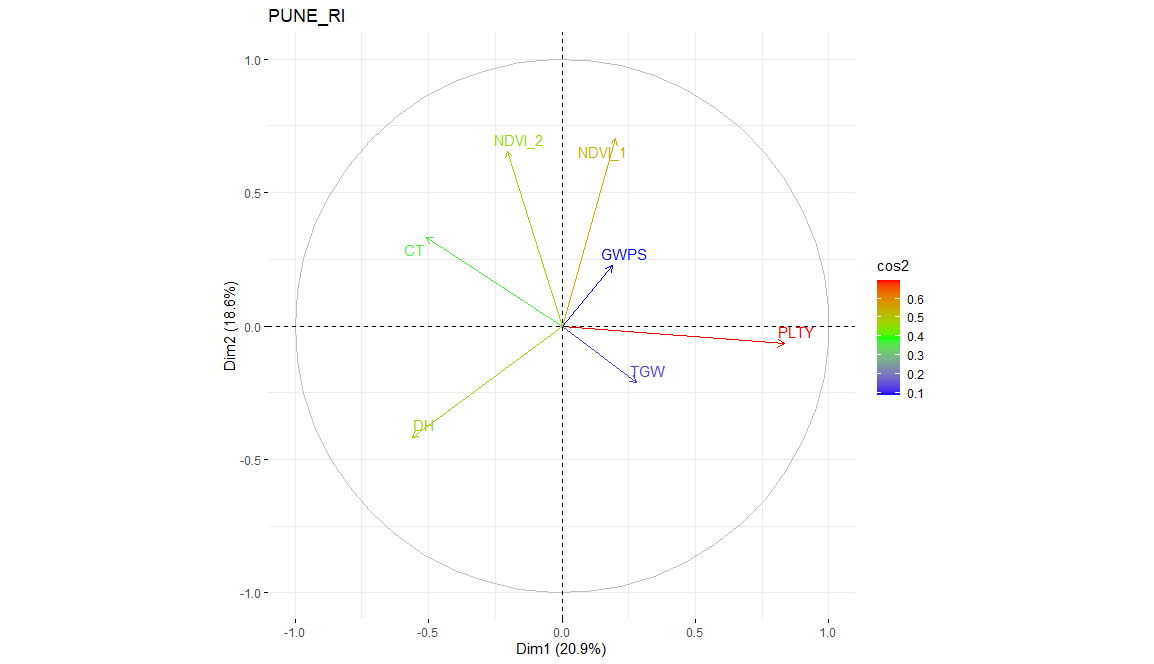

Supplement: Supplementary file 2 [file Data_Sheet_2.ZIP › Supp.Figure 3.docx]
