# Supplementary material for: Genome-wide association mapping for component traits of drought and heat tolerance in wheat
Source: Front Plant Sci. 2022 Aug 16;13:943033. doi: 10.3389/fpls.2022.943033 (PMC9429996; doi:10.3389/fpls.2022.943033)
Supplement: Supplementary file 2 [file Data_Sheet_2.ZIP › Supp.Figure 4.docx]

Supplementary Fig 4. Manhattan plots with significant MTAs Identified at treatment environments for the studied traits.

Bio mass

Days to Heading

Gwps

PH

TGW

PLTY

DM

NDVI

SPKL

TEPM
