# Supplementary figures and images for: Genome-wide association mapping for component traits of drought and heat tolerance in wheat
Source: Front Plant Sci. 2022 Aug 16;13:943033. doi: 10.3389/fpls.2022.943033 (PMC9429996; doi:10.3389/fpls.2022.943033)

DL_IR_2019


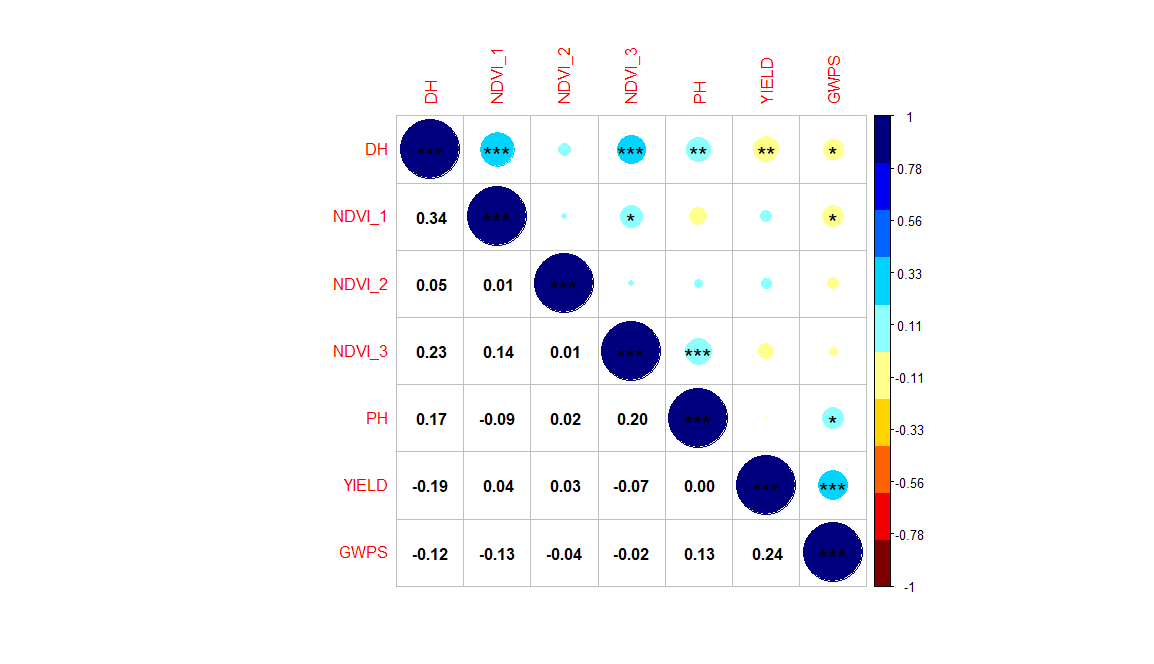


DL_RI_2019


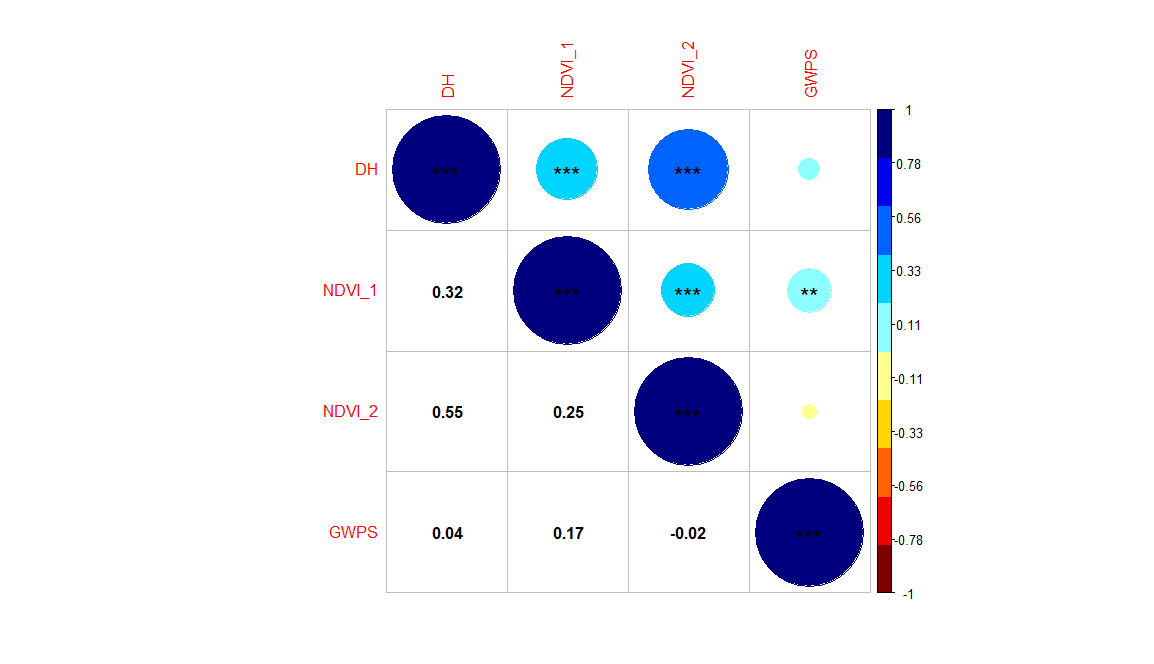


DL_LS_2019


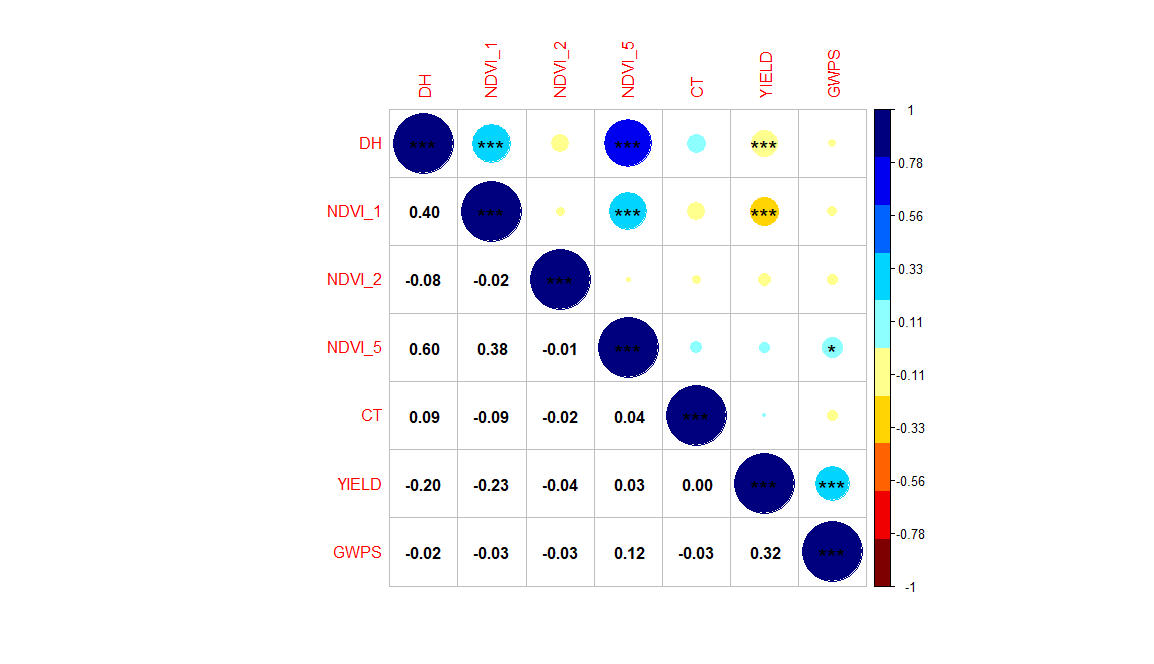


IIWBR_IR_2020


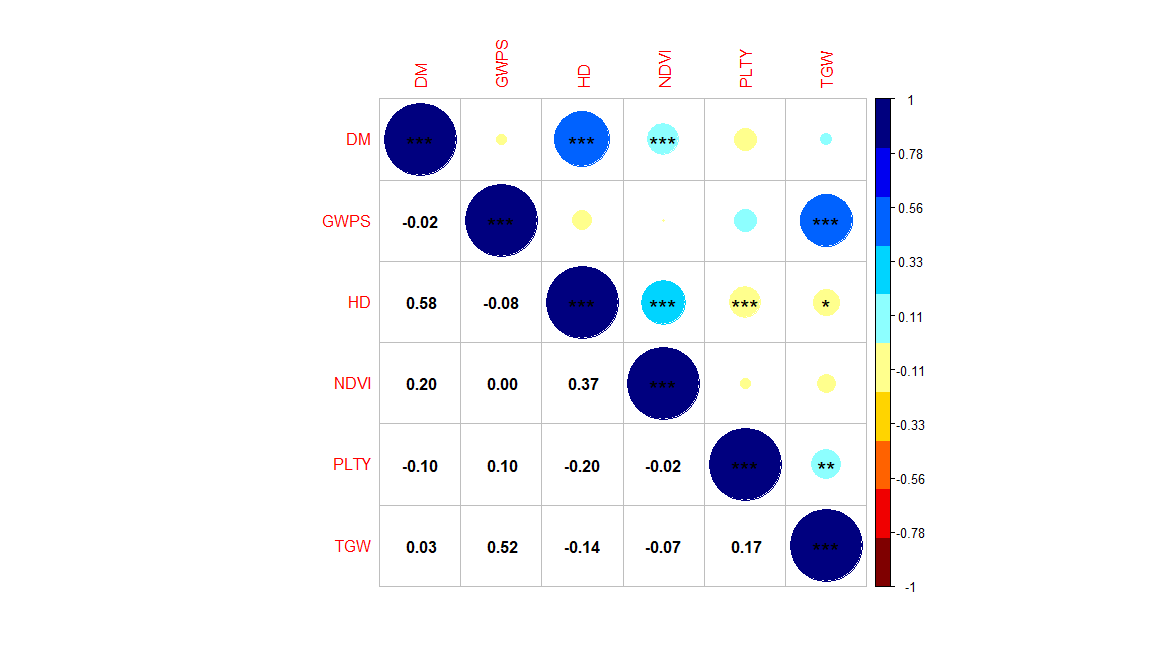


PUNE_IR_2020


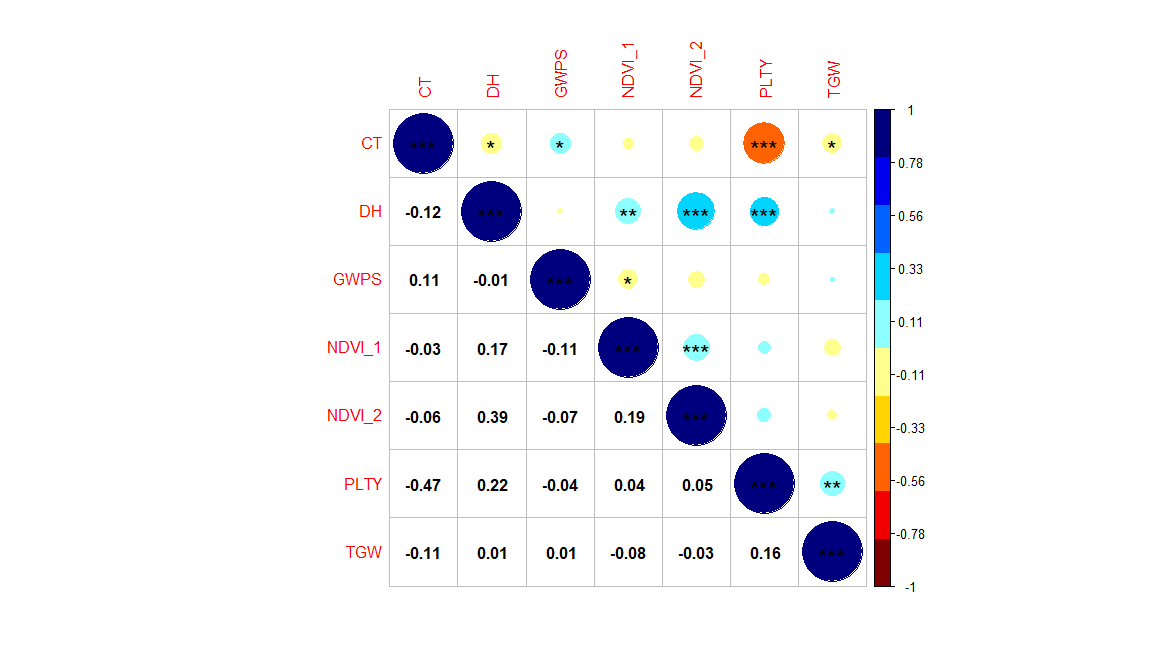


PUNE_RI_2020


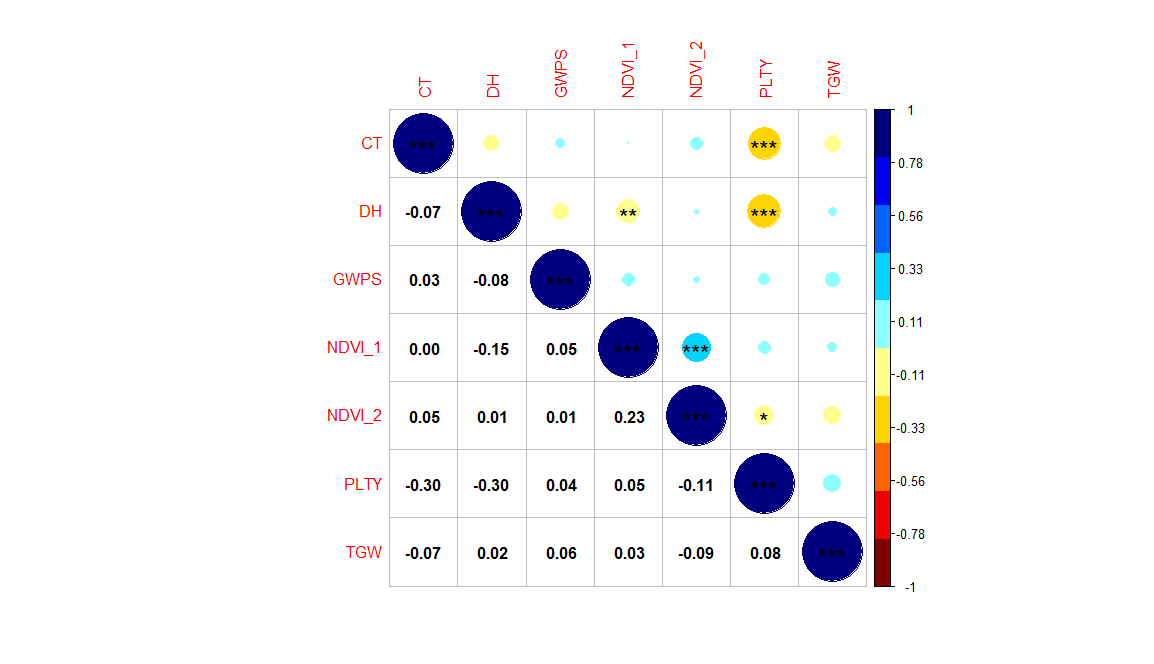


JR
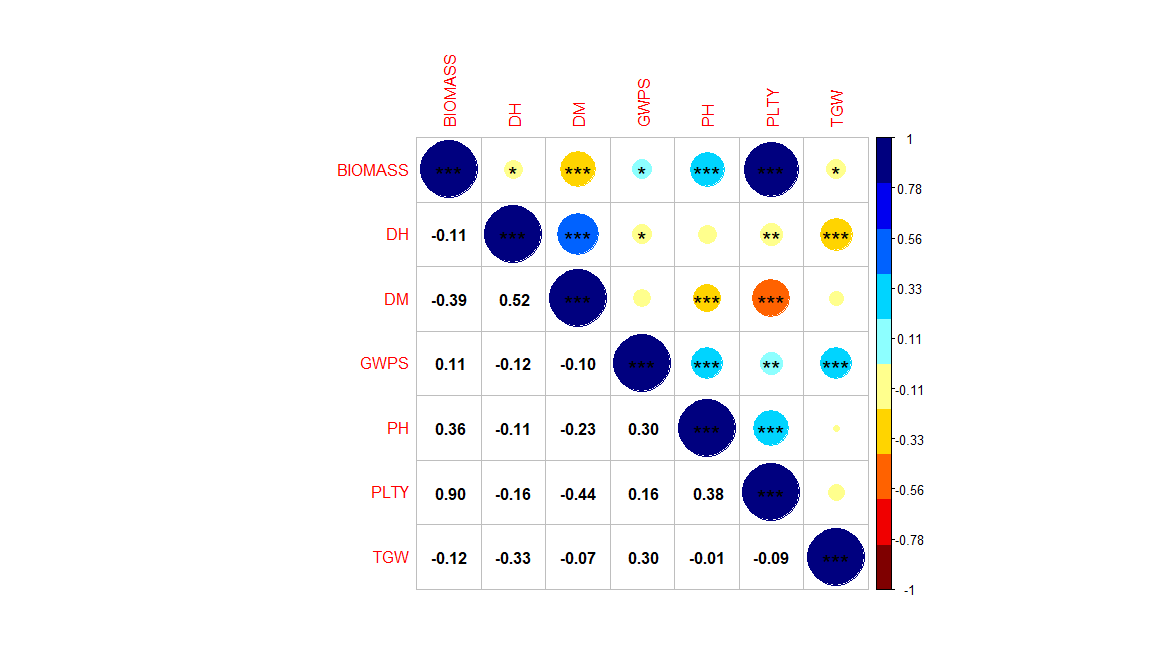

Supplement: Supplementary file 2 [file Data_Sheet_2.ZIP › Supp.Figure 2.docx]
